# A Systematic and Practical Framework on Gender and Sexual Diverse (GSD) Health for Internal Medicine Residents

**DOI:** 10.15766/mep_2374-8265.11535

**Published:** 2025-06-17

**Authors:** Sebastian Suarez, Emily Lupton Lupez, Katherine L. Modzelewski, Chad Hinkle, Carl G. Streed, Jennifer Siegel

**Affiliations:** 1 Associate Program Director, UM/JMH Internal Medicine Residency Program, University of Miami Miller School of Medicine; 2 Assistant Professor of Medicine, Section of General Internal Medicine, Department of Medicine, Boston University Chobanian and Avedisian School of Medicine; 3 Associate Program Director, Internal Medicine Residency Program, Department of Medicine, Boston University Medical Center; 4 First-Year Fellow, Section of Infectious Diseases and Global Health, Department of Medicine, The University of Chicago; 5 Associate Professor of Medicine, Section of General Internal Medicine, Department of Medicine, Boston University Chobanian and Avedisian School of Medicine; Research Lead, Gender Care Center, Boston Medical Center; 6 Medical Director, Transgender Health Program; Primary Care Program Director, Internal Medicine Residency Program, Massachusetts General Hospital

**Keywords:** Gender and Sexual Diverse, Pre-Exposure Prophylaxis, PrEP, Gender Equity, Health Equity, Human Sexuality, LGBTQ+ Health, Internal Medicine, Case-Based Learning

## Abstract

**Introduction:**

Internal medicine (IM) residents often lack the knowledge, comfort, and competency to care for gender and sexual diverse (GSD) patients, contributing to health disparities. We developed a practical, evidence-based framework providing residents with a systematic, stepwise approach to addressing GSD health needs.

**Methods:**

The curriculum was implemented at an urban safety-net hospital for 46 IM residents. Two 2-hour sessions utilized a case-based, small-group, interactive scenario of a transgender woman requiring screening and treatment of sexually transmitted illnesses (STIs), pre-exposure prophylaxis (PrEP) prescription, and gender-affirming hormone therapy (GAHT) initiation. One-page handouts with step-by-step instructions were provided. Surveys evaluated knowledge, attitudes toward the importance of GSD care, and perceptions of confidence in performing specific skills related to GSD care. Wilcoxon signed-rank tests were used to compare pre- and postsession answers.

**Results:**

Residents’ perceived confidence in providing GSD sexual health care and prescribing PrEP and GAHT increased after the sessions (*p* < .001). Attitudes regarding the importance of obtaining comprehensive sexual histories, discussing gender identity, assessing gender dysphoria, and offering GAHT for GSD patients also increased (*p* < .001). The median number of correct responses to five knowledge-based questions increased from 1 to 4 (*p* < .001).

**Discussion:**

This curriculum improved residents’ perceptions of the importance of providing GSD care, as well as their knowledge and confidence in clinical skills related to STI screening and treatment, PrEP prescription, and GAHT initiation. This curriculum offers IM residents one of the first systematic frameworks, with immediately useable materials, to address GSD health.

## Educational Objectives

By the end of this activity, learners will be able to:

1. Identify the differences between sex, gender identity, gender expression, and sexual orientation.
2. Obtain an inclusive medical history using language to avoid assumptions about gender and sexual diverse (GSD) patients.
3. Obtain a sexual history using the 7 P's: permission, partners, practices, protection from sexually transmitted illnesses (STIs), prior STIs, pleasure/pain, and pregnancy.
4. Describe the screening strategies for STIs using an inclusive approach.
5. Recognize the most up-to-date therapeutic regimens for gonorrhea and chlamydia.
6. Describe the indications, prescription regimens, and monitoring parameters for pre-exposure prophylaxis (PrEP) to prevent infection with human immunodeficiency virus (HIV).
7. Adapt an evidence-based eight-step approach on how to prescribe gender-affirming hormone therapy (GAHT) for patients with gender dysphoria.

## Introduction

Gender and sexual diverse (GSD) patients, including those who identify as lesbian, gay, bisexual, transgender, queer, and intersex (LGBTQI+), face significant health inequities.^1^ These disparities are exacerbated by clinicians' insufficient knowledge, comfort, and competency in providing care to GSD patients. GSD patients often have to educate health care providers about their own needs,^2,3^ which deters them from engaging with the health care system.^4^ In a 2022 US transgender survey, nearly half of transgender individuals reported having negative experiences with health care providers, including refusal of care, misgendering, verbal abuse, or physical mistreatment.^5^

Availability and content of medical school curricula on GSD health remain variable,^6,7^ leading to differences in knowledge and perceived competency in caring for GSD patients.^8–11^ A dearth of curricula in residency programs is also evident.^12–14^ The educational gap is especially pronounced in transgender health and gender-affirming care,^15^ with almost no published curricula specifically targeting internal medicine (IM) residents.^16,17^ Since many IM residents will serve as primary care providers (PCPs) for GSD patients, addressing these gaps is crucial. The American College of Physicians recommends that IM residency programs “incorporate LGBT health issues into their curricula,”^18^ but the ACGME has yet to provide specific guidelines, stating only that residents should meet the competency of “respect and responsiveness to diverse patient populations.”^19^ Given the lack of curricula in residency programs and limited faculty with expertise in GSD health, there has been a “call to action” for graduate medical education to enhance LGBTQI+ health education.^13^

Current published curricula tend to be siloed into individual topics, such as pre-exposure prophylaxis (PrEP) for men who have sex with men.^20^ In *MedEdPORTAL*, most of the published curricula focused on GSD health are targeted to medical students, and are primarily introductory and didactic in format.^9,20–22^ There are some curricula targeting IM residents, with one program focused on teaching sexual history taking and screening for sexually transmitted infections (STIs),^23^ and another on health promotion and disease prevention for GSD patients.^24^ These educational interventions involved mostly passive learning, such as lectures, and did not develop standardized clinical approaches.^9,12,21,22,25^ Curricula using standardized patients (SPs) have focused on teaching sexual history taking^26^ or assisting family medicine residents in formulating hormone-prescribing plans.^27^ The only curriculum involving real patients had residents take sexual histories, provide counseling, and prescribe PrEP,^28^ which increased the residents’ self-reported confidence and comfort.

We previously conducted a needs assessment among IM residents at our institution that revealed varying levels of knowledge, comfort, and perceived competency in caring for GSD patients, while also identifying implementation facilitators, barriers, and preferred educational methods.^29^ We designed the instructional approach of our curriculum based on the preferences reported by residents in our needs assessment. Residents specifically preferred small-group discussions and case-based learning, the former promoting critical thinking and participation,^30^ and the latter encouraging self-directed learning and integration into practice.^31^ Based on these survey results and considering the developmental stage of IM residents, we developed a novel, systematic, stepwise curriculum grounded in the World Professional Association for Transgender Health (WPATH) Standards of Care for Transgender and Gender Diverse People, version 8 (SOC-8)^32^ guidelines and the AAMC competencies to address GSD health needs comprehensively. This curriculum emphasizes concrete, high-value skills tailored for busy residents, supported by a user-friendly handout as a practical reference for infrequent GSD patient encounters.

## Methods

### Setting

We developed a curriculum at Boston Medical Center (BMC), an urban safety-net hospital in Boston, Massachusetts. BMC is committed to delivering exceptional care, particularly to vulnerable populations, as evidenced by its GenderCare Center, which provides care to transgender and gender-diverse patients. Similarly, the IM residency program at BMC recruits residents with a strong social mission and a history of community engagement. Target learners consisted of residents in the IM residency program, with 46 residents in each postgraduate year over a span of 3 years.

### Curriculum Design

The curriculum was collaboratively designed by a team of internists with diverse clinical and academic expertise in GSD health, primary care, medical education, infectious diseases, and endocrinology. We designed this curriculum using Kern's six-step approach.^33^ First, we identified a lack of GSD health education in the residency program. An interest group was created among residents and faculty to discuss gaps and priorities. Then, we completed a targeted needs assessment,^29^ which provided unique insights to implement a learner-centered and developmentally appropriate curriculum. We developed objectives using the AAMC competencies to create a systematic framework on practical skills to care for GSD patients. With our survey results, the educational strategy was selected based on the residents’ preferences for small-group, case-based discussions during protected time. The 2-hour case-based, interactive sessions were implemented for an entire cohort of IM residents. All participants completed pre- and postsession surveys that evaluated changes in knowledge and perceptions regarding GSD health care. Participants were asked for feedback on the sessions.

Through an iterative process, we selected learning objectives (Figure) based on our needs assessment results,^29^ the AAMC Professional Competencies to Improve Health Care for People Who Are or May Be LGBT, Gender Nonconforming, and/or Born With DSD,^34^ and prior work previously published in *MedEdPORTAL*.^24^ The curricular content was specifically tailored for general internists and PCPs, who commonly serve as primary providers for GSD patients. The content to address the learning objectives was primarily derived and adapted from the Fenway Guide to Lesbian, Gay, Bisexual, and Transgender Health,^35^ the CDC guidelines on taking a sexual history,^36,37^ STIs,^38^ and PrEP,^39^ the WPATH SOC-8 (new compared to most curricula),^32^ the University of California, San Francisco (UCSF) Guidelines for the Primary and Gender-Affirming Care of Transgender and Gender Nonbinary People,^40^ and the Johns Hopkins Quick Guide to Hormone Therapy for the PCP.^41^

**Figure.**
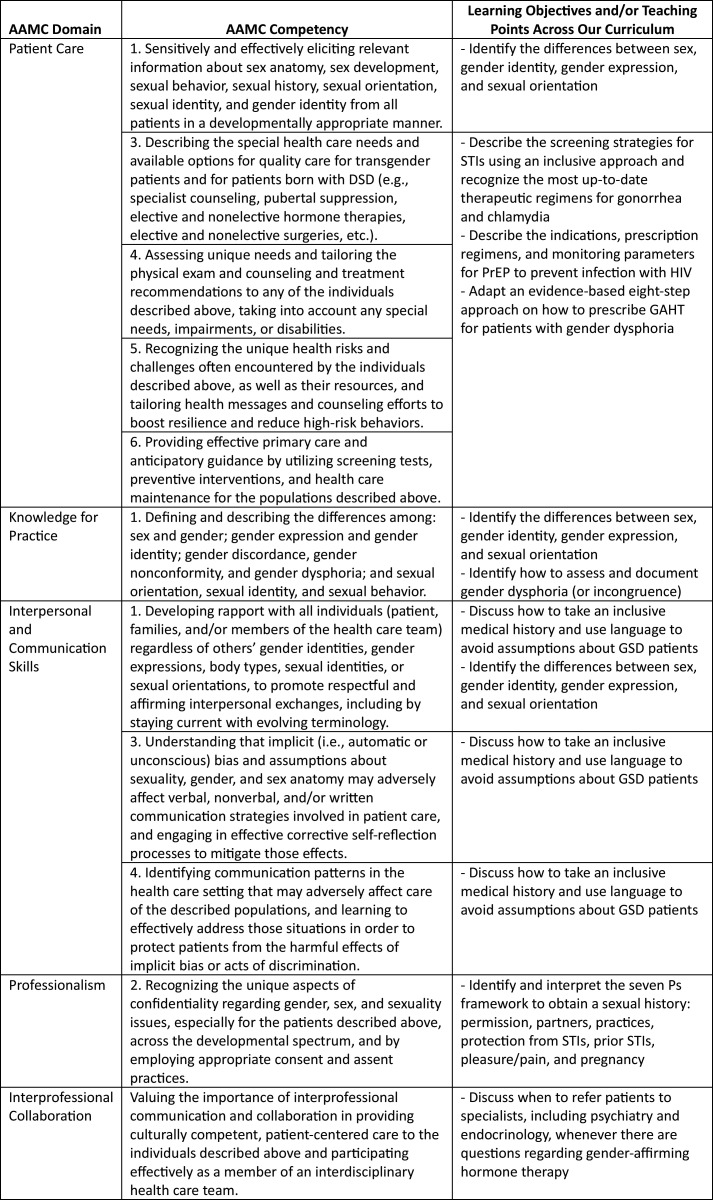
Learning objectives linked to the AAMC Competencies to Improve Health Care for People Who Are or May Be LGBT, Gender Nonconforming, and/or Born With DSD. Abbreviations: DSD, differences of sexual development; STI, sexually transmitted infection; PrEP, pre-exposure prophylaxis; GAHT, gender-affirming hormone therapy; GSD, gender and sexual diverse.

The first author (Sebastian Suarez) performed an extensive review of the literature and developed the framework used in this curriculum. Senior authors (Jennifer Siegel and Katherine L. Modzelewski) with expertise in GSD health and endocrinology further reviewed the curriculum for accuracy and clinical relevance. The curriculum consisted of two 2-hour sessions centered on a clinical scenario involving a transgender woman with diverse health care needs, including STI screening and treatment, PrEP prescription, and gender-affirming hormone therapy (GAHT) initiation. To foster an environment conducive to active learning and engagement, this partly learner-led curriculum was designed so that more than 50% of the time was allocated to activities such as small-group discussions, think-pair-share exercises, problem-based learning–style interactions, multiple-choice questions, and interactive activities. To aid learners and facilitate spaced learning and repetition, two concise one-page documents were created to include the framework (Appendix A and Appendix B), offering practical step-by-step instructions on how to address GSD sexual health and GAHT needs.

### Curriculum Implementation

A step-by-step implementation strategy for this curriculum is included in the Facilitator Guide (Appendix C). We used PowerPoint (Microsoft Corporation, Redmond, Washington) to portray the curricular content and activity prompts using basic audiovisual equipment. The first 2-hour session (Appendix D) was conducted in February 2022 for all IM interns during academic half-day, a dedicated and mandatory educational block for all residents during their ambulatory weeks (BMC follows an X+Y scheduling model). We chose interns because the initial session covered topics essential to sexual health, which are universally applicable to all patients during routine annual examinations. The second 2-hour session (Appendix E), conducted in February and March 2023, targeted the same cohort of residents during their PGY-2, when they possessed foundational knowledge allowing discussions on gender dysphoria and GAHT. The Institutional Review Board at Boston University Medical Campus and BMC considered the study exempt since it constituted a routine educational activity within an established setting.

Facilitators were senior residents (Emily Lupton Lupez and Chad Hinkle) and a chief medical resident (Sebastian Suarez) at BMC, all with special interest in GSD health and experience in medical education. Sessions were attended by preceptors with expertise in GSD health and medical education (Jennifer Siegel and Katherine L. Modzelewski).

### Evaluation

We evaluated each session with anonymous and voluntary 7-minute–long surveys (Appendix F and Appendix G) that explored knowledge, attitudes toward the importance of providing GSD care in primary care, and perceptions of confidence in performing specific skills related to GSD care. These surveys were collaboratively created by the lead author (Sebastian Suarez) and reviewed by faculty members specializing in GSD health (Jennifer Siegel) and endocrinology (Katherine L. Modzelewski) to ensure content validity. Due to the absence of validated questionnaires in the literature, we adapted questions from previous studies and modified to align with the curriculum's learning objectives.^20,24,42,43^ Attitudes toward the importance of providing GSD care in the clinic and perceptions of confidence in performing specific skills related to GSD care were evaluated using 5-point Likert scales (ratings based on degree of importance [1 = *not important*, 5 = *extremely important*] and level of agreement [1 = *strongly disagree*, 5 = *strongly agree*]), with questions related to gender and sexuality, sexual health, PrEP prescription, and initiation of GAHT. Knowledge of STI screening, PrEP, and GAHT was assessed through multiple-choice questions. Feedback was requested with open-ended questions. Presession surveys were emailed to residents 1 week before each session, with reminders sent 2 days prior to each session. To increase response rates, a QR code was displayed on screen before the session began. At the end of the session, another QR code was shown with identical postsession surveys.

### Statistical Analysis

Descriptive statistics were analyzed using the RStudio program. As data were non-normally distributed, a Wilcoxon signed-rank test was employed to compare pre- and postsession answers. Reliability was not evaluated given the small number of respondents per item. Responses to open-ended questions were analyzed using content and theme analysis, determining the frequency and percentage of responses in each theme.

## Results

Of the 46 eligible IM residents, 42 (91%) participated in the first session, of whom 41 (98%) completed the presession survey and 34 (81%) completed the postsession survey. Additionally, 41 residents (89%) participated in the second session, of whom 39 (95%) completed the presession survey and 40 (98%) completed the postsession survey. Four residents (9%) were in the primary care track, while three residents (6%) did not disclose their track, and the rest were in the categorical track. Nine residents (22%) identified as lesbian, gay, bisexual, transgender, or queer or as a GSD individual.

On the presession survey from the first session, when participants were asked if they routinely take a comprehensive sexual history, 11 respondents (27%) disagreed, 19 (46%) were neutral, 11 (27%) agreed, and 1 (2.4%) did not respond. When asked if they feel more comfortable discussing sexual history with a cisgender, heterosexual patient than with a GSD patient, 8 respondents (20%) disagreed, 19 (46%) were neutral, 14 (34%) agreed, and 1 (2.4%) did not respond.

Outcome data represented Kirkpatrick level 2 evaluation of learning. From pre- to postsession, there was a significant increase in Likert-scale scores regarding the residents’ perception, as a PCP, of the importance of taking a GSD patient's comprehensive sexual history and discussing gender identity, assessing gender dysphoria, and offering GAHT to GSD individuals (Table 1). Similarly, after the sessions, there was a significant increase in residents’ confidence in their ability to adequately care for GSD patients.

**Table 1.**
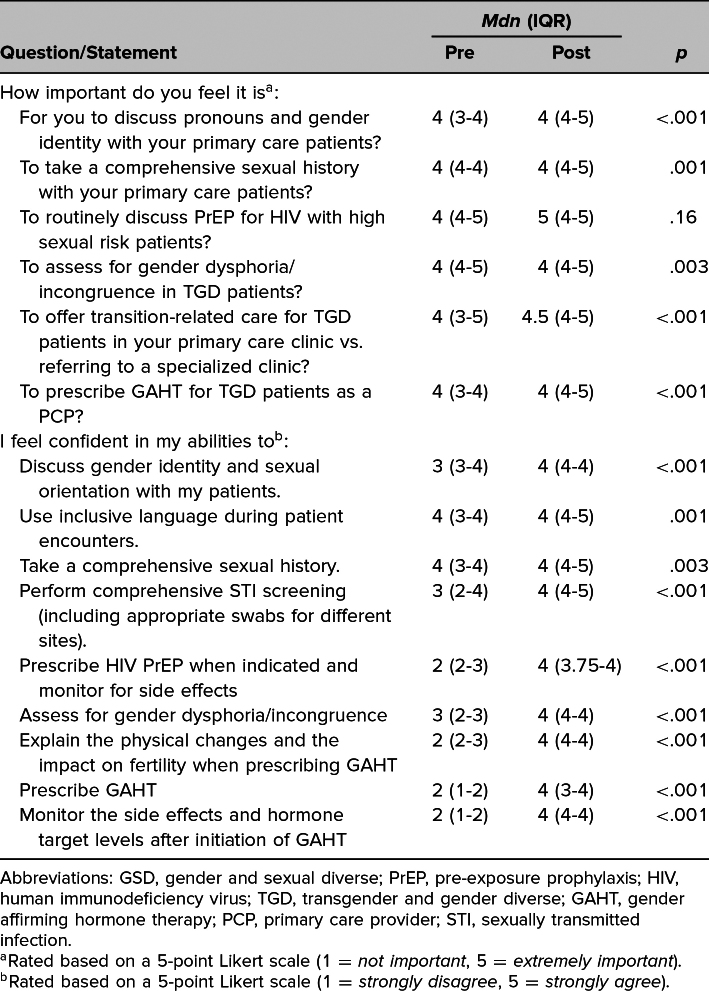
Participant Likert-Scale Scores on Attitudes Regarding the Importance of Providing GSD Care in Primary Care and Perceptions of Confidence in Performing Specific Skills Related to GSD Care

When the IM residents completed the knowledge-based, multiple-choice portion of the surveys, the pre- and postsession median scores remained unchanged across the five questions. However, the Wilcoxon signed-rank test showed statistically significant changes in median scores after the sessions (Table 2). The median number of correct responses to the five knowledge-based questions increased from 1 to 4 (*p* < .001; Table 2). This suggests that while the central tendency did not change, individual experiences increased toward the median, and the variance decreased.

**Table 2.**
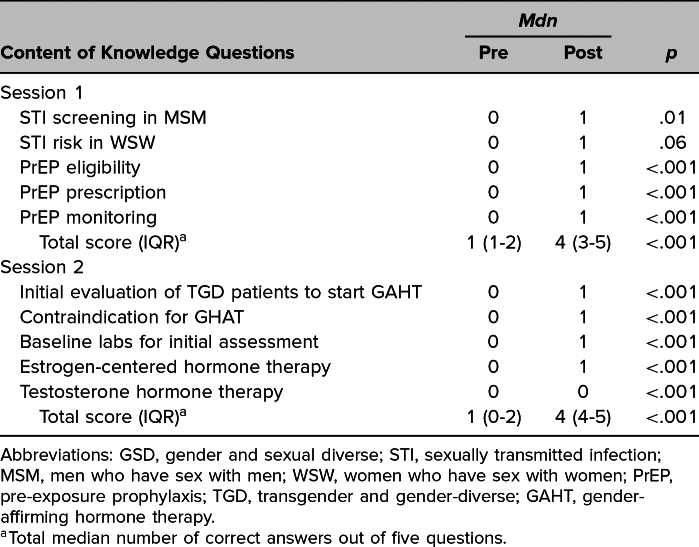
Median Number of Participant Correct Answers to Knowledge Questions on GSD Health

For the first and second sessions, 97% and 100% of residents, respectively, either agreed or strongly agreed that the sessions were clearly presented and well organized, and that the instructors were knowledgeable and prepared.

When asked to rate the utility of the session as IM residents, 24 residents (71%) rated the first session as *excellent* and 8 (24%) as *very good*. All residents either strongly agreed or agreed that the content of the first session would improve their communication with GSD patients. For the second session, 19 (48%) rated it as *excellent* and 19 (48%) as *very good*, while 1 (3%) rated the session as *average* and 1 (3%) as *poor*. Among the survey respondents, 26 (65%) strongly agreed, 13 (33%) agreed, and 1 (3%) strongly disagreed that the second session would improve their ability to prescribe GAHT for transgender and gender-diverse patients.

Through open-ended responses, residents suggested improvements, including adding more interactive components, cases, and questions; receiving the handout prior to the session; involving faculty with more patient care experience; and incorporating real patients to apply what was learned.

## Discussion

After implementing this evidence-based, practical step-by-step approach on GSD health for IM residents, there was an immediate increase in residents’ perception of the importance of providing GSD care, as well as in their knowledge and confidence in clinical skills related to STI screening and treatment, PrEP prescription, and GAHT initiation. Before the curriculum, most IM residents did not feel comfortable discussing GSD health issues with their patients, and many felt either neutral or more comfortable discussing these topics with cisgender, heterosexual patients than with GSD patients. Even in our program with faculty expertise and high rates of residents self-identifying as GSD, residents had poor knowledge of and discomfort with GSD care, suggesting a similar baseline state as that of residents elsewhere. One advantage of implementation was the lack of resistance from leadership, along with residents’ high awareness, which helped minimize biases during the session.

One of our key successes was the improvement in residents’ confidence in both their communication skills and their ability to prescribe PrEP and GAHT for GSD patients. We believe this could be partly attributed to the instructional approach that aligned with the needs assessment and focused on practical application of knowledge. By focusing on both communication skills and medical management of GSD health, we equipped residents with essential skills to provide holistic and compassionate care to GSD patients. Similarly, we provided residents with a user-friendly handout that served as a consolidated resource and framework for patient encounters, enabling them to focus on understanding the framework rather than memorizing it. Anecdotally, residents reached out to the instructors to share that they were using the framework and handout to prescribe PrEP and monitor GAHT in their clinic, and that the sessions were pivotal in empowering them to provide GSD care without referring to specialty clinics. Although anecdotal, it suggests some durability to the curriculum and reflects the benefit of providing materials that residents could put to immediate use in clinic. Some residents even asked if they could share the handout with people outside the institution.

This curriculum was included in mandatory educational blocks for all residents, which likely contributed to the high response rates, enhancing the internal validity of our study. As the curriculum was implemented by a chief medical resident and senior residents with interest in the topic, it potentially allows for other training programs to adapt the implementation. Similarly, our curriculum serves as a practical resource to support situations where cost or feasibility (e.g., absence of a GSD health–centered clinic or infrequent patient encounters related to GSD health) prevent the implementation of other educational strategies. If adapted at institutions with less diversity of expertise, we suggest inviting a patient who identifies as GSD to share their story about discrimination in the health care system as well as in other aspects of their lives, which could foster empathy and enhance learning. Residents in settings without GSD experts can leverage online resources like the WPATH or UCSF guidelines, collaborate with interdisciplinary teams including social workers and psychologists who may have additional resources, and refer to endocrinologists who are more likely to have the necessary training to support GSD patients.

This curriculum has limitations. Since there were no validated questionnaires available when the curriculum was created, we used prior study surveys as references.^24^ Future iterations should refine the questionnaire to enhance question quality and align with best practices, such as avoiding negatives and ensuring choices are mutually exclusive. Additionally, we intentionally limited survey length to encourage participation and completion rates. We did not include doxycycline postexposure prophylaxis (doxy PEP) since the CDC guidelines were published after our curriculum was developed. However, the current curriculum is adaptable to updates in best practices, such as doxy PEP. Future iterations should incorporate more multiple-choice questions and interactive components throughout the sessions. Similar to the experience of others working in GSD health issues,^27^ we now recognize that using terms like “masculinizing” and “feminizing” are not specific and may perpetuate gender stereotypes. We recommend using gender-neutral language (e.g., testosterone therapy and estrogen-centered therapy) instead of language focused on the effect that hormones may have on a patient's body.

As a single-site study with a gender care center, the findings should be generalized with caution and adapted to local contexts that have different expertise in this subject. Delivering this curriculum without prior GSD health experience may be challenging. To support implementation, we included a detailed facilitator guide. Future efforts could include the development of facilitator trainings or a CME module. The short-term nature of our assessment made it difficult to evaluate whether the changes in our outcomes were sustained with time. Furthermore, we did not measure whether the curriculum impacted residents’ skills, practices, or GAHT prescription behaviors, although we did hear anecdotal reports from residents that were encouraging. The impact could be further studied with qualitative research that evaluates the residents’ experiences of using the framework and handout in their clinical encounters.

In the future, this curriculum could be expanded to include additional modules focused on surgical options and preventive health of GSD patients. It could also include SPs or direct observation in a clinic dedicated to GSD patients, as has been done in other studies.^26–28,44,45^ Given the robust evidence supporting the use of SPs in other areas of medical education, their inclusion in future GSD health curricula should be a priority. Curriculum designers have an opportunity to actively engage GSD SPs and ensure appropriate compensation when doing so. By distributing this curriculum across the US, we can provide targeted and practical GSD health education to a diversity of IM and family medicine residency programs. Lastly, this curriculum can be used to train faculty who may currently lack knowledge in this area, so that they can better support learners and care for GSD patients. A “train the trainer” approach, as employed previously,^24^ could be particularly effective in achieving this goal.

In conclusion, this evidence-based, practical framework increased IM residents’ knowledge, confidence in GSD health clinical skills, and perception of the importance of GSD care. The curriculum, grounded in the AAMC competencies, along with a user-friendly handout, could provide a quick and practical resource for real-life patient encounters.

## Appendices


GSD Health Handout.pptxGAHT Handout.pptxFacilitator Guide.docxGSD Health - Part 1.pptxGSD Health - Transgender Health.pptxGSD Health Survey.docxTGD Health Survey.docx
*All appendices are peer reviewed as integral parts of the Original Publication.*
