# Supplementary material for: A Systematic and Practical Framework on Gender and Sexual Diverse (GSD) Health for Internal Medicine Residents
Source: MedEdPORTAL. 2025 Jun 17;21:11535. doi: 10.15766/mep_2374-8265.11535 (PMC12170925; doi:10.15766/mep_2374-8265.11535)
Supplement: Supplementary file 1 — GSD Health Handout.pptxGAHT Handout.pptxFacilitator Guide.docxGSD Health - Part 1.pptxGSD Health - Transgender Health.pptxGSD Health Survey.docxTGD Health Survey.docx [file mep_2374-8265.11535-s001.zip › C. Facilitator Guide.docx]

**Appendix C**

A Systematic and Practical Framework on Gender and Sexual Diverse (GSD)

Health for Internal Medicine Residents

Facilitator Guide

**Overall Goals**

Internal medicine (IM) residents often lack knowledge, comfort, and competency needed to care for gender and sexual diverse (GSD) patients (commonly known as LGBTQI+), contributing to health disparities. We created and implemented a practical, evidence-based framework that provides residents with a systematic and stepwise approach to address GSD health needs.

This is the facilitator guide to lead the large-group curricular sessions and small group sessions. Facilitators can use the guide to use before and during each session. Only one facilitator is needed for each session, but having two facilitators helps with group activities.

**Target learners**

- First session: All IM interns during academic half-day, a dedicated and mandatory educational block for all residents during their ambulatory weeks
- Second session: The same cohort of residents during their PGY2, when they possessed foundational knowledge to allow discussions on gender dysphoria and GAHT

**Curriculum Objectives**

By the end of this activity, learners will be able to:

1. Identify the differences between sex, gender identity, gender expression, and sexual orientation
2. Obtain an inclusive medical history using language to avoid assumptions about gender and sexual diverse (GSD) patients
3. Obtain a sexual history using the “7 Ps”: permission, partners, practices, protection from sexually transmitted illnesses (STIs), prior STIs, pleasure/pain, and pregnancy
4. Describe the screening strategies for STIs using an inclusive approach
5. Recognize the most up-to-date therapeutic regimens for gonorrhea and chlamydia
6. Describe the indications, prescription regimens, and monitoring parameters for pre-exposure prophylaxis (PrEP) to prevent infection with human immunodeficiency virus (HIV)
7. Adapt an evidence-based eight-step approach on how to prescribe gender-affirming hormone therapy (GAHT) for patients with gender dysphoria.

**Pre-training reading materials**

Learners will not have any pre-training reading materials. The content for the curriculum is included in this facilitator guide, as well as in the speaker notes for the individual sessions. However, if the facilitator does not have any baseline knowledge on the GSD health, it is recommended that they read the materials below prior to delivering the curriculum. These references would also be the ones recommended for residents who wish to expand more on their clinical skills.

First session:

1. Centers for Disease Control and Prevention. A Guide to Taking a Sexual History. Atlanta, GA: US Department of Health and Human Services, CDC. Available from: <https://stacks.cdc.gov/view/cdc/12303>
2. Suarez S, Lupez E, Siegel J, Streed C. The Annual Examination for Lesbian, Gay, and Bisexual Patients. Primary Care: Clinics in Office Practice. 2021;48(2):191-212.
3. Centers for Disease Control and Prevention. *Sexually Transmitted Diseases Treatment Guidelines, 2021.* U.S. Department of Health and Human Services;2021. Available from: <https://www.cdc.gov/std/treatment-guidelines/STI-Guidelines-2021.pdf>
4. Centers for Disease Control and Prevention: US Public Health Service. *Preexposure Prophylaxis for the Prevention of HIV Infection in the United States – 2021 Update: A Clinical Practical Guideline.*  2021. Available from: <https://stacks.cdc.gov/view/cdc/112360>

Second session:

1. UCSF Gender Affirming Health Program-Department of Family and Community Medicine-University of California San Francisco. Guidelines for the Primary and Gender-Affirming Care of Transgender and Gender Nonbinary People. 2nd edition. Deutsch MB, ed; June 2016. Available from: transcare.ucsf.edu/guidelines
2. Coleman E, Radix AE, Bouman WP, et al. Standards of Care for the Health of Transgender and Gender Diverse People, Version 8. Int J Transgend Health. 2022;23(Suppl 1):S1-s259.
3. Hedian HF, Norwood A, Siegel J, Loeb D. Transgender and Gender Diverse Hormone Therapy Johns Hopkins University2023. Available from: <https://www.hopkinsmedicine.org/-/media/center-for-transgender-health/documents/tgd-gaht-quick-guide.ashx>

**Curriculum Materials**

- Computer and projector for a PowerPoint Presentation
- Optional: copies of blank tables to fill out during breakout sessions (see Presentation Script below)
- Facilitator Guide
- 1 digital copy of Appendix D or E (PowerPoint presentation), with any modifications.
- Access to Qualtrics or other survey-creation software to conduct pre- and post- session surveys (Appendices F or G) electronically.
- 1 printed copy of Appendix A or B for facilitator reference during the session
- 1 digital copy of Appendix A or B to send via email to learners at the end of the session

**Promoting a safe and inclusive learning environment**

Creating a safe and supportive learning environment is essential when teaching residents about GSD health, especially in institutions that may not have established inclusive policies or cultures. Below are strategies that residency programs can implement to promote a learning environment that supports the development of competency in GSD health, regardless of institutional inclusivity.

1. Establish Ground Rules: Set clear expectations for respect, confidentiality, and open-minded discussion at the start of any teaching session. Encourage psychological safety by reinforcing that questions and uncertainties are welcome and that everyone is here to learn.
2. Acknowledge that mistakes will be made: it should be a safe space to make those mistakes that will be addressed as a group
3. Incorporate Simulation Learning: Using role-playing or case discussions allows residents to practice communication and clinical skills in a controlled, low-risk setting before engaging with real patients.
4. Identify faculty or staff champions: Faculty who can model inclusive practices and mentor residents helps reinforce learning and provide support
5. Faculty education: If no champions found at the institution, support a faculty member with interest to train at national or international trainings/courses on GSD health.
6. Highlight Institutional Policies: Highlighting existing non-discrimination policies, accreditation requirements, or professional organization guidelines can provide backing for this training.
7. Encourage Self-Reflection: Providing space for residents to reflect on their own biases, experiences, and challenges fosters growth. Maybe even doing additional sessions on self-social and global biases.
8. Community engagement: invite members of the community to talk about their experiences with the healthcare system and examples of discrimination, to start building trust and empathy towards a minoritized population
9. Acknowledge uncertainty: if there is ever a situation where a resident asks the questions that the facilitator does not know the answer to or feels uncomfortable, the facilitator should acknowledge this, and mention to the group that they will find out the answer and get back to them. They can also assign somebody from the group to look up the answer to the question.

When residents exhibit behaviors that are consciously or subconsciously non-inclusive, it’s important to address the situation directly and immediately, but in a way that fosters learning (calling in) rather than shutting down (calling out) dialogue. Below are a few strategies:

1. "Pause and Reflect" Approach: If a resident makes an inappropriate or biased remark, pause and ask: “Can you clarify what you meant by that?” or “How do you think a GSD patient might perceive that statement?”. This encourages self-reflection without immediate confrontation.
2. Frame it as a learning opportunity: If a resident makes a mistake (e.g. misgenders a person), instead of saying, “That’s wrong,”, you can say: “Using a patient’s correct name and pronouns is a standard of care that improves outcomes and trust. This is based on evidence.”
3. Using “Oops” or "Ouch": If someone misspeaks or makes an assumption, allow space for correction without excessive embarrassment. “Ouch, that comment feels hurtful”, and then continue to provide a brief explanation of why the statement or behavior was problematic.
4. Role model inclusive behavior: Demonstrate the correct way to handle mistakes. For example, if someone uses non-inclusive language, can say “just a reminder, Callie uses they/them pronouns”
5. Address challenges directly: If a resident disengages or avoids conversations, can directly ask “I’ve noticed that you seem less engaged in discussions around GSD health. Can you share your thoughts on this topic?” Validate their perspective while reinforcing professional expectations: “It’s okay if this is new for you. Our role as physicians is to provide the best care for all patients, and that includes learning about populations we may not personally identify with.”
6. Set clear expectations and accountability: Reinforce that inclusivity is not optional but a professional standard.

If problematic behavior persists, escalate appropriately within the residency program leadership or the resident’s advisor.

**Follow up, adaptation, and evaluations**

Whenever this curriculum is adapted, we recommend adding doxycycline post exposure prophylaxis (Doxy PEP). We initially did not include this topic since the CDC guidelines were published after our curriculum was developed. We also recommend using gender-neutral language. For example, instead of using terms, masculine and feminizing hormone therapy that can perpetuate gender stereotypes, we recommend using testosterone therapy and estrogen-centered therapy, which is more specific to the treatment and not on the effect that hormones may have in a patient’s body. A third module can be added for residents in their third year that is focused on surgical options and preventative health (e.g. cancer screening).

In terms of evaluation, future iterations should try and incorporate whether resident skills practices or behaviors changed and were sustained over time since our outcomes cannot measure this. Other researchers can also incorporate qualitative data regarding resident experiences in clinic. If using a similar questionnaire, the quality of questions should be enhanced and aligning with best practices such as avoiding negatives and ensuring choices are mutually exclusive. Pre-post session surveys can be deployed using QR codes at the beginning and at the end of the session to enhance response rates, such as the ones obtained in our study.

**Suggested Agenda and Timeline**

The duration of each session is approximately 120 minutes (2 hours), for a total of 4 hours for this curriculum. An overview of the sessions is provided in the tables below. The facilitator should attempt to adhere to the timeline.

First Session (Appendix D):

| **Section** | **Slides** | **Duration** |
| --- | --- | --- |
| Deployment of pre-session survey (Appendix F) |  | X minutes |
| Introduction and discussion on differences Between sex, gender, identity, gender expression, sexual orientation | 1-6 |  |
| Taking an inclusive sexual history and gender-neutral language | 7-11 |  |
| Getting a sexual history using the “7 Ps” approach   - Breakout session #1 | 12-26 |  |
| STI screening strategies and up-to-date guidelines for treatment of gonorrhea and chlamydia   - Breakout session #2 | 27-37 |  |
| Indications, regimens, and monitoring parameters of PrEP for prevention of HIV | 38-46 |  |
| Conclusion and digital sharing of Appendix A | 47 |  |
| Deployment of post-session survey (Appendix F) |  |  |

Second Session (Appendix E):

| **Section** | **Slides** | **Duration** |
| --- | --- | --- |
| Deployment of pre-session survey (Appendix G) |  | X minutes |
| Introduction to gender identity, transgender and gender diverse patients, and GAHT | 1-15 |  |
| Initial evaluation prior to collection of orders and initiation of treatment   - Breakout session #1 | 16-33 |  |
| Prescribing GAHT and follow-up   - Breakout session #2 | 34-62 |  |
| Conclusion and digital sharing of Appendix B | 63 |  |
| Deployment of post-session survey (Appendix G) |  |  |

**Presentation Script**

Review curriculum slides and update and change if necessary. Slides with questions intended for the audience will be marked with an asterisk (*). Instructions on the purpose and what to expect from each answer in the script.

First session:

| **Slide Number** | **Facilitator Script** |
| --- | --- |
| 1 | Introduce this topic as something that is applicable to all patients that we will see in clinic, and that there is specific clinical material relevant to demographic populations such as the gender and sexual diverse population, many times referred as the LGBTQI+ population, or lesbian, gay, bisexual, transsexual, queer, intersex, and others  The orange color of the rainbow flag represents “healing” , which was the reason why the entire presentation is formatted using the orange color, which is appropriate for us in the healthcare field |
| 2 | [Read the case] |
| 3* | [Read the question]  This question is trying to assess whether learners are able to identify how to address somebody who may be transgender or gender diverse. You can ask learners about different ways. They would approach the situation as there is no one right answer. This question serves more as an opening statement to this learning objective. Appropriate ways will be divergent later in the slides. |
| 4* | Use the animations and after the patient says that they want to be called Callie, the next animation, prompt learners to think what they would say next (*), especially if they realize that there may be a difference between sex and gender. One strategy is to ask the patient what pronouns they use, in this situation Callie refers that she uses she her hers.  Acknowledge to learners that this conversation does not need to happen at the beginning of the encounter necessarily. Gender identity may come up at any point during the encounter.  For example, I self-disclose that I usually don’t share my pronouns at the beginning of encounters. Some patients may feel forced to out themselves, and some may not feel this is safe. I allow it to emerge through the conversation. At the same time, other physicians routinely introduce themselves with their pronouns. This may be an opportunity to spark discussion of what works best for your learner group.  We should mention that this is an opportunity to advocate for clinics to have more inclusive forms and mention that some surveys have shown that GSD patients prefer to disclose identities in private ways such as a form instead of face to face  Ask residents if they have successfully elucidated pronouns and share whenever it was uncomfortable too.  Some clinicians have their pronouns in their badge, or even just the flag to make pts comfortable, which studies have supported  The facilitator should note that current federal regulations require that all EHRs must have SOGI (sexual orientation and gender identity) data if they are receiving any funding from federal government, which includes the majority of hospitals. EPIC, a very common EMR, also includes this data nationally. |
| 5 | This latest meant to teach learners about basic definitions. The facilitator can click through the different animations and below they can find the explanations to each definition.  **Gender Identity**: a person's internal sense of their own gender, regardless of external appearance.^3^  *Cisgender*: person whose anatomical birth sex is congruent with gender identity  *Transgender*: person whose anatomical birth sex is incongruent with gender identity  *Genderqueer*: an umbrella term that encompasses a wide range of genders, such as those who don’t fall in the binary, as well as those who consider to have multiple genders or no gender.  **Gender expression**: a set of behaviors that have been socially assigned within a spectrum of masculine or feminine characteristics.^3^ such as actions, clothing, demeanor, and more. The way a person acts, dresses, speaks, and behaves (i.e., feminine, masculine, androgynous); it’s how we present ourselves to the world.  *Gender nonconformity*: Variation from developmental norms in gender role behavior,^4^ or people whose gender expression does not conform to society’s norm.  **Sex**: a person’s anatomical state that is commonly characterized by male and female phenotypes (e.g., genitals, sex characteristics, chromosomes, hormones). ^3^  *Differences of sex development (DSD):* phenotypes incongruent with the male vs. female binary  **Sexual Orientation**: a set of sexual attractions, behaviors and/or romantic feelings that can vary in the spectrum of sex and gender identity.^3^  Examples: gay, lesbian, bisexual, pansexual, asexual, straight  *MSM*: men who have sex with men, an epidemiologic term describing a man who has sex with men regardless of how he self-identifies  *WSW*: women who have sex with women, an epidemiologic term described a woman who has sex with women, regardless of how she self-identities. |
| 6 | [Read learning objective] |
| 7* | The facilitator should ask this question to the audience and see what their baseline knowledge is. The main point is to mention that GSD patients have many of the same medical problems and are at risk for all the same pathology as other patients. However, GSD patients have unique experiences and barriers to care that place them at higher risk for certain things including both chronic and infectious diseases, mental health disorders, and substance use disorders.  While much of a clinical encounter with a GSD patient will be like any other encounters, it’s important to be aware of the nuances in order to avoid missed opportunities to screen, detect, treat, and educate, which could further worsen health inequities.  To provide more equitable care for GSD patients, the Human Rights Campaign initiated the healthcare equality index (HEI) which is the national LGBTQI+ benchmarking tool that evaluates healthcare facilities' policies and practices related to the equity and inclusion of their LGBTQI+ patients, visitors and employees. One component of this education on GSD health. Some hospitals have HEI leadership status, meaning they follow certain recommendations that are implemented throughout the hospital. |
| 8 | GSD patients have historically faced, and currently face, interpersonal and systemic discrimination when seeking health care. Fear of discrimination often deters people from seeking care and a lack of adequate provider training facilitates inequitable care and can worsen existing disparities.  There are many barriers to equitable care including: fear of mistreatment, stigma and discrimination, and lack of clinical training  - **Animation guide**: as you mention each one of these barriers, you can click through the animation to give examples of each one of these categories, which will pop-up a box with content.  One other thing to consider depending on the state is that GSD patients more commonly lack health insurance and often don’t have benefits for same sex partners  Extra: 11% LGB reported abusive language in relation to their sexual orientation |
| 9 | The facilitator can mention that GSD patients often have a fractured relationship with healthcare providers and institutions, so it is essential that we create inclusive encounters. It’s important to note that a patient’s first impression during a health care encounter often precedes the clinician; it is influenced by physical spaces such as waiting areas and examination rooms, as well as the behavior of clinic staff. GSD patients often look for signs and cues that indicate that they are welcome including community symbols (ie, pride flag), posters with same-sex couples and transgender people, brochures on LGBTQI+ health, and staff wearing rainbow and pronoun pins.  A few ground rules that help create inclusive encounters. At this point, the facilitator can start clicking through the animations of the slide. After each one of the three points, there will be a pop-up box that explains in more detail. The slide can be read through the animations |
| 10 | Finally, if you make a mistake, acknowledge it, apologize, and ask what to do differently. You don’t have to be overly apologetic, we will all make mistakes. Most patients appreciate your honesty/humility and acknowledging a mistake can decrease the power differential. |
| 11 | [Read the learning objective] |
| 12* | [Read the case]  [Ask question]  After reading the next piece of information of her patient, the facilitator can click to the next animation to prompt the learners what else they would want to know (*).  If not brought up by residents themselves, it is important to ask them what else they want to know about the pain. If they ask, you can give the following information about the characteristics of the pain: The pain has intensity 5/10, is located in lower abdomen and radiates “down there”, worse with BMs, does not get better with anything. Has tried Tylenol with minimal relief. Associated symptoms include mild bleeding that she notices when she goes to the bathroom.  Ideally, one of the learners should ask what the patient refers or means by “down there”, at which point of facilitator should let them know that this patient means the rectal area. They don’t ask for clarifying questions to facilitator can ask them what this patient is referring to to encourage participation.  If residents ask about getting sexual history, ask why and how that is relevant. Learner should be disclosing that some patients can have rectal STIs that may present similar to this patient, which is the point of the following learning objective. However, unless they bring it up, don’t disclose anything about rectal gonorrhea, and instead move onto the next slide |
| 13 | This is just an introductory slide and the facilitator can just say that in this case, the physician is preparing to take a sexual history for the patient |
| 14* | [Ask question]  The question in the title should be an open, ended question to the learners to see how they would approach the situation and how they start conversations about taking a sexual history. Below the question is an example of an approach to starting the conversation. The three *** can be replaced for anything, but in this situation, it is supposed to replace the patient’s current presenting symptom, which is the abdominal pain.  The facilitator should mention that if the patient is uncomfortable, it is OK to stop and explain why it’s important. If the patient remains uncomfortable, it’s OK to stop and move on without getting this information in order to respect patient wishes. |
| 15 | **Breakout session #1**  This is the first breakout session of the curriculum. The facilitator should first describe what the activity is and then divide people into different groups in order to not divide their attention. The approach used is to ask them what type of questions they would include when taking a sexual history without giving them any framework to begin with. Then divide them into small groups of 4–5 people.  While they chat, divide a whiteboard in 2 columns and 6 rows (one for each “P”). Don’t write or categorize anything inside the column/rows for now  After 5-10 mins, bring the group back and ask them to share what they talked in groups. As they share the questions they would ask, it is your job to categorize them and write them according to the 6 “P’s”. Do not write the name of the categories yet.  Once they have shared their questions, share the framework for the 6 “p”s and write each P in the board.  Once you have given them, the framework and you have filled with some questions, you can add other questions that you think are pertinent. All examples of pertinent questions are included in slides 17–23, which are currently hidden. |
| 16 | This is an example of how to divide the board  Remember not to add the word “Category” at the top as illustrated until after the learners have shared examples of questions they ask. |
| 17 | The slide serves as a summary of the framework used, and we also add a 7P which is permission. Learners already should’ve asked for permission before asking all these questions which was reviewed in slide 14.  Facilitator should say that some of these questions can be challenging for non-English speaking patients and with the use of an interpreter. you might need to spell it all out. Patients are more comfortable if you’re comfortable. |
| 18 | [For facilitator reference] |
| 19 | [For facilitator reference] |
| 20 | [For facilitator reference] |
| 21 | [For facilitator reference] |
| 22 | [For facilitator reference] |
| 23 | [For facilitator reference] |
| 24* | [Ask question]  The facilitator can ask the open-ended question in the title and see how the learners would approach this. After getting their thoughts, the facilitator can click through the animations to give an example of a good approach in a situation like this. The point of this slide is to work together with the patient and provide a safe space where you ask them if they have anything they would like to know before you proceed or anything that would make them more comfortable. |
| 25* | In this slide, it’s a good idea to ask(*) the learners to list quickly the 7 “P’s” of taking a sexual history. Summarize that the objective is to give them a framework on how to take a comprehensive sexual history that is applicable to all patients. |
| 26 | [Read the learning objective] |
| 27* | [Read the case]  In this slide, the facilitator should read through the text, which explains a little more information in terms of Callie’s sexual history. Then there are four pop-ups with questions. The answers to these questions are below, but the facilitator should first prompt the audience to answer the questions (*) first based on the information available in the slide.  Based on what she’s told us:   1. What is her gender identity: transgender woman (because SHE SAID so; remember, gender ID is someone’s own internal sense of their gender, so only they can tell you what it is; being assigned male at birth and preferring female pronouns does not necessarily mean they would describe themself as transgender) 2. What is her sex: probably male assigned at birth (although we have no information about that) 3. What is her sexual orientation: unclear. We know she has sex with men but we don’t know if she has sexual/romantic attraction to men and or others. This goes back to not making assumptions and letting the patient self describe! 4. Should she get tested: Yes she should get tested: multiple partners, inconsistent condom use, hx of STI. If residents don’t mention rectal gonorrhea, don’t disclose yet |
| 28 | **Breakout Session #2**  This is the second breakout session of the curriculum people the facilitator should tell the learners that they should go back to their previous groups of 4–5 people and fill out the following table. They should be told to think about STI testing in general and not just specific to this patient. Highlight who should be screened regardless of symptoms  The facilitator should give the residents about 5–10 minutes to discuss this in their groups and fill out the table. They can either leave this table projected, or the facilitator can also print multiple copies of this table to distribute to the learners.  After time is done, the facilitator should bring residents back to main group and start filling one by one in the screen or in a whiteboard. Using animations is more difficult here because we don’t know the order in which the residents will start mentioning the different STIs. |
| 29 | There should be a complete table at the end of the exercise, which should take about 10–15 minutes to fill out together.  This table highlights those who should be screened regardless of symptoms  The facilitator should note that even though these are the indications per guidelines, it also goes without saying that anyone after an exposure, anyone at high risk, and anyone with symptoms should be tested at any period. Also, these are CDC based guidelines based in part on prevalence in certain groups. However, it’s important to remember that not all groups have same behaviors, and we shouldn’t be looking just at sexual orientation to determine STI risk, but instead risk factors.  Also notable is that women who have sex with woman (WSW) are rarely included in CDC guidelines, in part due to limited studies in this group.  Glossary of terms: HIV, human immunity deficiency virus. MSM, men who have sex with men. NAAT: Nucleic Acid Amplification Tests; they detect genetic material (nucleic acids) and amplify them (make copies) -> can detect very small amounts |
| 30 | This is a great slide to place an image of a throat/rectal swab available at the institution of the facilitator so learners get introduced to which one they have to use |
| 31 | This is a great slide to place an image of a endocervical/vaginal swab available at the institution of the facilitator so learners get introduced to which one they have to use  Facilitator should mention that endocervical swab and self-vaginal swab have very similar sensitivity (in the high 90s) so as a general rule of thumb, self swab should be offered first. If a female declines self swab, provider or RN can do swab.  Urine is not as good (low 90s), but an alternative for those women or other individuals assigned female at birth who decline vaginal/endocervical swab. Depends on the patient |
| 32 | As we said earlier, it’s important to evaluate overall STI risk instead of just using sexual orientation or gender identity groups to guide screening. High risk behaviors including substance use, multiple partners, sex w/out barrier protection, and sex work put ANYONE at increased risk.  However, there are some group or population specific considerations we should keep in mind.  The facilitator can scroll through the animations to read through the next two pop-ups:  For MSM (men who have sex with men) and trans-women: rectal mucosa is more susceptible to infection and there are documented higher rates of HIV and gonorrhea in the community. This is what drove a lot of the screening guidelines we just mentioned.  For WSW (women who have sex with women): it’s notable that STI transmission is less efficient, but BV and HSV may be more common. |
| 33* | [Read case]  The facilitator should read the next prompt about what happens next in the clinical encounter and then can scroll through the animation that asks what STI testing will need to be obtained. Essentially, the goal is to apply what was just learned in terms of the guidelines to this specific patient.  Based on the history she gave us, which testing would you want to get (*) (can get everything but what don’t you want to miss). This is to prompt residents to get rectal and throat swabs based on this patient’s specific sexual activity  Sexual hx: Callie shares that she is currently sexually active and has been with 2 male partners in the last 3 months. She recently came out as a transgender woman. She has oral and receptive anal sex. She is usually under the influence of alcohol when having sex. She uses condoms 50% of the time because of the ”heat of the moment”. She had genital herpes when she was a teenager but no other STI that she is aware of. There are certain behaviors that places her at higher risk of STIs. |
| 34 | [Read case]  In this life, the facilitator is just sharing what the results were of the swaps that were obtained, and that they should have requested to collect in the previous light |
| 35 | A lot of medical students and residents may be taught about treatment of gonorrhea and chlamydia using previous guidelines. However, in 2020 there were new guidelines for gonorrhea. The facilitator can share that previously, it was thought that dual therapy with CTX and azithromycin would in theory reduce the risk of emerging resistance to CTX and help treat the common co-infection with chlamydia. However, CTX appeared to have less resistance problems and as we will get into in a minute, we don’t use azithromycin for chlamydia anymore. Additionally, more evidence was arising regarding azithro resistance in both gonorrhea other organisms. Ultimately, the risk of decreasing susceptibility to azithromycin in *N. gonorrhoeae* and other organisms is now thought to outweigh any theoretical benefit.  Of note, we still treat for chlamydia if it has not been excluded. We will get into that on the next slide  The facilitator should also discuss expedited partner therapy. EPT is when the sex partners of people diagnosed with gonorrhea or chlamydia infection are treated without first examining or testing the partner. This is based on state specific laws and while the CDC recommend EPT for both gonorrhea and chlamydia, there are some states that don’t allow both.  Of note, this is ONLY for heterosexual couples and NOT MSM (men who have sex with men) due to concerns about the high prevalence of other STI creating missed opportunities for HIV and syphilis diagnosis and treatment, ultimately increasing transmission. Most recently the CDC changed their recommendations that for same sex-sex partners it is shared decision making regarding EPT in MSM.  EPT for gonorrhea is a single oral dose of Cefexime 800 mg if chlamydia was excluded.  -Increased variability in Ceftriaxone levels in pharynx are what guide increased dose |
| 36* | [Ask question]  Facilitator should ask the question to determine if learners know the recent guidelines on management of chlamydia infections.  A lot of medical students and residents may be taught about treatment of gonorrhea and chlamydia using previous guidelines. However, in 2021 there were new guidelines for chlamydia.  A meta-analysis and a Cochrane systematic review determined that treatment failure among men was higher for azithromycin than for doxycycline and that doxy was more efficacious for rectal chlamydia in men and women.  EPT is the same prescription.  Doxycycline is more efficacious for rectal *C. trachomatis* infection for men and women than azithromycin (among MSM – men who have sex with men - cure was 100% with doxycycline and 74% with azithromycin)  Although unclear significance of oropharyngeal *C. trachomatis* infection, can be transmitted to genitals; therefore, if identified from oropharyngeal specimen, it should be treated. |
| 37 | [Read the learning objective] |
| 38* | [Ask question]  This is an open question to prompt the learners to talk about prep as another preventative strategy in patient’s word higher risk of STIs. In case they need further clarification, the facilitator can specify whether there’s anything else in terms of prevention of sexual risk |
| 39 | This is just an introductory slide to PrEP, with no additional information needed |
| 40 | The facilitator can just read the indications for PrEP and ask if there’s any questions but no additional information to share here. |
| 41 | These are the initial labs that should be collected for all patients who are interested in starting pre-exposure prophylaxis for HIV.  The following are pieces of information that the facilitator should share with the learners.   - The HIV test can have false negative results during the first week when the viral load is still too low. If there were any sexual encounters within this timeframe, it might be better to delay start of PrEP and instead repeat testing within the next week. An additional strategy could be to get a viral load and adapt share decision-making. - PrEP is contraindicated in patients who have active, acute hepatitis B virus infection because antivirals used for PrEP make cars increased resistant patterns for HBV, and it stopped can cause acute reactivation of hepatitis B infection - If patient had exposure to someone with HIV within 72 hours, they have indication to start post exposure prophylaxis (PEP). But if past this threshold, and they have acute viral symptoms, it is advisable to get a viral load and delay the start of. PrEP |
| 42 | The table compares the two medications that are available orally for pre-exposure prophylaxis. The facilitator can just go through the table and compare each one of these. Then a pop-up will appear showing common side effects share between both medications.  TRUVADA: emtricitabine (FTC)/tenofovir disoproxil fumarate (TDF) for all patients  DESCOVY: emtricitabine (FTC)/tenofovir alafenamide (TAF) for MSM and transgender women |
| 43 | This table also shows the doses for the medication’s. It’s not for the purpose of the learners to memorize the dose, but no the different regiment.  The facilitator should also mention that physician should only prescribe PrEP for 3 months so they come to clinic to get repeat testing. Reasoning is that if patient infected with HIV, having only 2 meds will cause resistance, so it might even be more beneficial to develop active infection than resistance. |
| 44 | This line is to show which are the monitoring parameters for patients and pre-exposure prophylaxis and the different time periods when they should be collected.  BMP (basic metabolic panel) Q3M: to monitor the reversible Cr bump with TDF/FTC and discuss switching to TAF/FTC etc if it occurs.  Facilitator should mention that CDC recommends Cr once at first 3 month FU and then every 6-12 months but many states and local experts do it Q3M |
| 45 | This light is to share that there is a subcutaneous option for pre-exposure prophylaxis is called Cabotegravir (also known as Apretude)  The indication for this medication is not for IVDU, only for sexual exposure for MSM and transgender women and cisgender women  Cabotegravir does not need BMP, only HIV/STI q2-4m.  Apretude is given first as two initiation injections administered one month apart, and then every two months thereafter  Could also do PO 2 months then every other month injection |
| 46 | [Read the learning objective] |
| 47 | Concluding statements, open for facilitator to choose |

Second session:

| **Slide Number** | **Facilitator Script** |
| --- | --- |
| 1 | Introduction, open for facilitator to guide, can be very brief. |
| 2 | [Read the case]  The facilitator is prompted to read this case, which is a continuation from the case in the first session. |
| 3 | The facilitator can click through the animations while reading why the patient is here in clinic for this visit. |
| 4 | This slide is an introduction to the definition of gender-affirming treatment. Gender-affirming treatment is the process of recognizing or affirming, transgender and gender diverse (TGD) people in their gender identity. There is no one-size-fits-all approach, meaning that there are multiple ways of approaching this depending on each individual patient. Institutions often take a multi-disciplinary approach to gender-affirming care given the diversity of care goals and services needed, which can include mental health, specialist, endocrinologist, surgeons, and communication specialist, TGD peer navigators, among others. It is key for the facilitator to mention that gender, affirming treatment is safe and effective, and that it is not experimental given the decades of clinical experience and research regarding this topic. It has a solid evidence base with areas of emerging inquiry – similar evidence as many other things we do in medicine. |
| 5 | The facilitator should acknowledge that coming out as transgender, gender, diverse, and the process of transitioning is time-consuming and emotionally challenging in different ways to different patients. Gender-affirmation means something different to everyone. There is no one, single pathway towards gender-affirmation, as this may have different meanings to each individual patient. Therefore, gender-affirmation should be an individualized approach.  There are different categories of gender-affirmation. The facilitator can click through the different animations which will bring up the different categories and includes different example within each category. For example, social affirmation involves changes in name, pronouns, haircut, clothing, and other external appearances. Legal affirmation implies changing official ID documents. Behavioral affirmation requires mental health support through counseling and peer support. Medical affirmation involves gender, affirming hormone therapy or hair removal. And surgical affirmation includes surgeries, aimed to change facial characteristics, chest, genitals, and others. |
| 6* | [Ask question]  Ask audience about examples on how to bring this conversation during a patient encounter. Also remind them about the different examples that were discussed during the first session when discussing how to take a sexual history and how open-ended questions are the most effective ones to establish a trusting relationship in an inclusive space. |
| 7 | Once the audience has given their initial impressions to the question in the previous light, present this framework as a good two-step process to objectively identify how the patient currently identifies.   1. The first question to ask a patient is to ask them what there is sex assigned at birth was. Patients are either assigned male at birth (AMAB) or assigned female at birth (AFAB. There are smaller minority of individuals may be designated as intersex or having a difference of sexual development. 2. The second question is asking patients how they describe themselves: Male/man, female/woman, transgender, other   Plug in the first session and how we talked about asking for pronouns and how patients want to be called. Ask about name, how they want to be addressed and if there are any preferred pronouns. Can also ask patient. “How do you describe your gender identity”? |
| 8 | [Read case]  Hear the facilitator continues the case, saying that Callie identifies as a transgender woman and no identifies as a transgender man. The facilitator should mention that not all patients like identifying with AMAB or AFAB, but we need to keep accurate medical information in the chart (maybe can be placed in identifier and not in one-liner). They only need to put their sex assigned at birth and their gender identity separately |
| 9 | Below is the information that should be mentioned for the first animation in the slide:   - The proportion of trans people is 0.6%, however it is estimated that the proportion of TGD people ranges from 0.5% to 4.5% (difficulty in estimating true proportions so we typically describe as a range)   - **Definitions** differ internationally, so proportions may vary between countries   - Proportions are variable across time **as people become more comfortable, and visibility increases**   - Only capture the those who have sought care   With each individual animation that follows, facilitator should mention the following:   1. There are multiple examples of human rights violation among the trans population, including: education, health, work, housing, privacy, security, family, fair trial, freedom from torture. It varies across the world and even within the US it varies between different states 2. 15% unemployment in trans patients (vs 5% of cisgender patients) 🡪 trans and gender diverse patients have higher likelihood of not having health insurance which widens health disparities compared to cis patients 3. 30% homelessness in TGD people: secondary to being kicked out of home, harassed by shelter/LTAC staff, turned away from shelters, or sexual assault 4. 20% of TGD people work in underground economy (sex work, drug sales, others) -> increased risk HIV and HCV due to harmful behaviors 5. Stigma and prejudice also contributes to poor access to healthcare, lack of care in correctional facilities and LTAC/SNF 6. 29% of TGD people live in poverty (vs 12% of cisgender counterparts) |
| 10 | Hear the facilitator shares that TGD patients have increased rates of mental illness, physical illness, and lack of access to high-quality care. They can mention that 40% of trans people have attempted suicide (x9 higher than gen population).  Facilitator can also add to the third point open, bearing lack of access to high-quality care):   - Lack of access to care and provider knowledge leads many TGD to take non-prescribed hormones (potentially supratherapeutic and unsafe) and rely on info from community members (not always accurate) - Trans patients have to frequently teach their own doctor how to receive care (24%), are asked invasive questions unrelated to the visit (15%), or are outright refused care (8%). - ¼ avoid seeking care because of fear of mistreatment. 1/3 avoid due to cost - 3x more likely to travel >50 miles for trans care - Some insurances don’t cover services |
| 11* | [Ask question]  This is a question that aims for residents to understand what patients mean by transition, what their goals are, why they are requesting to transition, etc. It is very important that audience understands that each patient has individual and unique goals and we must ask open ended questions to get to that answer. The facilitator can share that similar to any other patient with any other health concern, physician should try and understand what the patient’s goals are.  Essentially: elicit information to understand where the patient is coming from and what gender-affirmation means to them |
| 12 | [Read case]  The facilitator moves on with the case and mentions that Callie and Noah want to start hormones |
| 13 | This slide serves to mention the definition of gender-affirming hormone therapy (GAHT). This definition was based on acknowledgments and guidelines from the AMA, endocrine society, and W path. The goal of GAHT is to induced physical changes to the patient’s gender identity. GAHT is medically necessary and GAHT is standard of care. Physicians must understand that these medications are not elective, cosmetic or for convenience. They are used for the above stated reasons. All medications used for GAHT are medications that we already know and are comfortable using as physicians, especially as internists, so they have a well-known safety profile.  MH: mental health; QoL: Quality of Life |
| 14 | Facilitator should mention that PCPs should care for TGD patients or make appropriate referrals if they do not feel comfortable. But just like any other medical condition, we should know the basics and try to address first before referrals (unless too complex), which includes prescribing medication’s and assessing mental health and providing preventative care.  If it’s a complex case, refer to a specialist in trans care!  Preventive care includes cancer screening. |
| 15* | [Ask question]  Facilitator should post this as an open question to the big group. This question is intended. to assess baseline knowledge and see where the audience is at. It might help gauge how much information you should provide in the rest of the curriculum or if you can include more active participation. |
| 16 | Facilitator should first mention that this curriculum is only for patients were 18 years or older, or adults. This curriculum is not intended for minors or pediatric patients.  Facilitator should then read out loud each individual step to prescribe GAHT and that this is what’s going to be covered in most of the session. The rest of the slides are just going to be adding detail to what all of this means so there shouldn’t be more detail regarding these steps at this point |
| 17 | Facilitator can mention that any PCP can assess for gender dysphoria/incongruence. A mental health provider is NOT needed and therefore all participants should be able to assess this like the rest of their patients with other medical conditions. The same reason that a PCP can make a diagnosis of depression, anxiety, or PTSD, they can make a diagnosis of gender dysphoria/incongruence. |
| 18 | Lower the points of what the facilitator should say in each one of the individual bullet points that are advanced with each animation  1) Describe that Gender Dysphoria is a diagnosis in the DSM-V, and it has to be present for at least 6 months plus have 2 of:   - - Incongruence with sex characteristics   - Desire to get rid of sex characteristics   - Desire for the sex characteristics of the other gender   - Desire to be of another gender   - Desire to be treated as another gender   - Conviction to have feelings and reactions of another gender   2) Changes have to be marked in sustained. An abrupt or sudden change is insufficient. This should raise concerns for acute stress, or paranoia, or delusions, or other mental illnesses. This is a good situation where patient could be referred to mental health specialist. GAHT has irreversible changes and we have to ensure not to cause more harm than good in these unique situations (although they tend to be rare cases)  3) Not everyone has dysphoria related to their gender. Now there is an ICD-11 diagnosis called “gender incongruence” which is used when patients don’t have dysphoria   - Should clarify that PCP’s are NOT diagnosing gender identity (e.g. this person is trans). Instead diagnosing dysphoria or incongruence pertaining to gender. PCP are evaluating the **stress** **as a result of the incongruence**.   4) TGD people are not inherently disordered. It is **due to distress** from gender dysphoria. It is not a mental disorder or a mental health pathology  5) Gender dysphoria varies from person to person, and it may look different for one person than for the other  6) Some states required diagnosis in the medical chart to allow for coverage of medication and treatment |
| 19 | The provider’s role: educate TGD patients about the effects of GAHT and side effects, just like any other treatment or intervention where we get consent by informing the patient of benefits and risks. Understand that treatment should be individualized  **Mention the “Informed Consent” pathway**  Ensure patient has Capacity: able to understand risks, benefits, alternatives, unknowns, limitations, risks of no treatment  Consent should be given for each individual intervention or medication, just like for any medical condition.  Any PCP or physician can assess for capacity unless symptoms affect capacity to consent (for example if distress is too high that patient cannot understand risks of treatment)  If too complex medically or mentally, can always refer to specialists (psychiatry/psychology)  It’s a shared decision-making model, just as everything else in medicine! |
| 20 | Should explain that GAHT can be divided into two main groups, feminizing hormone therapy and masculinizing hormone therapy. This is a binary approach to simplify education, but always align with patient’s goals. |
| 21 | **Breakout session #1**  This is the first breakout session of this second part of the curriculum. The facilitator should divide all learners into small groups of 4–5 residents and instruct them to discuss what they expect to be the major changes in patience when they take estrogen and testosterone. Facilitator should instruct learners not to use any additional resources but try and think what these hormones may do and how they would alter sex characteristics by using their intuition. Intercalate which groups will discuss estrogen based therapy and which will discuss testosterone therapy. Assigning one type of hormone therapy to each group, and divide equally among all groups. After 10 minutes of group discussion, bring all small groups into a big group to discuss the expected changes. The facilitator should use the tables in the next few slides as a reference. There is no need to discuss onset and max effective change as this may be too complex for their level of training. Instead focus, and just mentioning the changes expected in the body and that should be sufficient. This should take an additional 5–10 minutes |
| 22 | When’s the facilitator and the learners have discussed the possible changes in patient’s bodies, the facilitator can show this table that summarizes the answer of the expected effects from estrogen as well as the maximum effect in terms of timeline. Below just a few additions that can be mentioned.   - Breast development: fewer than 20% reach Tanner 4-5 after 2 years. Usually it's Tanner 2-3. Important to set expectations - 22% meet hypoactive sexual disorder, although unclear if from estrogen, androgen blockers, or surgery or combination - Change in sweat and odor patterns |
| 23 | Breast growth (bolded) is an irreversible change. impact on sperm production/fertility can be irreversible. |
| 24 | When’s the facilitator and the learners have discussed the possible changes in patient’s bodies, the facilitator can show this table that summarizes the answer of the expected effects from testosterone. as well as the maximum effect in terms of timeline. Below just a few additions that can be mentioned.  - Cessation of menses 2/2 endometrial atrophy and cycle arrest  - Change in sweat and odor patterns  - Some people want to stop GAHT when they have hair loss. Treatment of hair loss is the same as in non-trans patients.  - Increase in libido also be seen. |
| 25 | Bolded are irreversible which is why it’s so important to set expectations and ensure that patients have thought these changes through and agree this is within their goals if it happens. |
| 26 | Facilitator should let the learners know that after discussing the changes in the body, they should set expectations with their patients. Below explanations for each of the animations of the slide   - The first thing is mentioning that the timeline is different for every single patient and that it’s difficult to predict when certain changes might happen. - However, it is known that the maximal effect of GAHT is anywhere between 2 to 5 years. - The older the patient is at answer of hormone therapy, the lower the benefit, and higher the risk of side effects. - Some patients may request alternative regimens 🡪 this is ok as long as this regimen is safe and falls within the standards. - However, it is important to note that NO regimen predicts specific changes of sex characteristics and there is no evidence or studies that have documented this. There are many online communities and forums sharing misinformation and it is important to set expectations with current evidence. - The best approach is to start with low-dose and titrate up until reaching target levels |
| 27 | All trans people should be counseled on effects of GAHT on fertility and reproduction before starting. This is part of the informed consent pathway and should always be discussed before initiation of any hormone or any therapy as in some cases it can be irreversible. |
| 28 | Facilitator should mention that GAHT has different effects on fertility:  Estrogen 🡪 can cause testicular atrophy and reduced sperm count. Semen quality is also negatively affected given reduced masturbation and tucking. For these reasons, it can be irreversible in some patients  Testosterone 🡪 causes cessation of menses and ovulation however may not cause any permanent changes. Therefore, in most cases, it’s reversible  After stopping GAHT, fertility may return impatience 3 to 6 months after cessation.  Infertility is not complete despite the use of GAHT, so TGD people need contraception to prevent pregnancy  If pregnancy is desired transgender men, testosterone should be stopped since it is teratogenic and should be resumed after lactation. |
| 29 | Many TGD people want parenthood 🡪 consultation with fertility specialist should be offered prior to initiation of GAHT  Some TGD patients can’t make future-based decisions because of dysphoria so difficult to make decisions on parenthood. This is where informed consent and evaluation of gender dysphoria is important. Refer to mental health specialist if any concerns for capacity or decision-making capabilities.  There are different options, such as sperm banks, prior preservation, fostering, and adoption  ~50% appreciate having the conversation and many end up choosing fertility preservation options prior to initiation of GAHT so should not be seen as “gatekeeping”  Insurances are starting to cover fertility options |
| 30 | Facilitator should mention that physician should rule out alternative causes of gender dysphoria, as well as ensuring that medical and mental health conditions are reasonably well controlled to prevent harm. This is also part of the informed decision pathway.   - Medical conditions such as CV risk factors - Assess whether interventions may result in unfavorable psychological or social outcomes or exacerbate underlying medical conditions. For example, some patients after they transition socially can get harassed, and this can exacerbate their underlying psychological conditions. |
| 31 | The facilitator can use the below points to describe each one of the bullet points in this slide  1) Some states/facilities still require mental health appointment and are gatekeeping. However, per guidelines, the services are not required to start GAHT, although they may be beneficial   - - Mental health is beneficial for support around gender dysphoria: Higher rates of concurrent stress, substance use, mental illness (2/2 social stigma), early childhood messages about gender norms, Family stress, feelings of needing to hide parts of oneself, Stressors around community, finances, decisions re: transition; frustrations around paperwork, legal documentation, etc.   2) Sometimes mental health, substance use disorder (SUD) and gender incongruence co-exist so should be treated simultaneously.  3) Withholding treatment can cause iatrogenic harm because GAHT reduces mental health symptoms in TGD people, so it may worsen mental health symptoms which may increase risk of suicide.  4) Some patient may have onset or exacerbation of mental health illnesses but stopping GAHT may cause more harm or can worsen mental health symptoms so recommendation is to continue GAHT and concomitantly assess and treat other mental health illnesses. |
| 32 | There really are three major contraindications: history of hormone sensitive cancer, which requires consultation with oncology, a patient in an acute psychotic episode, and a pregnant patient  GAHT should be used with caution in patients who are too depressed to engage in aftercare or follow up (neglect wound care, symptoms, postop care, follow up appointments).  Also assess risk of losing social support and financial stability after transitioning. Ensure patient has explored this and if there are contingency plans in case this is a possibility.  Homelessness is NOT a contraindication for hormone therapy or surgery. Should be case by case |
| 33* | The facilitator can continue reading through the case in this slide.  At the end, there is a question (*) to prompt to get audience to mention they should make a referral to a fertility specialist if the patients agree AND BASELINE LABS. Facilitator should really hold down to the point that a fertility specialist should be offered and also that the learner should start thinking about the next steps, which are baseline labs. Although this is not been discussed so far, it is preparing for the next learning objective. |
| 34 | The facilitator can list the next three final steps on how to prescribe GAHT without really adding any more detail and let the learners know that this is what’s going to be discussed in the rest of the session. |
| 35* | [Read step]  [Ask question]  Ask audience what type of labs they think they should get when prescribing GAHT. It’s not expected that the learners will know, most to see what deductions they can make or if they have any baseline knowledge. Also, a great space to assess whether there is any prior knowledge that was incorrect. |
| 36* | Facilitator should then show this slide of baseline labs. This is a slide meant for interaction with the audience.   - Ask (*) the learners why they think H/H (hemolgobin/hematocrit) and BMP are needed. H/H is needed to assess for polycythemia (baseline and to monitor with time) and BMP given spironolactone (changes in potassium) - Baseline and follow up lab practices may vary across institutions - Testosterone levels are needed as some individuals may conditions that may affect dosing to achieve therapeutic levels - **H/H**: reference ranges are based on the hormone being prescribe   When there are any baseline abnormalities and concern for underlying medical conditions, those should be treated and these are patients who may benefit from referrals to endocrinologist, especially if any concern about an endocrine pathology. |
| 37* | [Read case]  The facilitator should read through the case in this slide.  Then, with animation, there is a question (*) at the end asking learners if they want to place a referral to behavioral health. Once they have given their thoughts, remind audience that placing a referral to behavioral/mental health is NOT required as long as their mental health symptoms do not affect their capacity to understand the risk-benefits of treatment.  But that it **should be offered** as support to **all patients**. Should not be used as gatekeeping. |
| 38 | The facilitator can just mention that this is the step they have all been waiting for, and fortunately it is one of the easiest parts of the process given very safe and common medications. |
| 39* | This slide shows the same binary approach that was introduced earlier in the session. Then the facilitator can click through the animation and ask (*) if the audience has a baseline knowledge of which medications are used. Because it is so intuitivie, it can reinforce learning by then realizing they are correct if the mention estrogen and testosterone. |
| 40* | The presenter should specify that feminizing hormone therapy involves the use of estrogen and androgen blockers and that masculinizing hormone therapy involves the use of testosterone alone. Then the presenter can click through the animation and ask (*) the question in the slide regarding specific medications. Learners should attempt at answering and then when advancing the animation, the answer will show up.  - Presenter can clarify that androgen blockers reduce testosterone AND reduce the amount of estrogen needed |
| 41 | In this slide the facilitator should highlight that the encircled hormones (testosterone and estradiol) are chemically equivalent to the estrogen/testosterone secreted from the human ovary/testicle.  Estradiol given in GAHT is specifically 17-beta estradiol in case anyone asks  Testosterone given for GAHT is different than the one synthetic one used by bodybuilders. This is the chemical equivalent to what humans produce  For feminizing hormone therapy, mention that estradiol and spironolactone are usually started simultaneously  Some patients prefer to start spironolactone first to try androgen blockade first, that’s ok, or vice versa. Guidelines don’t find any contraindication for this variability. |
| 42* | As the facilitator advances to this slide, the facilitator can mention that the estradiol used is 17-beta estradiol. And a question pops up to ask (*) the audience why not use ethinyl estradiol and instead, since it’s already used commonly for contraception. This question is to start gauging what they know about types of estrogen, which is likely a knowledge gap in many IM residents. Once they have answered or guessed, can move on to next slide |
| 43 | Facilitator should mention that there is a higher risk of VTE in patients taking ethynyl estradiol or conjugated OCPs, and then move on to next slide |
| 44 | In this slide audience learns that estradiol only increases risk of VTE by small amount compared to other estrogen. With estradiol, absolute increase from 1/1,000-10,000 to ~2.5-4/1,000-10,000. Therefore the difference from 1 to 4 people in absolute terms is almost negligible so it should not be considered a contraindication but mentioned as a possible side effect of treatment.  There is an often-misquoted study that mentions that there is a 25x risk of VTE in trans women, but this study was done w/ ethynyl estradiol or conjugated estrogen, NOT estradiol, and smoking history was not accounted for |
| 45 | Let audience know that no need to memorize doses and slide mostly for reference and to know different formulation options. That they can use handout or other online resources when prescribing to know what dose is needed.  ***Initial-low is for personal goals (some genderqueer and nonbinary folks who prefer taking it slowly to see how their bodies change)**  ****Maximal dose does not mean maximum effect, doses depend on patient response and hormone levels in blood.**  PO titrate by 2mg Q3mo, Transdermal titrate by 0.05-0.1mg q3mo, spironolactone titrate by 50 mg q3mo  There are gel and sprays, also not as common  Here the facilitator should mention that there is IM/SQ estrogen, however the availability varies by state and region and also evidence is evolving so not too much detail in this curriculum. Also providers may not feel as comfortable if they have not prescribed before so ok to refer to a specialist although not required. |
| 46 | Same as prior slide, let audience know that no need to memorize doses and slide mostly for reference and to know different formulation options. That they can use handout or other online resources when prescribing to know what dose is needed. Here the facilitator should primarily note that primarily an IM/SQ medication that is given weekly or every 2 weeks. Also mention that providers should not forget to prescribe the syringes!  ***Initial-low is for personal goals (some genderqueer and nonbinary folks who prefer taking it slowly to see how their bodies change)**  ****Maximal dose does not mean maximum effect, doses depend on patient response and hormone levels in blood.**   - SQ preferred: less pain, scarring, fibrosis - Q2Wks may cause higher concentrations so may have increased side effects - Gel: should remain dry for at least 2 hours. avoid contact with others. Wash if close skin-skin contact is expected (for example if going to take care of children/babies) |
| 47 | [Read case] |
| 48 | **Breakout Session #2**  This is the second breakout session. The goal of this activity is to clarify the main side effects of GAHT. Most residents should have some baseline knowledge because these are medications they use for other indications.  The facilitator should mention that in this activity each group should fill out the entire table of side effects (second column only, comments will be done by facilitator afterwards). Once instructions are given, divide residents into three small groups and assign one medication class to each group. If a big group, can create 6 smaller groups and give one GAHT medication to 2 groups. Give them 5 to fill out their assigned task. Afterwards, regroup everyone and first ask the group that discussed estrogen to share the side effects. |
| 49 | The facilitator should show this slide after the residents have mentioned the side effects for estrogen.  A few comments that the facilitator can add:   - Migraine: to prevent, can start at lower dose or use transdermal estrogen - Pituitary **prolactinoma** - Few case reports reporting association with estrogen therapy, but no increased risk   - NO screening needed   - It is physiologic to have non-bloody minimal galactorrhea early in their therapy course and would not warrant further evaluation   - Prolactin levels should **only be checked**if symptoms of prolactinoma |
| 50 | Here facilitator should add some comments about risk of VTE. Those who smoke and older age are at higher risk. Also add that estrogen should not be stopped prior to surgery as no study has shown that trans women are at increased perioperative risk of VTE compared to general population. Also routine screening for prothrombotic state is NOT recommended   - Facilitator can click through first animation and mention that if a patient is at increased risk of VTE due to other risk factors (e.g. smoking), discuss changing modifiable risk factors and can also change formulation to transdermal estrogen that has minimal risk of VTE - Facilitator can click through animation to bring pop-up box and mention that patients >45 years of age and prior history of VTE should probably be on transdermal estradiol given increased baseline risk. |
| 51* | This is also an interactive slide to determine if residents have additional tools for patients who have a VTE. Pose it as an open-ended question (*). Once the audience has given their thoughts, the facilitator can start clicking through the different animations to show how to approach patient with a VTE and who are taking estradiol. At the end of the slide and at the end of animations, mentioned that having a VTE is not an end-all for estrogen therapy. Focus should be in prevention, risk control (smoking cessation), continuous treatment of VTE, and monitoring of persistent or progression of VTE. Should be shared-decision making |
| 52 | The facilitator then goes back to this table with the side effects and asks the groups who discussed spironolactone to comment on water the common side effects. After the residence of shared, the facilitator can click through the animation. |
| 53 | The facilitator starts the slide by asking the residence who were assigned to testosterone to share what the common side effects of this medication are. Once they have shared, the facilitator can click through the animation to reveal the content of the table. Facilitator can also add:   - For erythrocytosis/polycythemia, evaluate for other causes first before attributing to meds. Changing to more frequent and lower dosed injections (Qweek) or transdermal preparation may decrease this risk - In patients when there are inadequate results or side effects from testosterone when they’re at their testosterone range goal, patient may need additional labs, which would be beneficial to assess with an endocrinologist. |
| 54* | The facilitator reads through the slide and mentioning that the patient encounter has finished. Then a question pops up (*), asking when the patient should be back for follow up. Here is also a good opportunity for participation from the audience to try and mention how frequent to follow up labs and monitoring should be. But more importantly, ask them to give reasons as to why they think that specific amount of time to assess their knowledge of these labs in other situations. |
| 55 | Facilitator can read through the slide title |
| 56 | The facilitator starts to slide by saying that the target is to obtain sex steroid serum levels that match the patient’s gender identity. Then they can click through the first animation to show the levels of estradiol and total testosterone for patients who are trans feminine or using feminizing hormone therapy. The facilitator should make the following points:   - For SL estradiol, check levels 2-4 hours after dose - Titrate both estradiol and spironolactone until estrogen at desired levels - If persistently elevated testosterone with max androgen blockage, test for testicular cancer and if ruled out 🡪 endocrinology referral   After this, the facilitator can click through the next animation to show the levels of estradiol and total testosterone for patients who are trans masculine or using masculine hormone therapy. The facilitator should make the following points:   - Cessation of menses is likely a good goal, only check estradiol if menses persist; goal should be <50 pg/dL - In injectables, always get labs midweek between injections   Then facilitator can click through the final animation and mention that higher serum levels do not result in greater degree of feminization or masculinization. Also point out that not using levels may increase risk of undertreating, so always use levels |
| 57 | In this light, the facilitator shows what the typical follow up schedule is. For the first year, lab should be collected every three months after initiation of GAHT until target levels are reached. Testosterone is collected for all patients. For patients and feminizing hormone therapy, they should obtain estradiol and BMP. For patients on masculine hormone therapy, they should get an H/H. Doses of hormones can be titrated every three months until target levels are reached.   - In trans men using injectable testosterone, (lab) should be drawn midway between injections because if drawn too early the lab levels can be mistakenly high, and if taken too late, they can be mistakenly low.   The facilitator then click through the animation and says that once the target levels are reached, there is no need for frequent visits other than yearly visits. The same labs are collected on these yearly visits as well as any other labs as needed for preventative care, for example: onset of DM, thyroid dx, weight changes, virilization, new symptoms likely caused by hormones. |
| 58 | There are alternative regimens that some patients may ask to their providers. There is also a lot of misinformation in the Internet that a lot of trans patients have access to because it’s within their community. They may ask for regiments that are not standard of care. It is our job as physicians and primary care providers to adhere to the guidelines.   - Some patients may ask to start estrogen first or an antiandrogen blocker first. Based on the evidence, it is probably ok to start only 1 of 2 meds, as long as the patient is reminded that there is no evidence that a certain regimen can predict changes in body, despite what anecdotal evidence the patient may have. This is just to set realistic expectations.   If a patient requests any regiments that deviate from standard of care, it would be beneficial to refer to a specialist. Below are examples of situations that differ from standard of care and may need referral to specialists for more nuanced discussions:   - Different target levels have not shown any harm, although limited studies. - Progesterone: Anecdotal reports suggest progesterone may aid breast development and mood management in transgender women, but current evidence is **insufficient to confirm its benefits outweigh potential risks.** While no high-quality data support progestin therapy, some studies indicate potential harm with long-term use. If prescribed after a thorough risk-benefit discussion, patient response should be reassessed within a year.   - Micronized Progesterone: 100-200 mg QHS - **5-alpha reductase inhibitors** (finasteride and dutasteride) block conversion of testosterone to dihydrotestosterone (lesser effect)   - not enough data, only for those who can't tolerate spironolactone or seeking partial feminization   - Helpful for hair loss; not always covered by insurance |
| 59 | [Read through case] |
| 60 | One final comment before the end of the session, and can continue to the next slide |
| 61 | - The facilitator can read through these points. It is important to assess mental health as mentioned before. There should be an assessment on housing, food and security, financial and security and safety concerns and whether starting GAHT can negatively impact these aspects of their life. - It is also important to know that if a patient is admitted to a hospital or a psych unit for a mental health condition, GAHT should be continued, unless there is a specific contraindication because discontinuation can perpetuate and worsen symptoms of gender, dysphoria, and associated symptoms of depression, anxiety, or others - It should be pointed out that conversion therapy is not effective and can cause harm. There is no indication for its use in anything related to sexual orientation or gender identity. |
| 62 | This is a final slide to have the facilitator mentioned that social transition can improve mental health and reduce dysphoria. Some patients can start that social transition before starting medication’s, but it can’t always be done before GAHT, and that is done concomitantly. Most common reason to avoid social transition is fear of family/friend abandonment, economic loss and discrimination. Can increase suicidality among youth  Here are a few examples of social transitioning and practices:  **Packing:** use of soft fillings or penile prosthesis in underwear giving an outward appearance and reduce dysphoria  **Binding:** wearing tight bras, shirts, or binders to flatten chest. Can cause breast pain, breathing issues, skin irritation  **Tucking:** manually displacing the testes upward into the inguinal canal and positioning the penis and scrotal skin between legs and backwards. Tight underwear ("gaffe"), tape or even duct tape is used to keep in position. Sometimes kept even at bedtime. Can cause urinary reflux, prostatism, and UTI (urethra closer to anus), testicular pain |
| 63 | Conclusion, open for facilitator to close as they prefer. |

**Glossary of terms**

- **AAMC**: Association of American Medical Colleges
- **ACGME**: Accreditation Council for Graduate Medical Education
- **ACP:** American College of Physicians
- **CDC**: Centers for Disease Control and Prevention
- **Gender and Sexual Diverse (GSD)**: An umbrella term that encompasses individuals whose gender identity, gender expression, or sexual orientation
- **Gender Dysphoria**: Psychological distress resulting from a mismatch between an individual’s gender identity and their assigned sex at birth.
- **Gender Expression**: The external manifestation of gender, including clothing, hairstyle, and behavior.
- **Gender Identity**: An individual’s personal sense of their gender, which may or may not align with their sex assigned at birth.
- **Gender-Affirming Hormone Therapy (GAHT)**: Medical treatment that involves the administration of hormones to align a person’s physical traits with their gender identity.
- **GME**: graduate medical education
- **HIV**: Human Immunodeficiency Virus
- **LGBTQI+**: An acronym for Lesbian, Gay, Bisexual, Transgender, Queer, Intersex, and other diverse identities.
- **MSM**: men who have sex with men
- **PCP**: Primary care physician
- **PGY**: postgraduate year
- **PEP:** postexposure prophylaxis
- **PrEP**: Pre-exposure Prophylaxis, a preventive medication regimen used to reduce the risk of acquiring HIV.
- **Sexual Orientation**: A person's emotional, romantic, or sexual attraction to others.
- **STI: S**exually Transmitted Infections
- **WSW**: women who have sex with women
