# Supplementary material for: A Systematic and Practical Framework on Gender and Sexual Diverse (GSD) Health for Internal Medicine Residents
Source: MedEdPORTAL. 2025 Jun 17;21:11535. doi: 10.15766/mep_2374-8265.11535 (PMC12170925; doi:10.15766/mep_2374-8265.11535)
Supplement: Supplementary file 1 — GSD Health Handout.pptxGAHT Handout.pptxFacilitator Guide.docxGSD Health - Part 1.pptxGSD Health - Transgender Health.pptxGSD Health Survey.docxTGD Health Survey.docx [file mep_2374-8265.11535-s001.zip › D. GSD Health - Part 1.pptx]

## Slide 1
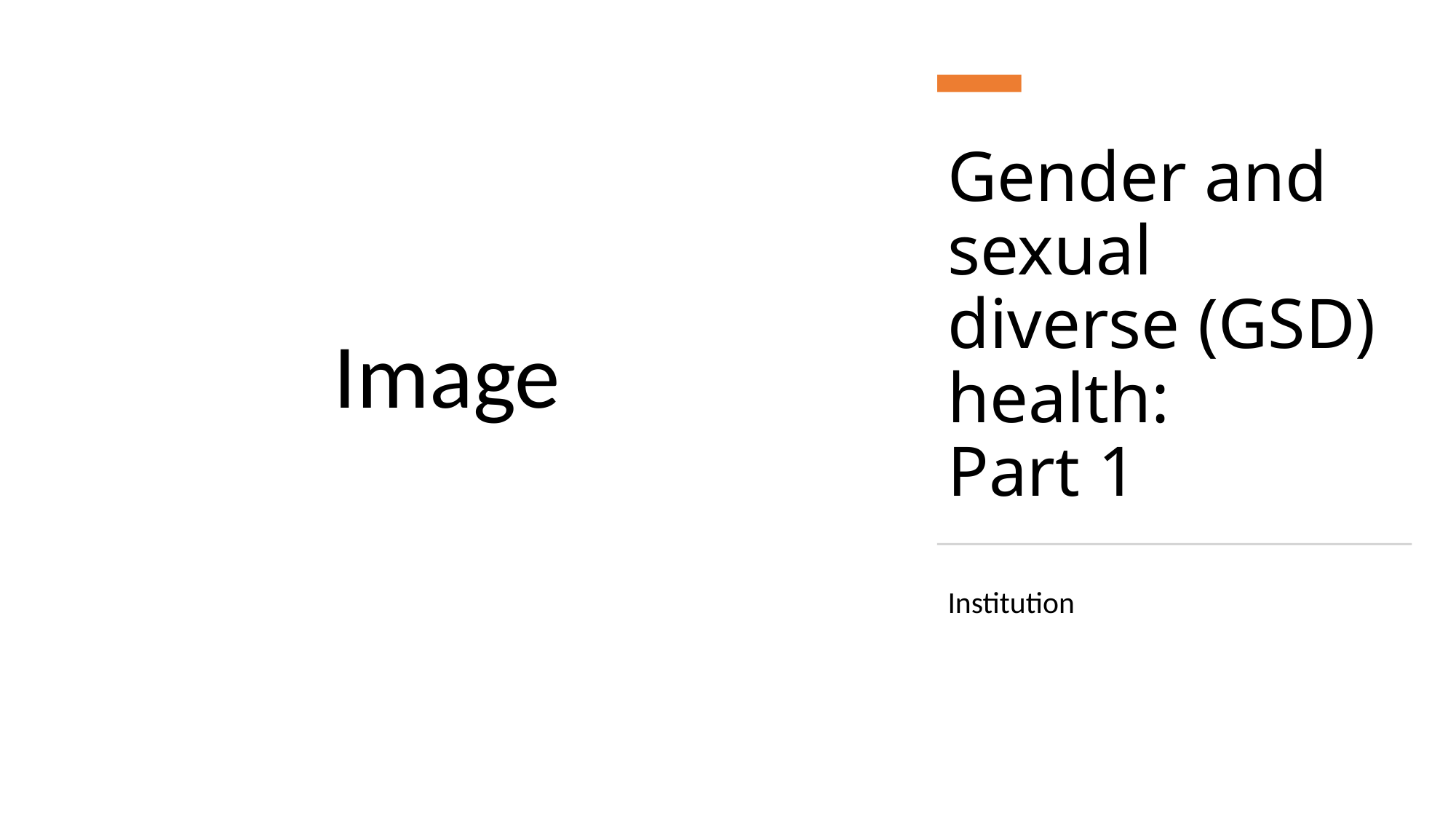

# Gender and sexual diverse (GSD) health: Part 1
Image
Institution

## Slide 2
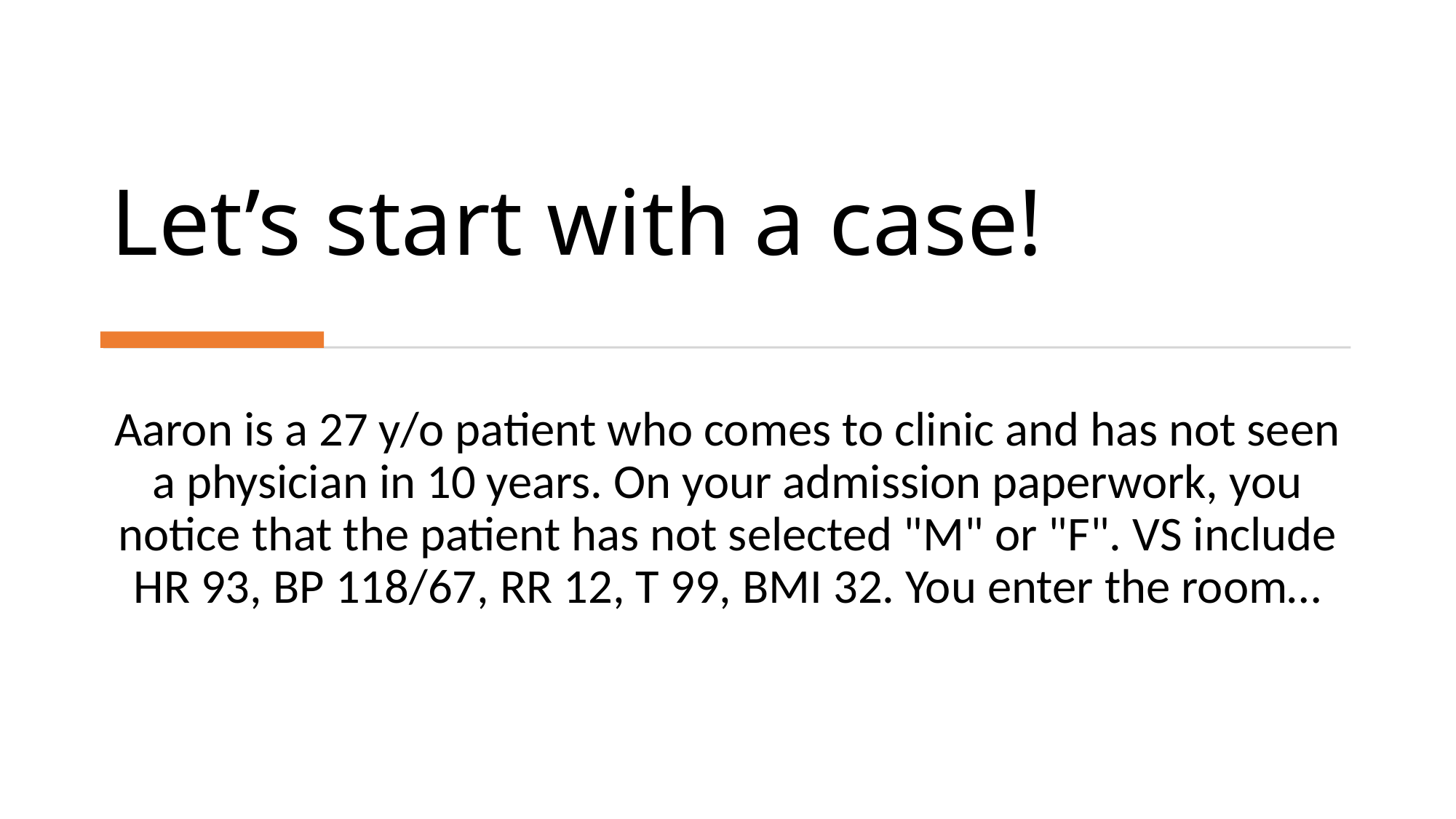

# Let’s start with a case!
Aaron is a 27 y/o patient who comes to clinic and has not seen a physician in 10 years. On your admission paperwork, you notice that the patient has not selected "M" or "F". VS include HR 93, BP 118/67, RR 12, T 99, BMI 32. You enter the room…

## Slide 3
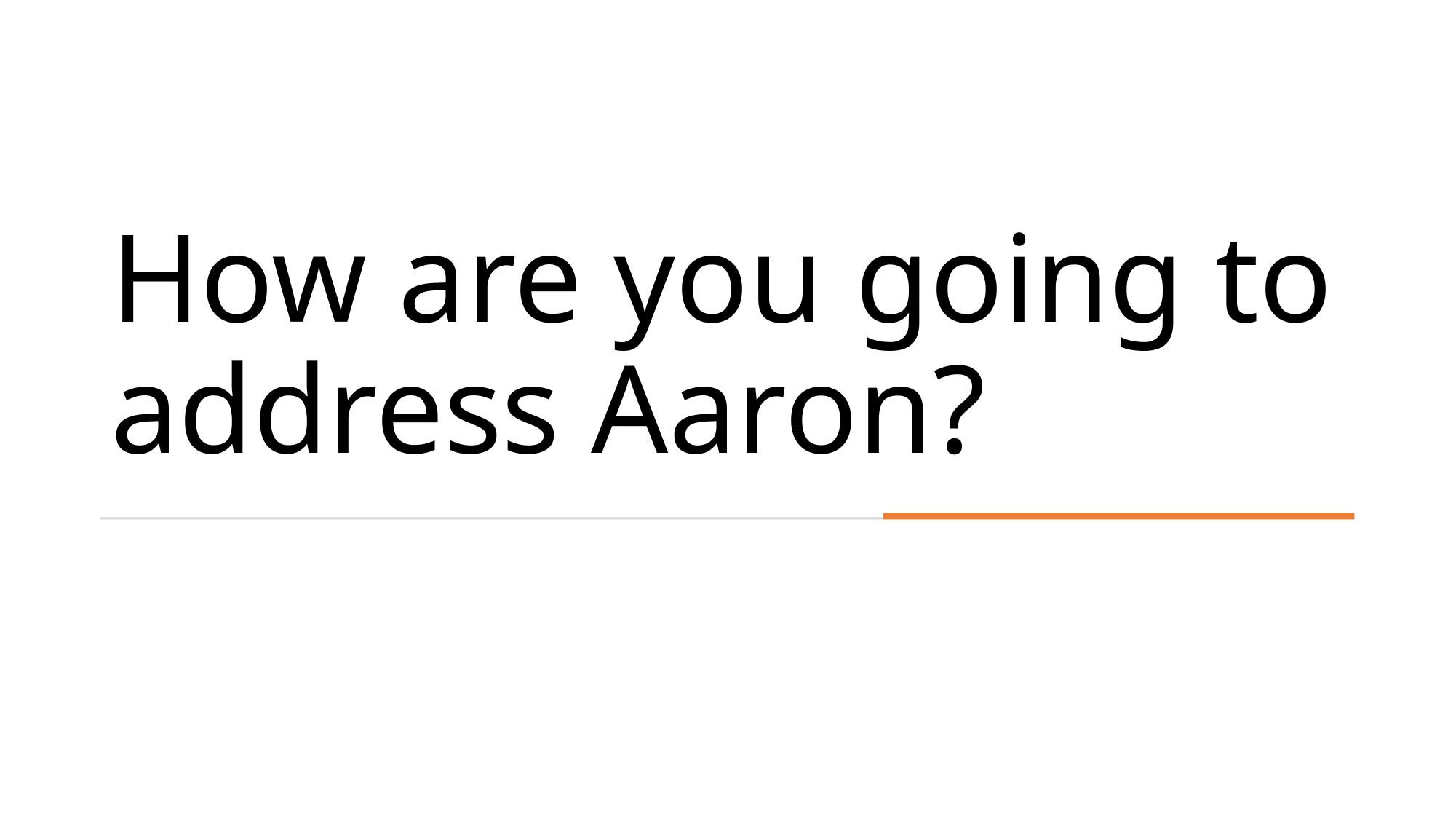

# How are you going to address Aaron?

## Slide 4
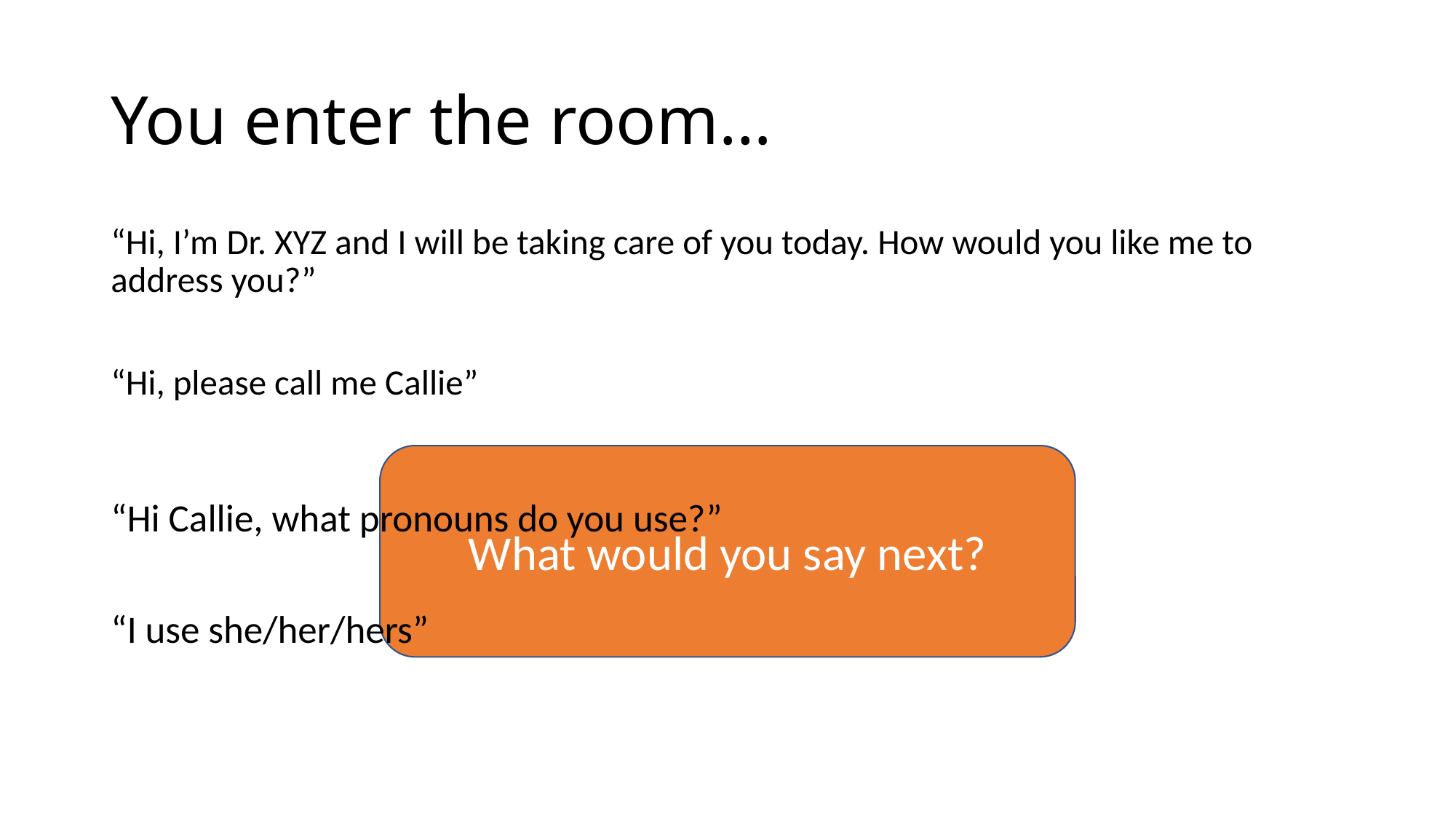

# You enter the room…
“Hi, I’m Dr. XYZ and I will be taking care of you today. How would you like me to address you?”
“Hi, please call me Callie”
What would you say next?
“Hi Callie, what pronouns do you use?”
“I use she/her/hers”

## Slide 5
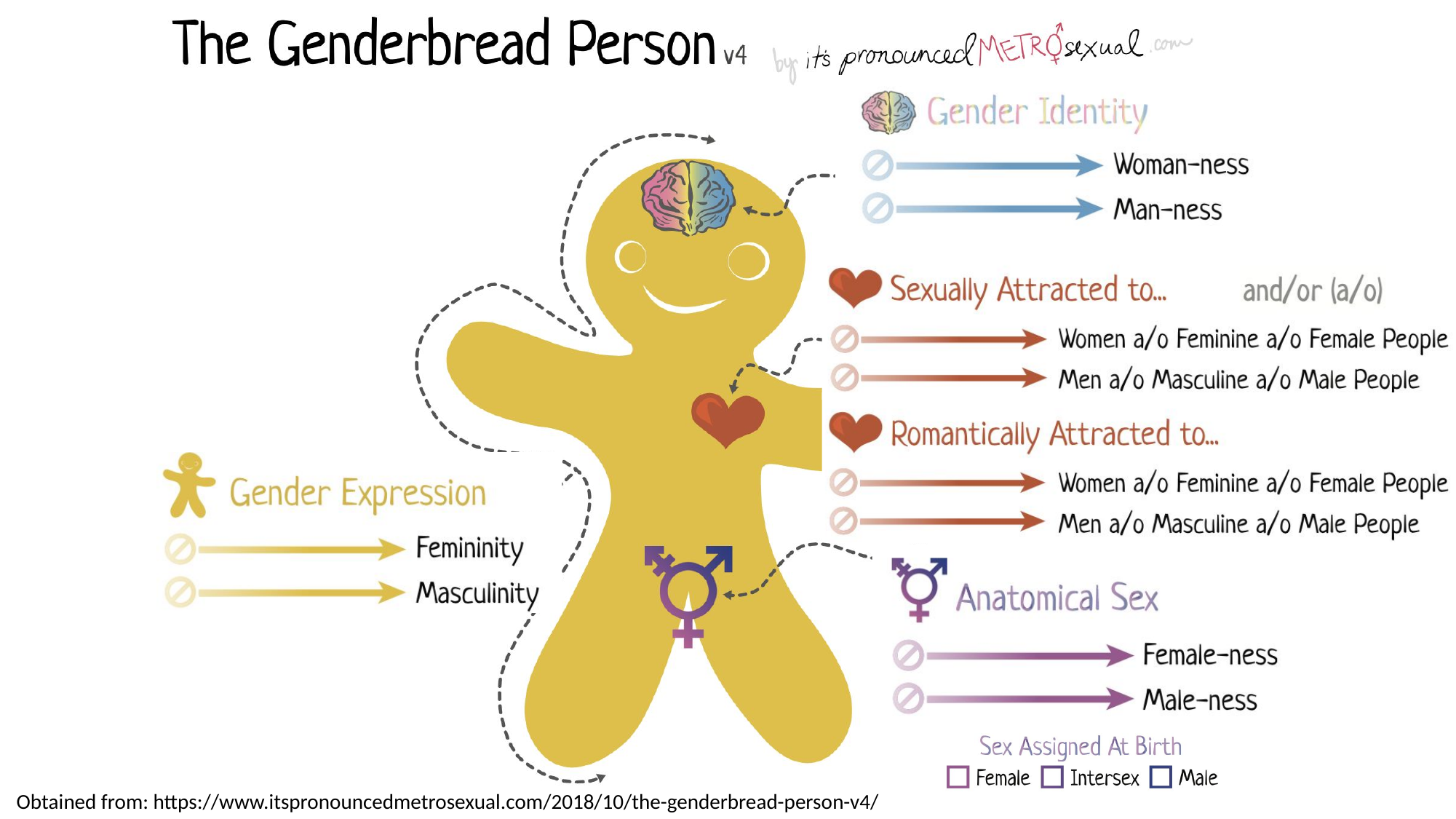

Obtained from: https://www.itspronouncedmetrosexual.com/2018/10/the-genderbread-person-v4/

## Slide 6
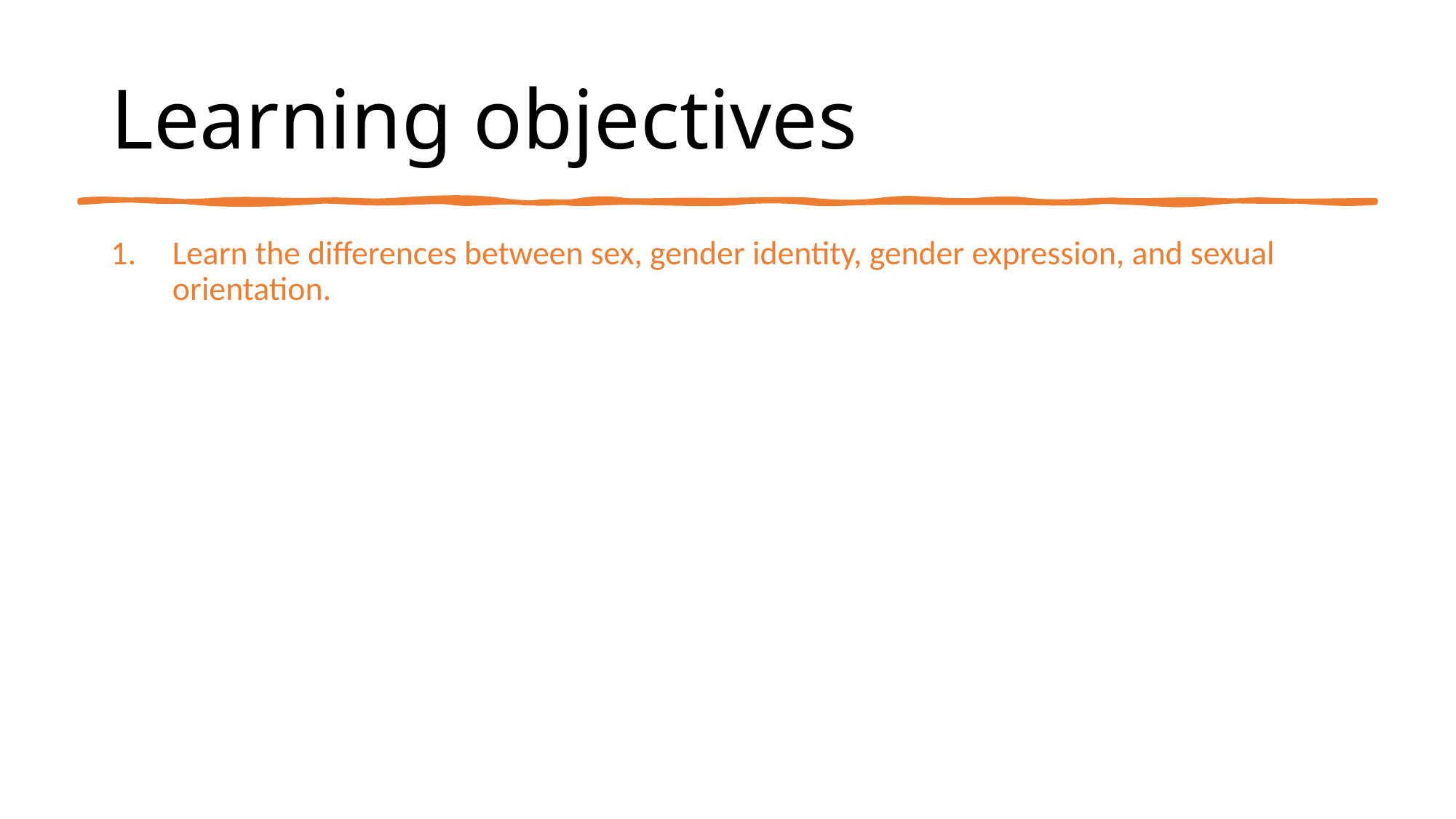

# Learning objectives
Learn the differences between sex, gender identity, gender expression, and sexual orientation.

## Slide 7
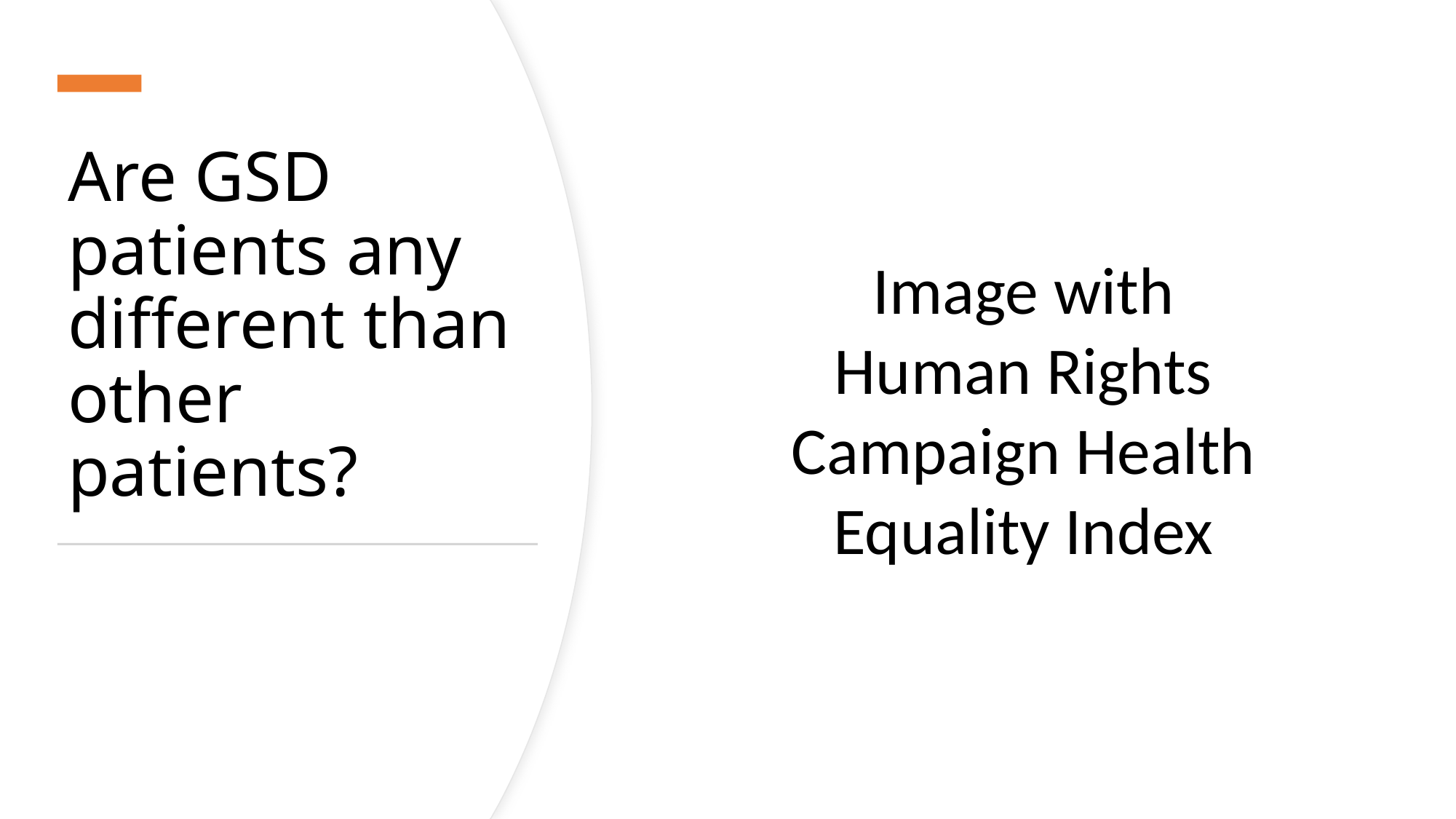

# Are GSD patients any different than other patients?
Image with Human Rights Campaign Health Equality Index

## Slide 8
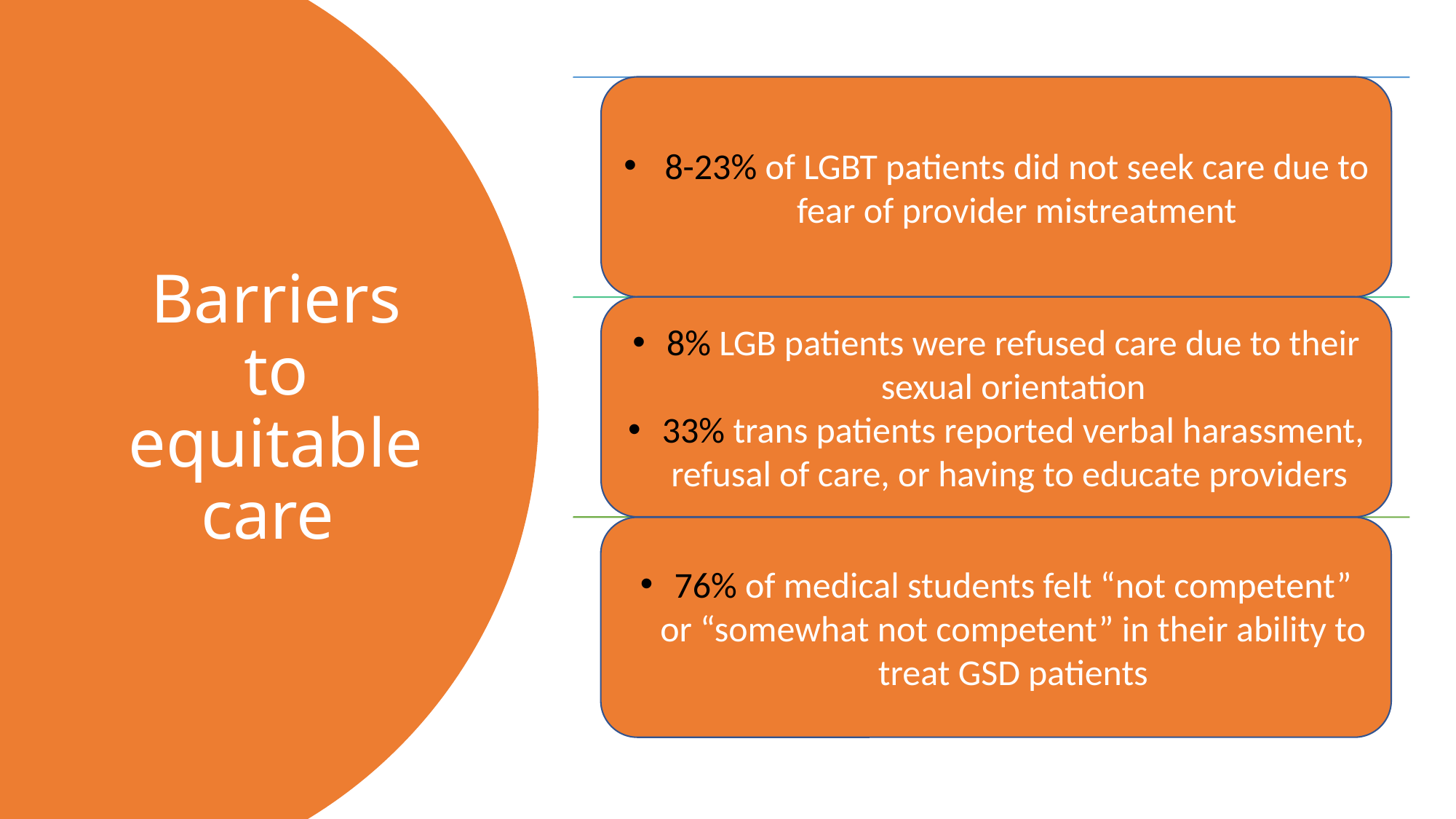

8-23% of LGBT patients did not seek care due to fear of provider mistreatment
# Barriers to equitable care
8% LGB patients were refused care due to their sexual orientation
33% trans patients reported verbal harassment, refusal of care, or having to educate providers
76% of medical students felt “not competent” or “somewhat not competent” in their ability to treat GSD patients

## Slide 9
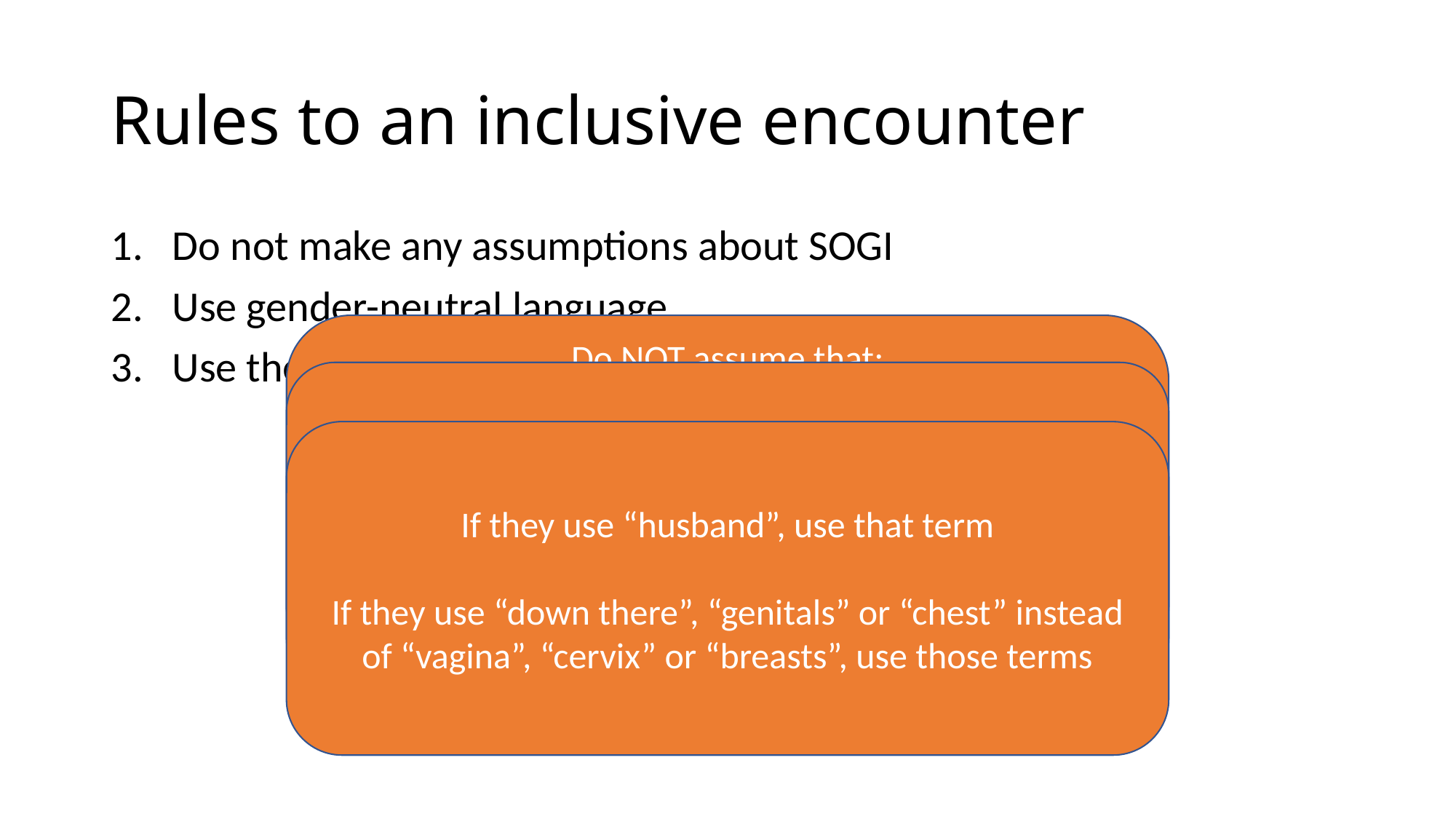

# Rules to an inclusive encounter
Do not make any assumptions about SOGI
Use gender-neutral language
Use the language that your patient uses
Do NOT assume that:
Being queer is always hard
Sexual orientation aligns with sexual behavior
All GSD patients are sexually active
All MSM are at high risk
GSD patients are not interested in parenting
Anything about the body of a GSD patient
Avoid words like “girlfriend” or “husband”,
Instead, use “partner” or “spouse”
If they use “husband”, use that term
If they use “down there”, “genitals” or “chest” instead of “vagina”, “cervix” or “breasts”, use those terms

## Slide 10
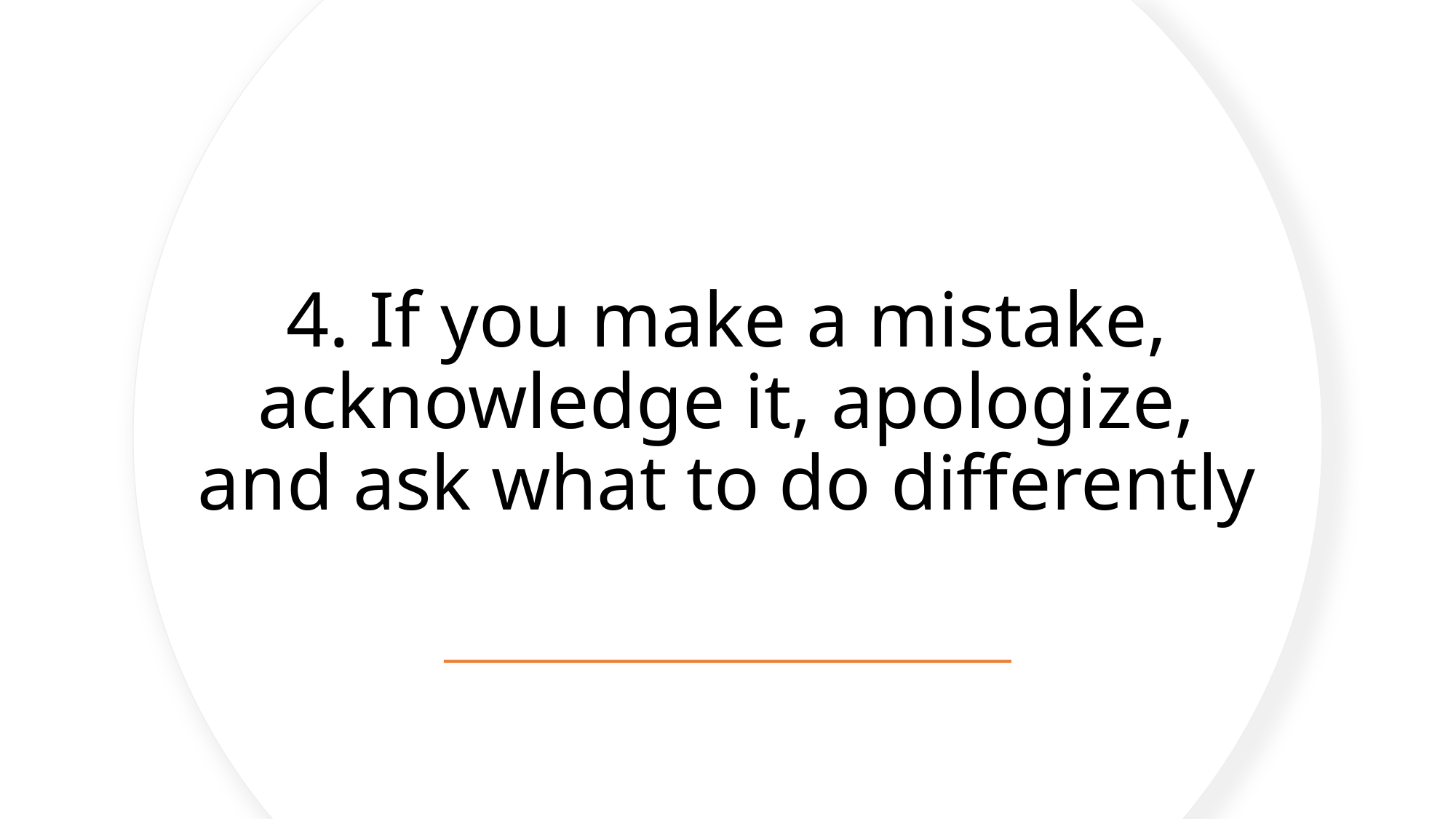

# 4. If you make a mistake, acknowledge it, apologize, and ask what to do differently

## Slide 11
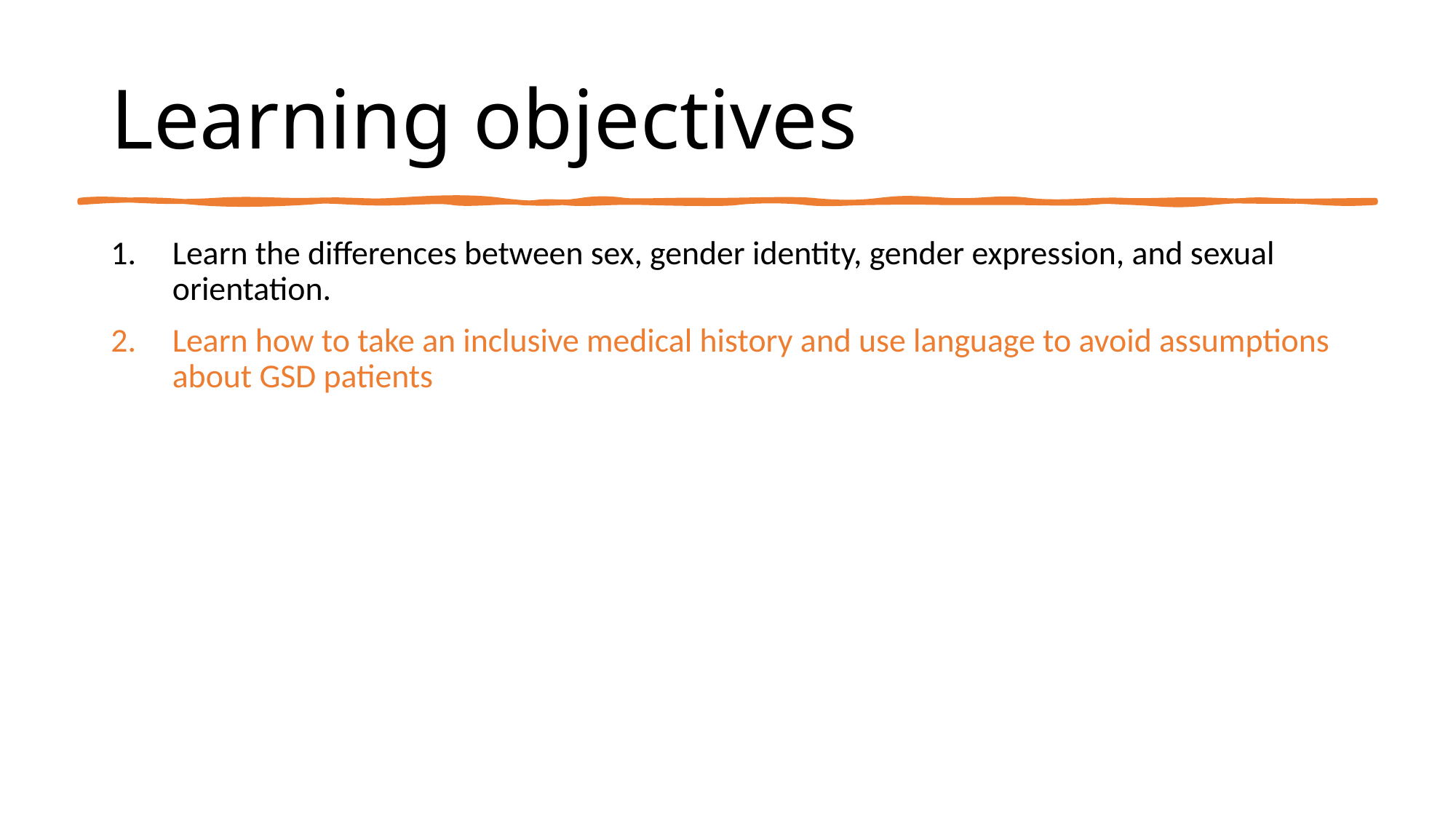

# Learning objectives
Learn the differences between sex, gender identity, gender expression, and sexual orientation.
Learn how to take an inclusive medical history and use language to avoid assumptions about GSD patients

## Slide 12
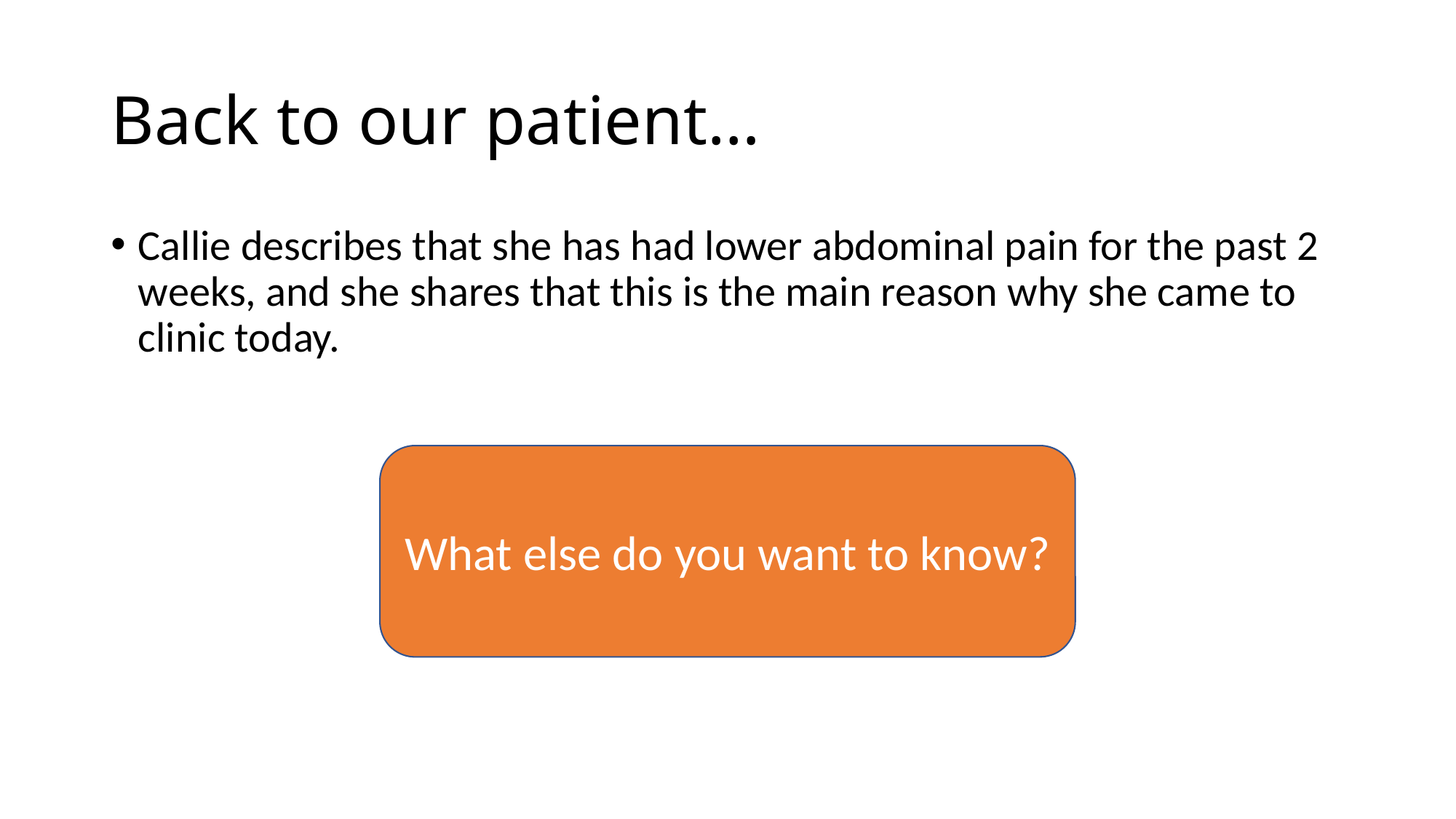

# Back to our patient…
Callie describes that she has had lower abdominal pain for the past 2 weeks, and she shares that this is the main reason why she came to clinic today.
What else do you want to know?

## Slide 13
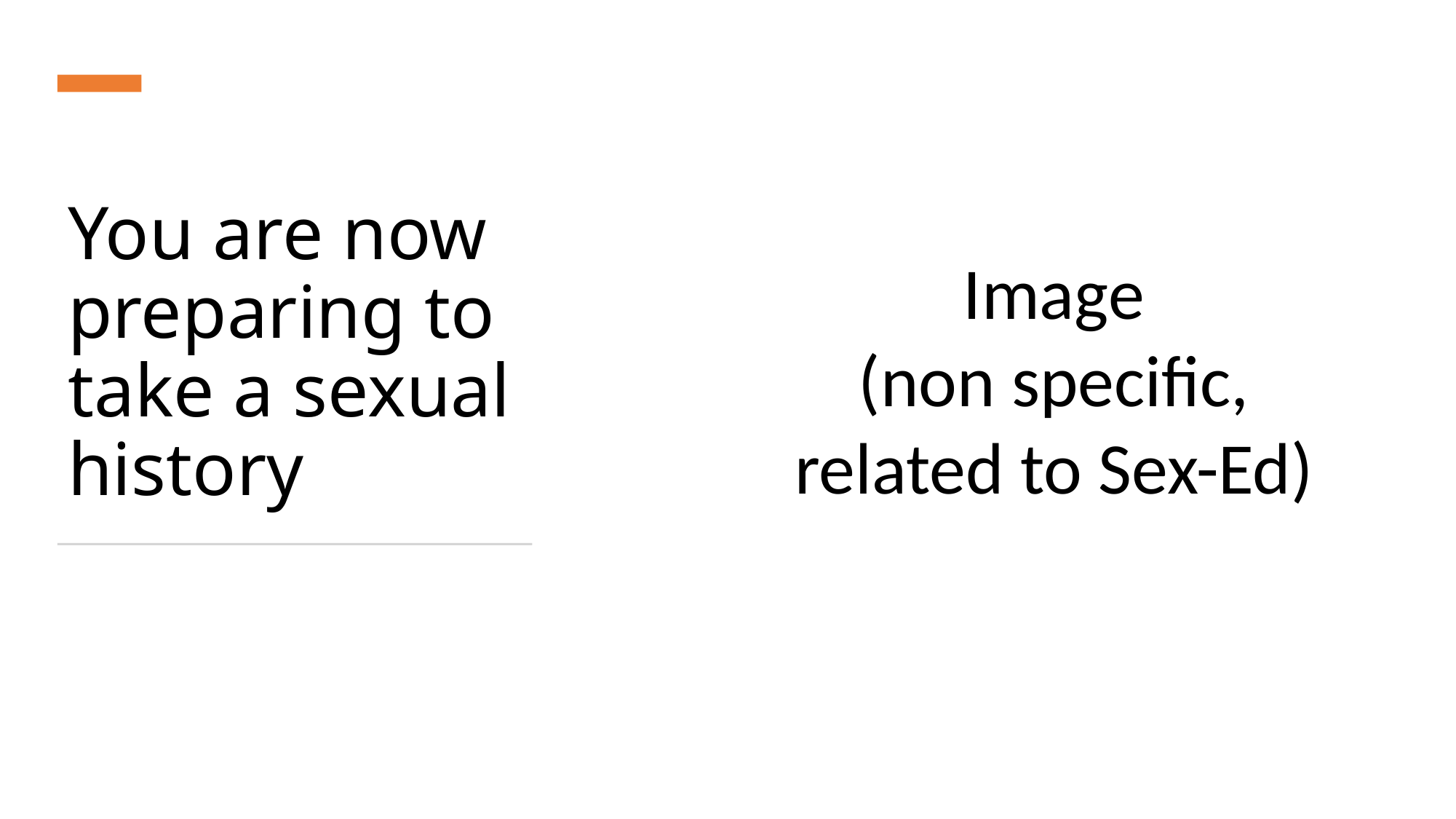

# You are now preparing to take a sexual history
Image
(non specific, related to Sex-Ed)

## Slide 14
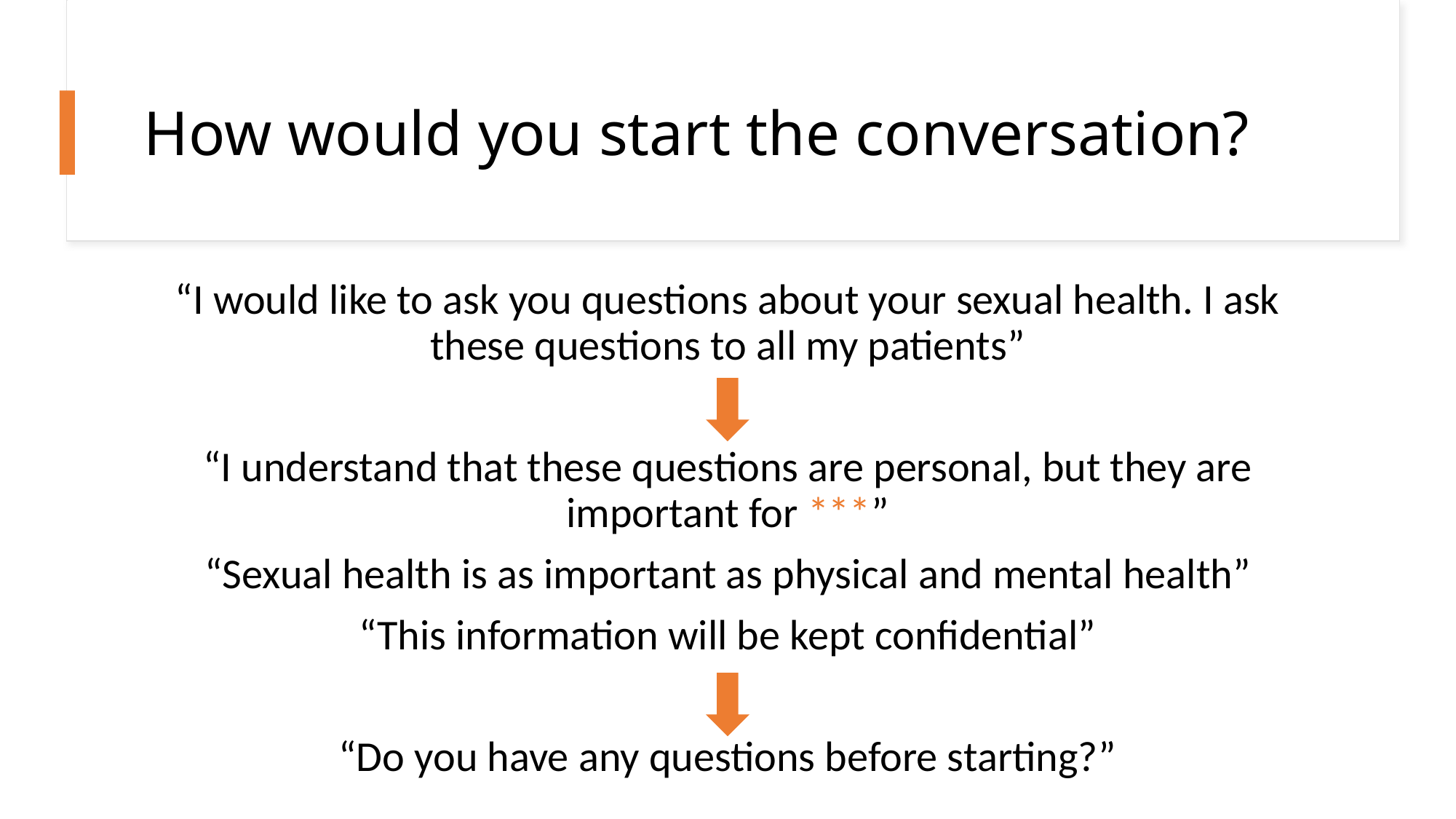

# How would you start the conversation?
“I would like to ask you questions about your sexual health. I ask these questions to all my patients”
“I understand that these questions are personal, but they are important for ***”
“Sexual health is as important as physical and mental health”
“This information will be kept confidential”
“Do you have any questions before starting?”

## Slide 15
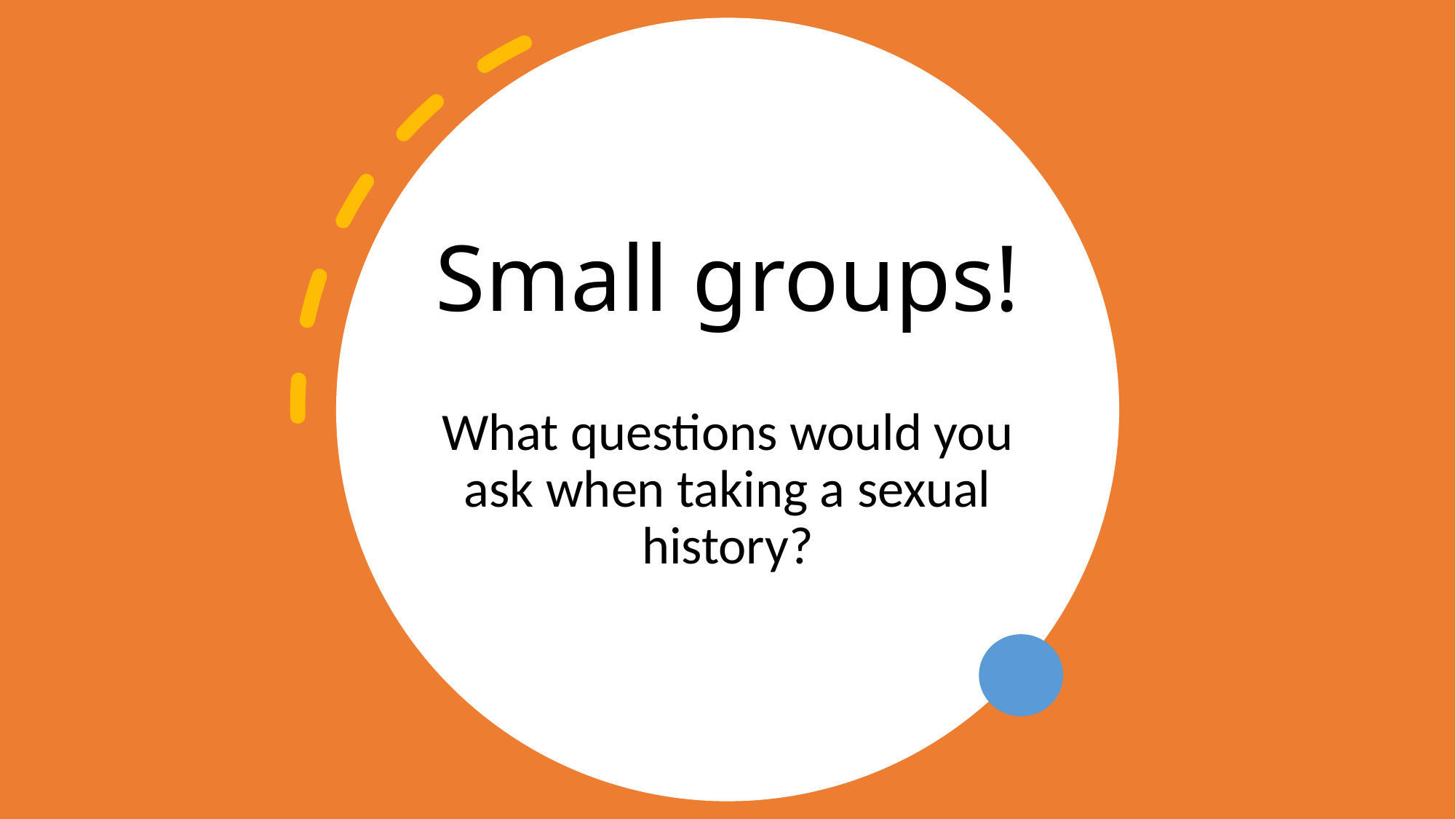

# Small groups!
What questions would you ask when taking a sexual history?

## Slide 16
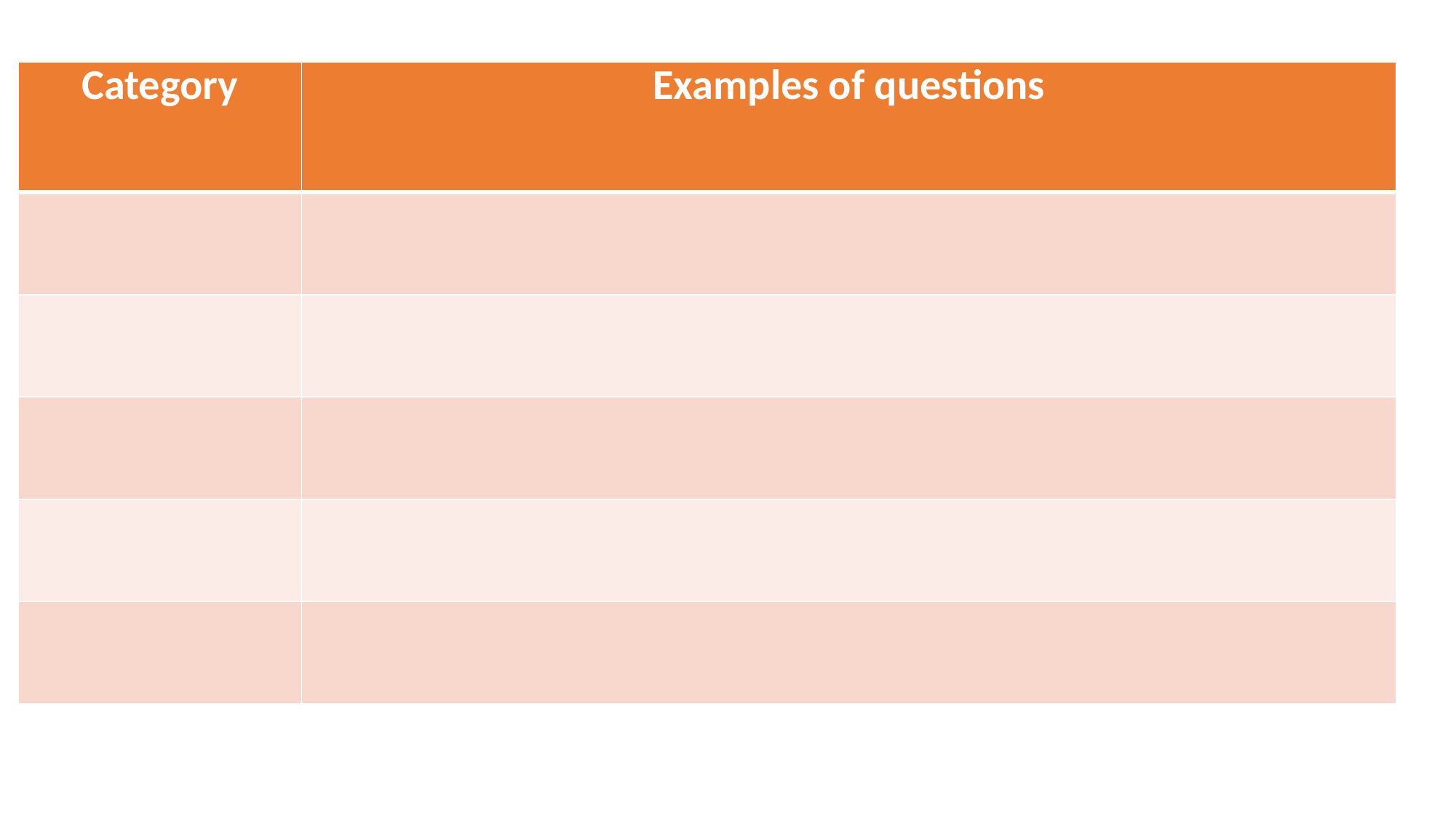

| Category | Examples of questions |
| --- | --- |
| | |
| | |
| | |
| | |
| | |

## Slide 17
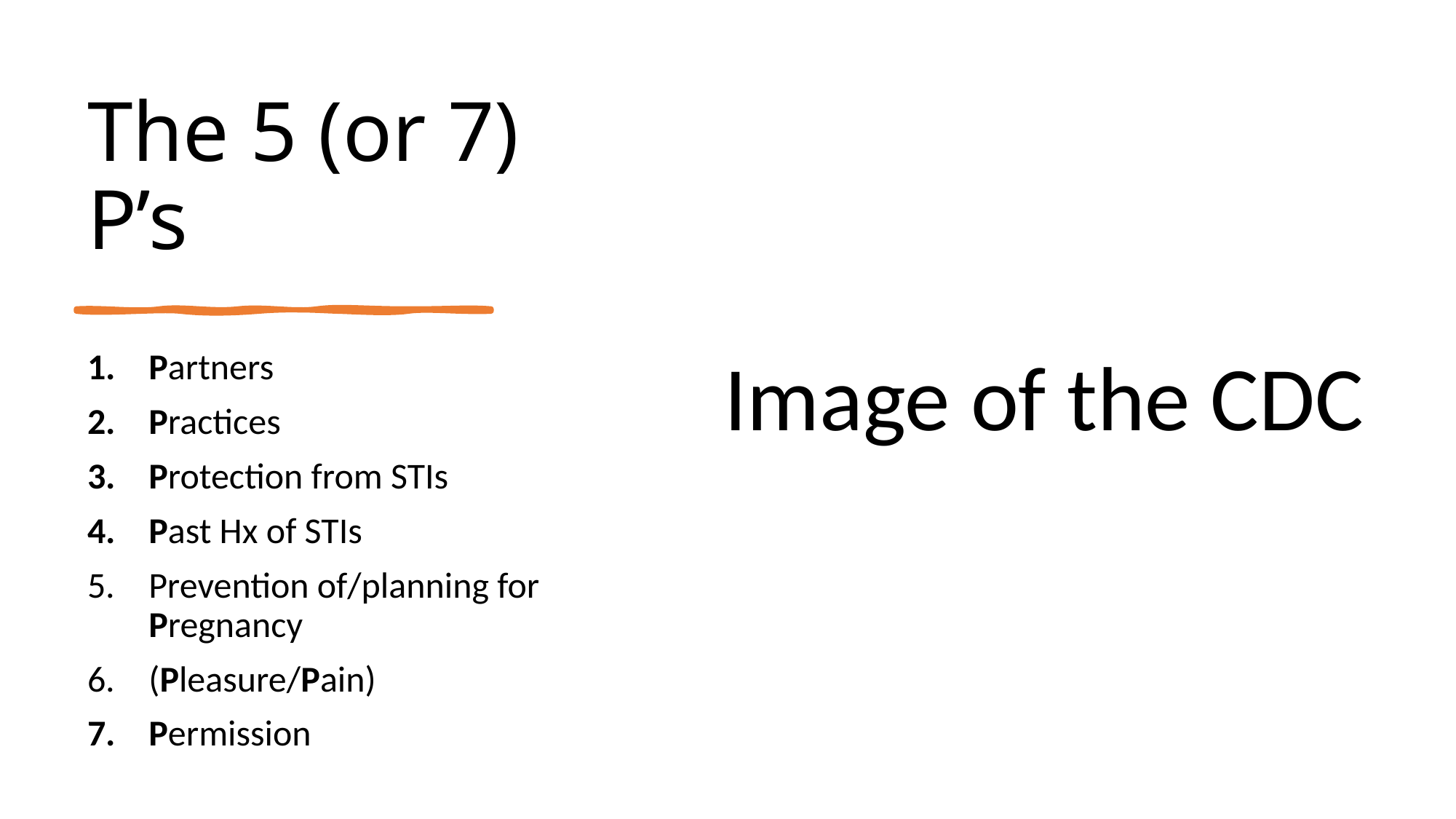

# The 5 (or 7) P’s
Image of the CDC
Partners
Practices
Protection from STIs
Past Hx of STIs
Prevention of/planning for Pregnancy
(Pleasure/Pain)
Permission

## Slide 18
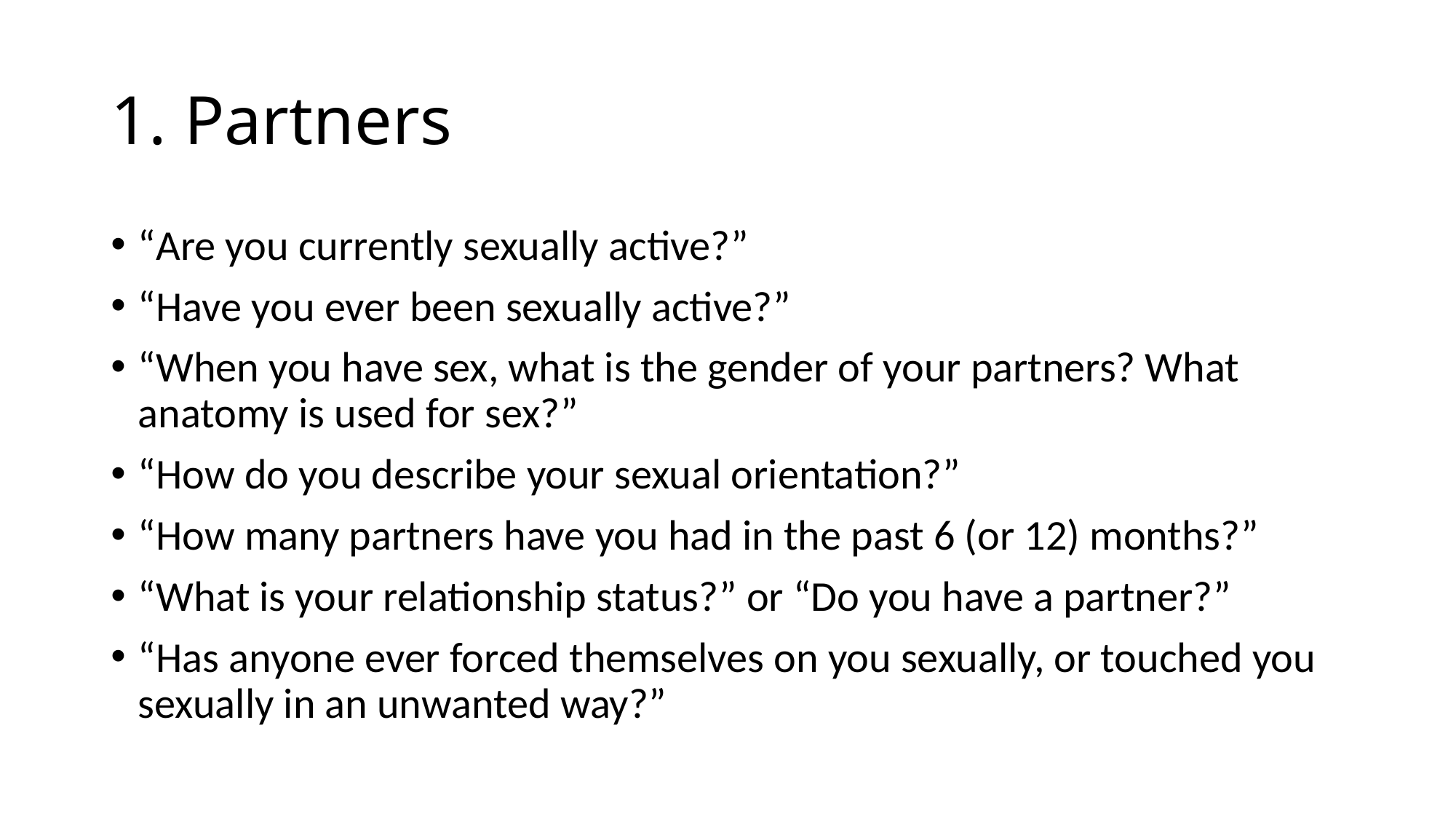

# 1. Partners
“Are you currently sexually active?”
“Have you ever been sexually active?”
“When you have sex, what is the gender of your partners? What anatomy is used for sex?”
“How do you describe your sexual orientation?”
“How many partners have you had in the past 6 (or 12) months?”
“What is your relationship status?” or “Do you have a partner?”
“Has anyone ever forced themselves on you sexually, or touched you sexually in an unwanted way?”

## Slide 19
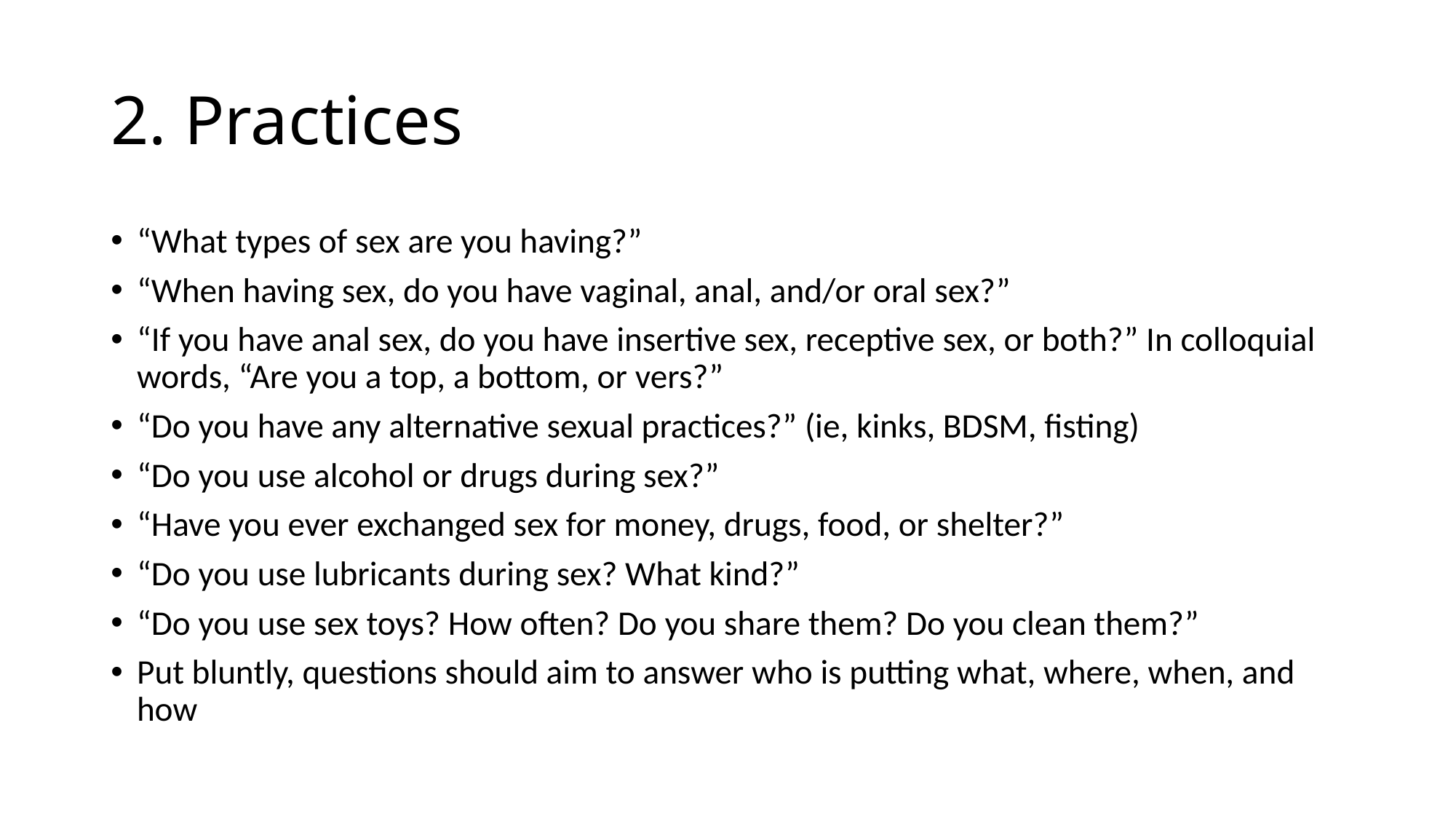

# 2. Practices
“What types of sex are you having?”
“When having sex, do you have vaginal, anal, and/or oral sex?”
“If you have anal sex, do you have insertive sex, receptive sex, or both?” In colloquial words, “Are you a top, a bottom, or vers?”
“Do you have any alternative sexual practices?” (ie, kinks, BDSM, fisting)
“Do you use alcohol or drugs during sex?”
“Have you ever exchanged sex for money, drugs, food, or shelter?”
“Do you use lubricants during sex? What kind?”
“Do you use sex toys? How often? Do you share them? Do you clean them?”
Put bluntly, questions should aim to answer who is putting what, where, when, and how

## Slide 20
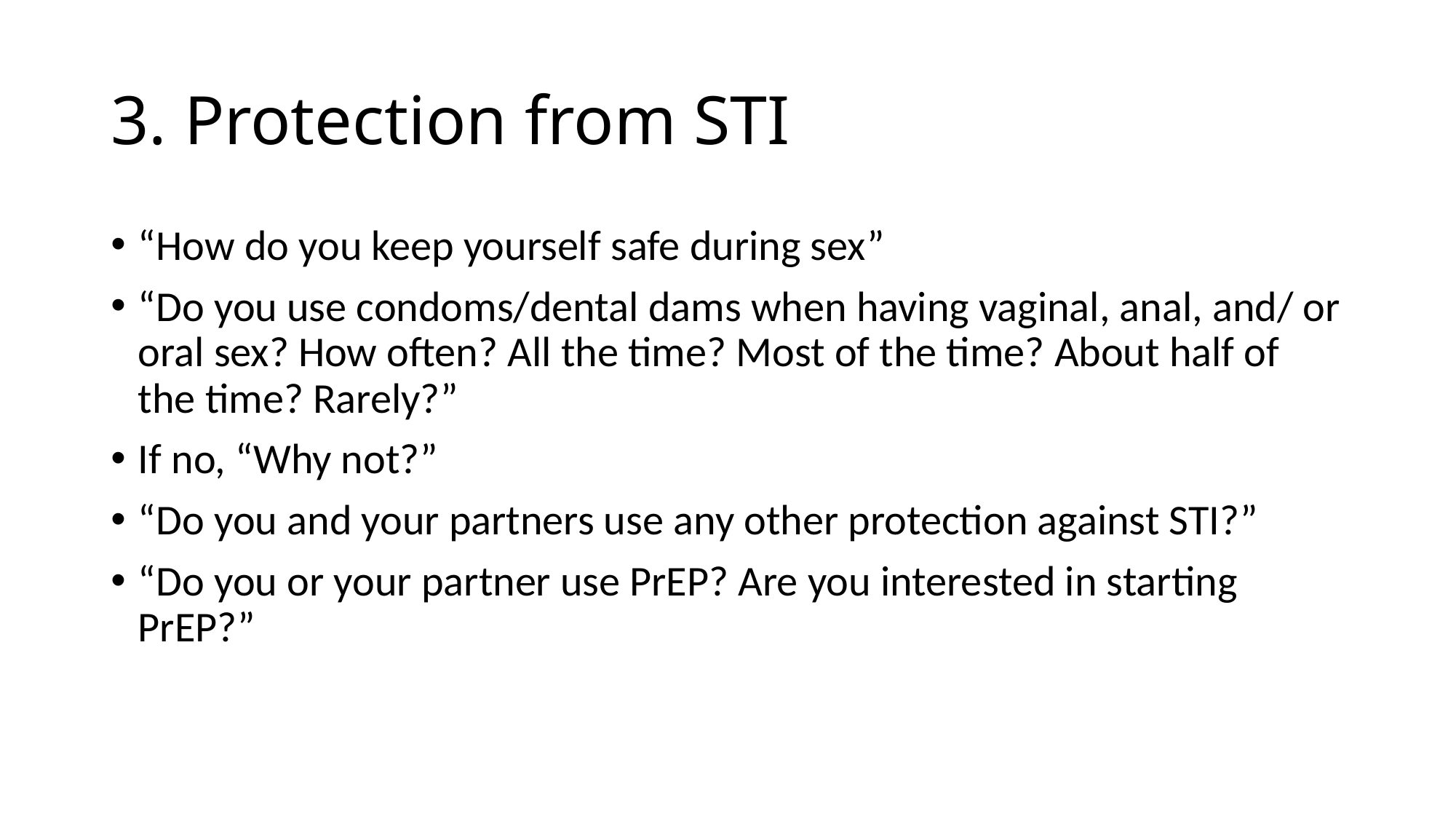

# 3. Protection from STI
“How do you keep yourself safe during sex”
“Do you use condoms/dental dams when having vaginal, anal, and/ or oral sex? How often? All the time? Most of the time? About half of the time? Rarely?”
If no, “Why not?”
“Do you and your partners use any other protection against STI?”
“Do you or your partner use PrEP? Are you interested in starting PrEP?”

## Slide 21
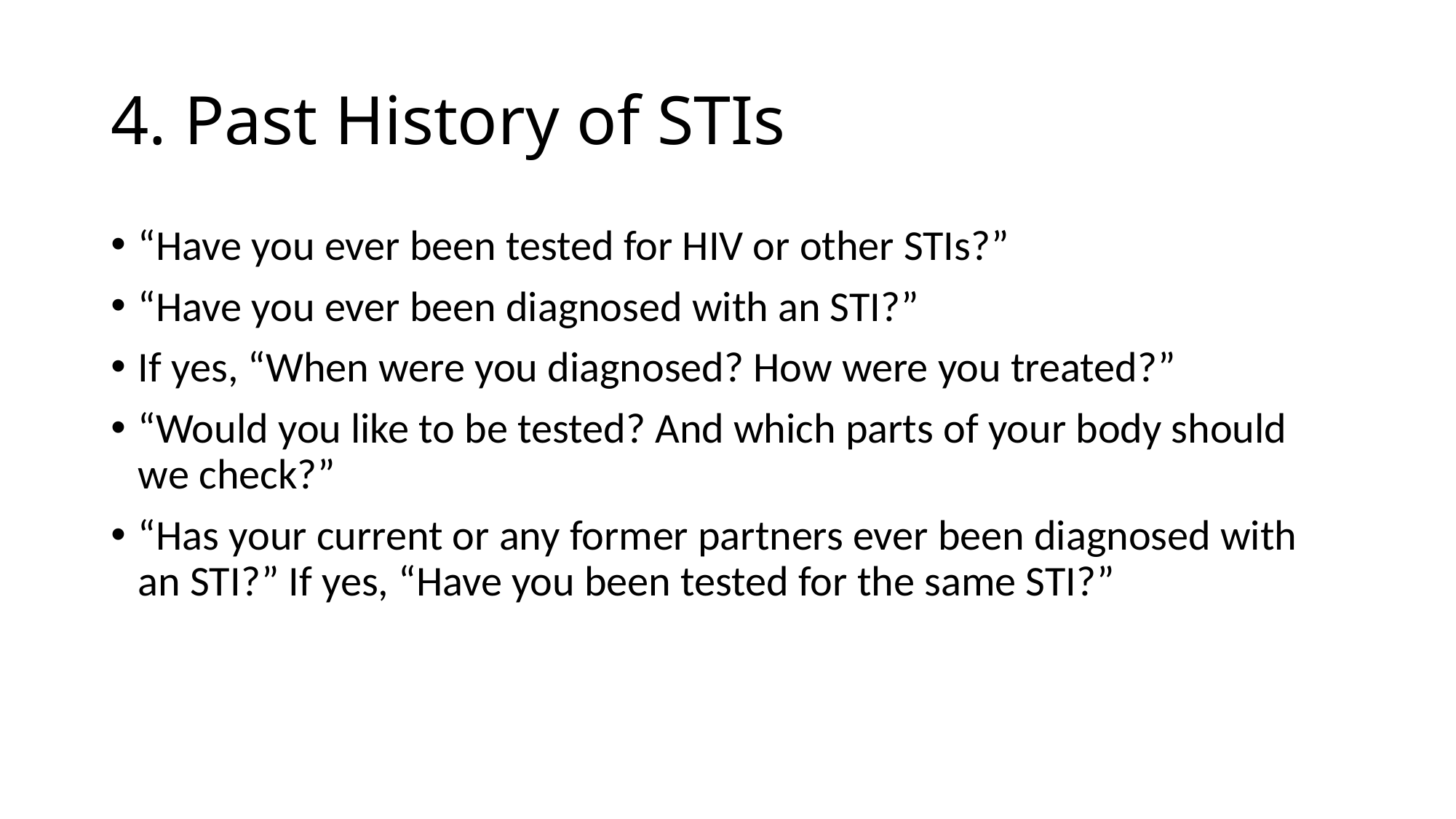

# 4. Past History of STIs
“Have you ever been tested for HIV or other STIs?”
“Have you ever been diagnosed with an STI?”
If yes, “When were you diagnosed? How were you treated?”
“Would you like to be tested? And which parts of your body should we check?”
“Has your current or any former partners ever been diagnosed with an STI?” If yes, “Have you been tested for the same STI?”

## Slide 22
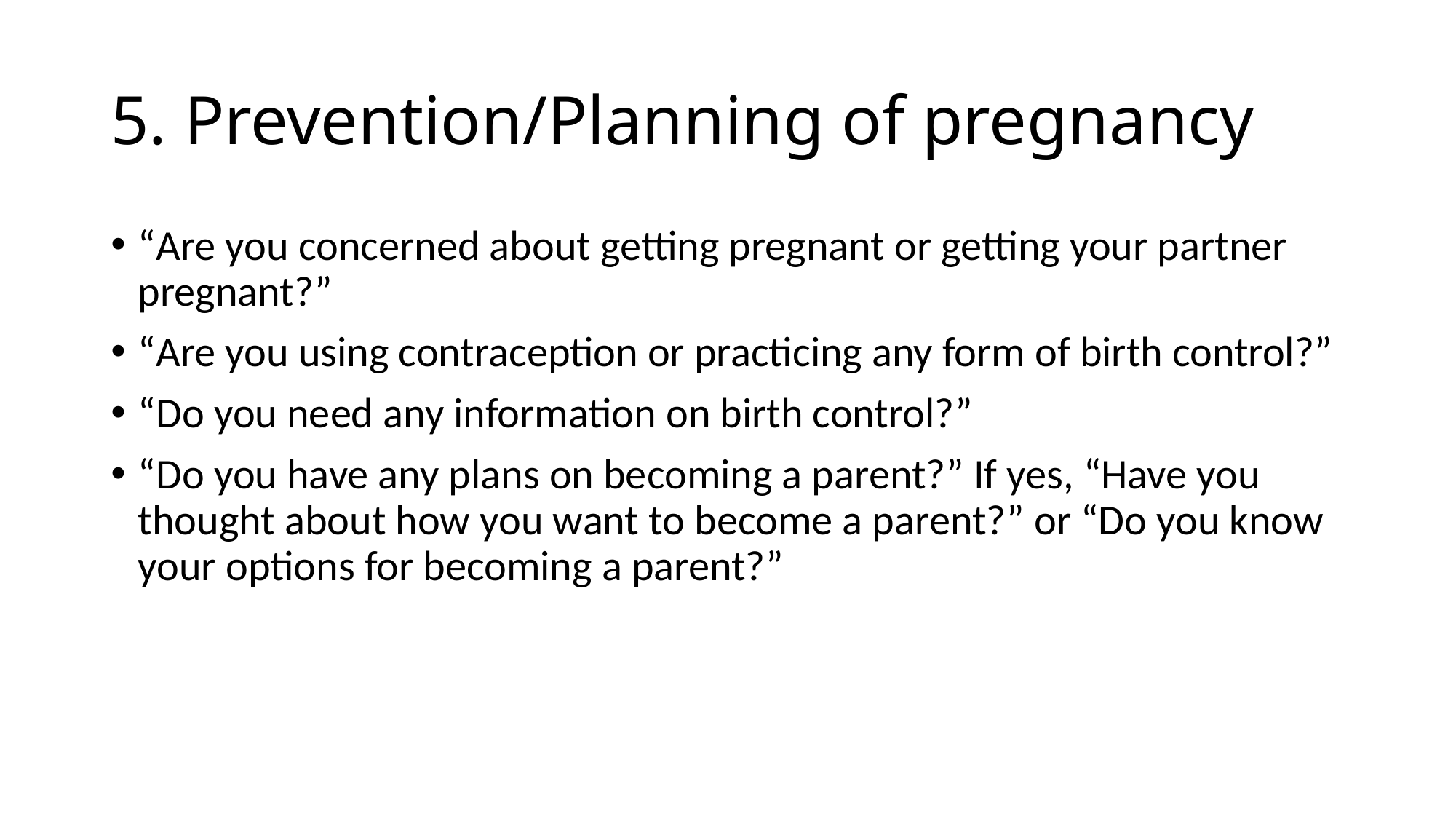

# 5. Prevention/Planning of pregnancy
“Are you concerned about getting pregnant or getting your partner pregnant?”
“Are you using contraception or practicing any form of birth control?”
“Do you need any information on birth control?”
“Do you have any plans on becoming a parent?” If yes, “Have you thought about how you want to become a parent?” or “Do you know your options for becoming a parent?”

## Slide 23
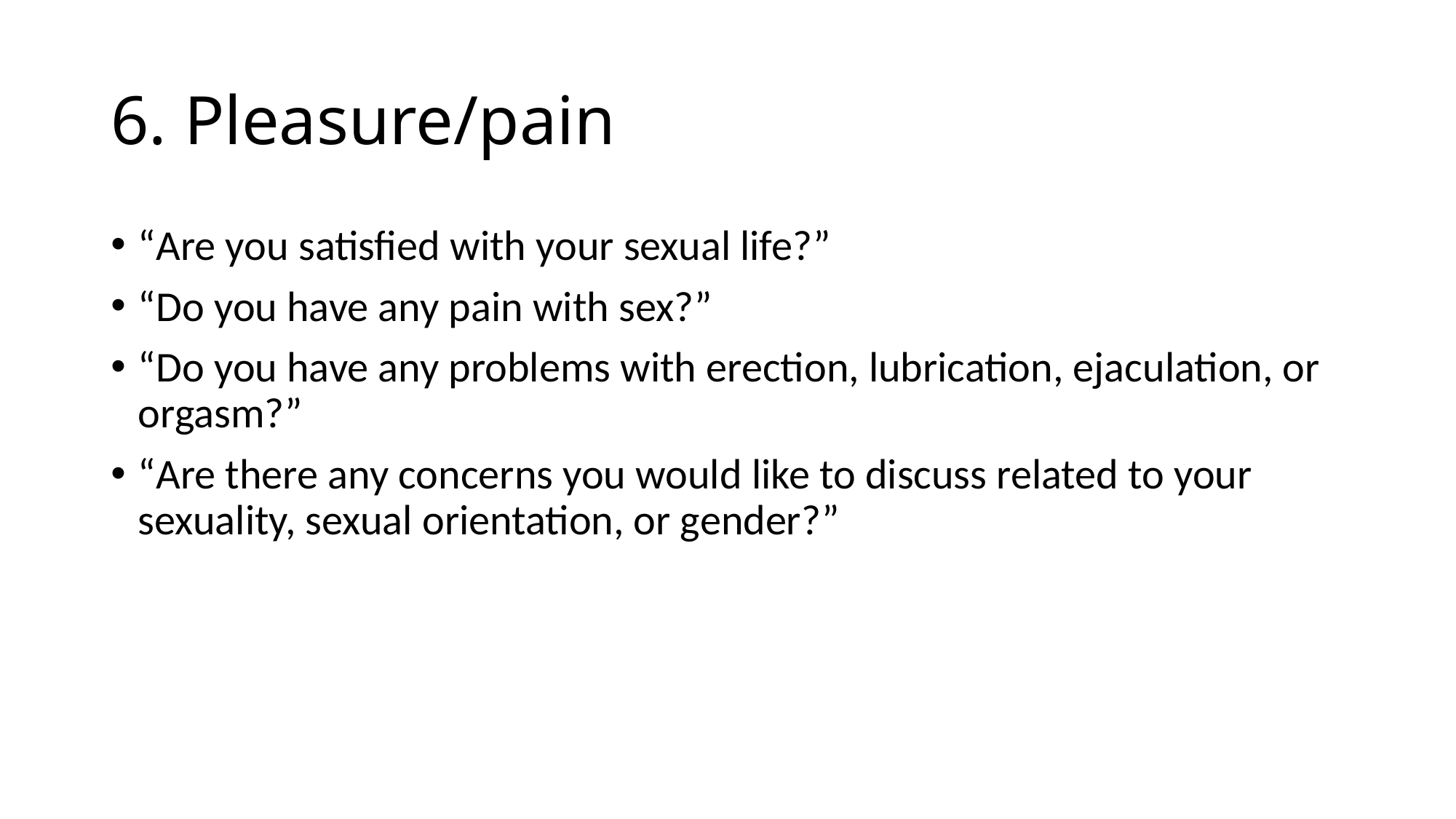

# 6. Pleasure/pain
“Are you satisfied with your sexual life?”
“Do you have any pain with sex?”
“Do you have any problems with erection, lubrication, ejaculation, or orgasm?”
“Are there any concerns you would like to discuss related to your sexuality, sexual orientation, or gender?”

## Slide 24
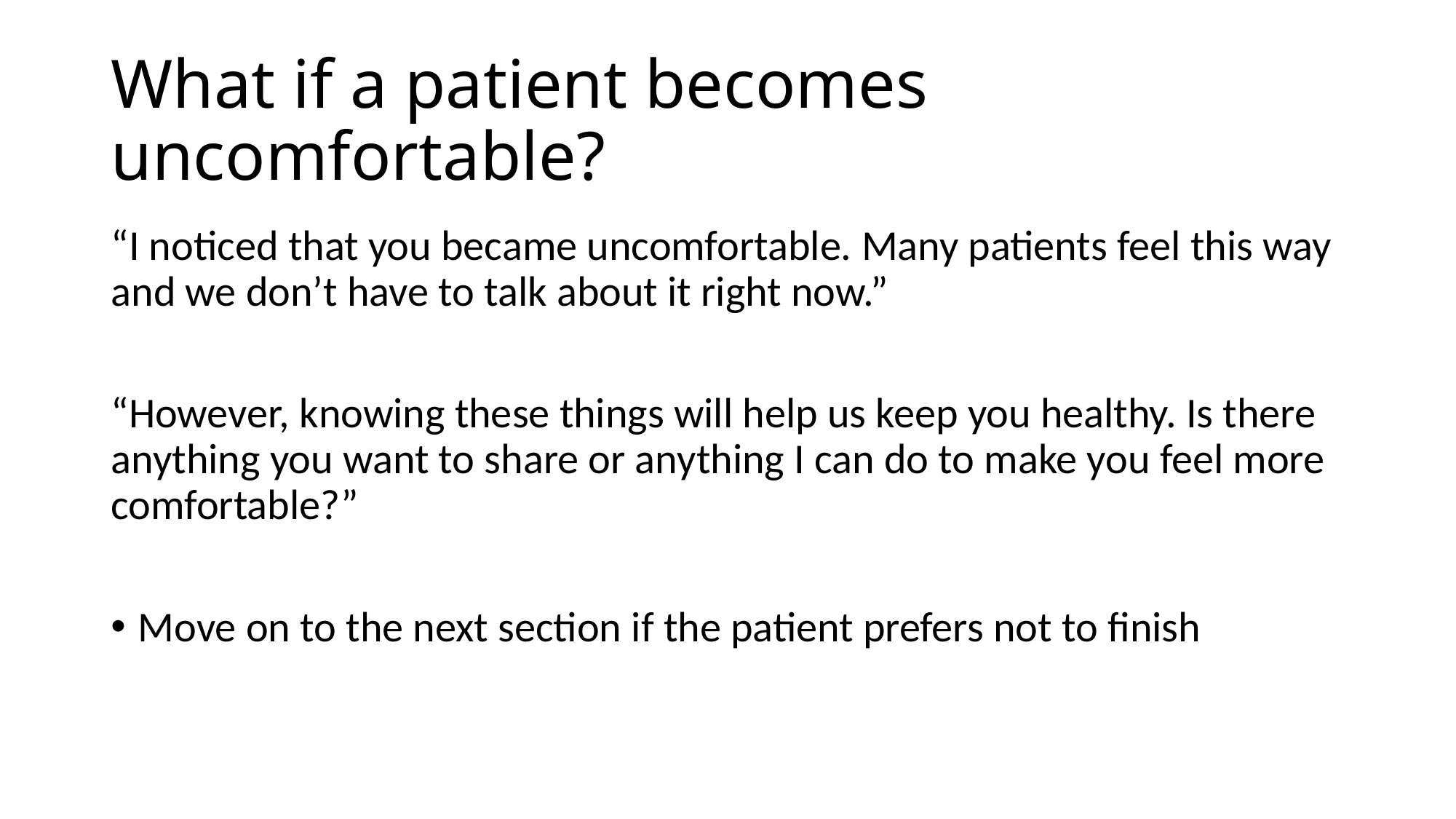

# What if a patient becomes uncomfortable?
“I noticed that you became uncomfortable. Many patients feel this way and we don’t have to talk about it right now.”
“However, knowing these things will help us keep you healthy. Is there anything you want to share or anything I can do to make you feel more comfortable?”
Move on to the next section if the patient prefers not to finish

## Slide 25
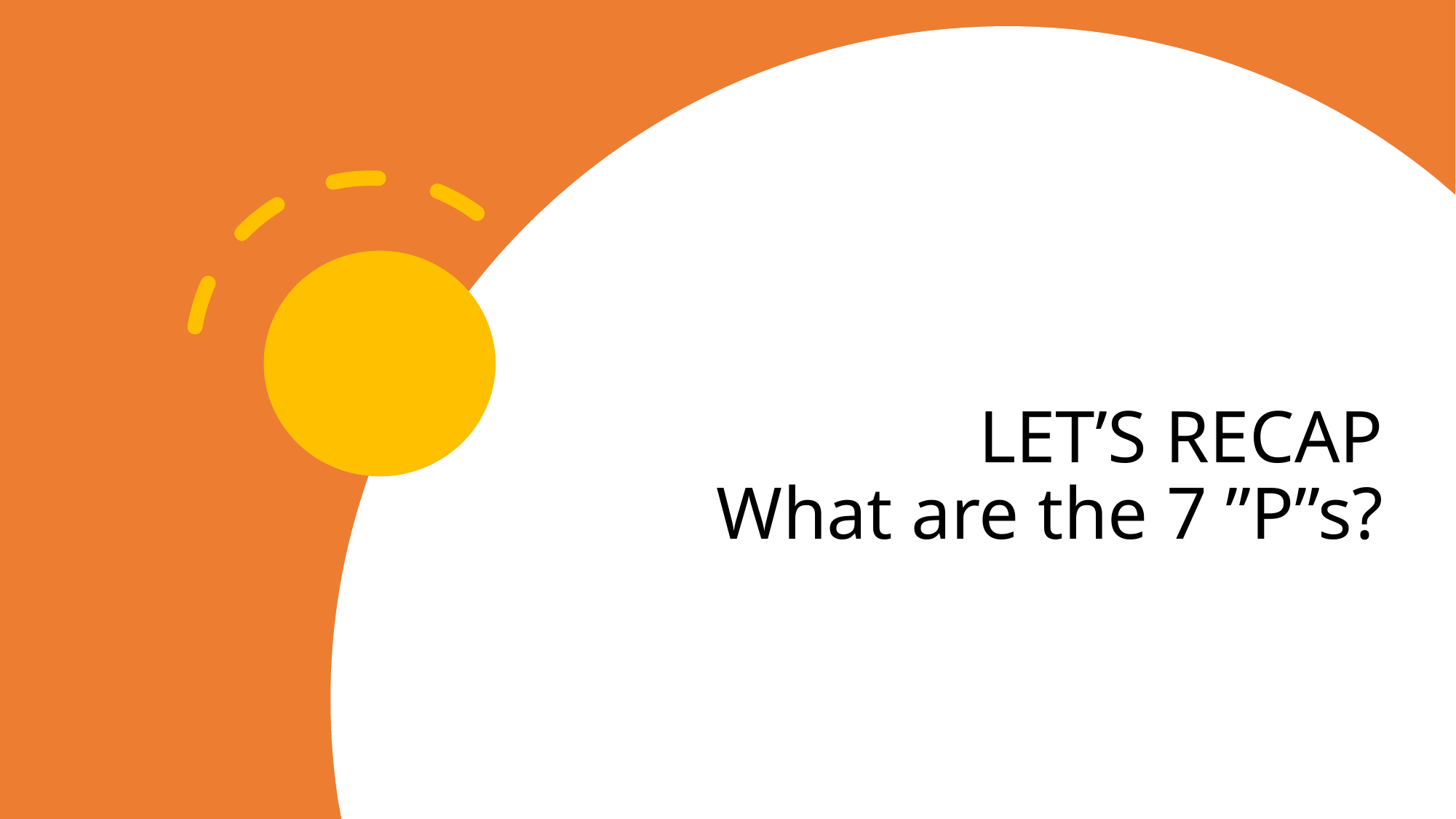

# LET’S RECAP What are the 7 ”P”s?

## Slide 26
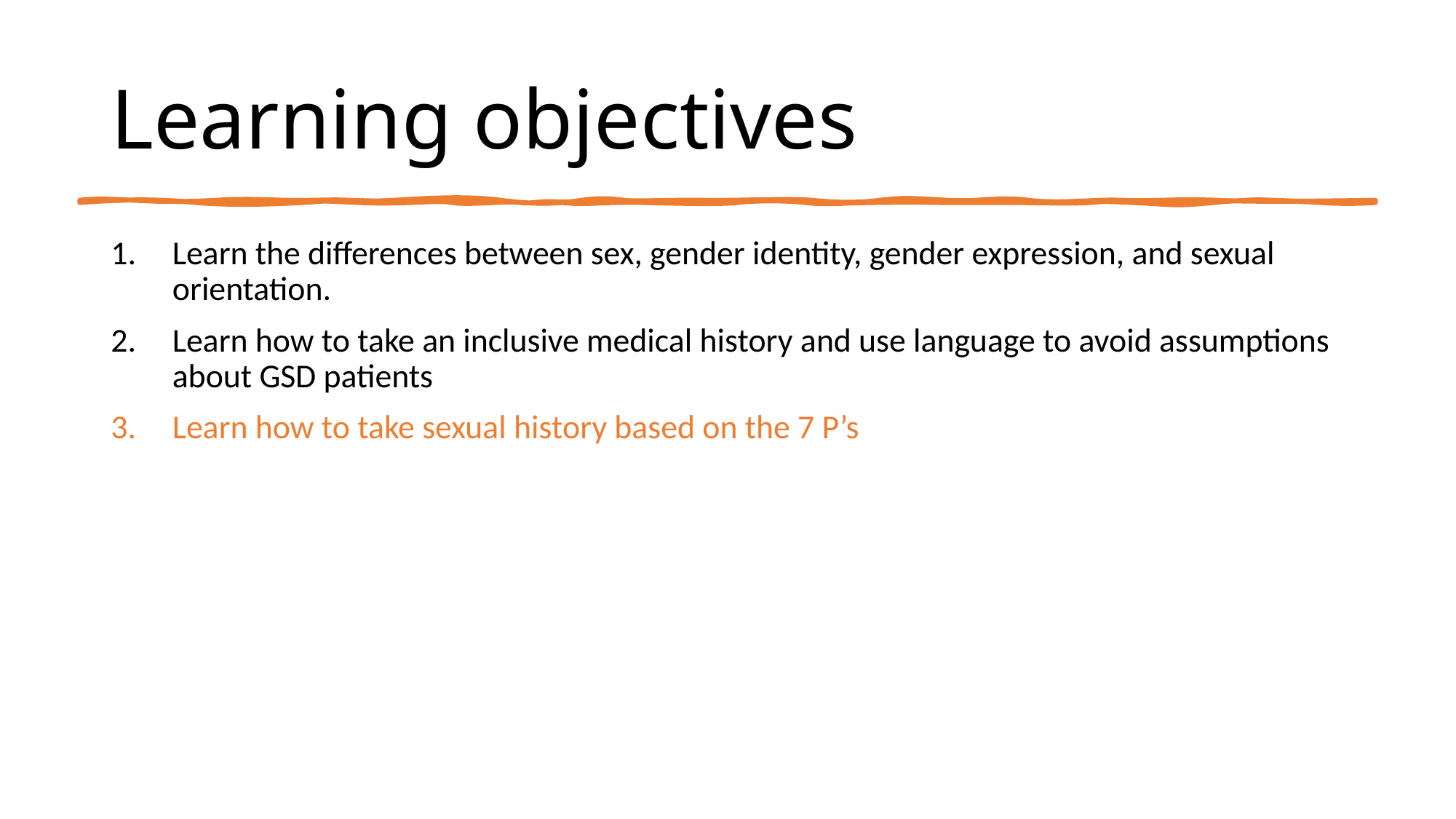

# Learning objectives
Learn the differences between sex, gender identity, gender expression, and sexual orientation.
Learn how to take an inclusive medical history and use language to avoid assumptions about GSD patients
Learn how to take sexual history based on the 7 P’s

## Slide 27
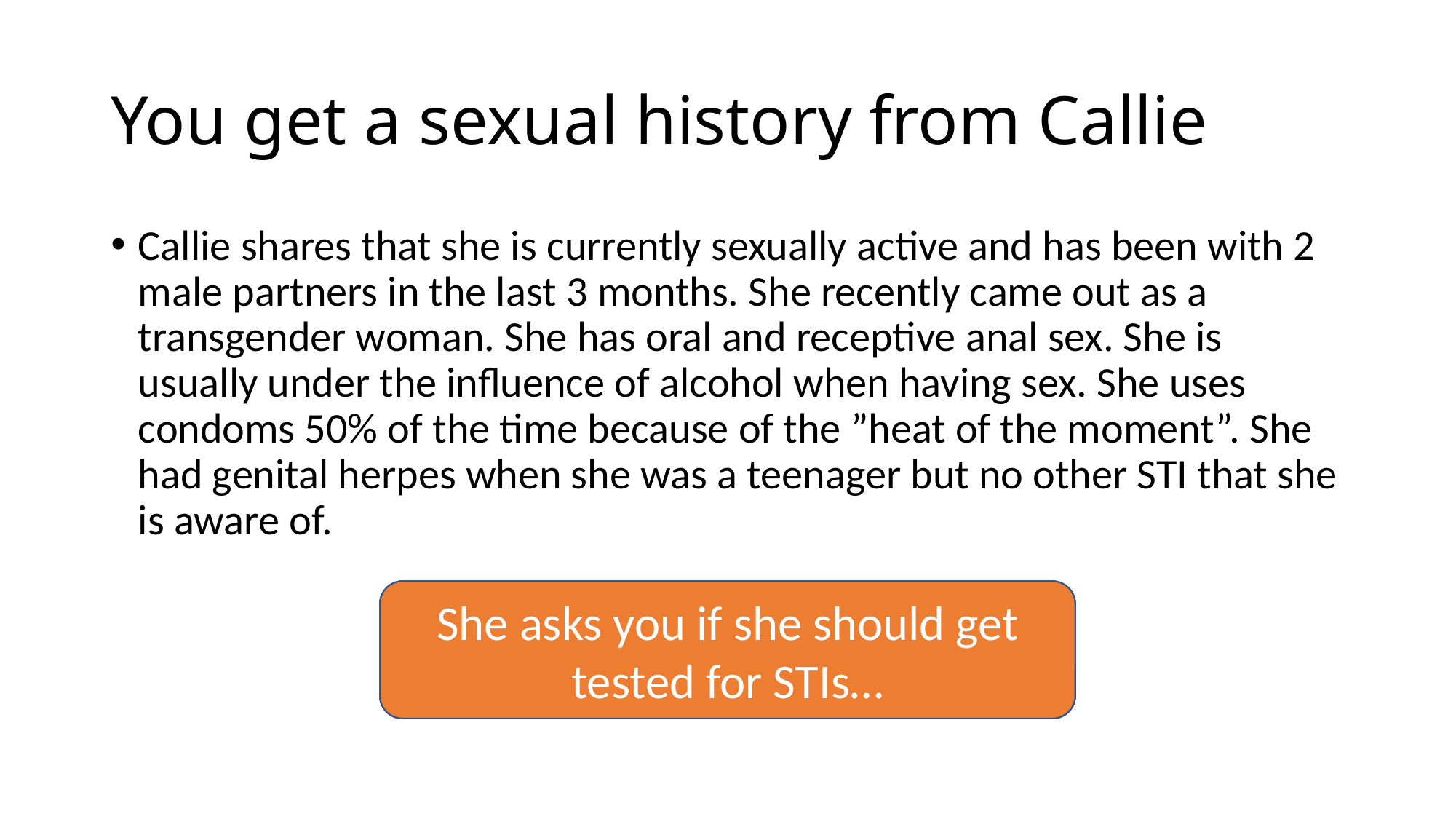

# You get a sexual history from Callie
Callie shares that she is currently sexually active and has been with 2 male partners in the last 3 months. She recently came out as a transgender woman. She has oral and receptive anal sex. She is usually under the influence of alcohol when having sex. She uses condoms 50% of the time because of the ”heat of the moment”. She had genital herpes when she was a teenager but no other STI that she is aware of.
What is her sexual orientation?
She asks you if she should get tested for STIs…
What is her sex?
What is her gender identity?

## Slide 28
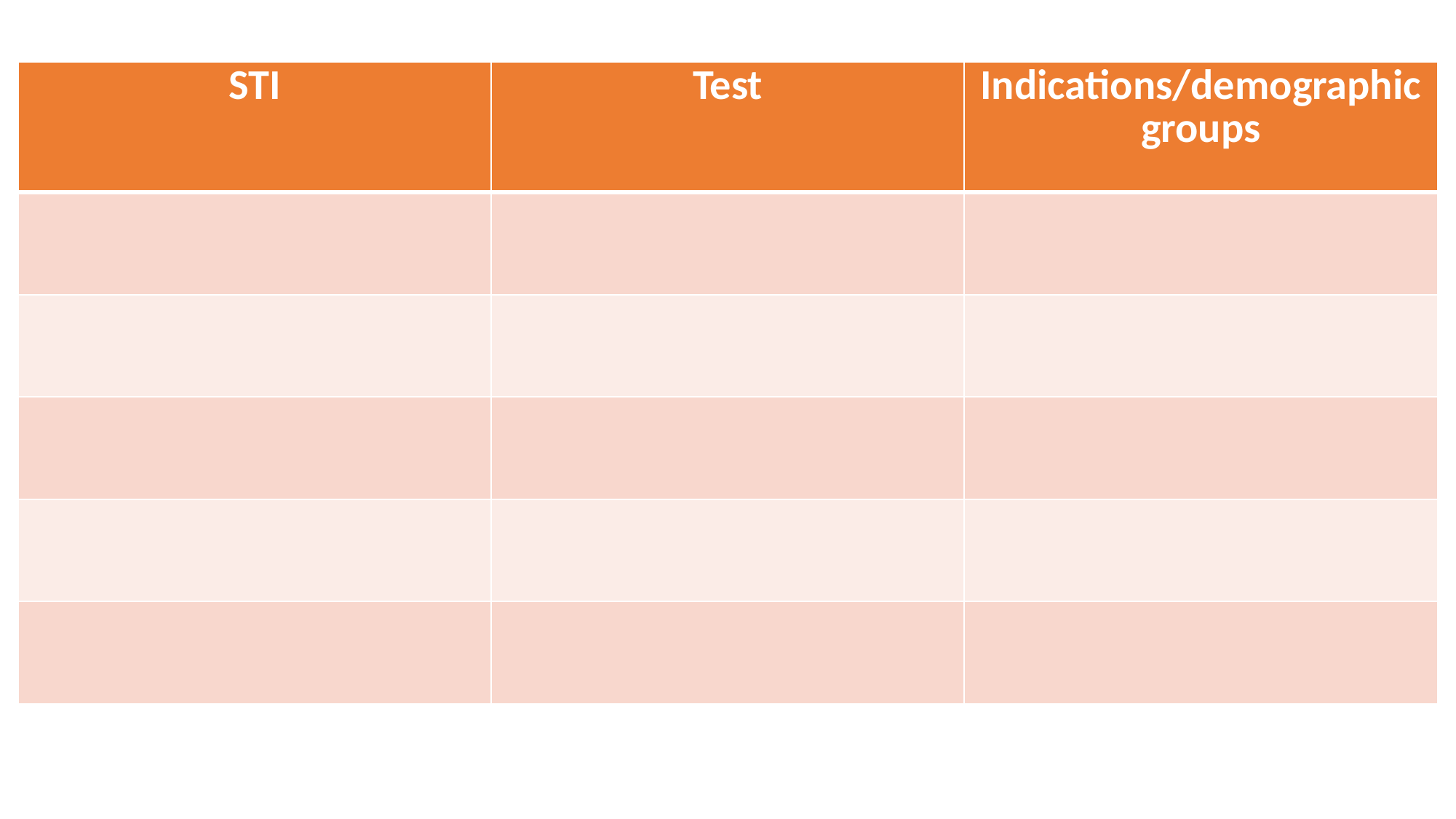

| STI | Test | Indications/demographic groups |
| --- | --- | --- |
| | | |
| | | |
| | | |
| | | |
| | | |

## Slide 29
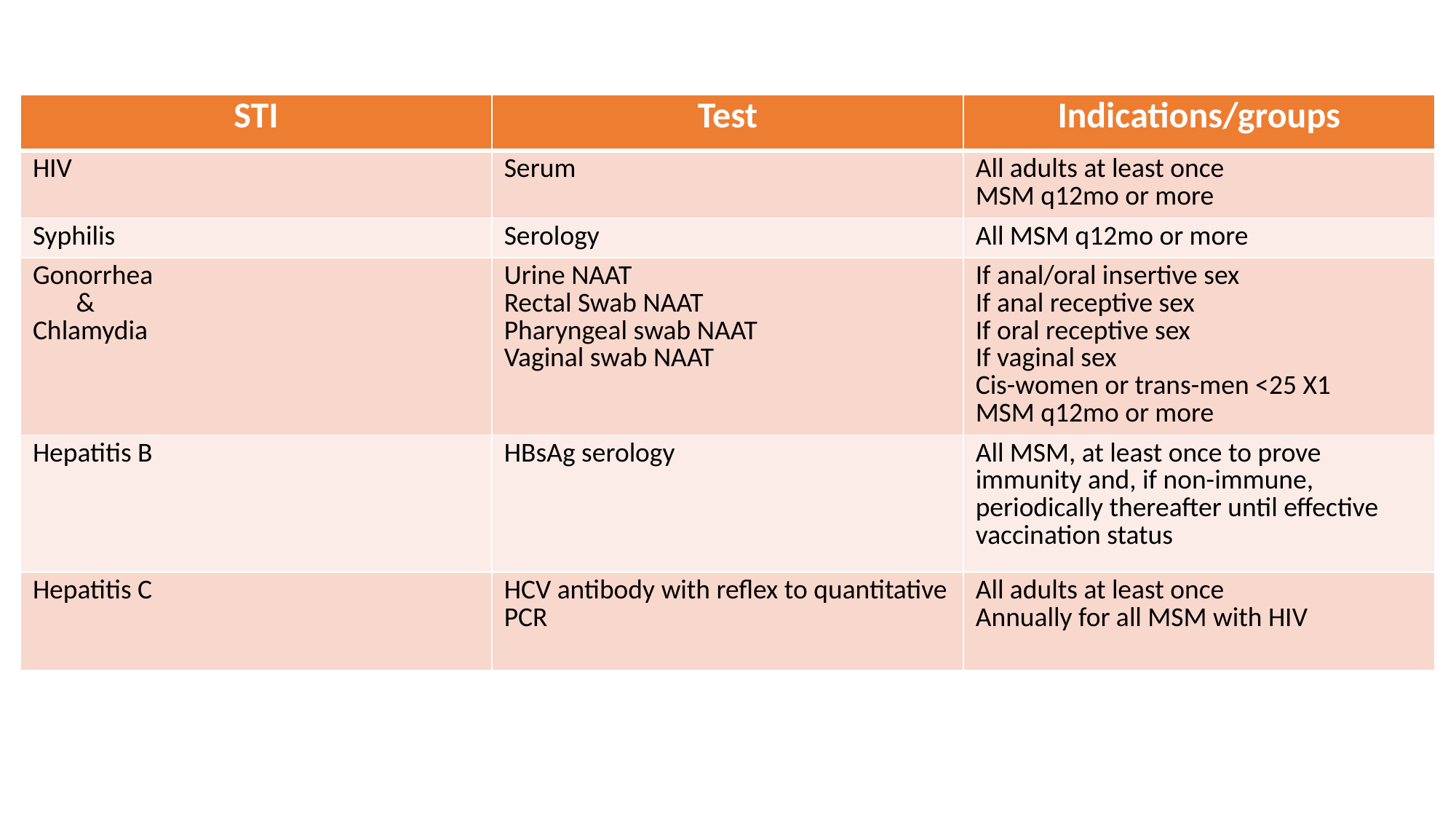

| STI | Test | Indications/groups |
| --- | --- | --- |
| HIV | Serum | All adults at least once MSM q12mo or more |
| Syphilis | Serology | All MSM q12mo or more |
| Gonorrhea & Chlamydia | Urine NAAT Rectal Swab NAAT Pharyngeal swab NAAT Vaginal swab NAAT | If anal/oral insertive sex If anal receptive sex If oral receptive sex If vaginal sex Cis-women or trans-men <25 X1 MSM q12mo or more |
| Hepatitis B | HBsAg serology | All MSM, at least once to prove immunity and, if non-immune, periodically thereafter until effective vaccination status |
| Hepatitis C | HCV antibody with reflex to quantitative PCR | All adults at least once Annually for all MSM with HIV |

## Slide 30
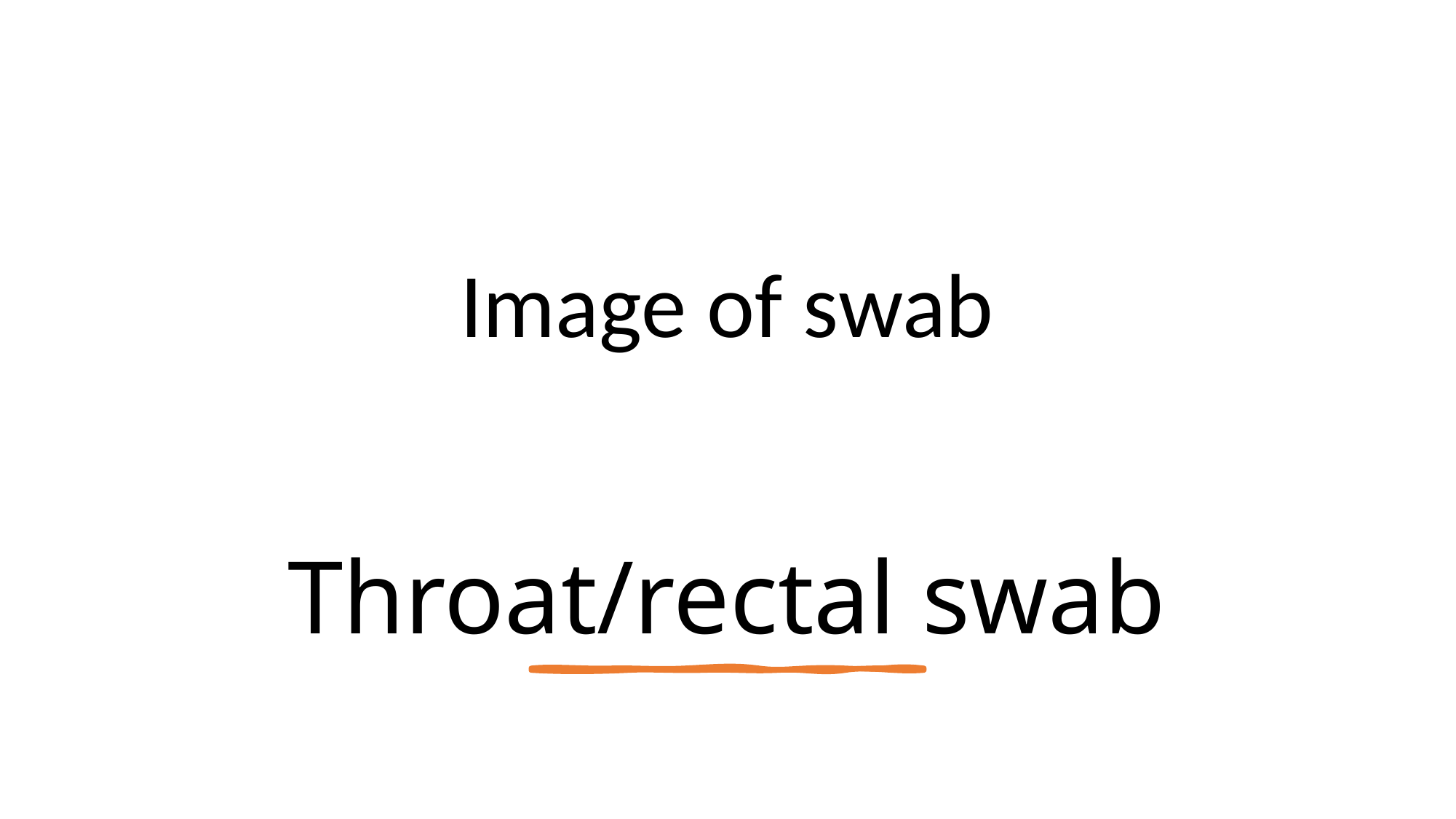

Image of swab
# Throat/rectal swab

## Slide 31
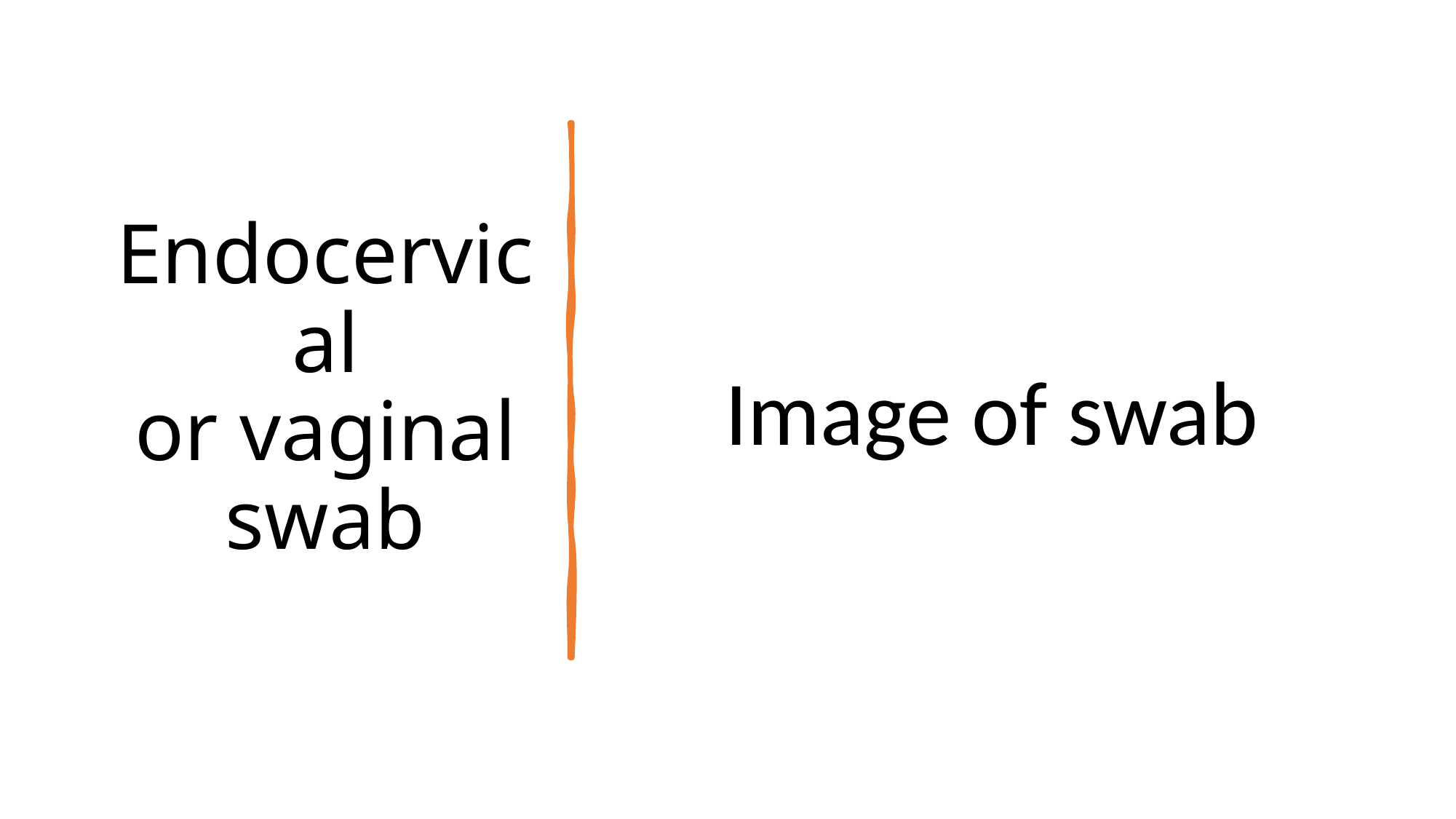

# Endocervical or vaginal swab
Image of swab

## Slide 32
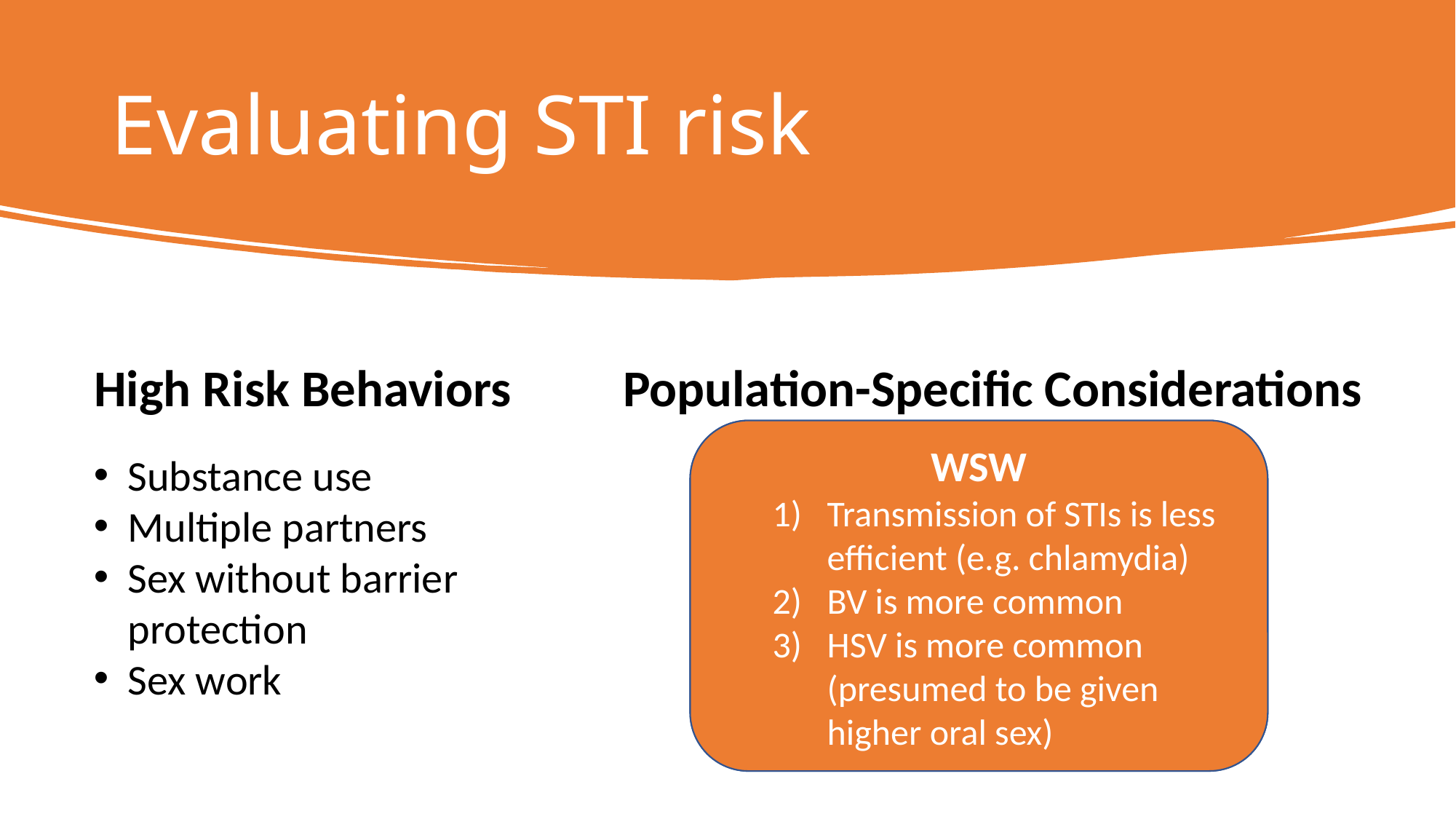

# Evaluating STI risk
High Risk Behaviors
Population-Specific Considerations
MSM and trans women
Rectal mucosa is more susceptible to transmission
High prevalence of HIV and gonorrhea in the community
WSW
Transmission of STIs is less efficient (e.g. chlamydia)
BV is more common
HSV is more common (presumed to be given higher oral sex)
Substance use
Multiple partners
Sex without barrier protection
Sex work

## Slide 33
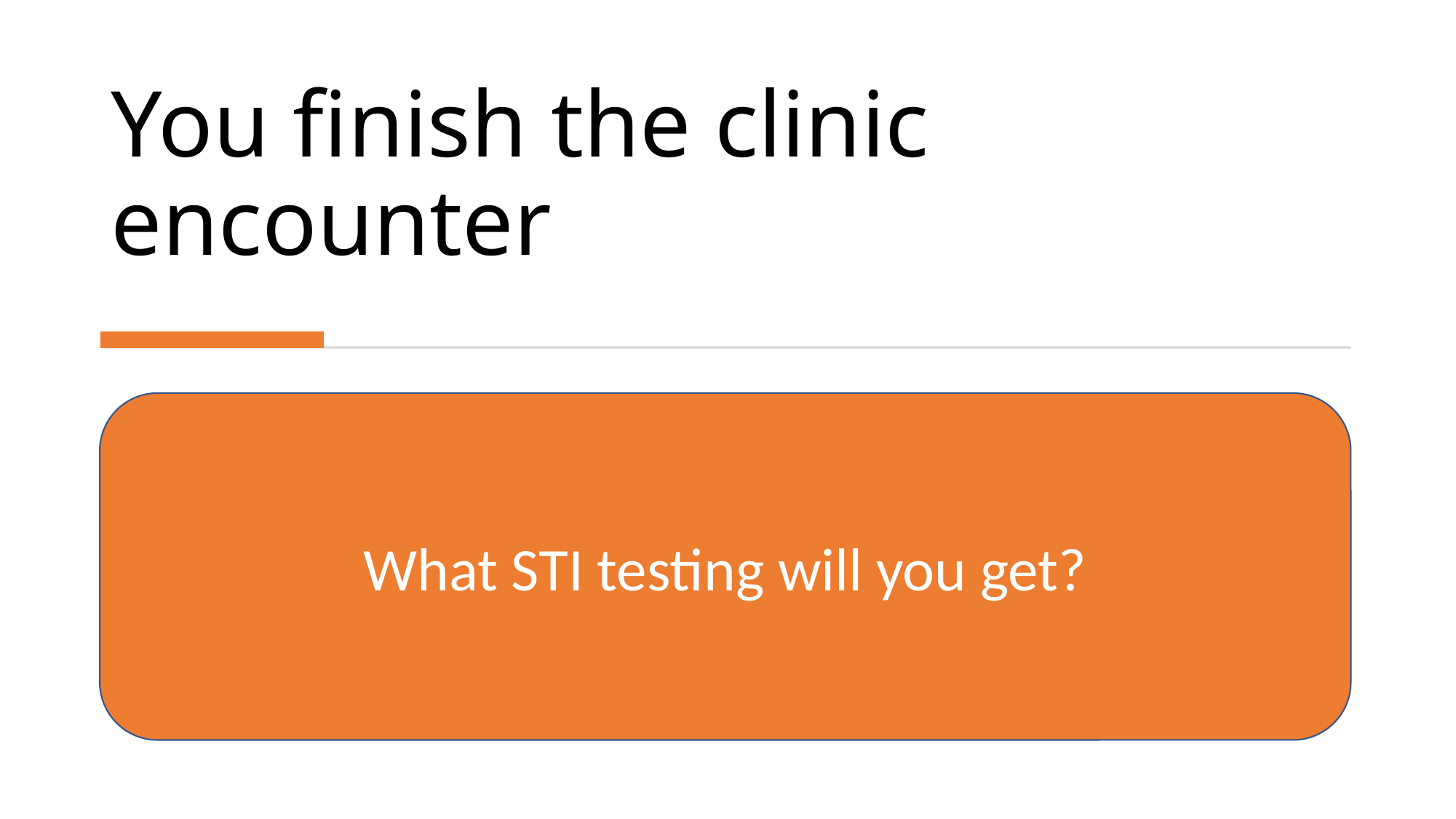

# You finish the clinic encounter
What STI testing will you get?
On your exam, you notice mild TTP on LLQ. Callie declines a rectal exam. Based on your assessment, you treat Callie for constipation and hemorrhoids, and you send an STI panel. You tell her to call back if her symptoms get worse and you schedule her for a visit in 1-2 weeks to discuss results and complete an annual physical.

## Slide 34
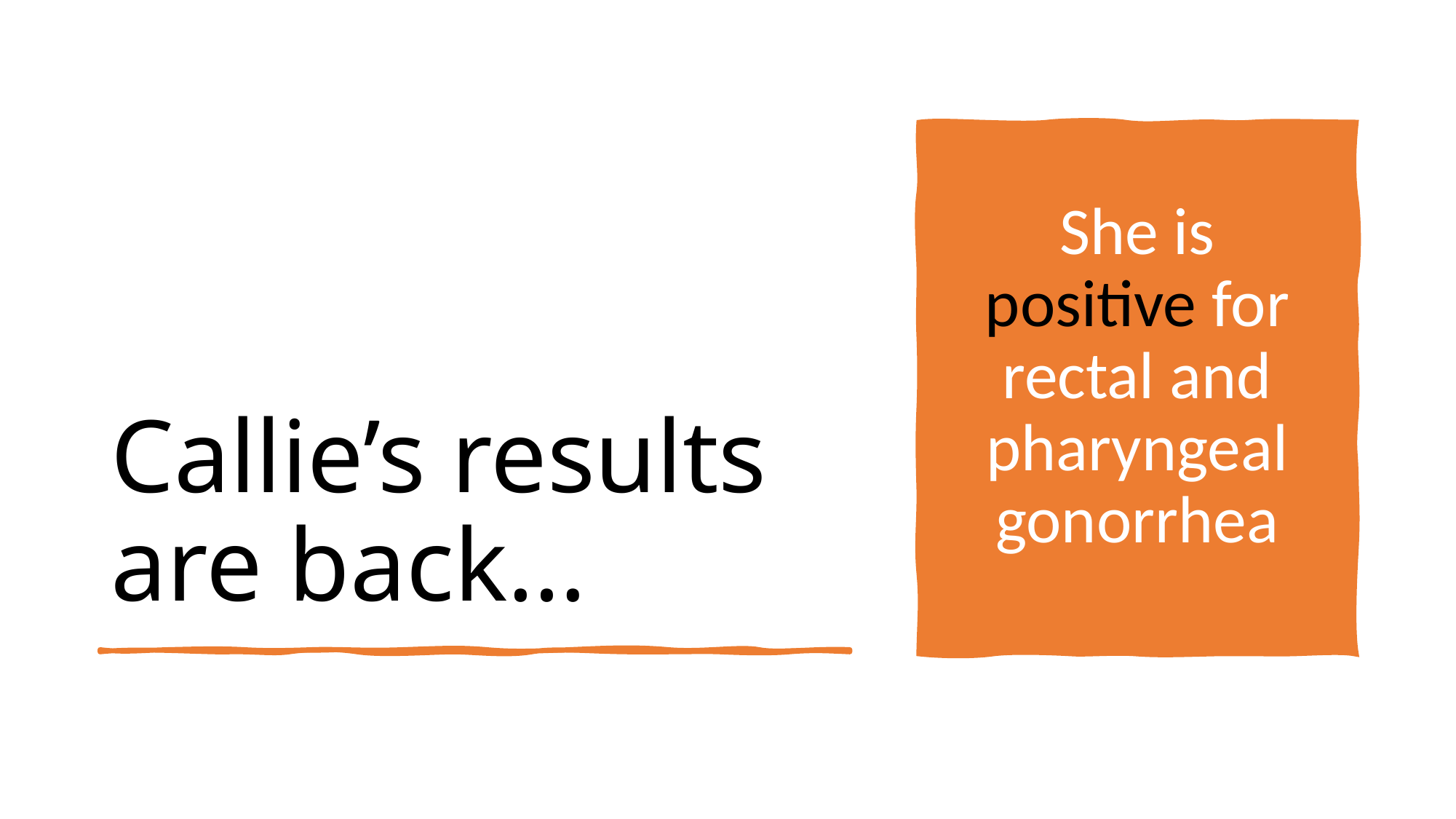

# Callie’s results are back…
She is positive for rectal and pharyngeal gonorrhea

## Slide 35
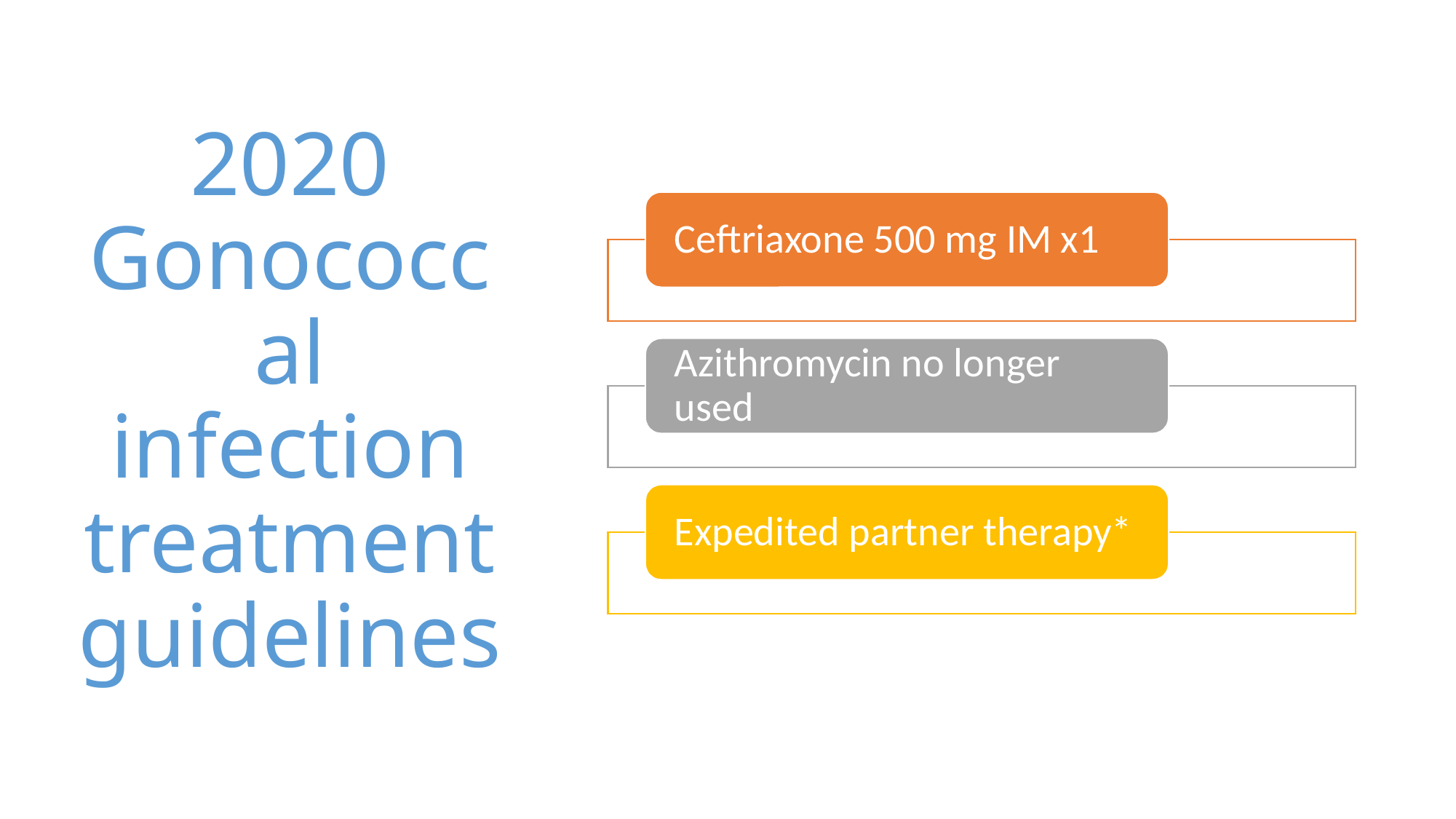

# 2020 Gonococcal infection treatment guidelines

## Slide 36
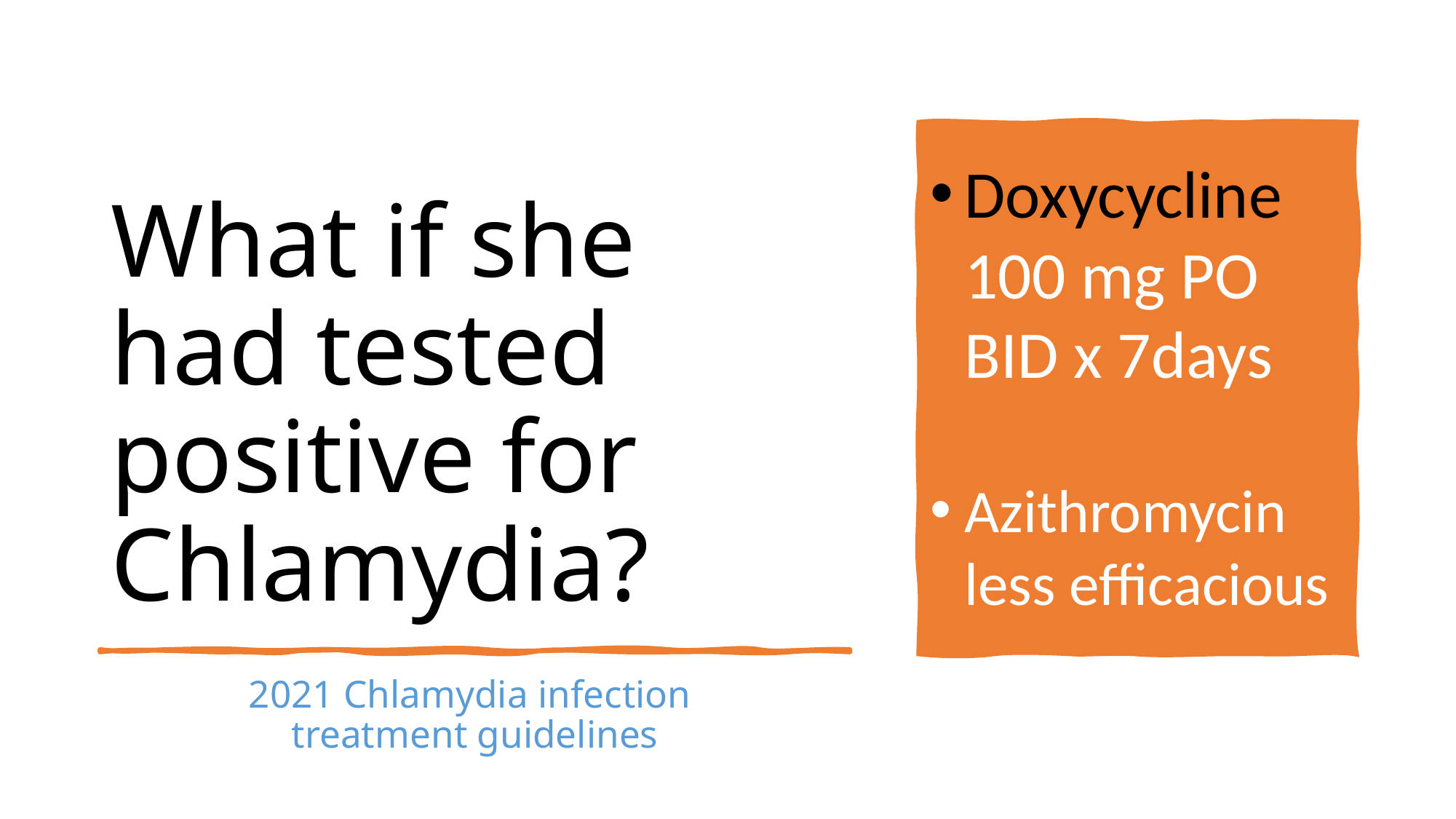

# What if she had tested positive for Chlamydia?
Doxycycline 100 mg PO BID x 7days
Azithromycin less efficacious
2021 Chlamydia infection
treatment guidelines

## Slide 37
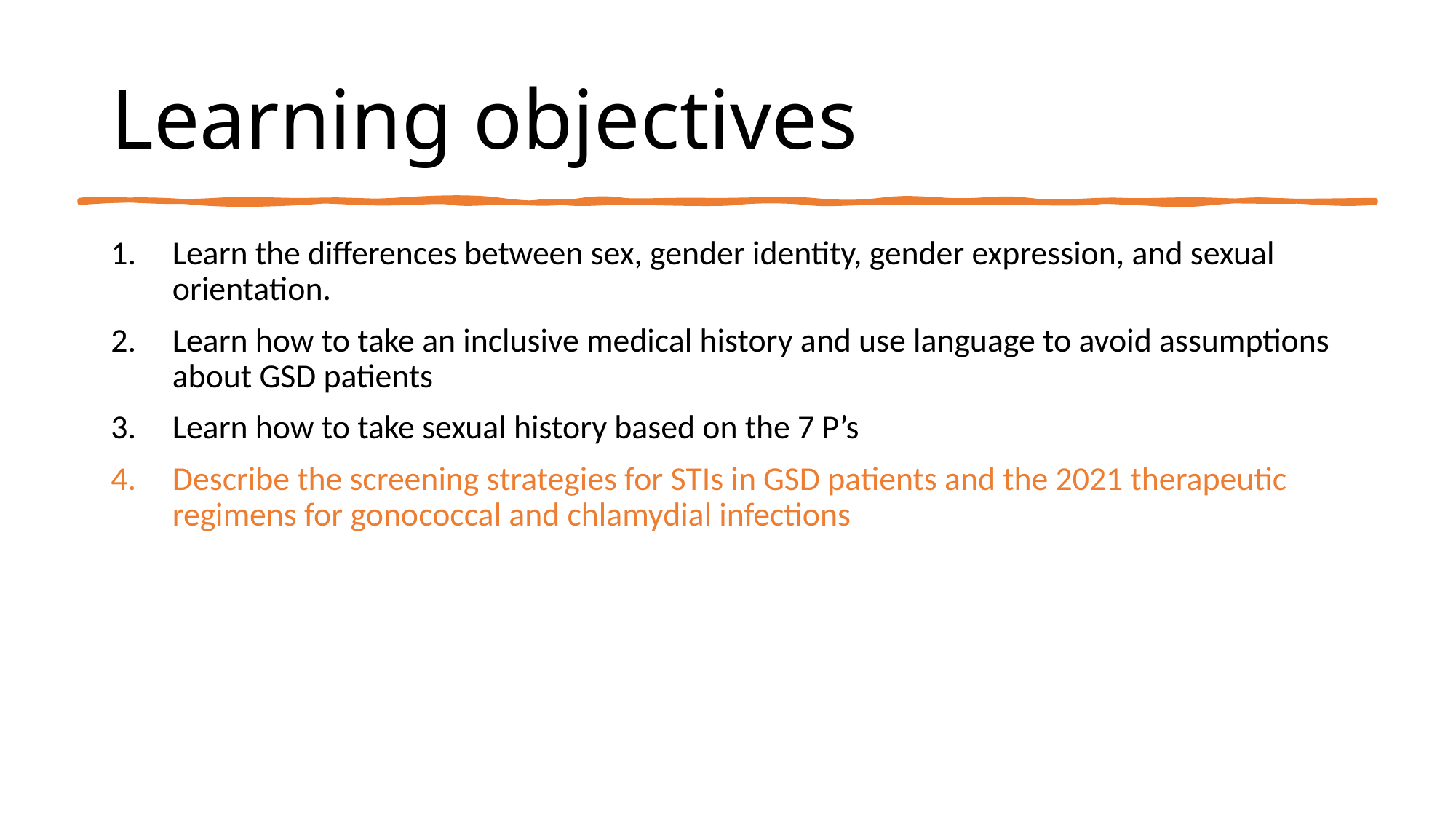

# Learning objectives
Learn the differences between sex, gender identity, gender expression, and sexual orientation.
Learn how to take an inclusive medical history and use language to avoid assumptions about GSD patients
Learn how to take sexual history based on the 7 P’s
Describe the screening strategies for STIs in GSD patients and the 2021 therapeutic regimens for gonococcal and chlamydial infections

## Slide 38
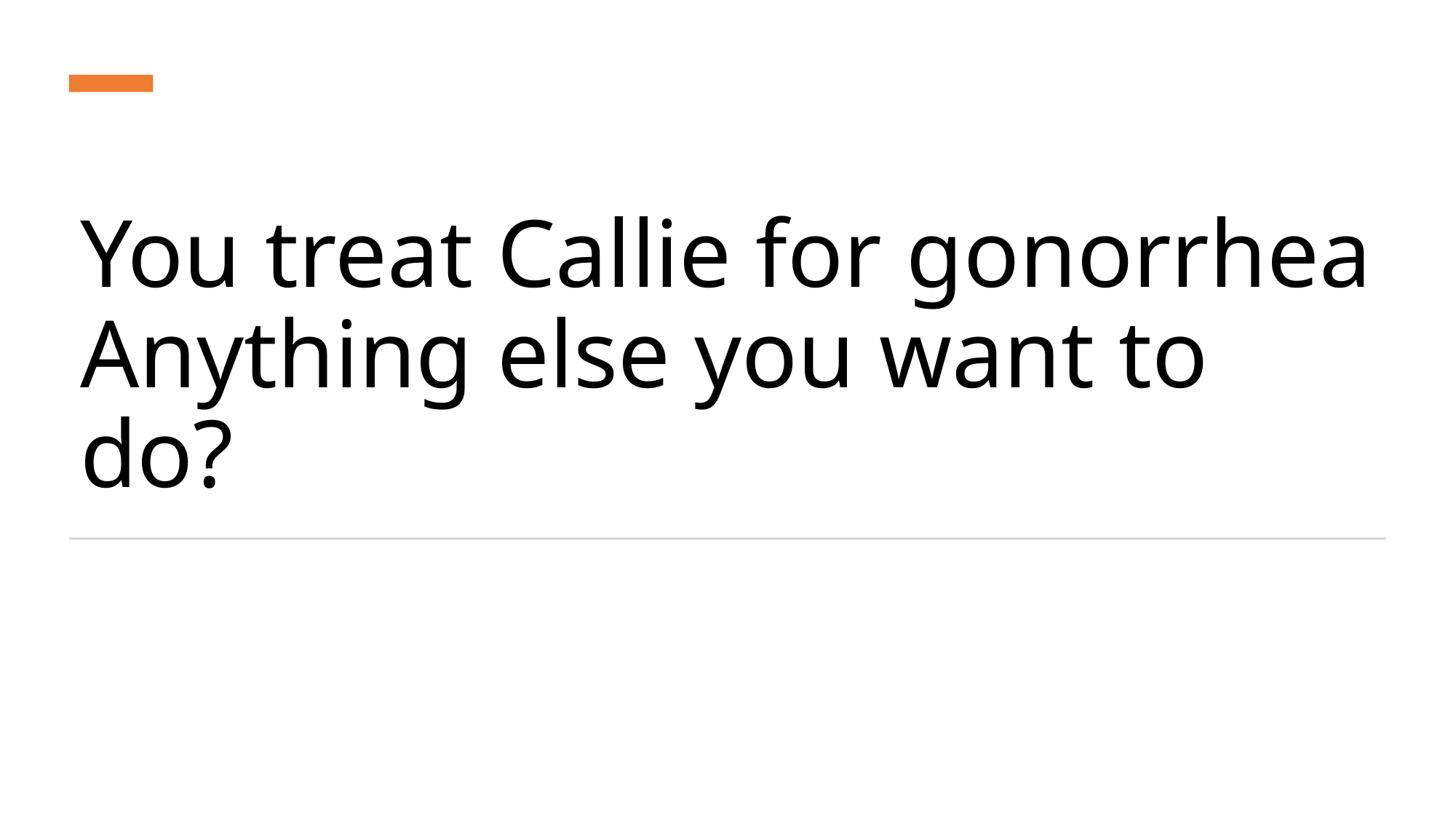

# You treat Callie for gonorrhea Anything else you want to do?

## Slide 39
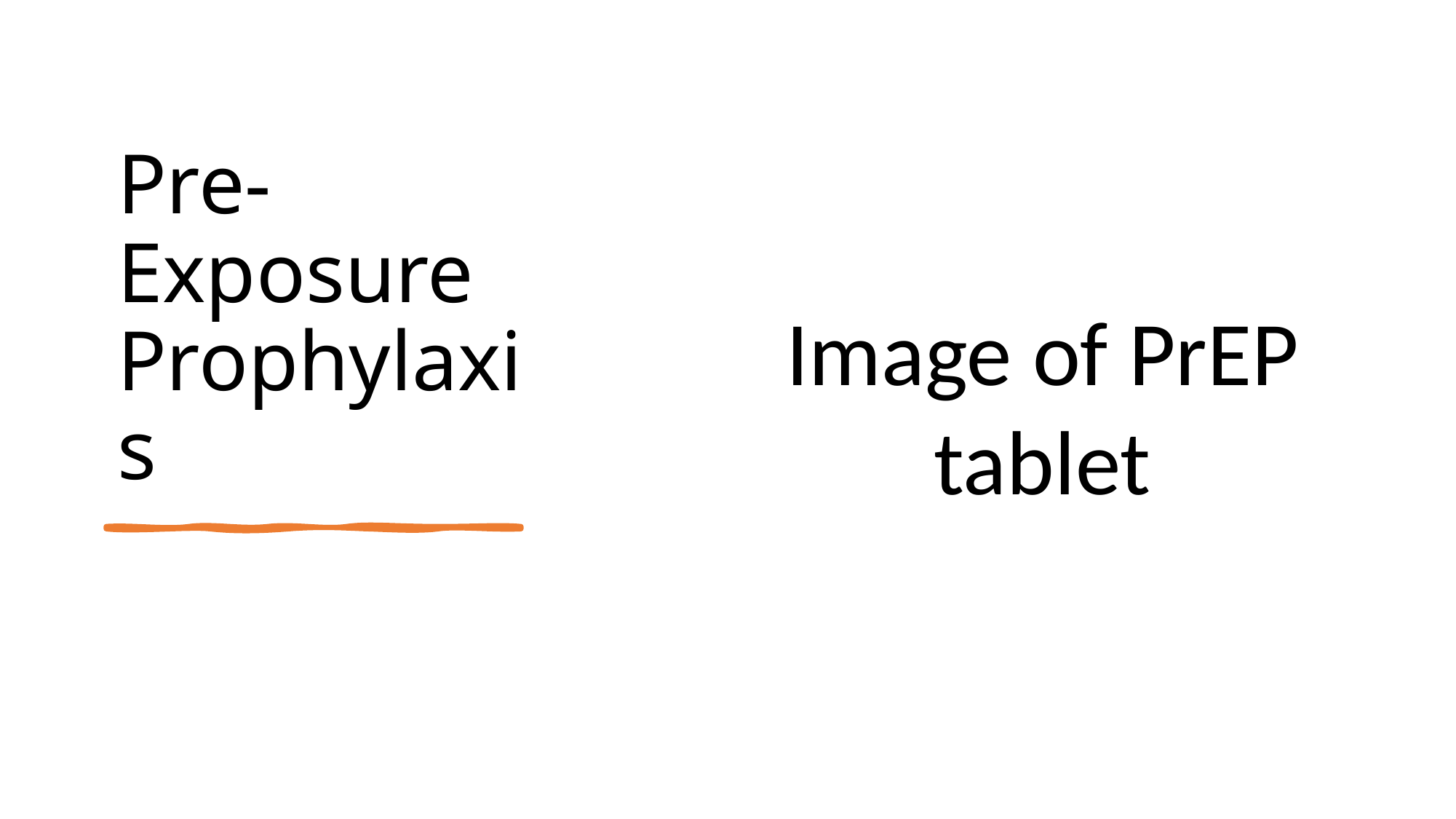

# Pre-Exposure Prophylaxis
Image of PrEP
tablet

## Slide 40
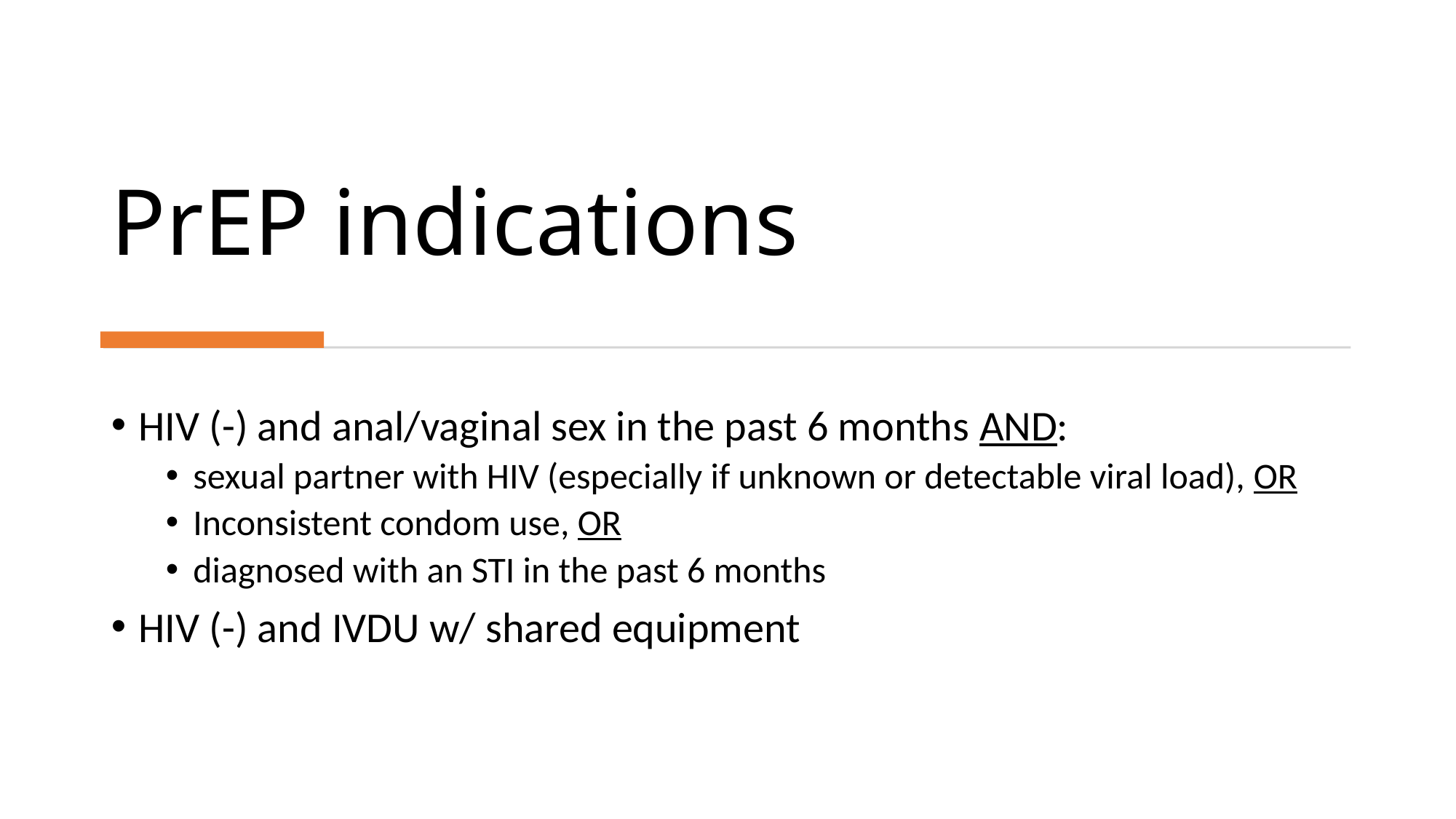

# PrEP indications
HIV (-) and anal/vaginal sex in the past 6 months AND:
sexual partner with HIV (especially if unknown or detectable viral load), OR
Inconsistent condom use, OR
diagnosed with an STI in the past 6 months
HIV (-) and IVDU w/ shared equipment

## Slide 41
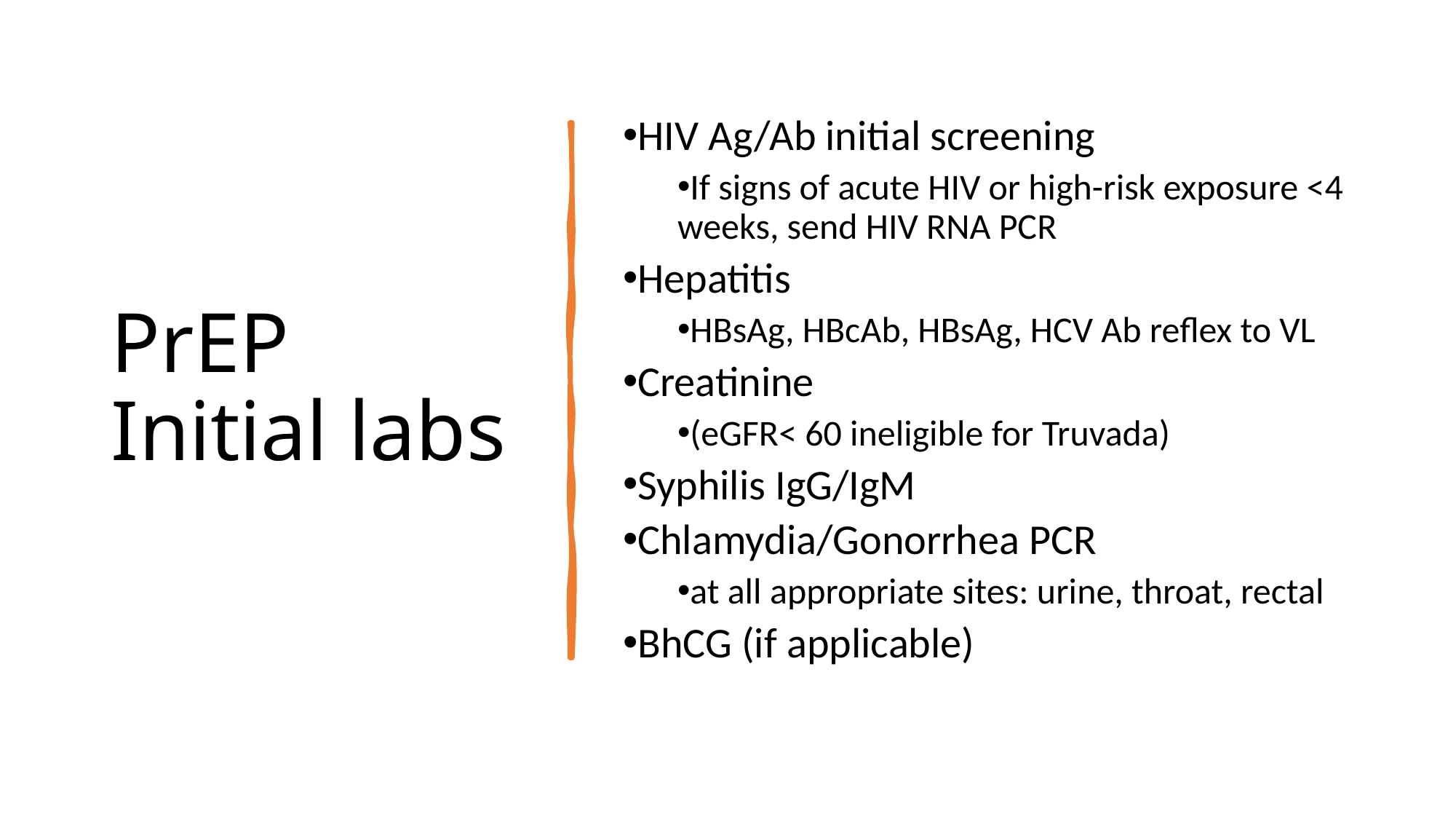

# PrEP Initial labs
HIV Ag/Ab initial screening
If signs of acute HIV or high-risk exposure <4 weeks, send HIV RNA PCR
Hepatitis
HBsAg, HBcAb, HBsAg, HCV Ab reflex to VL
Creatinine
(eGFR< 60 ineligible for Truvada)
Syphilis IgG/IgM
Chlamydia/Gonorrhea PCR
at all appropriate sites: urine, throat, rectal
BhCG (if applicable)

## Slide 42
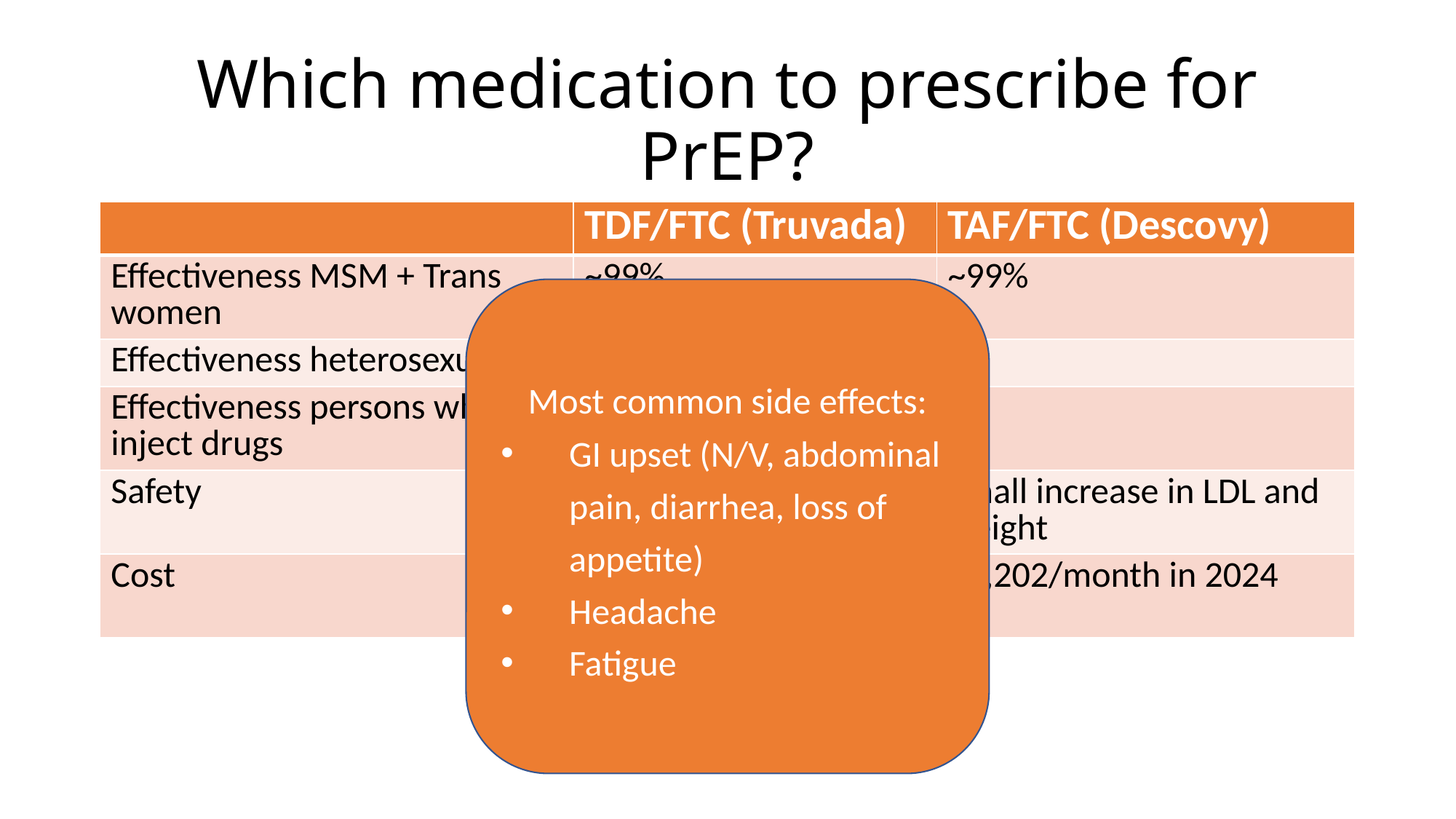

# Which medication to prescribe for PrEP?
| | TDF/FTC (Truvada) | TAF/FTC (Descovy) |
| --- | --- | --- |
| Effectiveness MSM + Trans women | ~99% | ~99% |
| Effectiveness heterosexuals | ~99% | ? |
| Effectiveness persons who inject drugs | 74-84% | ? |
| Safety | Small decrease eGFR and BMD | Small increase in LDL and weight |
| Cost | Covered by insurance, low cost | $2,202/month in 2024 |
Most common side effects:
GI upset (N/V, abdominal pain, diarrhea, loss of appetite)
Headache
Fatigue

## Slide 43
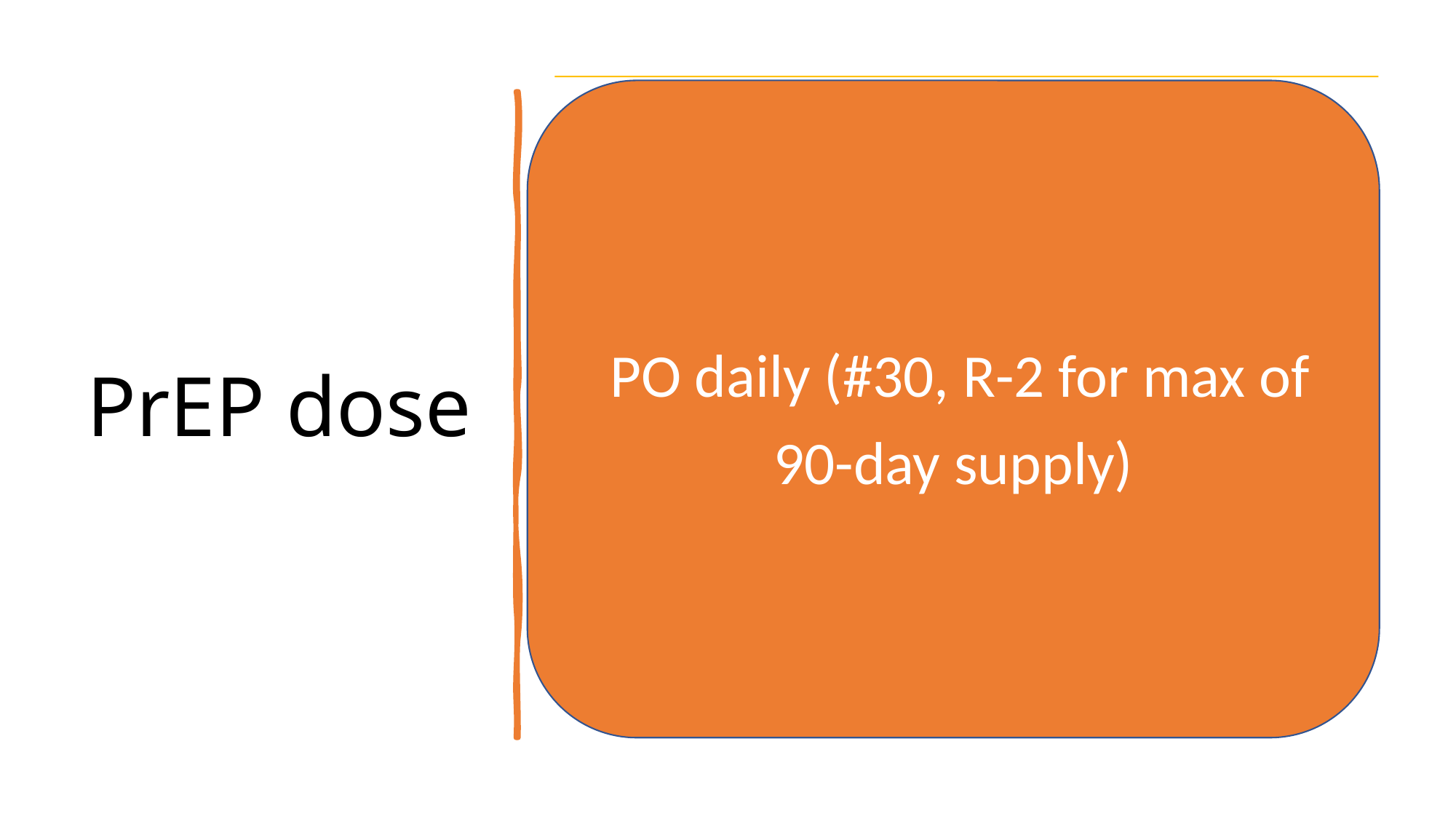

# PrEP dose
 PO daily (#30, R-2 for max of 90-day supply)

## Slide 44
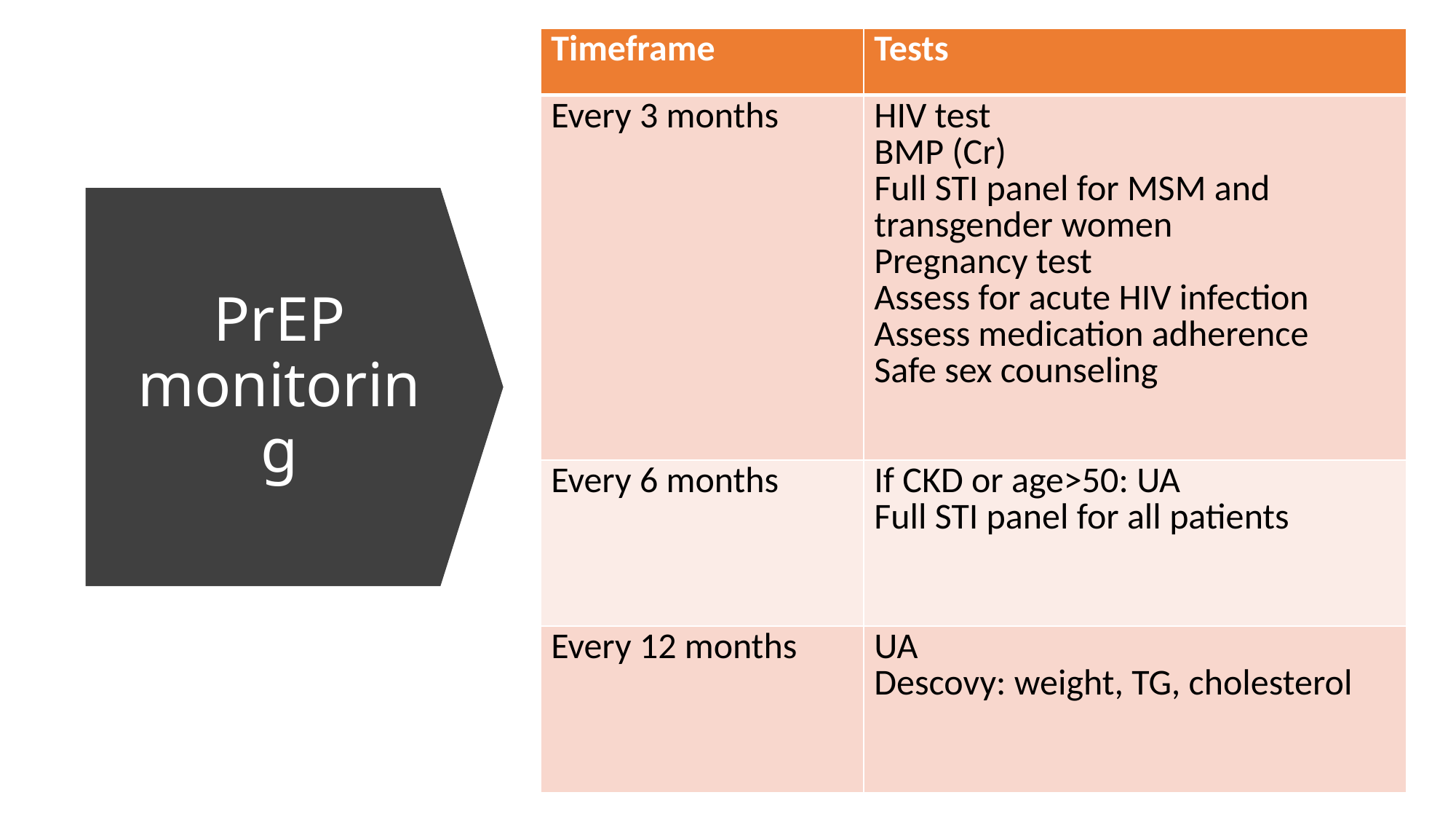

| Timeframe | Tests |
| --- | --- |
| Every 3 months | HIV test BMP (Cr) Full STI panel for MSM and transgender women Pregnancy test Assess for acute HIV infection Assess medication adherence Safe sex counseling |
| Every 6 months | If CKD or age>50: UA Full STI panel for all patients |
| Every 12 months | UA Descovy: weight, TG, cholesterol |
# PrEP monitoring

## Slide 45
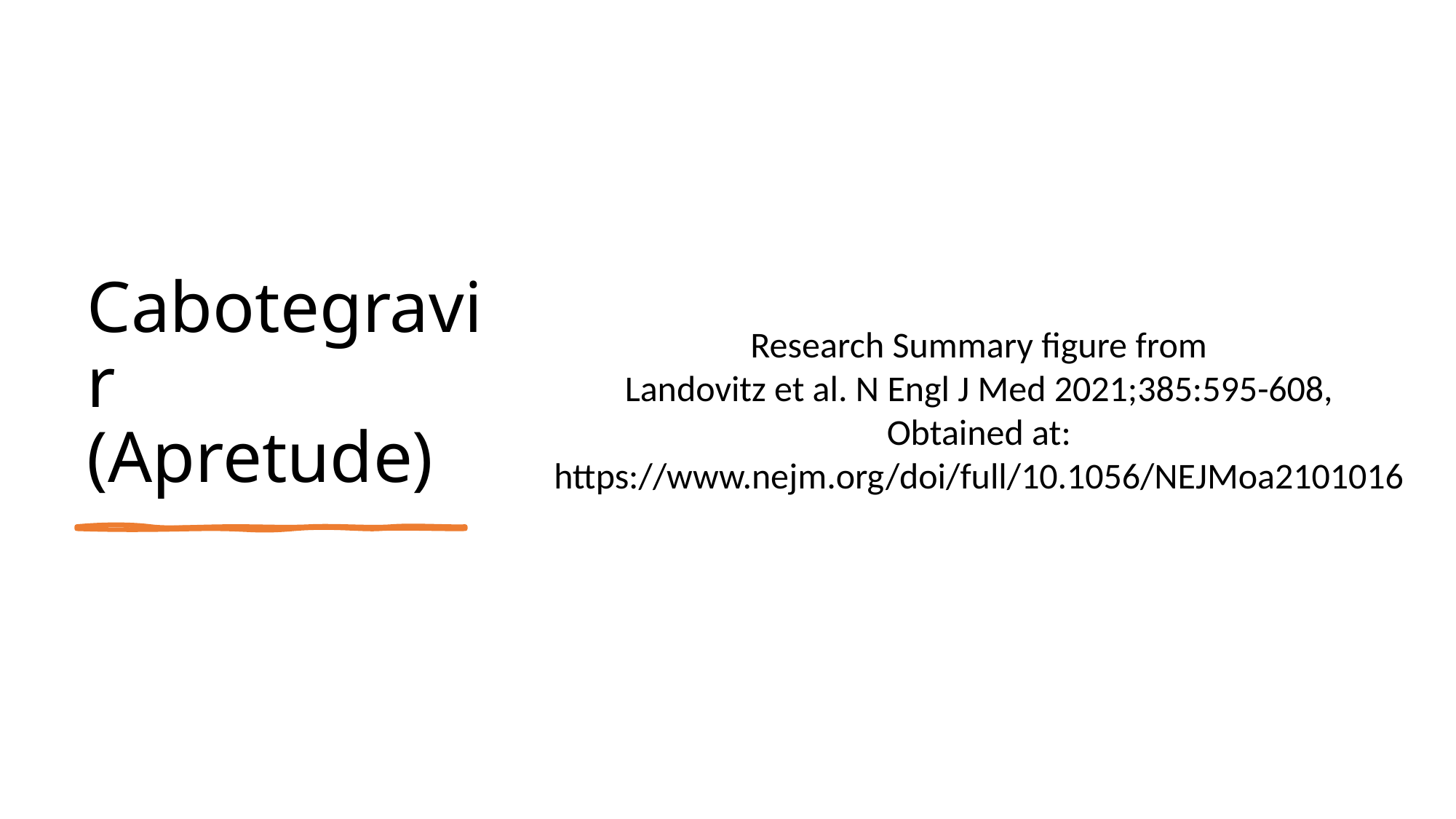

# Cabotegravir (Apretude)
Research Summary figure from
Landovitz et al. N Engl J Med 2021;385:595-608,
Obtained at: https://www.nejm.org/doi/full/10.1056/NEJMoa2101016

## Slide 46
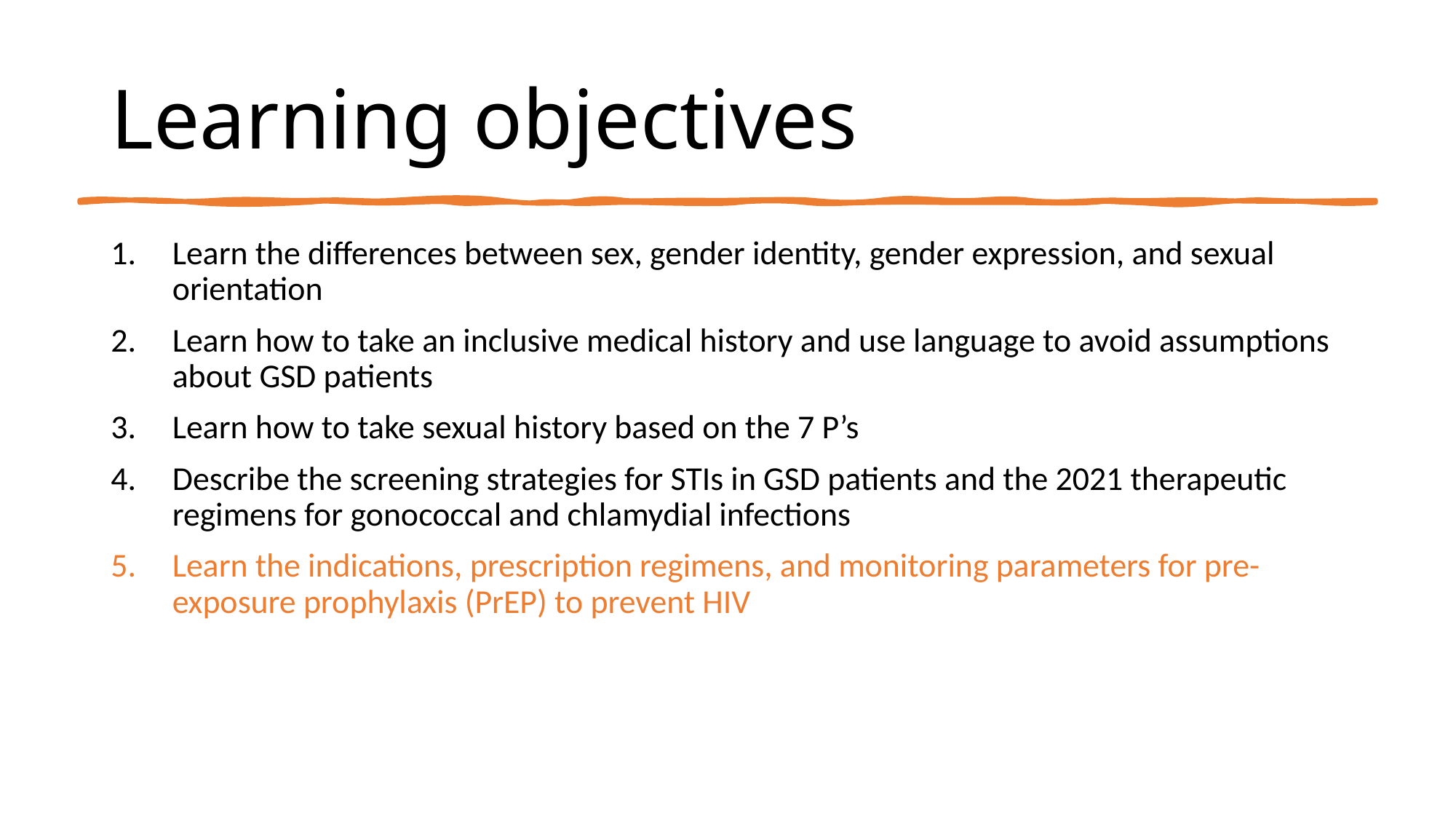

# Learning objectives
Learn the differences between sex, gender identity, gender expression, and sexual orientation
Learn how to take an inclusive medical history and use language to avoid assumptions about GSD patients
Learn how to take sexual history based on the 7 P’s
Describe the screening strategies for STIs in GSD patients and the 2021 therapeutic regimens for gonococcal and chlamydial infections
Learn the indications, prescription regimens, and monitoring parameters for pre-exposure prophylaxis (PrEP) to prevent HIV

## Slide 47
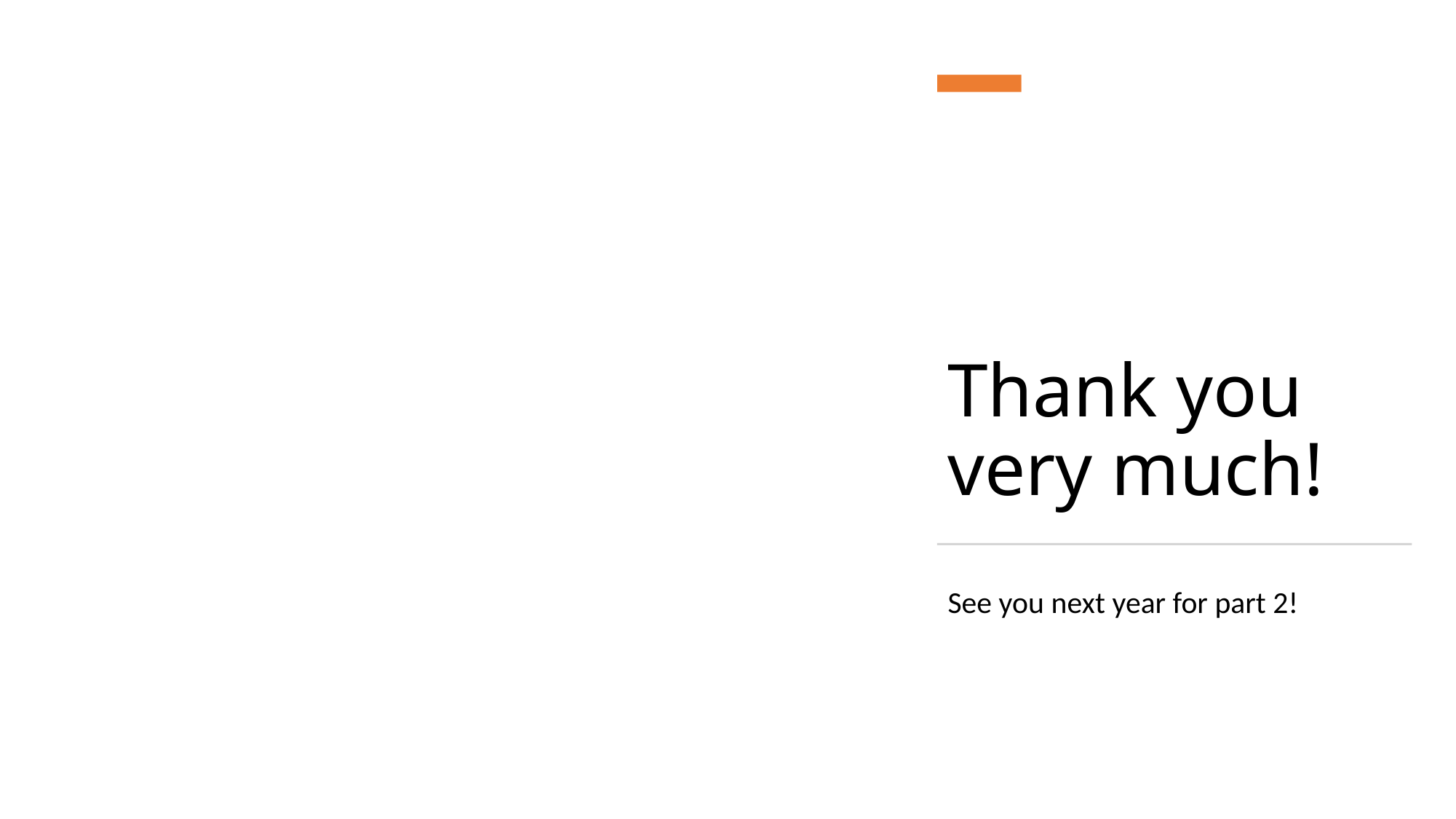

# Thank you very much!
See you next year for part 2!
