# Supplementary material for: A Systematic and Practical Framework on Gender and Sexual Diverse (GSD) Health for Internal Medicine Residents
Source: MedEdPORTAL. 2025 Jun 17;21:11535. doi: 10.15766/mep_2374-8265.11535 (PMC12170925; doi:10.15766/mep_2374-8265.11535)
Supplement: Supplementary file 1 — GSD Health Handout.pptxGAHT Handout.pptxFacilitator Guide.docxGSD Health - Part 1.pptxGSD Health - Transgender Health.pptxGSD Health Survey.docxTGD Health Survey.docx [file mep_2374-8265.11535-s001.zip › E. GSD Health - Transgender Health.pptx]

## Slide 1
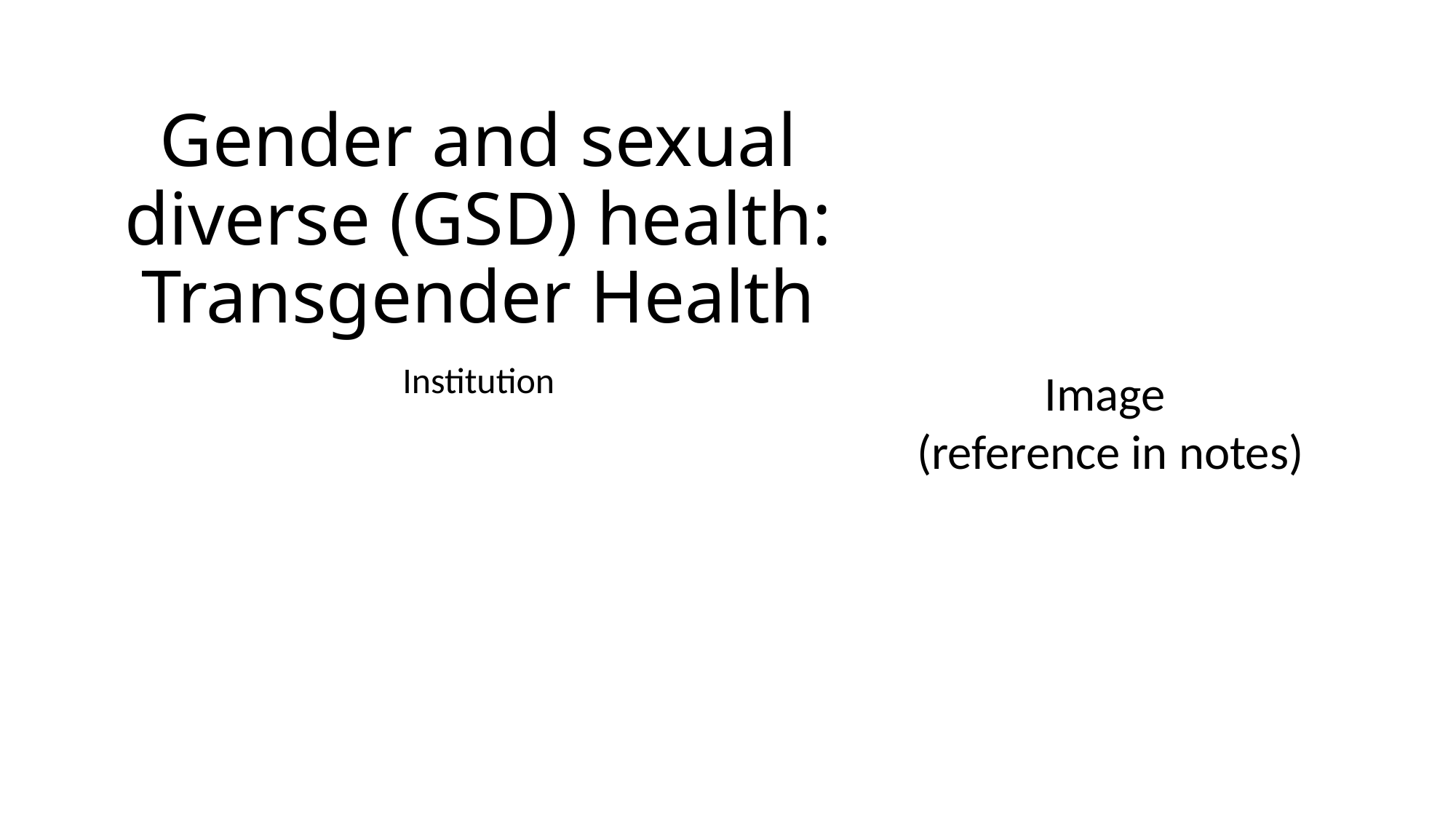

# Gender and sexual diverse (GSD) health: Transgender Health
Institution
Image
(reference in notes)

## Slide 2
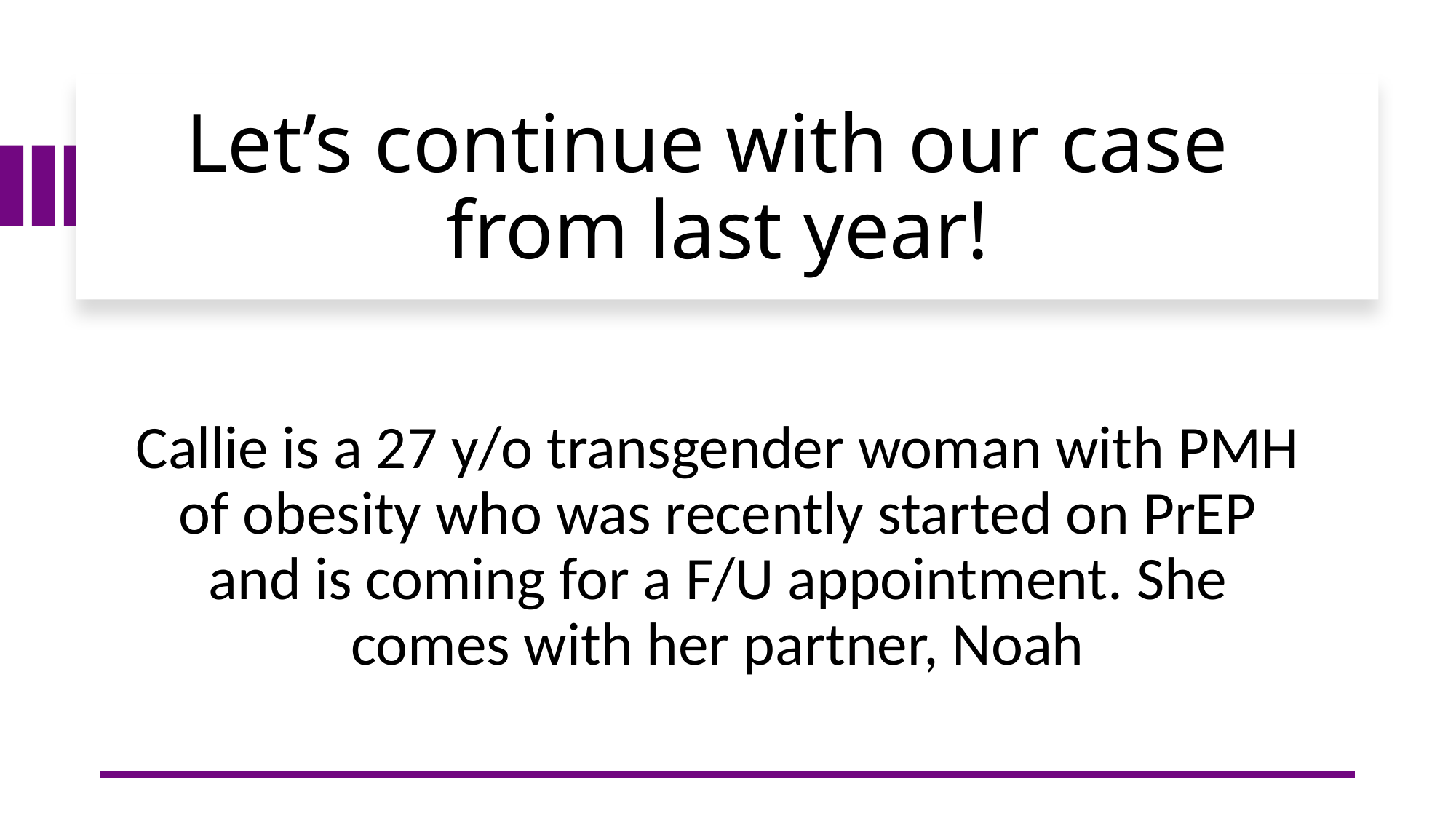

# Let’s continue with our case from last year!
Callie is a 27 y/o transgender woman with PMH of obesity who was recently started on PrEP and is coming for a F/U appointment. She comes with her partner, Noah

## Slide 3
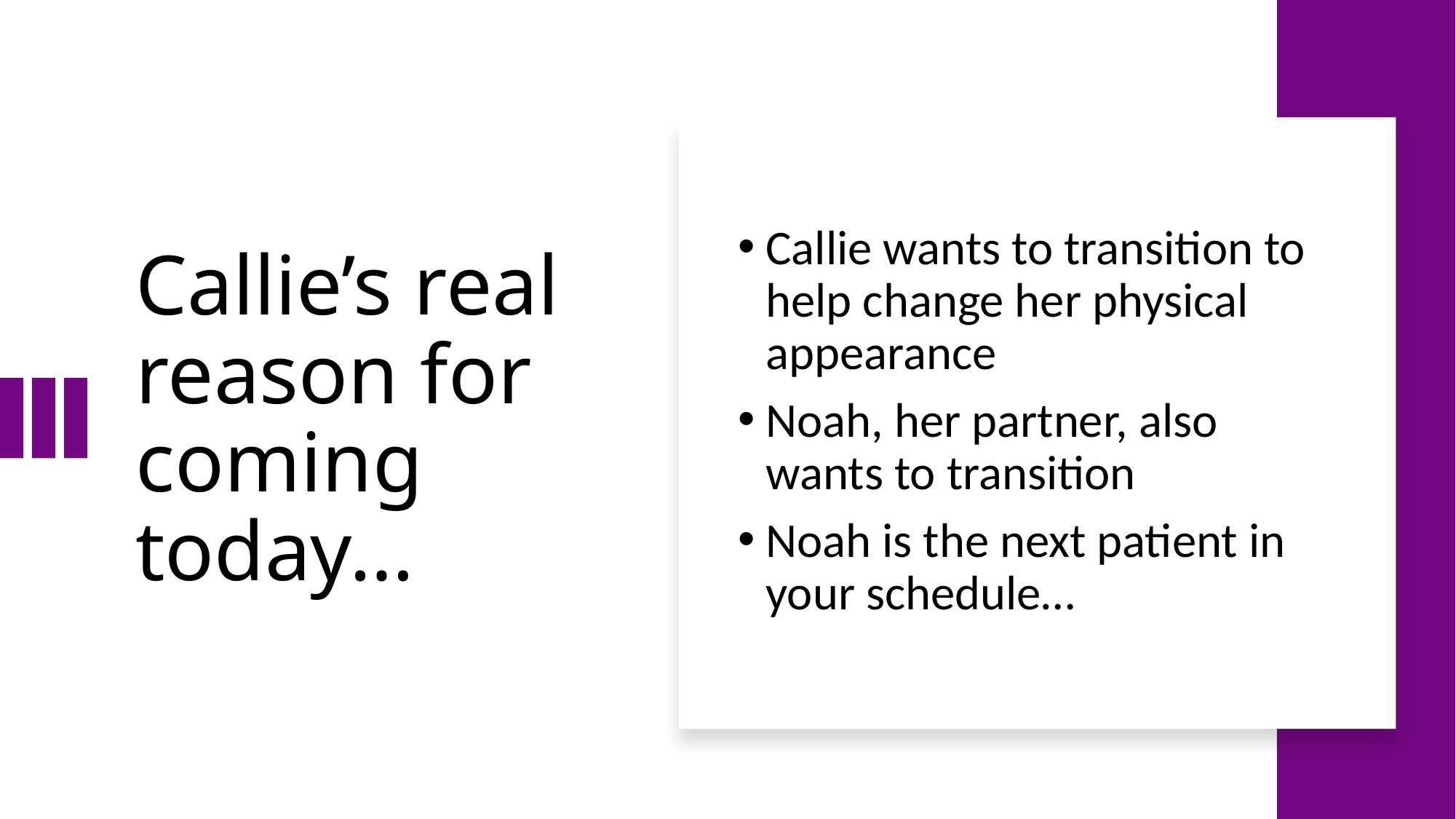

# Callie’s real reason for coming today…
Callie wants to transition to help change her physical appearance
Noah, her partner, also wants to transition
Noah is the next patient in your schedule…

## Slide 4
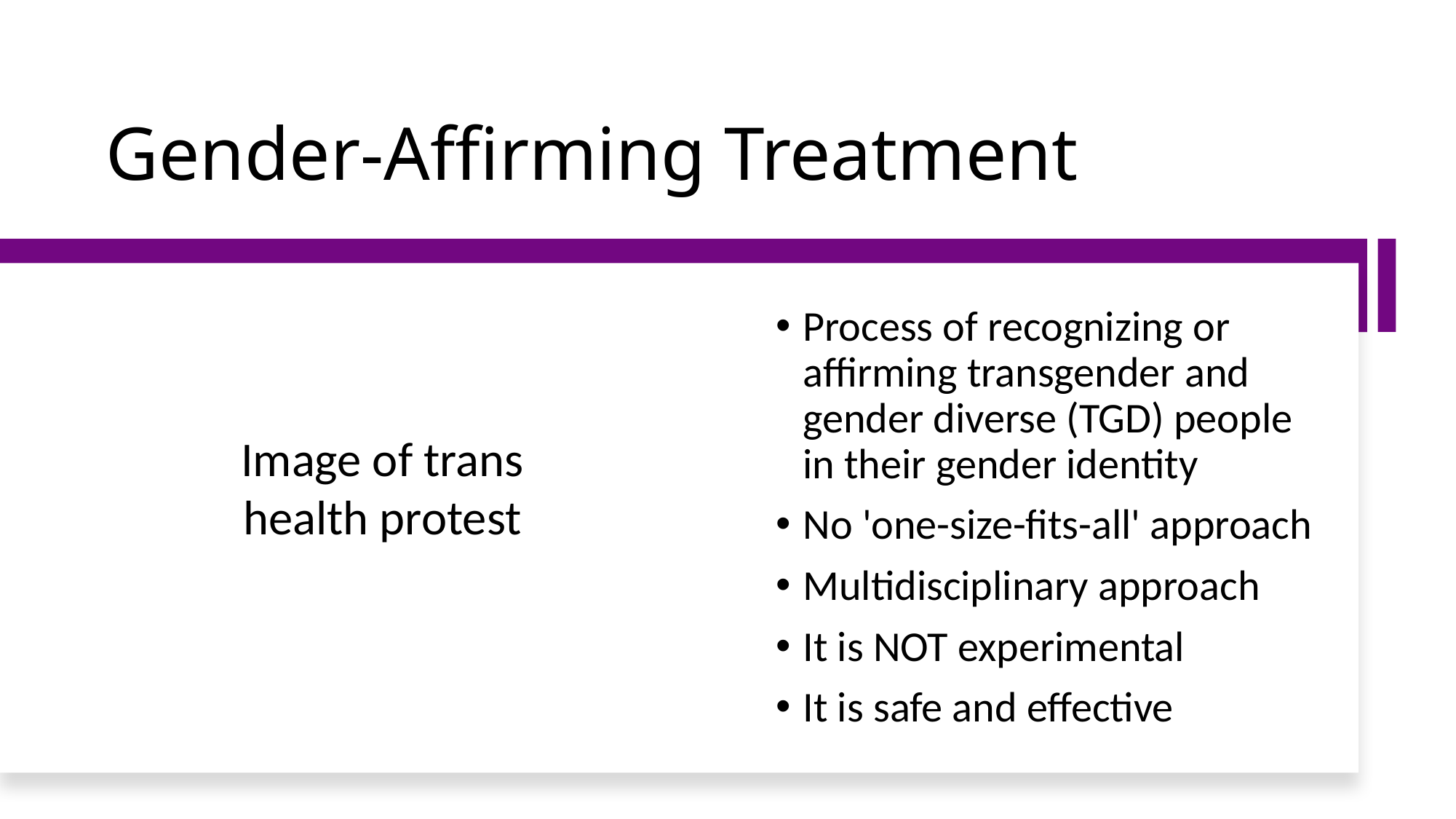

# Gender-Affirming Treatment
Process of recognizing or affirming transgender and gender diverse (TGD) people in their gender identity
No 'one-size-fits-all' approach
Multidisciplinary approach
It is NOT experimental
It is safe and effective
Image of trans
health protest

## Slide 5
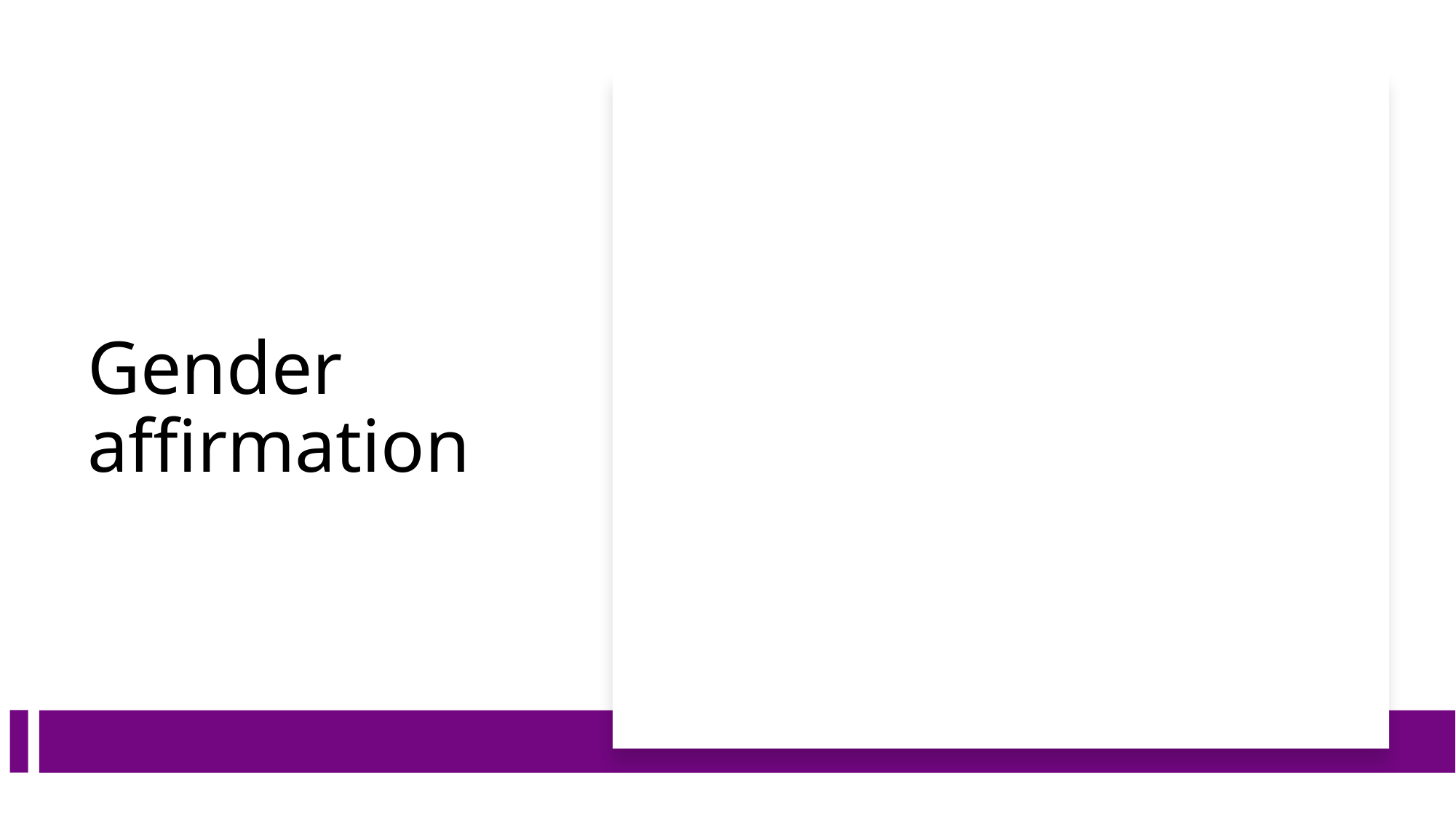

# Gender affirmation

## Slide 6
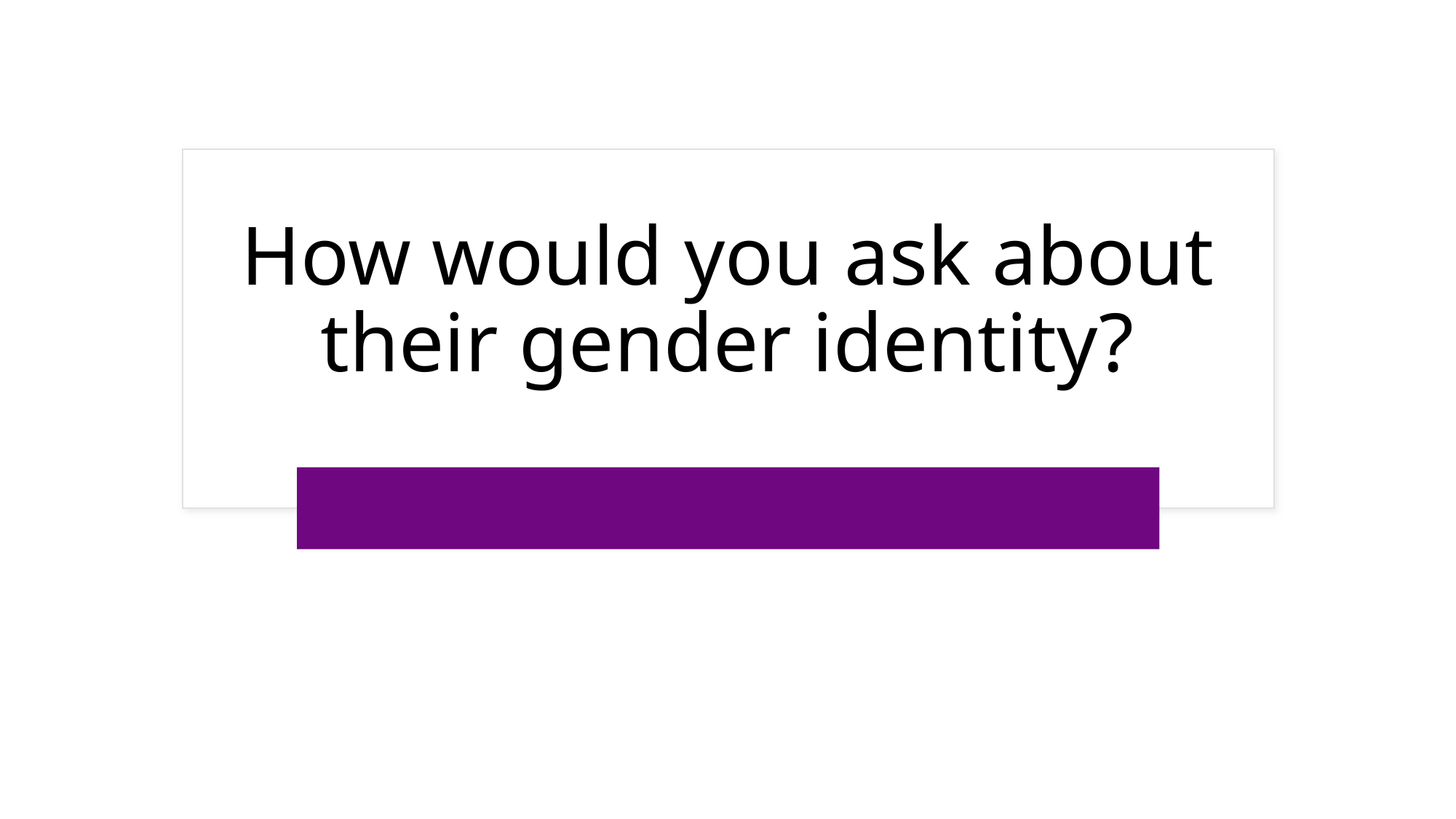

# How would you ask about their gender identity?

## Slide 7
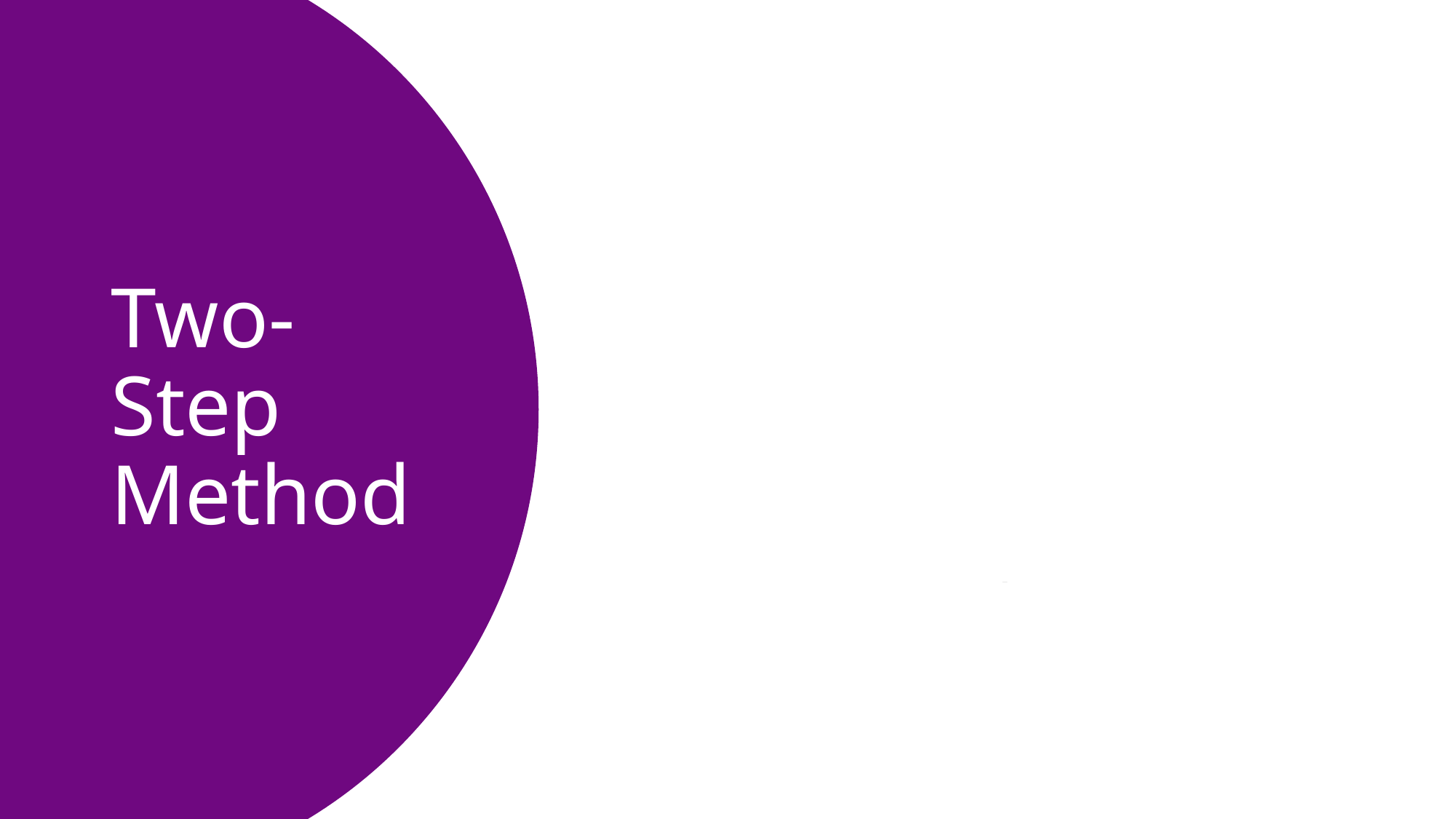

# Two-Step Method
Male (AMAB), Female (AFAB)
Male/man, female/woman, transgender, other

## Slide 8
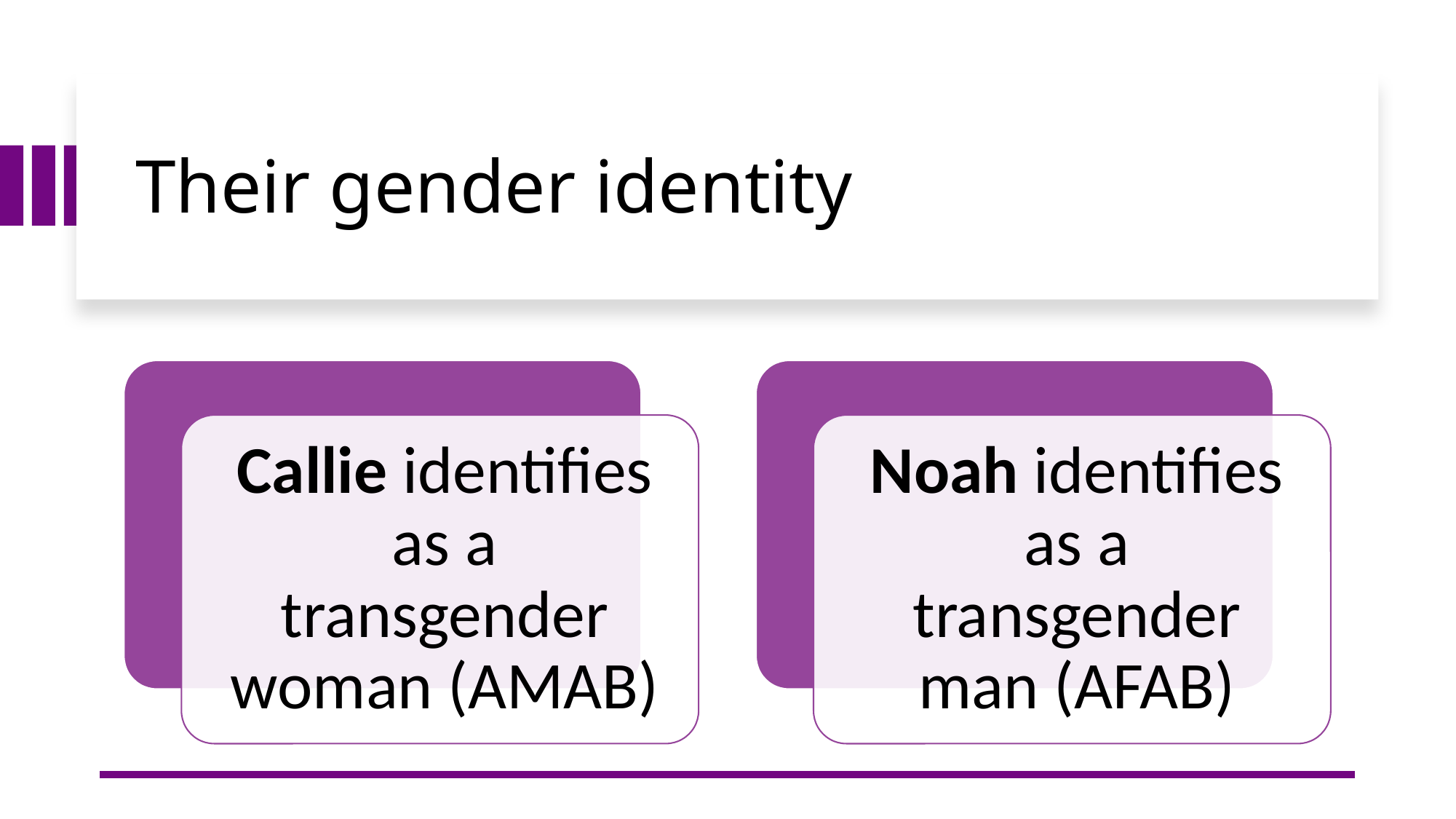

# Their gender identity

## Slide 9
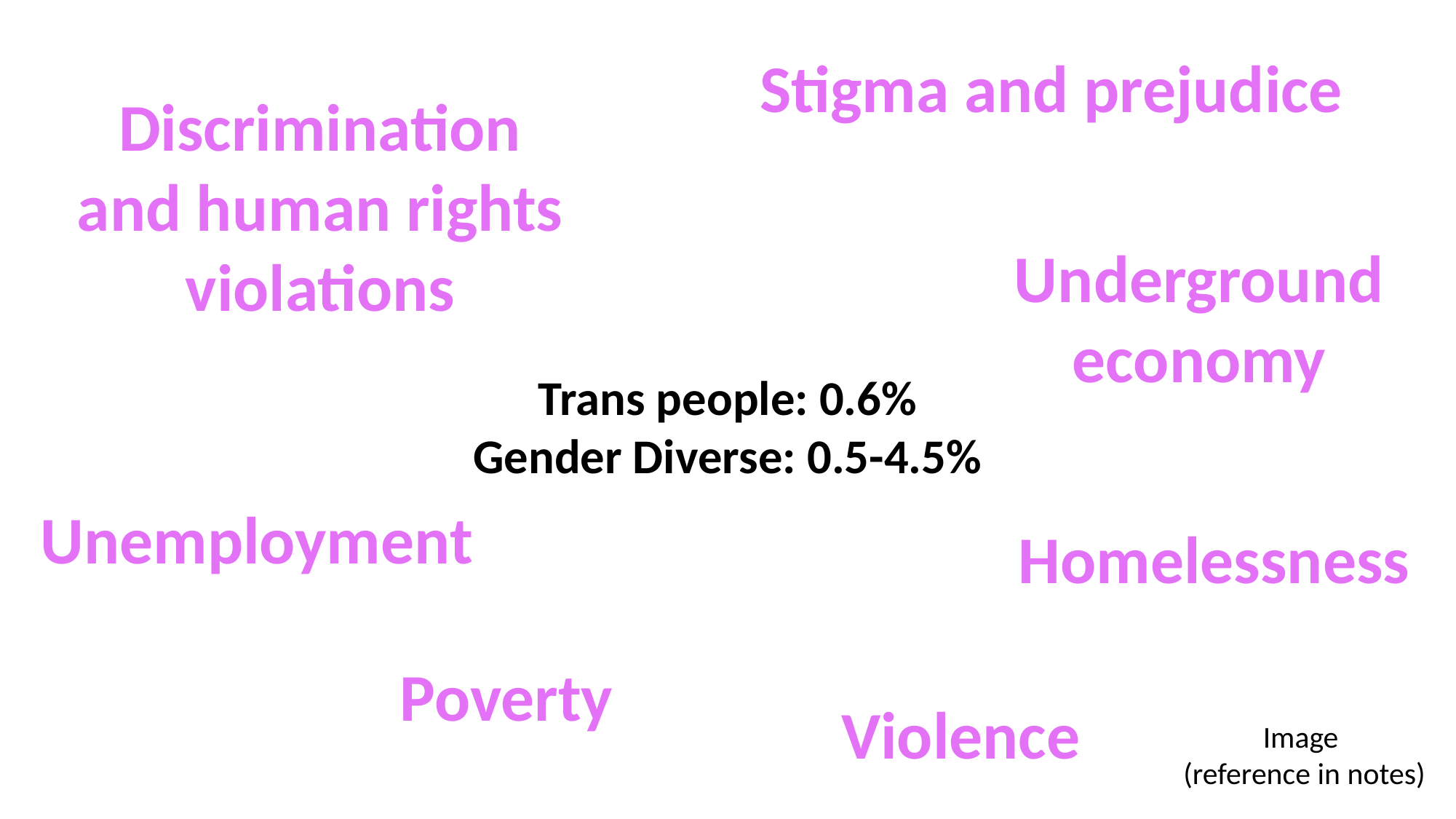

Stigma and prejudice
Discrimination and human rights violations
Underground
economy
Trans people: 0.6%
Gender Diverse: 0.5-4.5%
Unemployment
Homelessness
Poverty
Violence
Image
(reference in notes)

## Slide 10
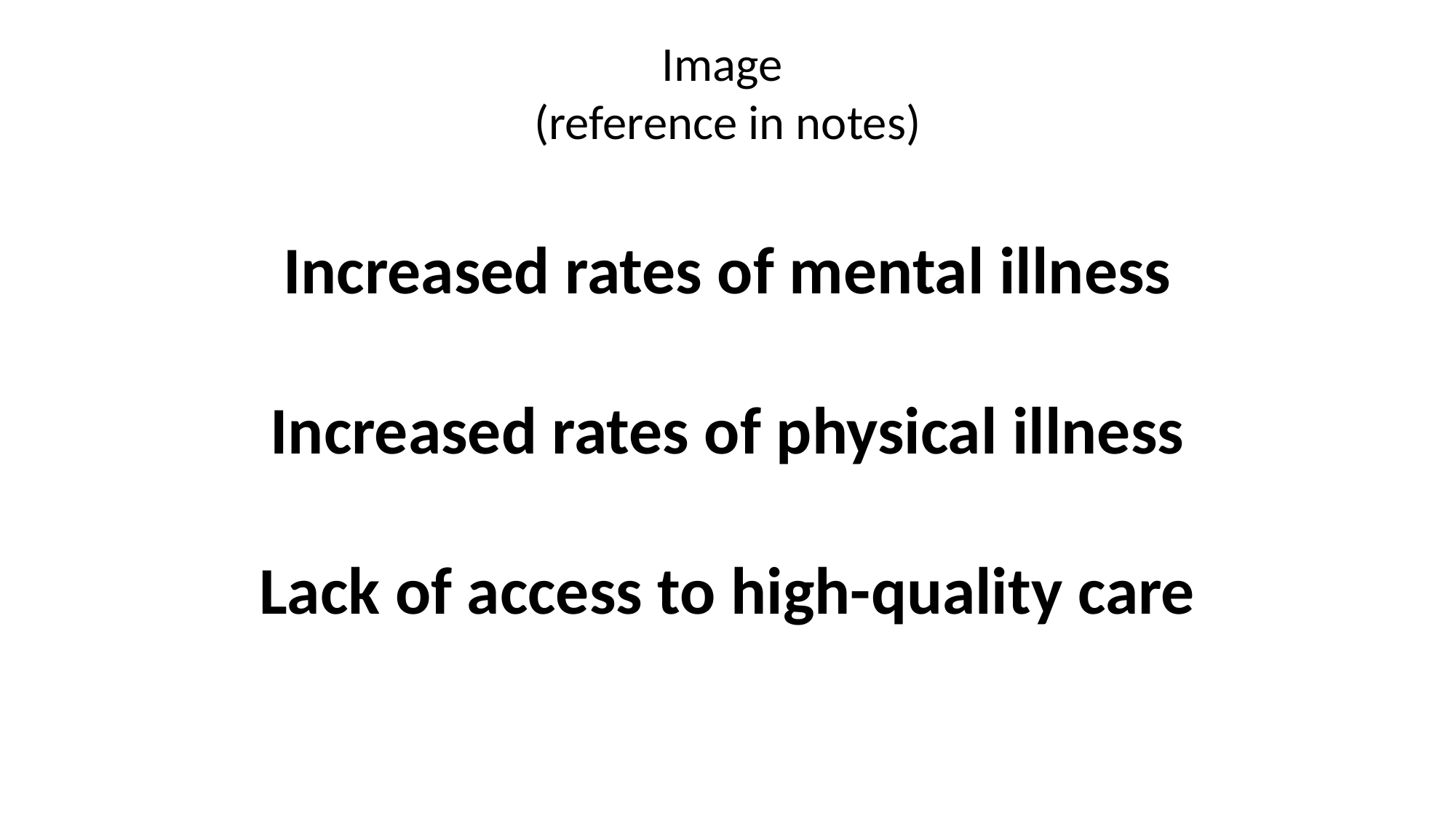

Image
(reference in notes)
Increased rates of mental illness
Increased rates of physical illness
Lack of access to high-quality care

## Slide 11
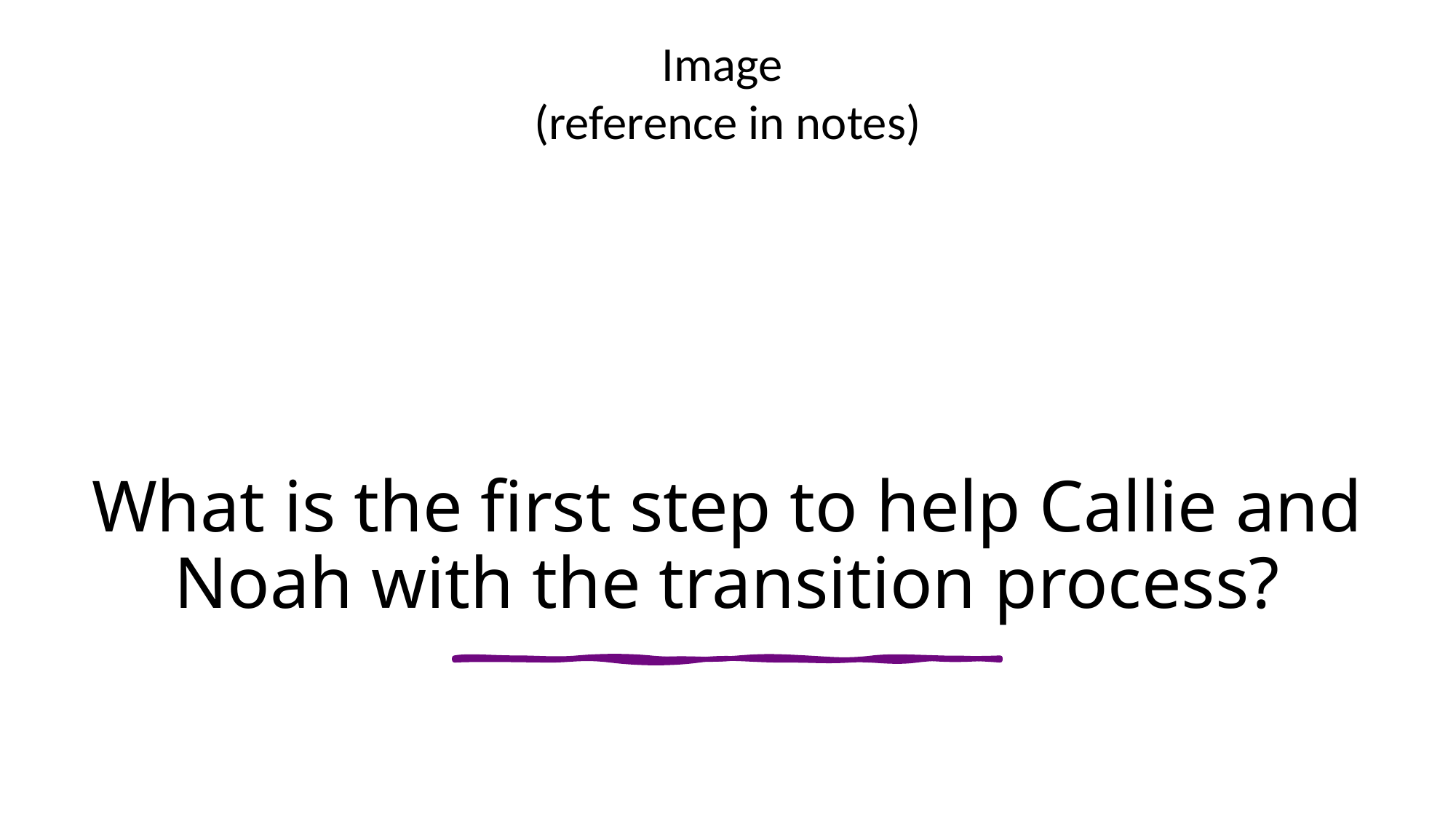

Image
(reference in notes)
# What is the first step to help Callie and Noah with the transition process?

## Slide 12
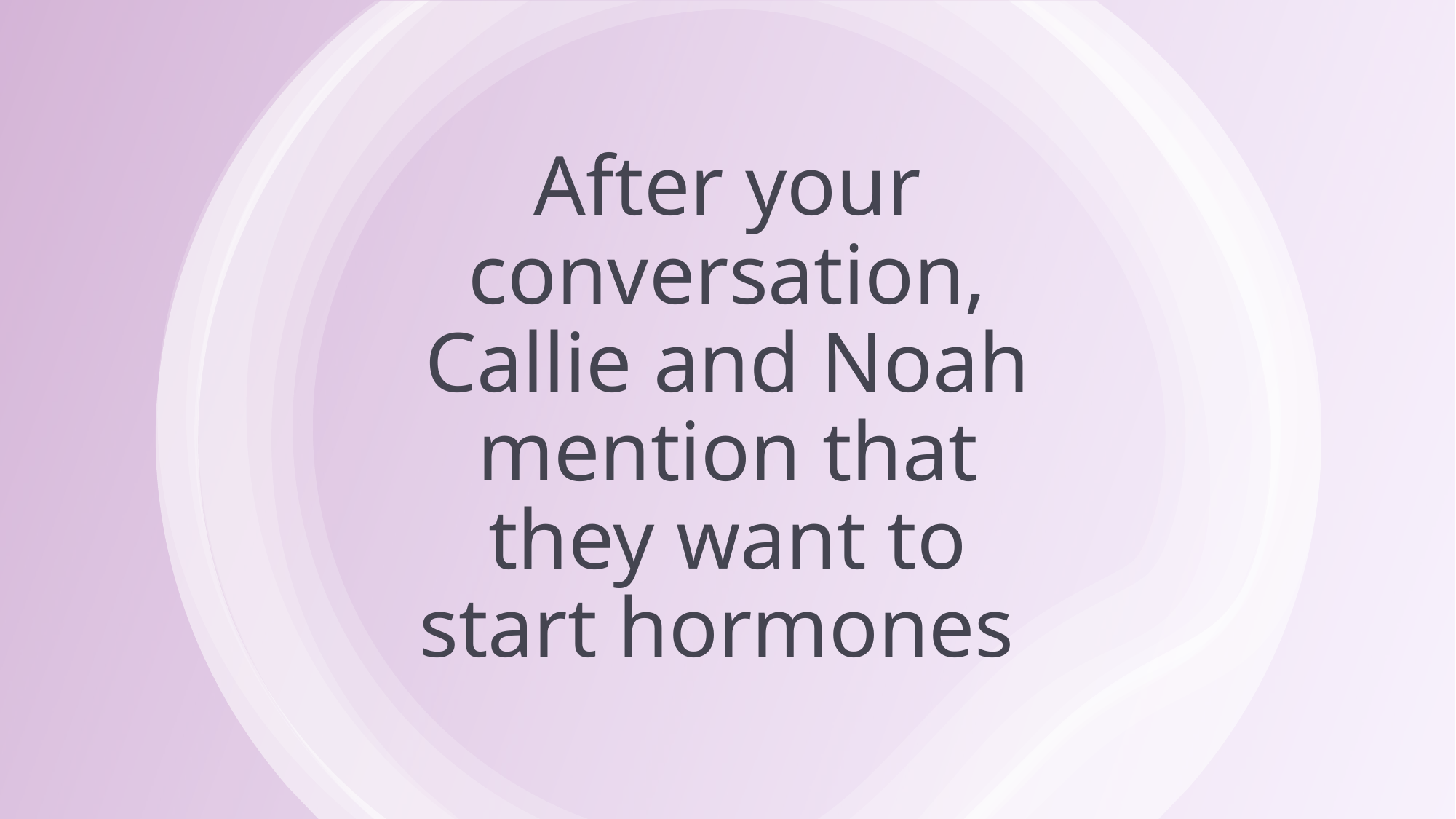

# After your conversation, Callie and Noah mention that they want to start hormones

## Slide 13
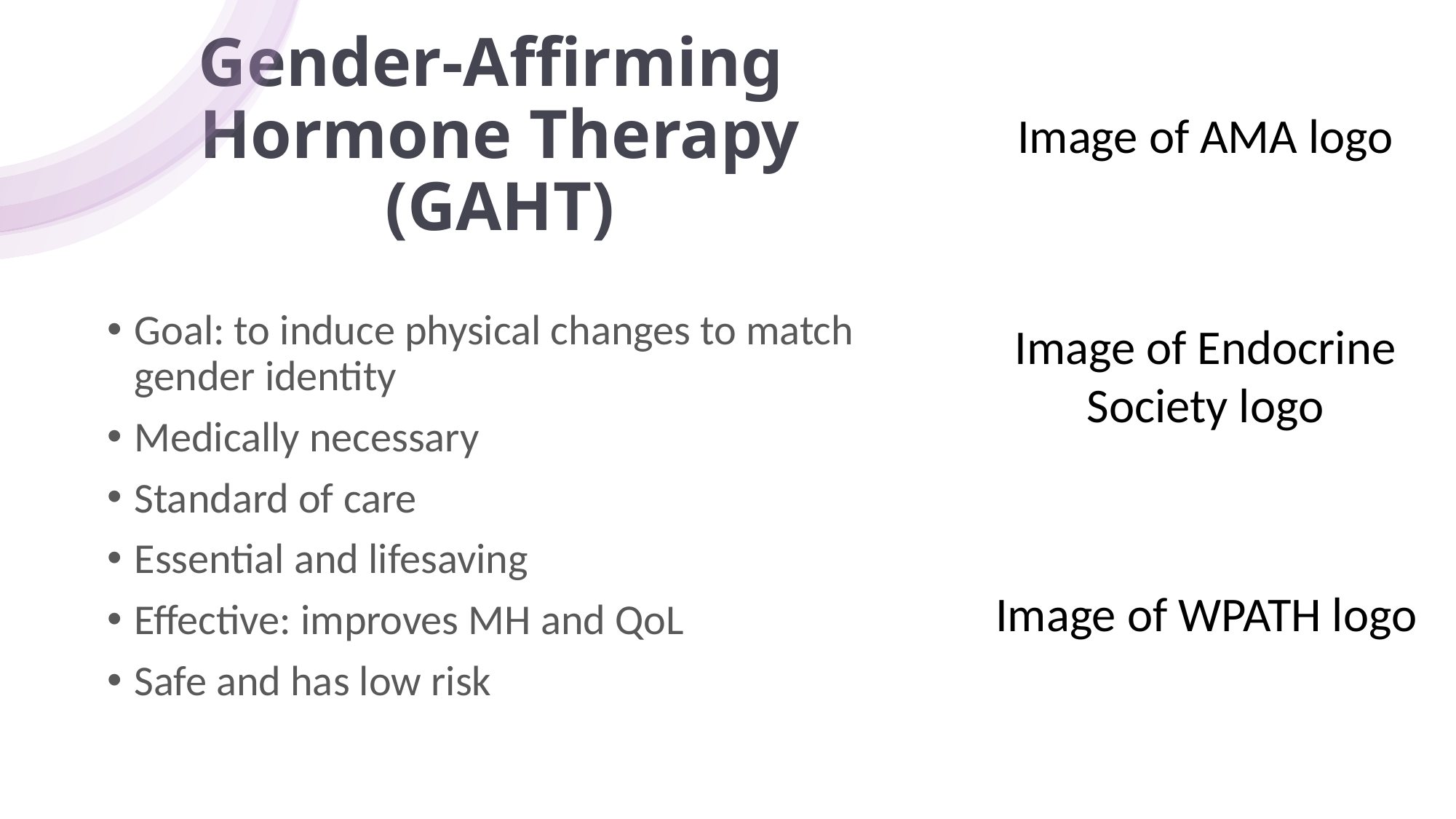

# Gender-Affirming Hormone Therapy (GAHT)
Image of AMA logo
Goal: to induce physical changes to match gender identity
Medically necessary
Standard of care
Essential and lifesaving
Effective: improves MH and QoL
Safe and has low risk
Image of Endocrine Society logo
Image of WPATH logo

## Slide 14
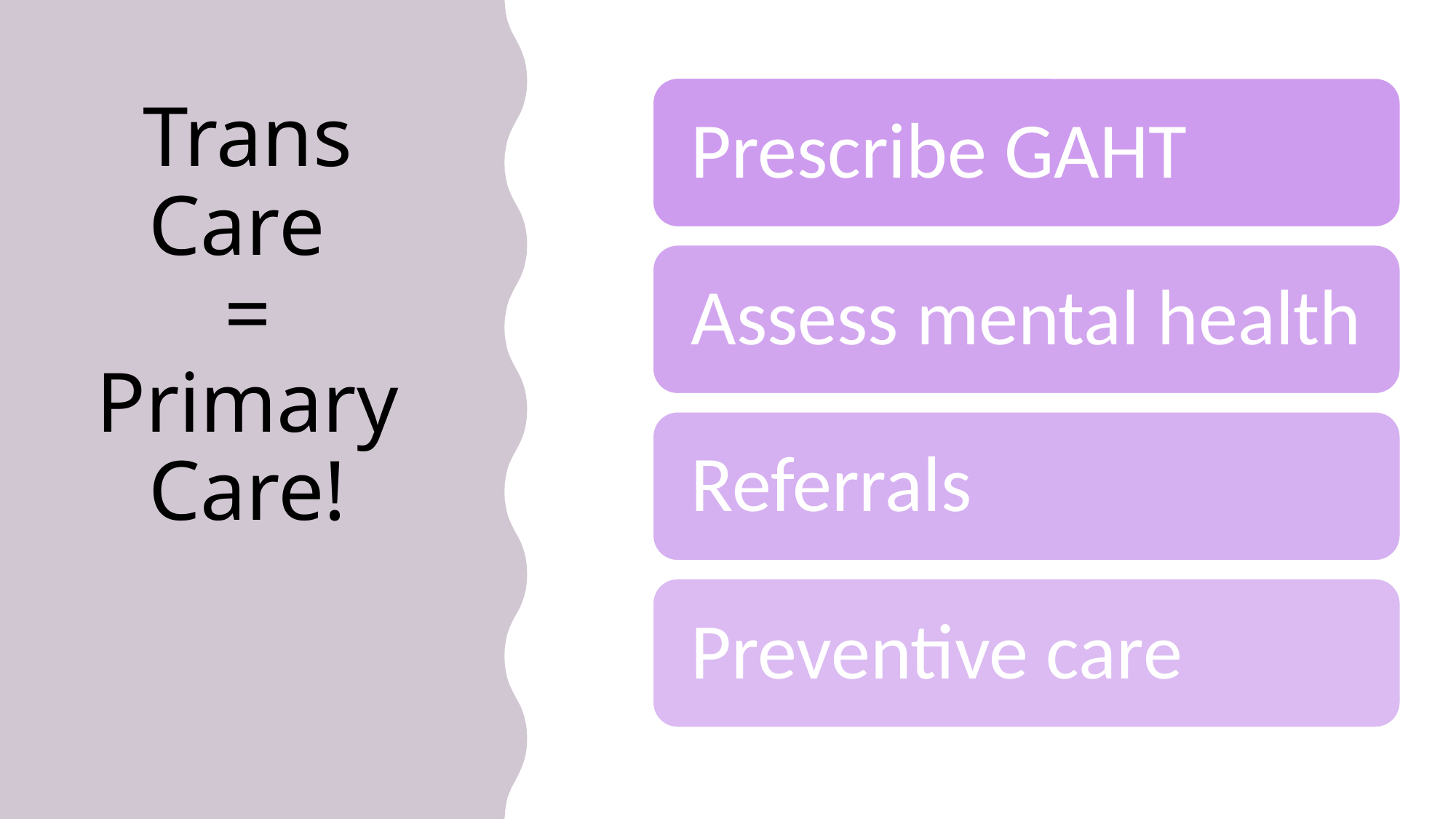

# Trans Care = Primary Care!

## Slide 15
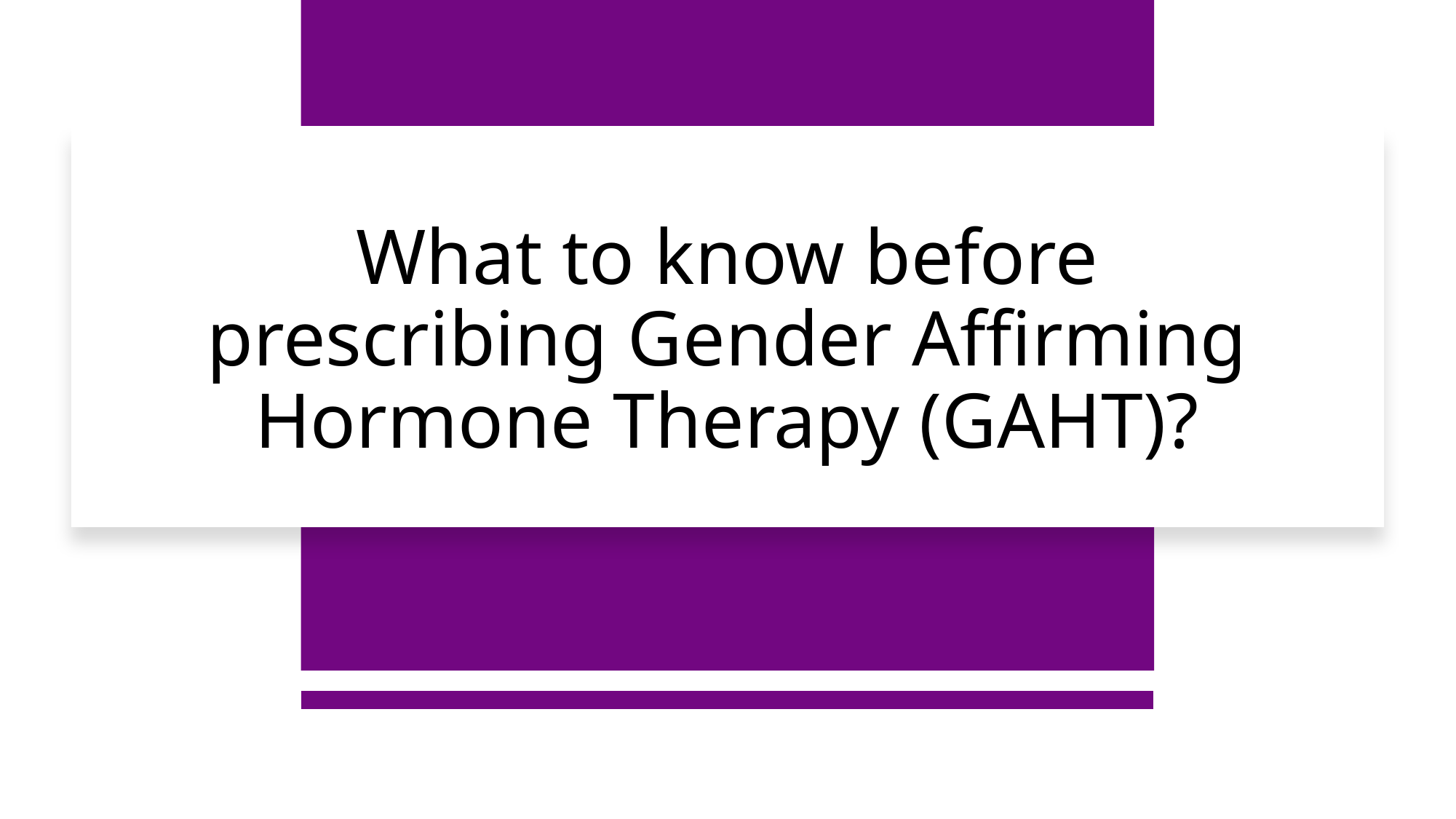

# What to know before prescribing Gender Affirming Hormone Therapy (GAHT)?

## Slide 16
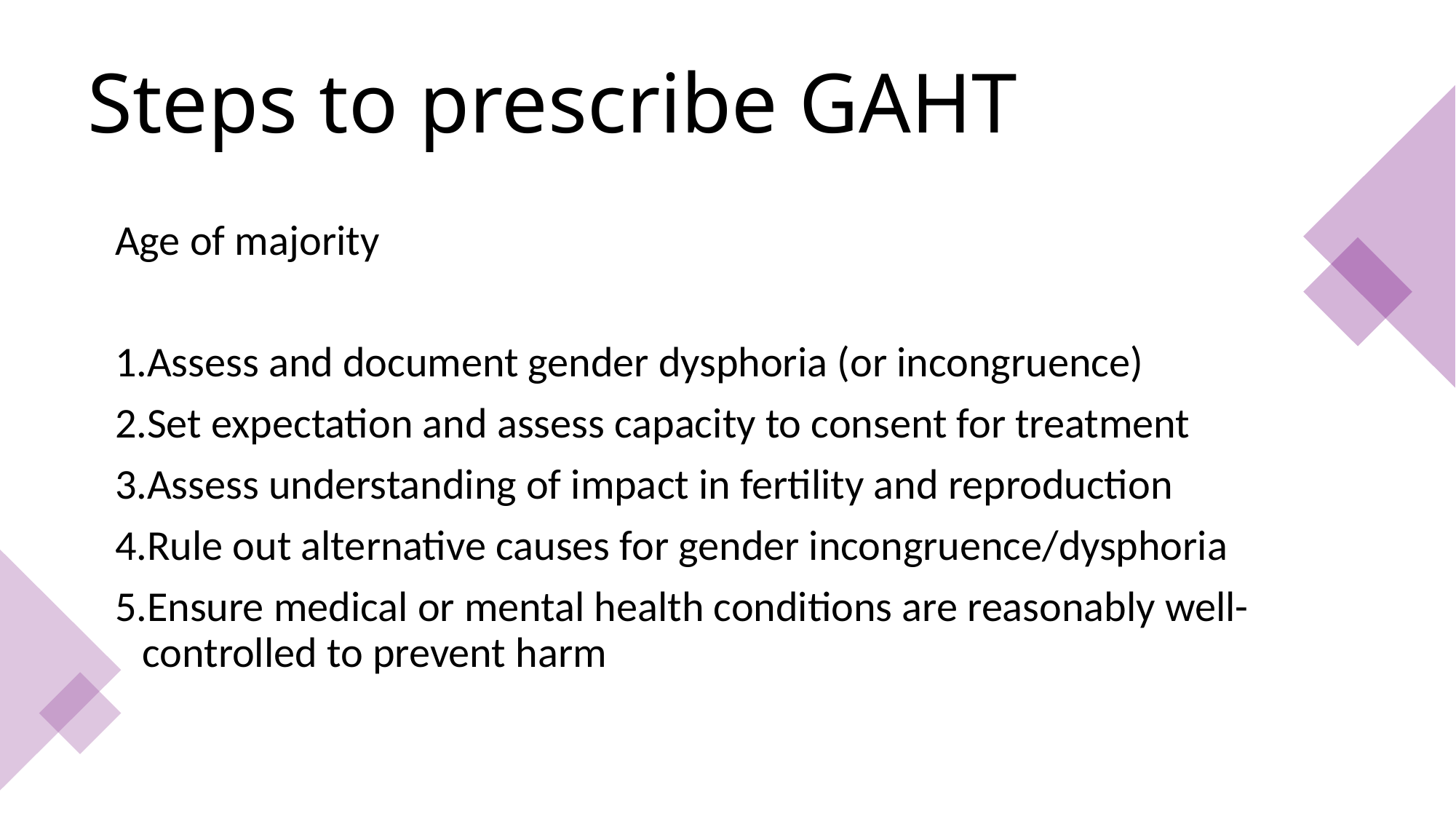

# Steps to prescribe GAHT
Age of majority
Assess and document gender dysphoria (or incongruence)
Set expectation and assess capacity to consent for treatment
Assess understanding of impact in fertility and reproduction
Rule out alternative causes for gender incongruence/dysphoria
Ensure medical or mental health conditions are reasonably well-controlled to prevent harm

## Slide 17
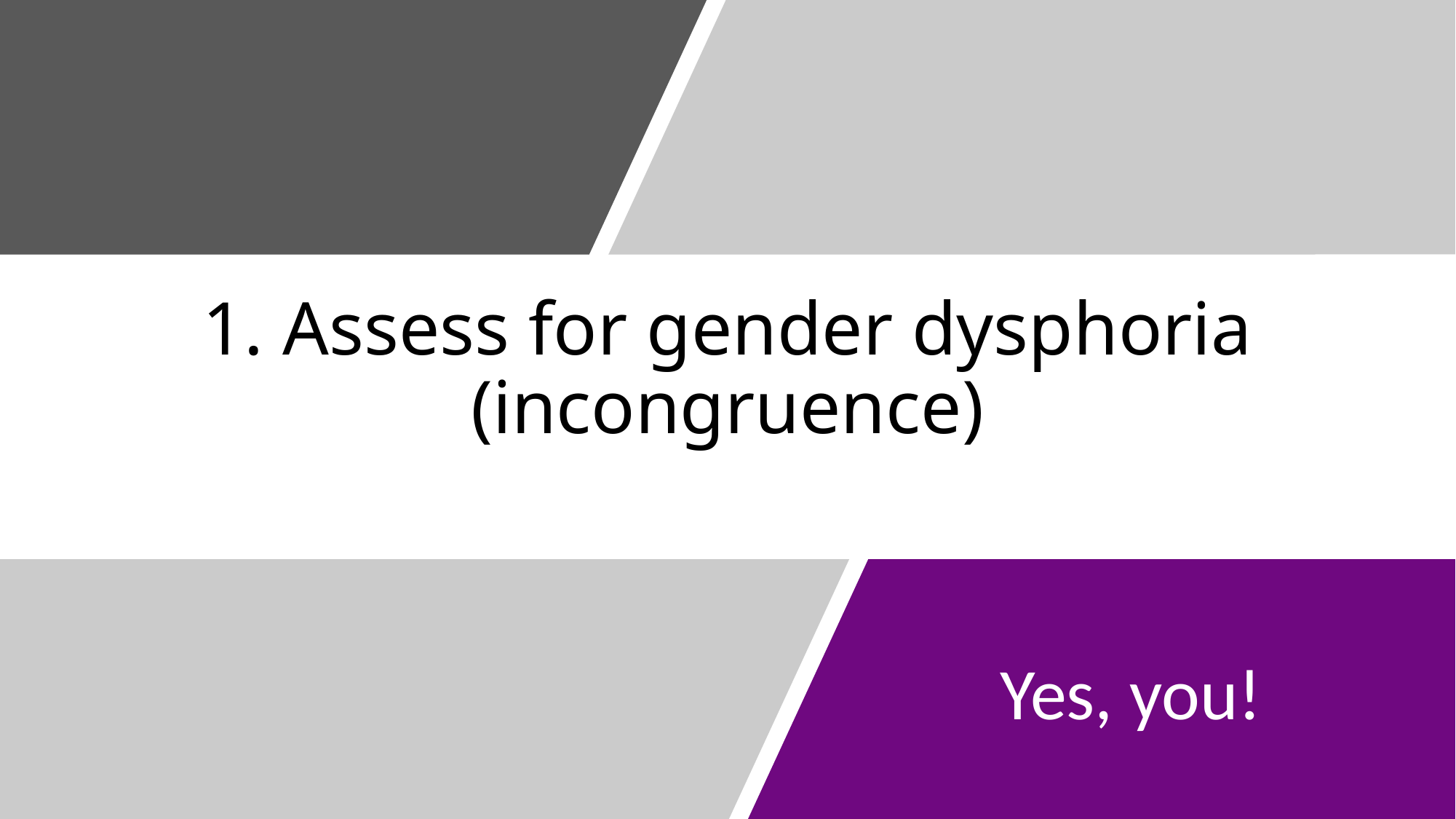

# 1. Assess for gender dysphoria (incongruence)
Yes, you!

## Slide 18
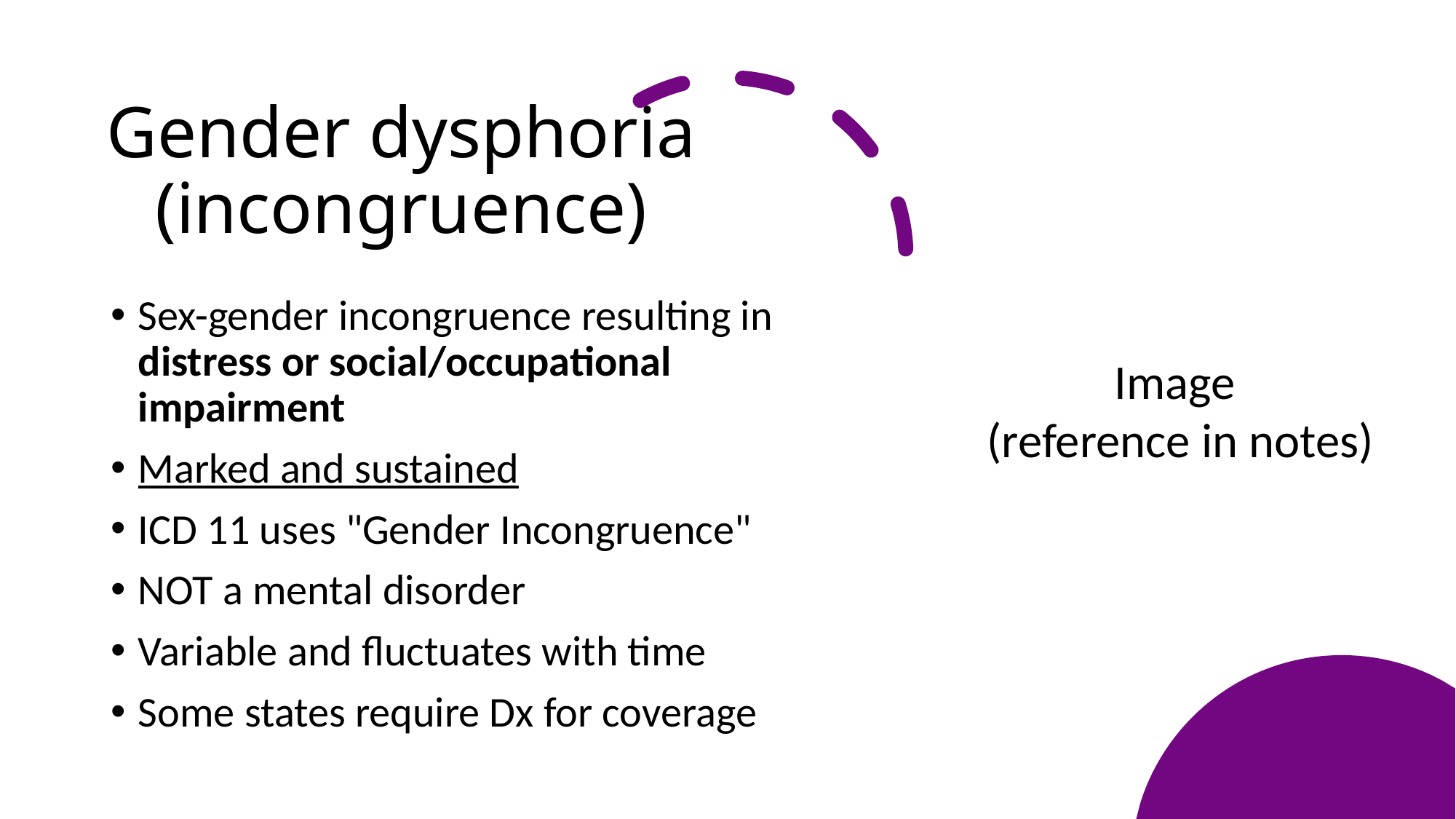

# Gender dysphoria (incongruence)
Sex-gender incongruence resulting in distress or social/occupational impairment
Marked and sustained
ICD 11 uses "Gender Incongruence"
NOT a mental disorder
Variable and fluctuates with time
Some states require Dx for coverage
Image
(reference in notes)

## Slide 19
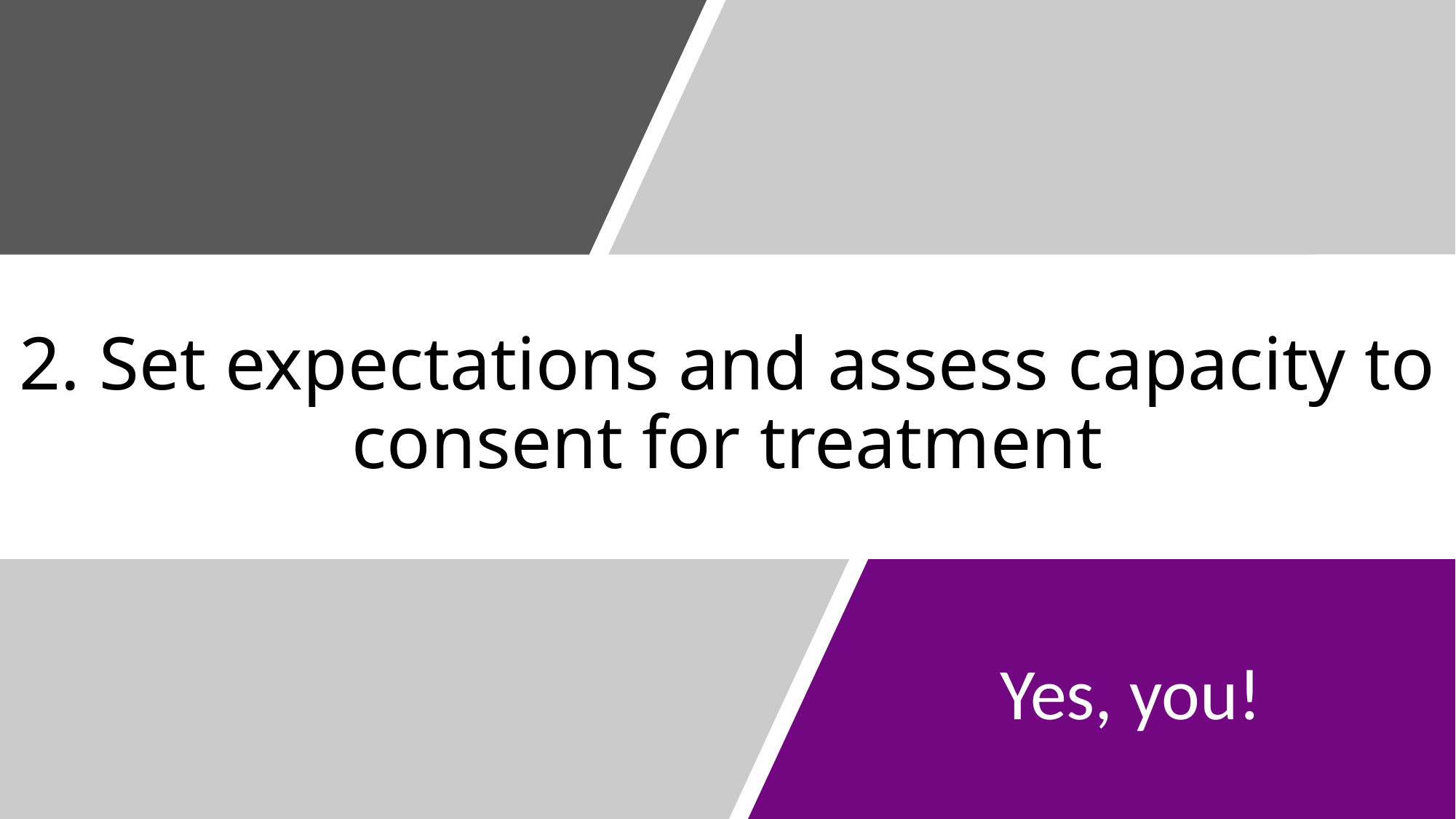

# 2. Set expectations and assess capacity to consent for treatment
Yes, you!

## Slide 20
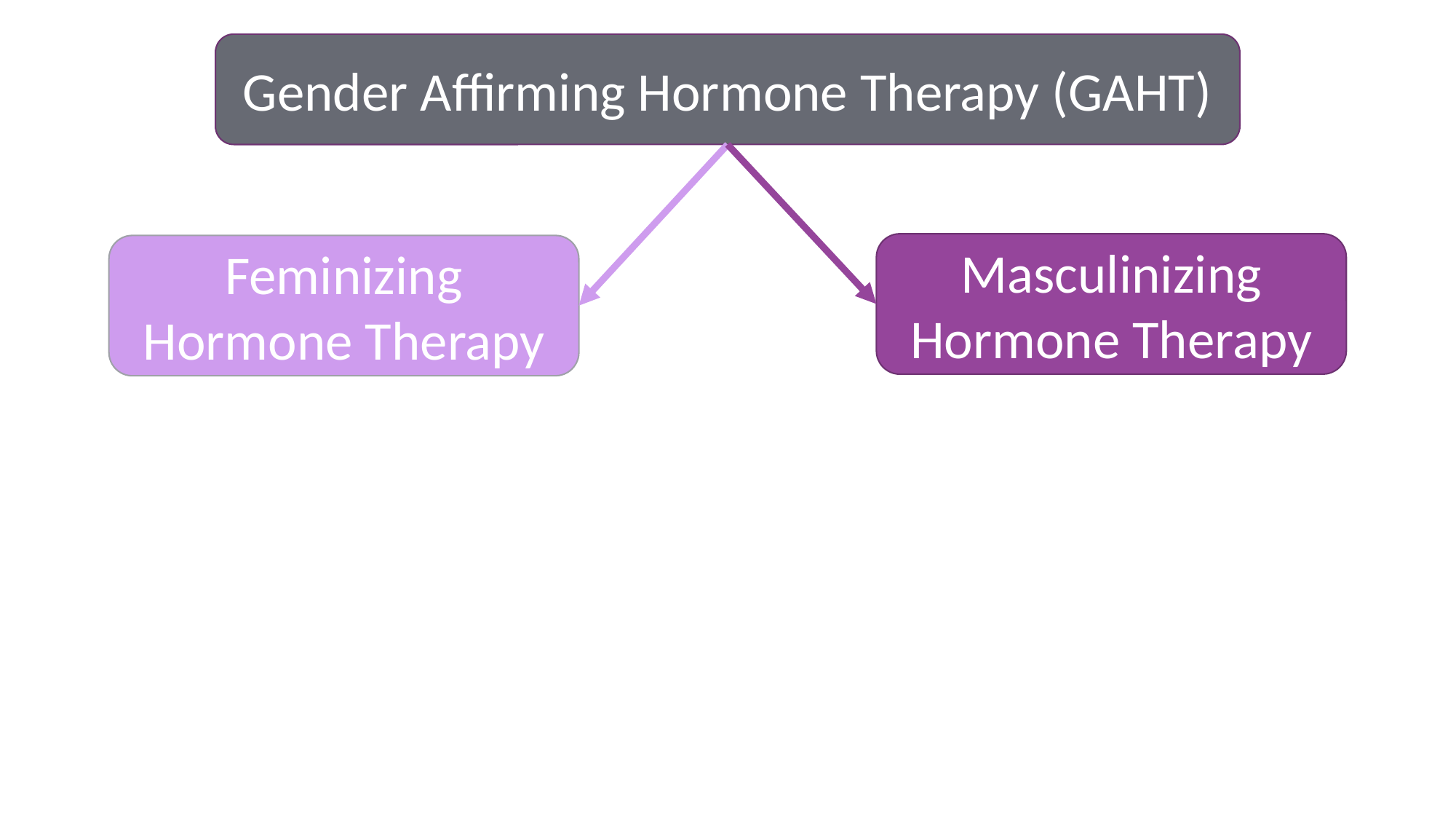

Gender Affirming Hormone Therapy (GAHT)
Masculinizing
Hormone Therapy
Feminizing
Hormone Therapy

## Slide 21
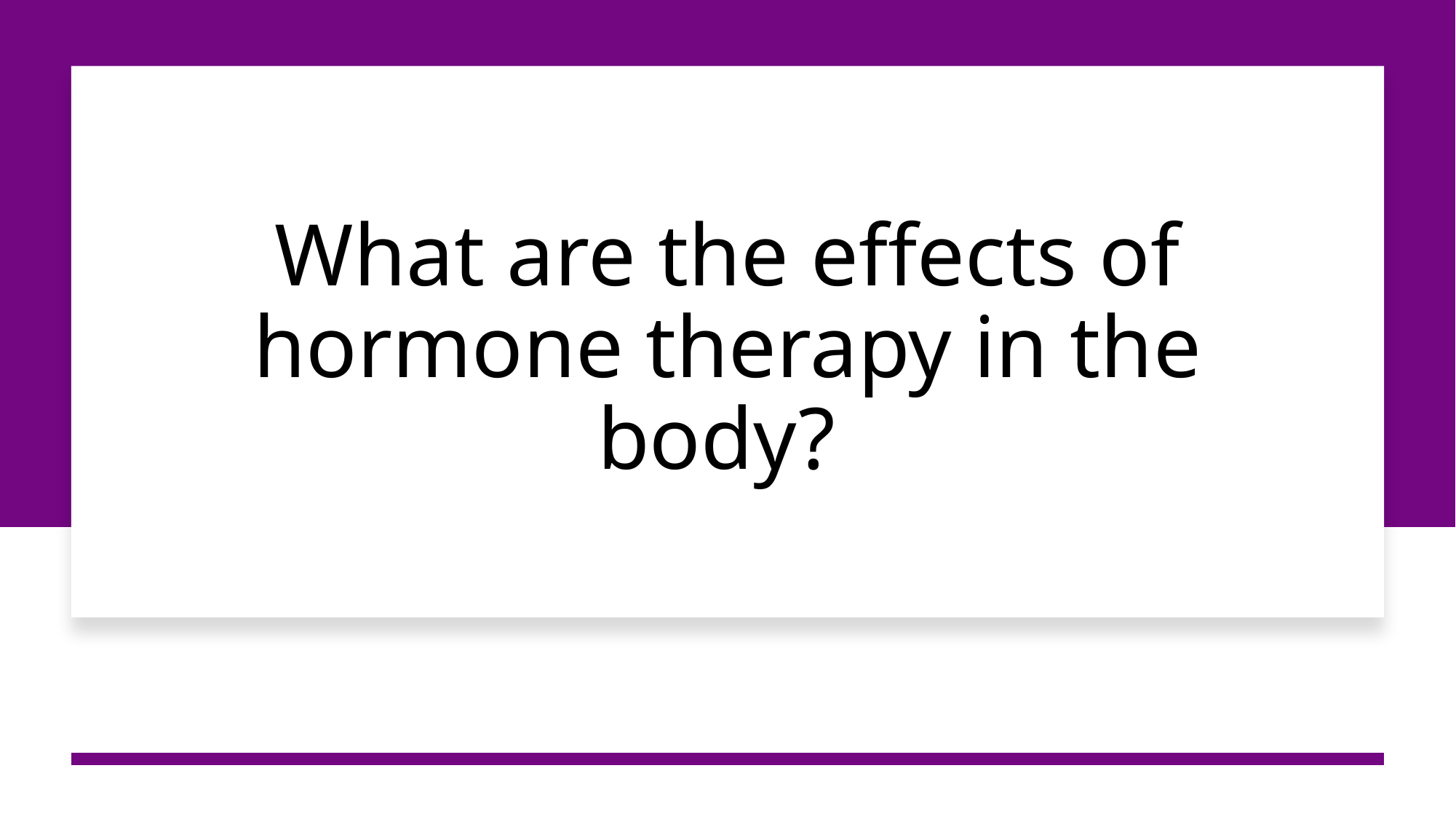

# What are the effects of hormone therapy in the body?

## Slide 22
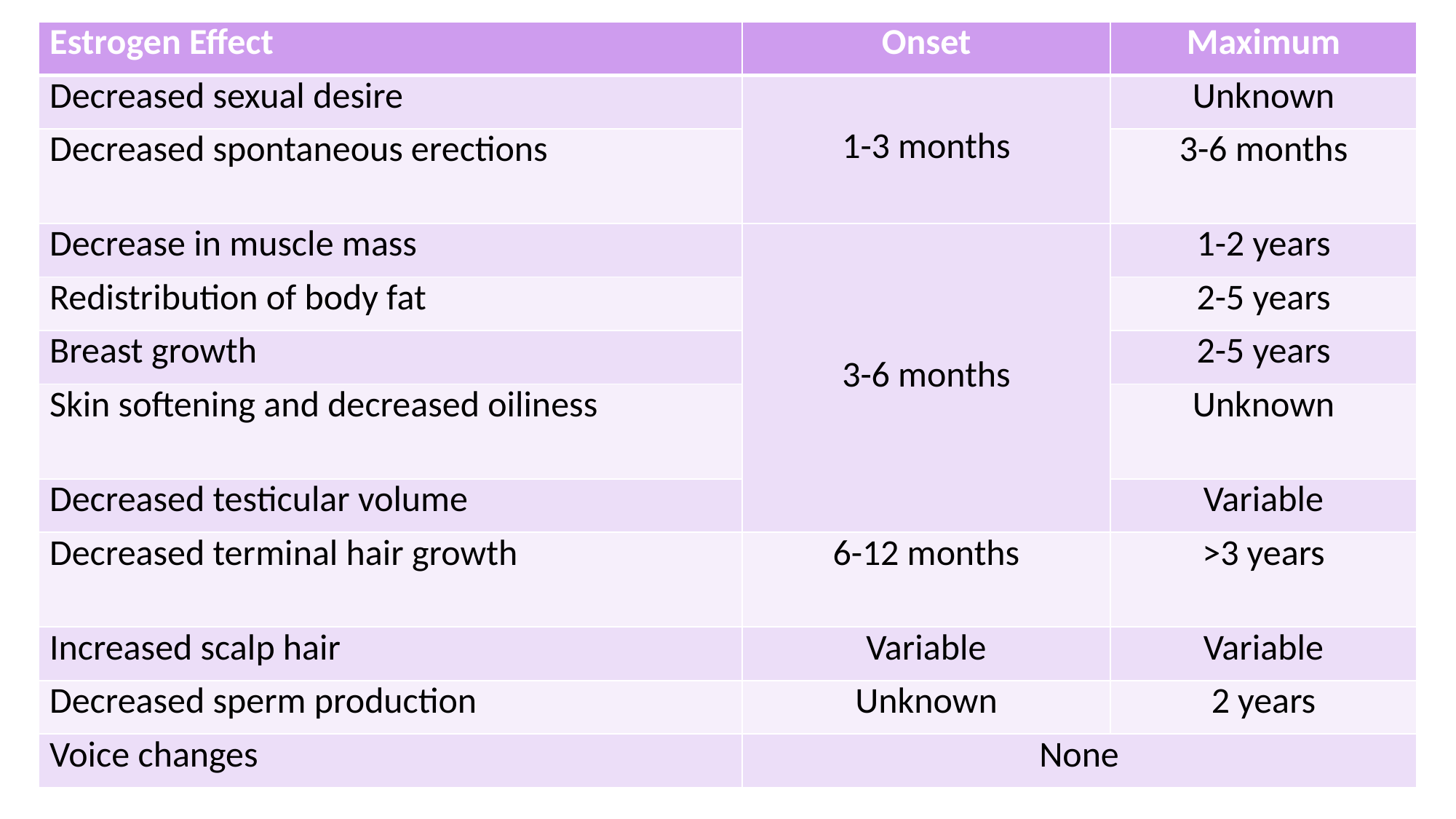

| Estrogen Effect | Onset | Maximum |
| --- | --- | --- |
| Decreased sexual desire | 1-3 months | Unknown |
| Decreased spontaneous erections | | 3-6 months |
| Decrease in muscle mass | 3-6 months | 1-2 years |
| Redistribution of body fat | | 2-5 years |
| Breast growth | | 2-5 years |
| Skin softening and decreased oiliness | | Unknown |
| Decreased testicular volume | | Variable |
| Decreased terminal hair growth | 6-12 months | >3 years |
| Increased scalp hair | Variable | Variable |
| Decreased sperm production | Unknown | 2 years |
| Voice changes | None | |

## Slide 23
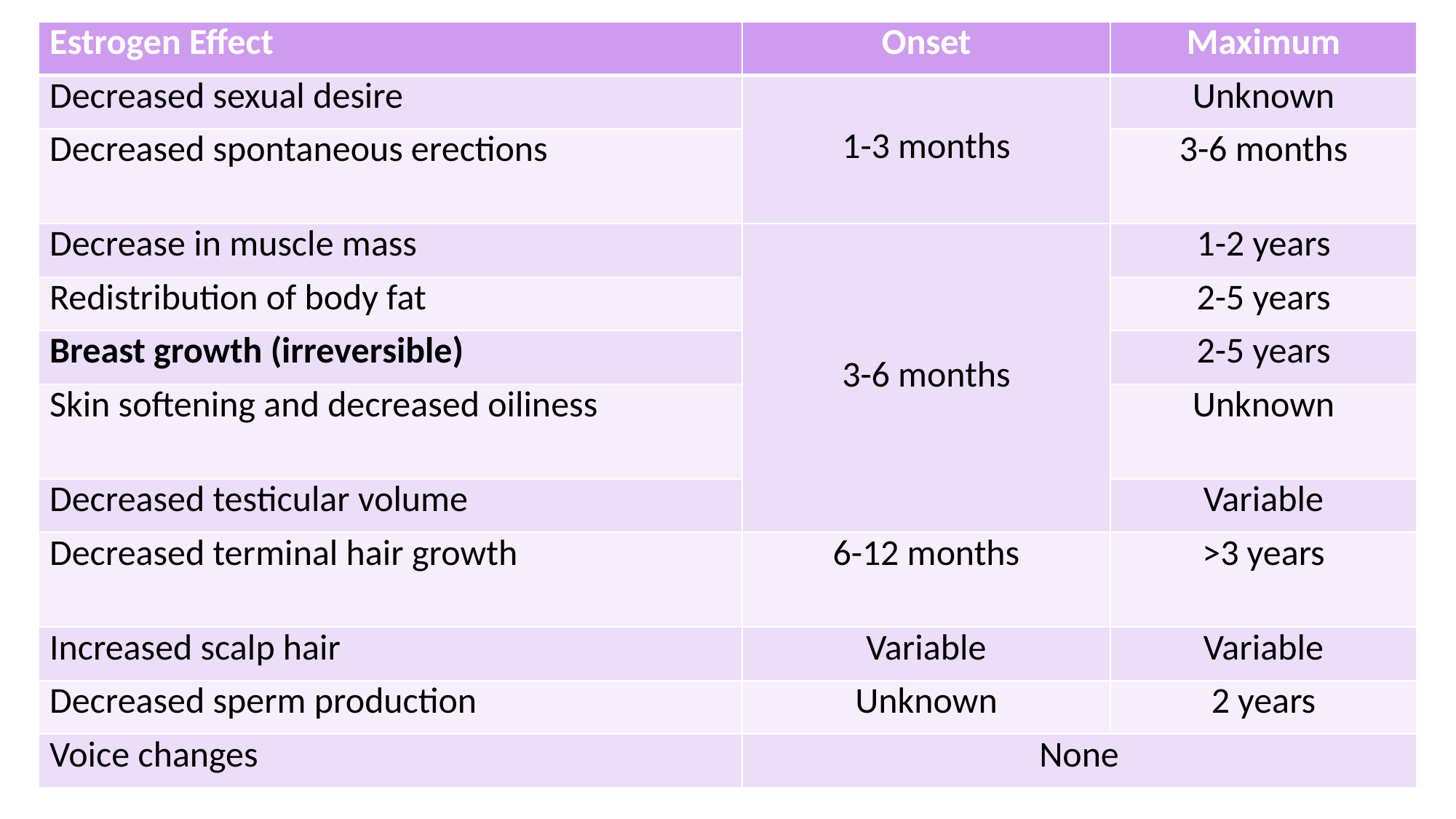

| Estrogen Effect | Onset | Maximum |
| --- | --- | --- |
| Decreased sexual desire | 1-3 months | Unknown |
| Decreased spontaneous erections | | 3-6 months |
| Decrease in muscle mass | 3-6 months | 1-2 years |
| Redistribution of body fat | | 2-5 years |
| Breast growth (irreversible) | | 2-5 years |
| Skin softening and decreased oiliness | | Unknown |
| Decreased testicular volume | | Variable |
| Decreased terminal hair growth | 6-12 months | >3 years |
| Increased scalp hair | Variable | Variable |
| Decreased sperm production | Unknown | 2 years |
| Voice changes | None | |

## Slide 24
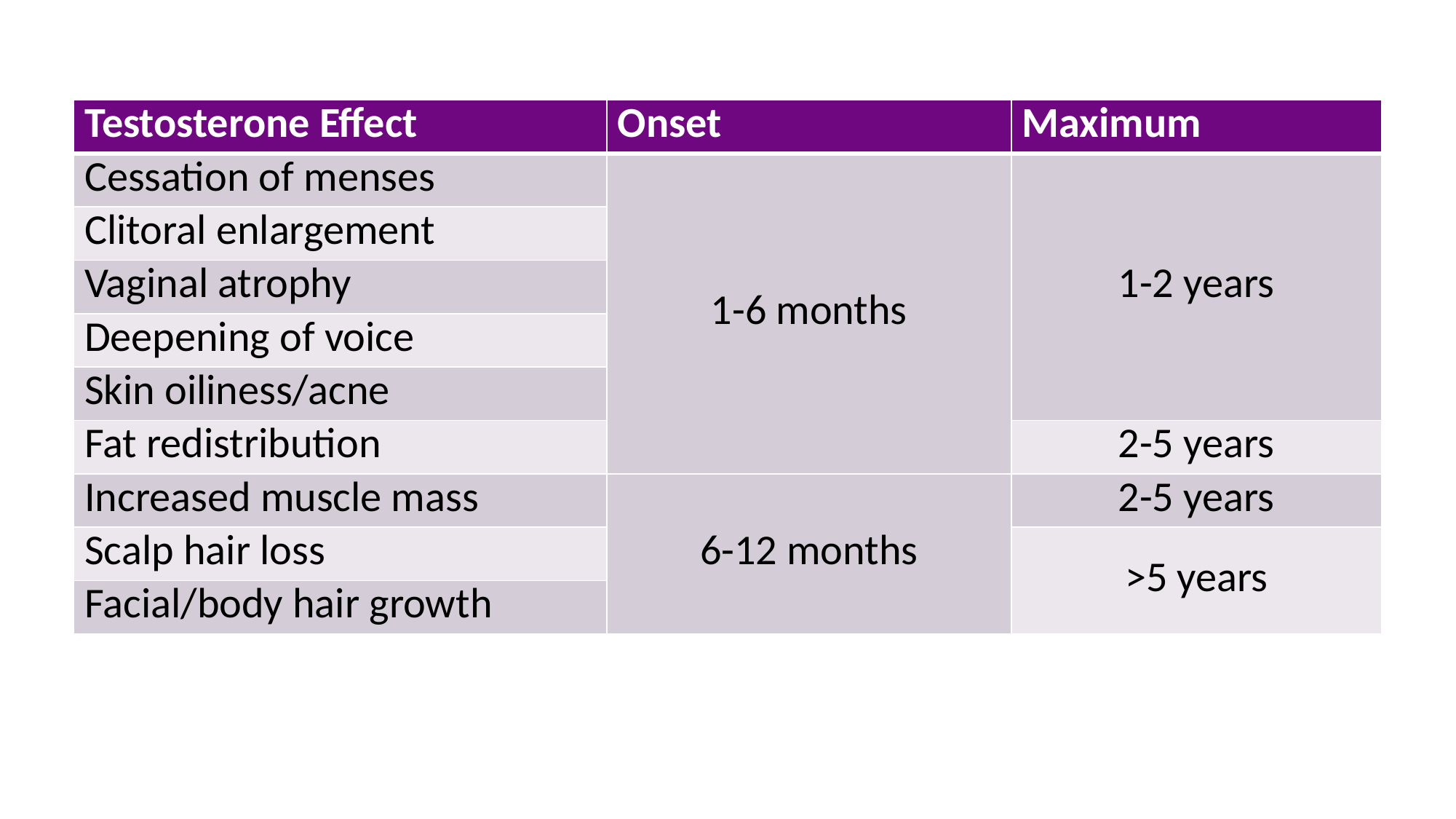

| Testosterone Effect | Onset | Maximum |
| --- | --- | --- |
| Cessation of menses | 1-6 months | 1-2 years |
| Clitoral enlargement | | |
| Vaginal atrophy | | |
| Deepening of voice | | |
| Skin oiliness/acne | | |
| Fat redistribution | | 2-5 years |
| Increased muscle mass | 6-12 months | 2-5 years |
| Scalp hair loss | | >5 years |
| Facial/body hair growth | | |

## Slide 25
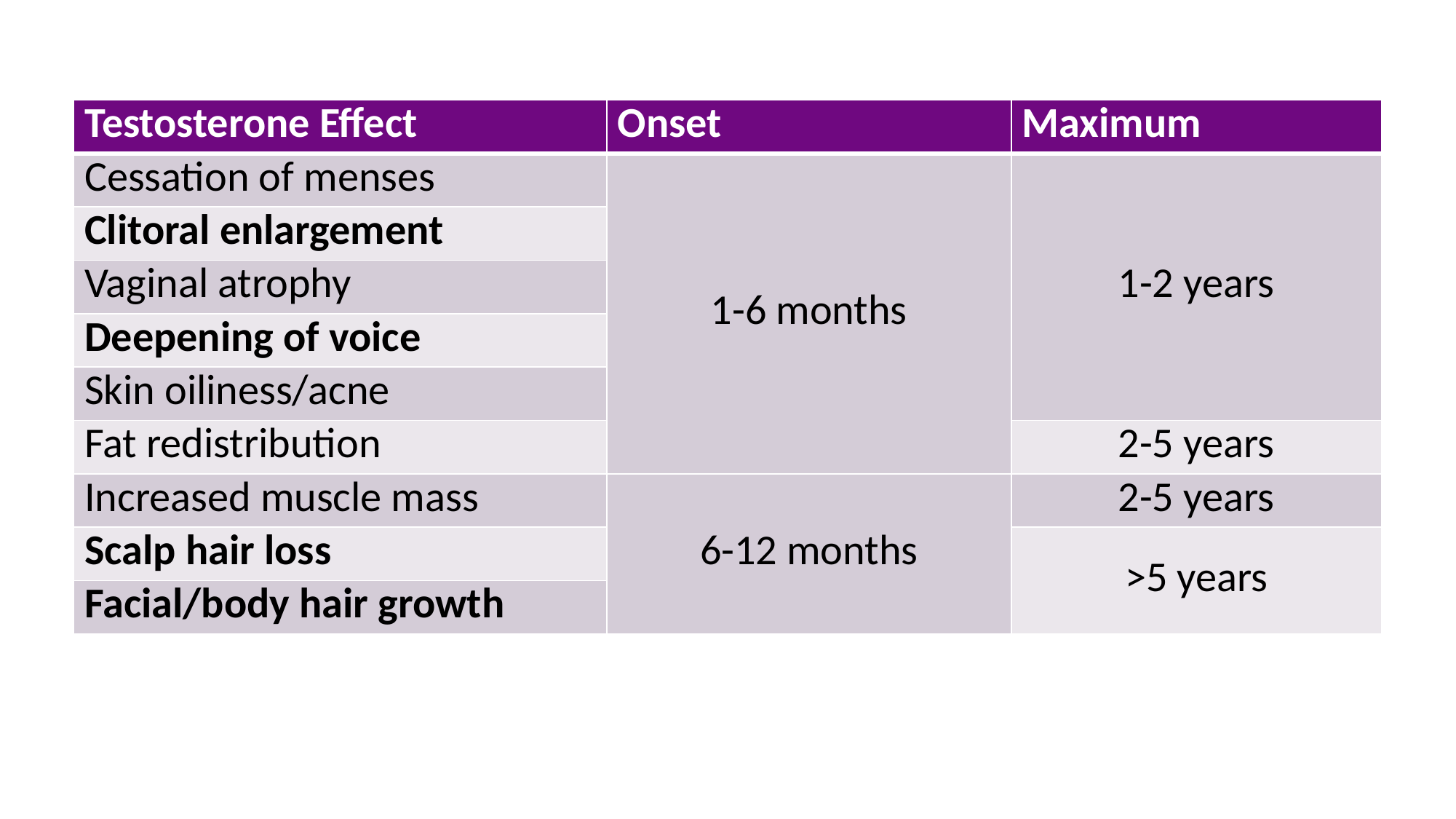

| Testosterone Effect | Onset | Maximum |
| --- | --- | --- |
| Cessation of menses | 1-6 months | 1-2 years |
| Clitoral enlargement | | |
| Vaginal atrophy | | |
| Deepening of voice | | |
| Skin oiliness/acne | | |
| Fat redistribution | | 2-5 years |
| Increased muscle mass | 6-12 months | 2-5 years |
| Scalp hair loss | | >5 years |
| Facial/body hair growth | | |

## Slide 26
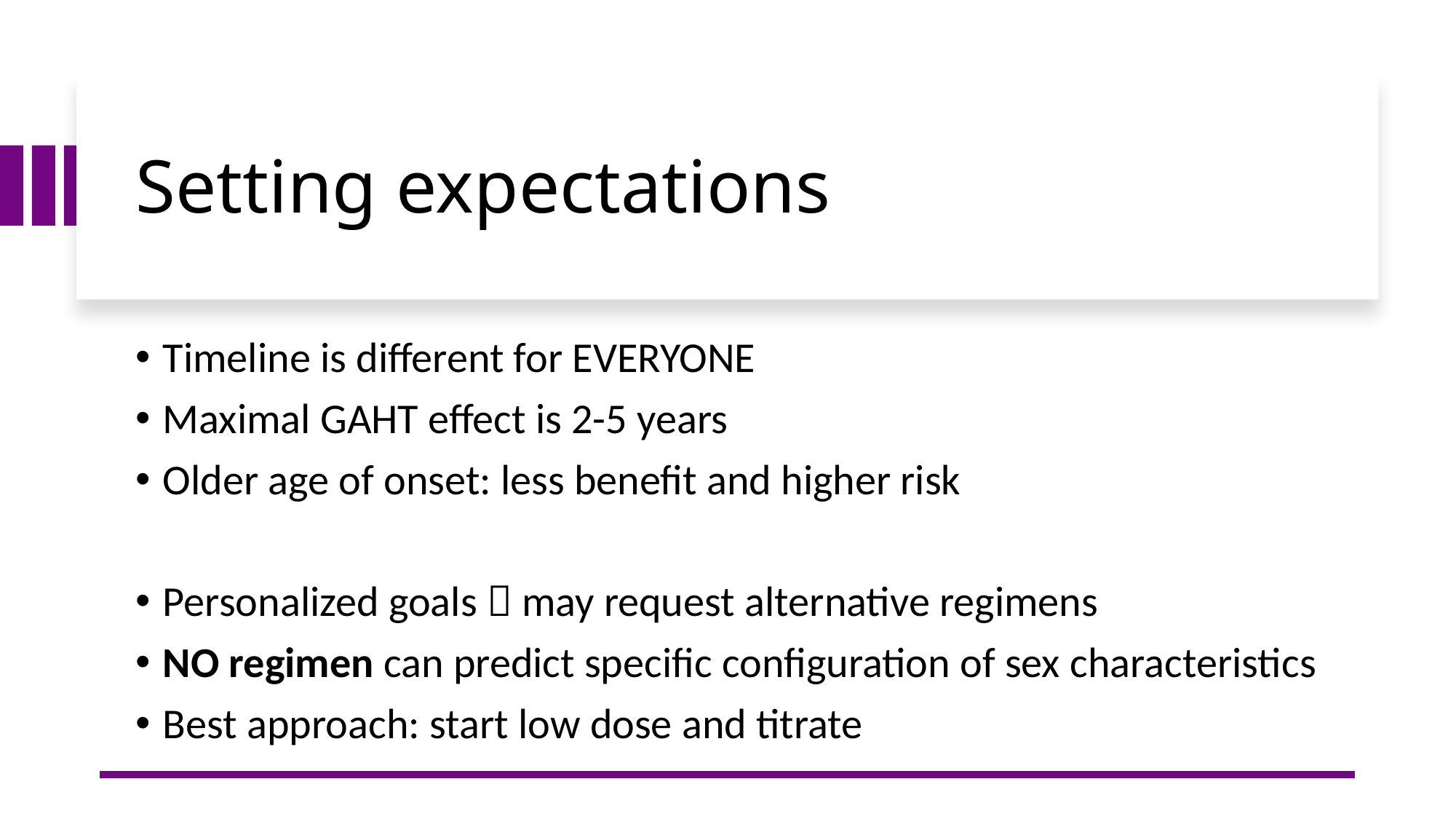

# Setting expectations
Timeline is different for EVERYONE
Maximal GAHT effect is 2-5 years
Older age of onset: less benefit and higher risk
Personalized goals  may request alternative regimens
NO regimen can predict specific configuration of sex characteristics
Best approach: start low dose and titrate

## Slide 27
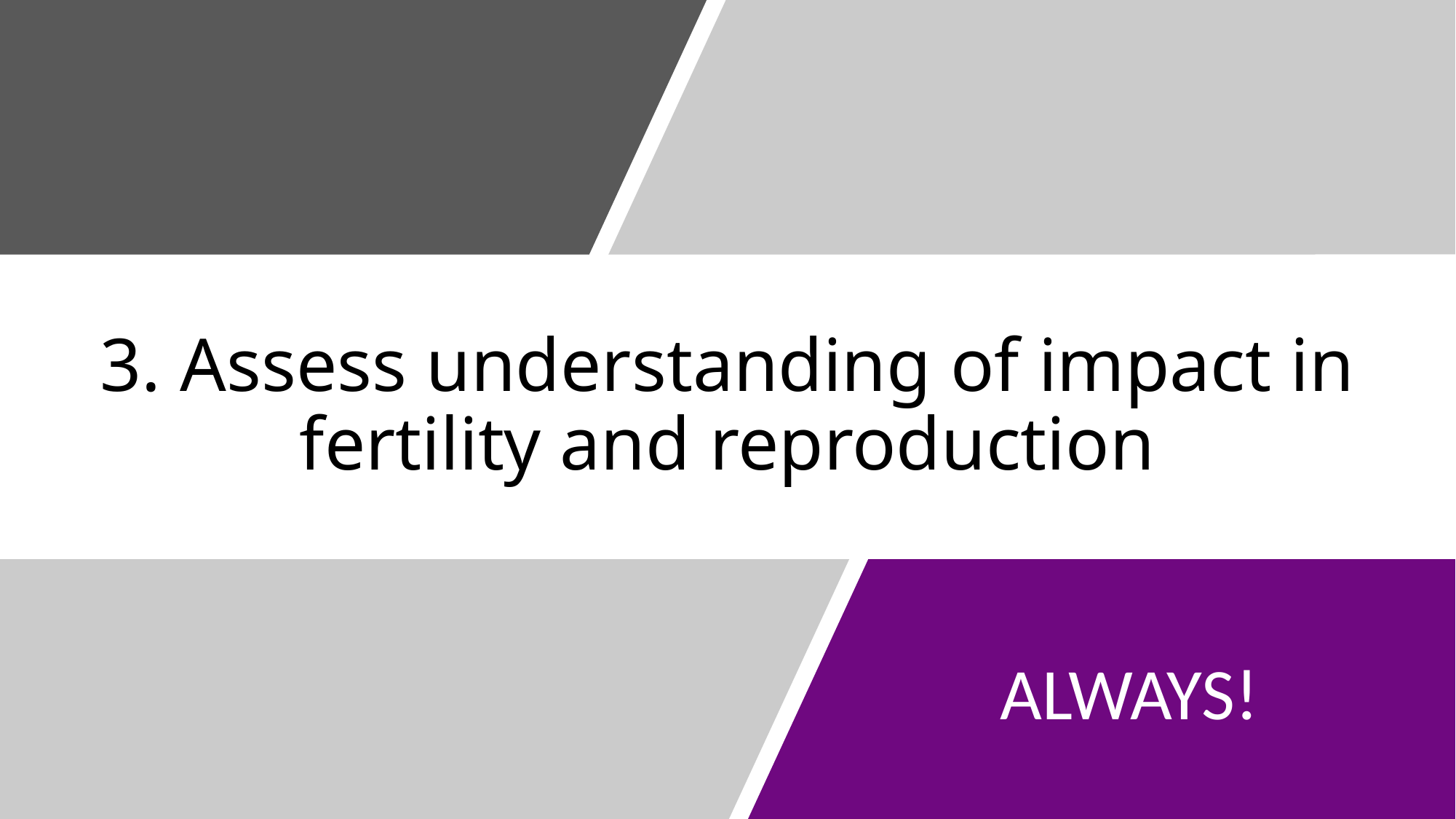

# 3. Assess understanding of impact in fertility and reproduction
ALWAYS!

## Slide 28
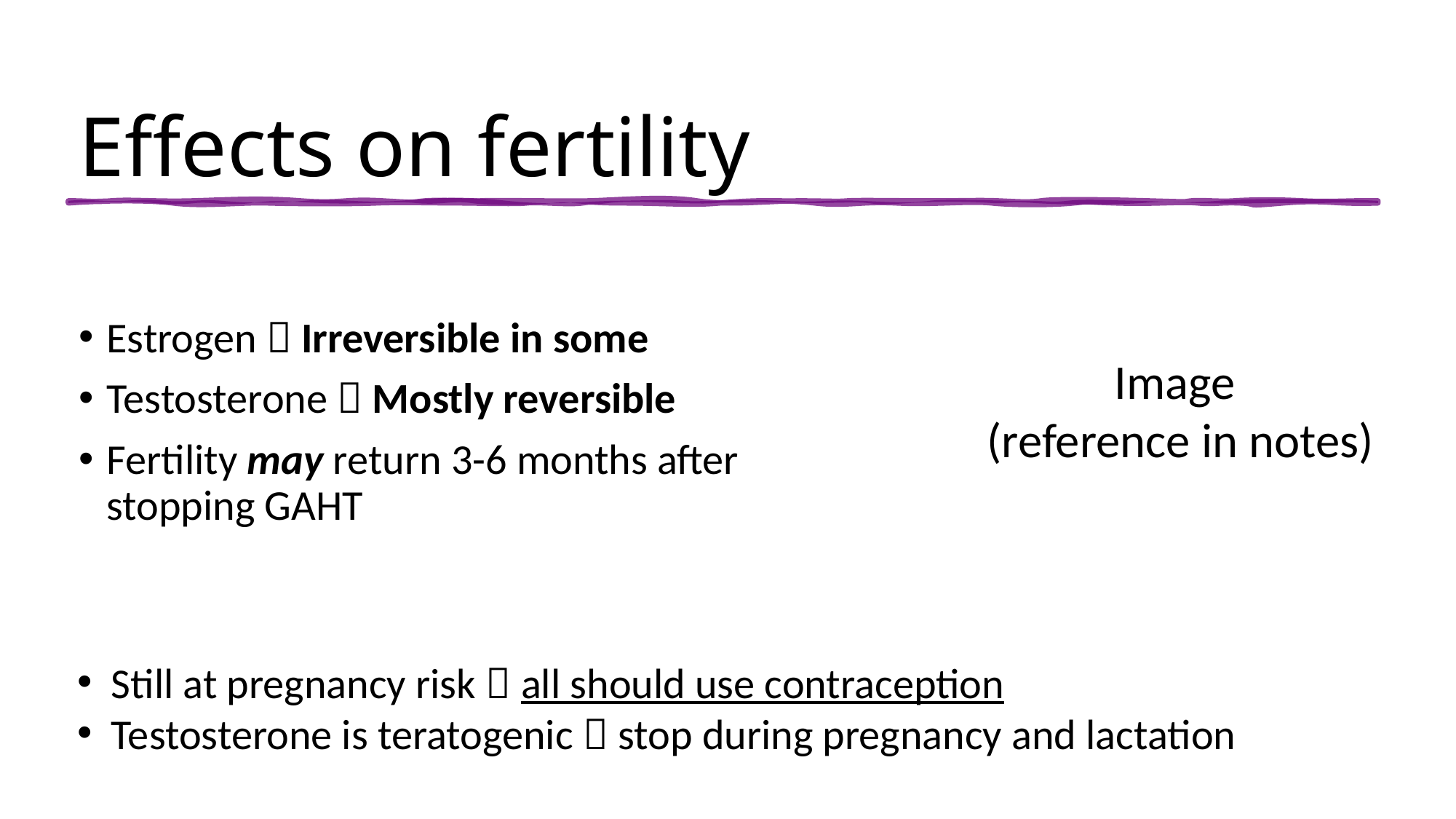

# Effects on fertility
Estrogen  Irreversible in some
Testosterone  Mostly reversible
Fertility may return 3-6 months after stopping GAHT
Image
(reference in notes)
Still at pregnancy risk  all should use contraception
Testosterone is teratogenic  stop during pregnancy and lactation

## Slide 29
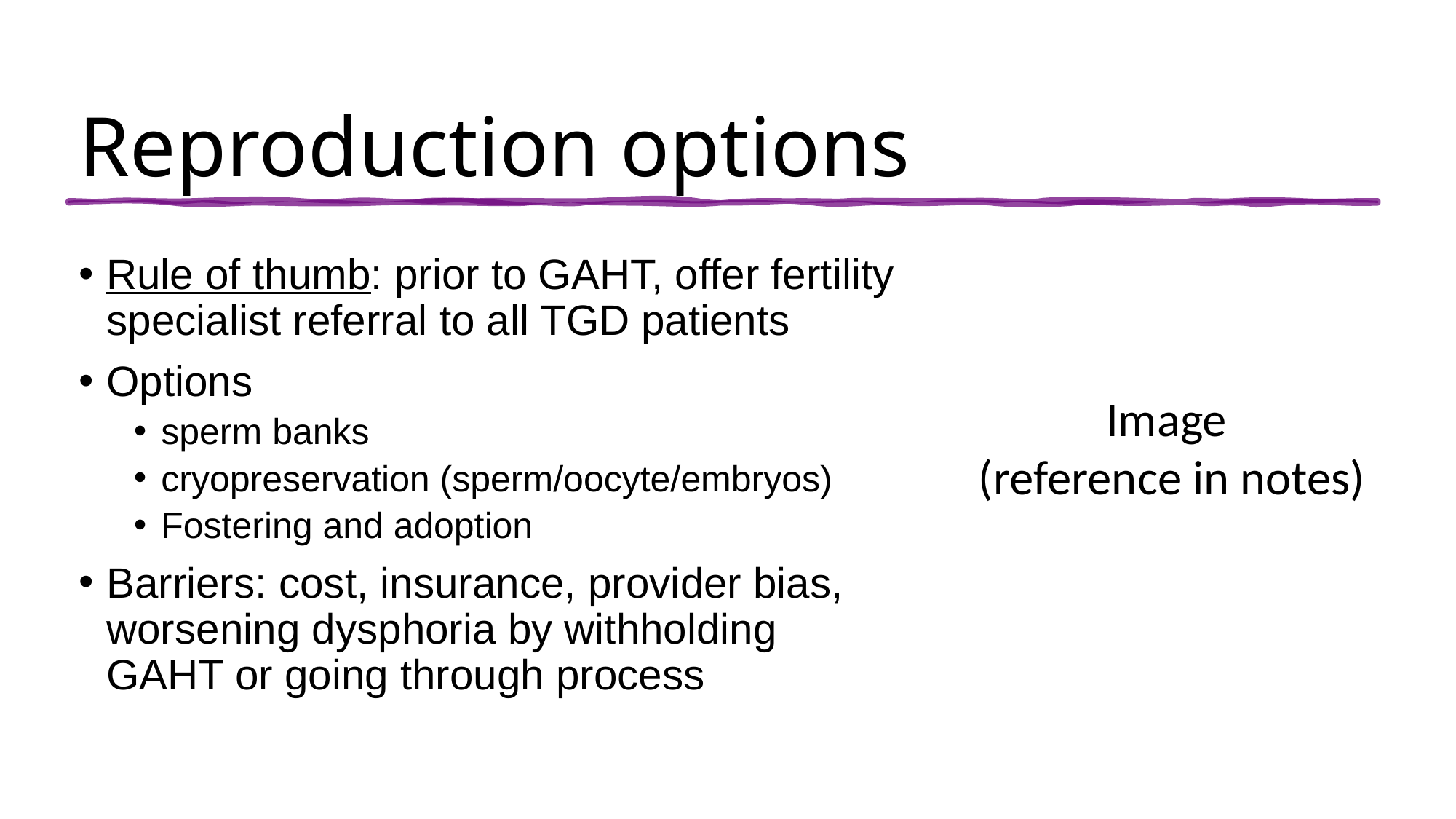

# Reproduction options
Rule of thumb: prior to GAHT, offer fertility specialist referral to all TGD patients
Options
sperm banks
cryopreservation (sperm/oocyte/embryos)
Fostering and adoption
Barriers: cost, insurance, provider bias, worsening dysphoria by withholding GAHT or going through process
Image
(reference in notes)

## Slide 30
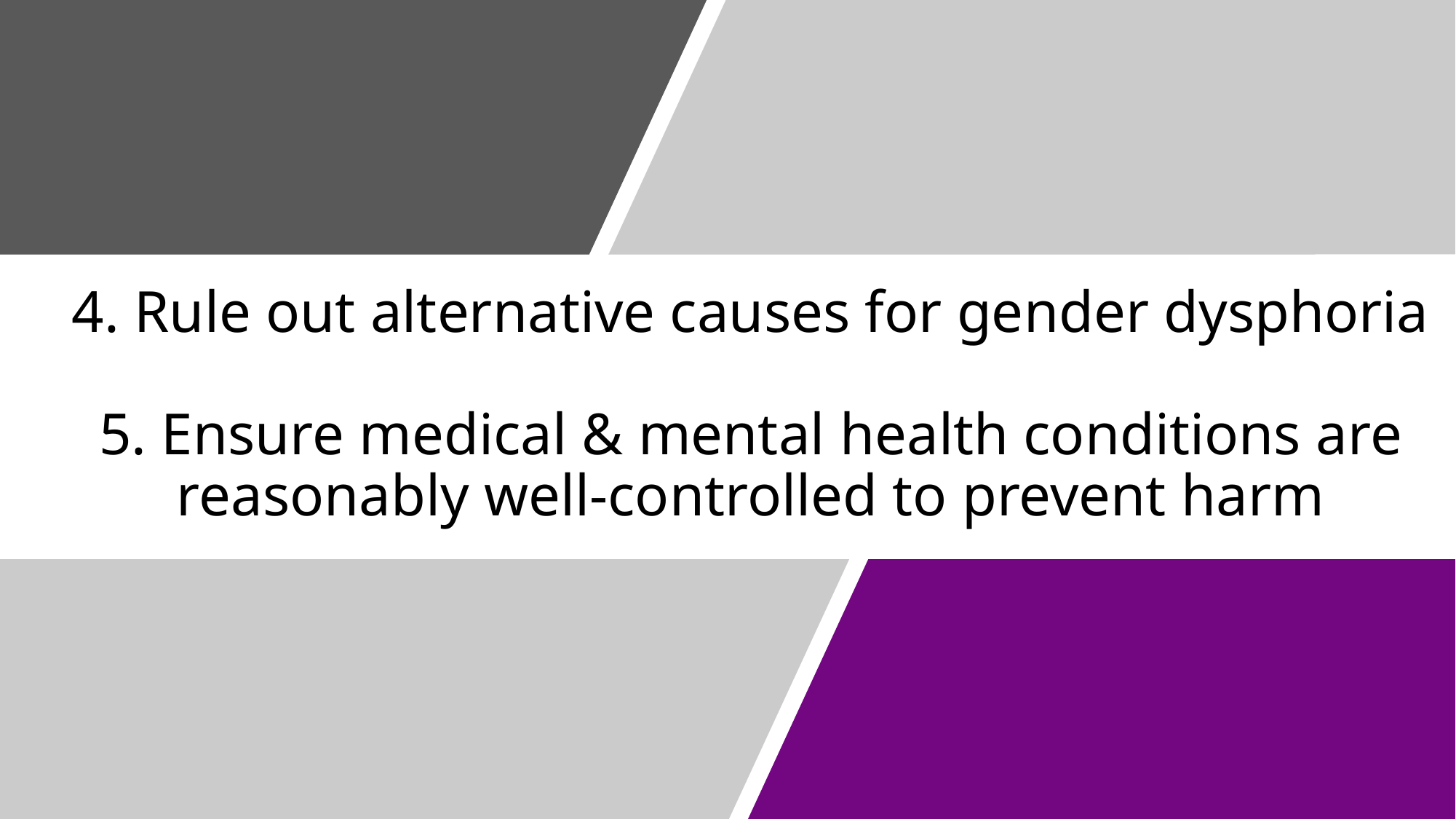

# 4. Rule out alternative causes for gender dysphoria 5. Ensure medical & mental health conditions are reasonably well-controlled to prevent harm

## Slide 31
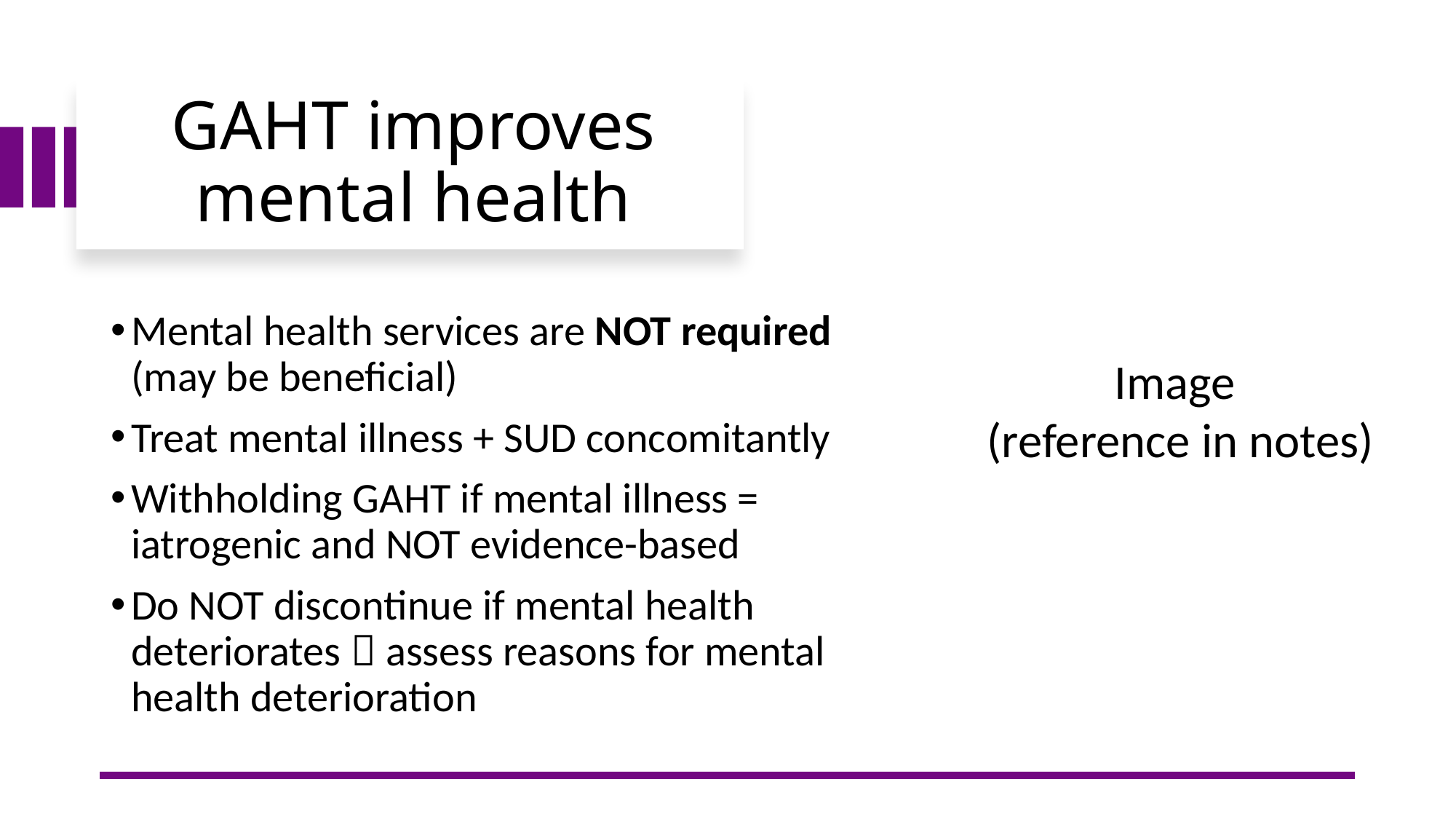

# GAHT improves mental health
Mental health services are NOT required (may be beneficial)
Treat mental illness + SUD concomitantly
Withholding GAHT if mental illness = iatrogenic and NOT evidence-based
Do NOT discontinue if mental health deteriorates  assess reasons for mental health deterioration
Image
(reference in notes)

## Slide 32
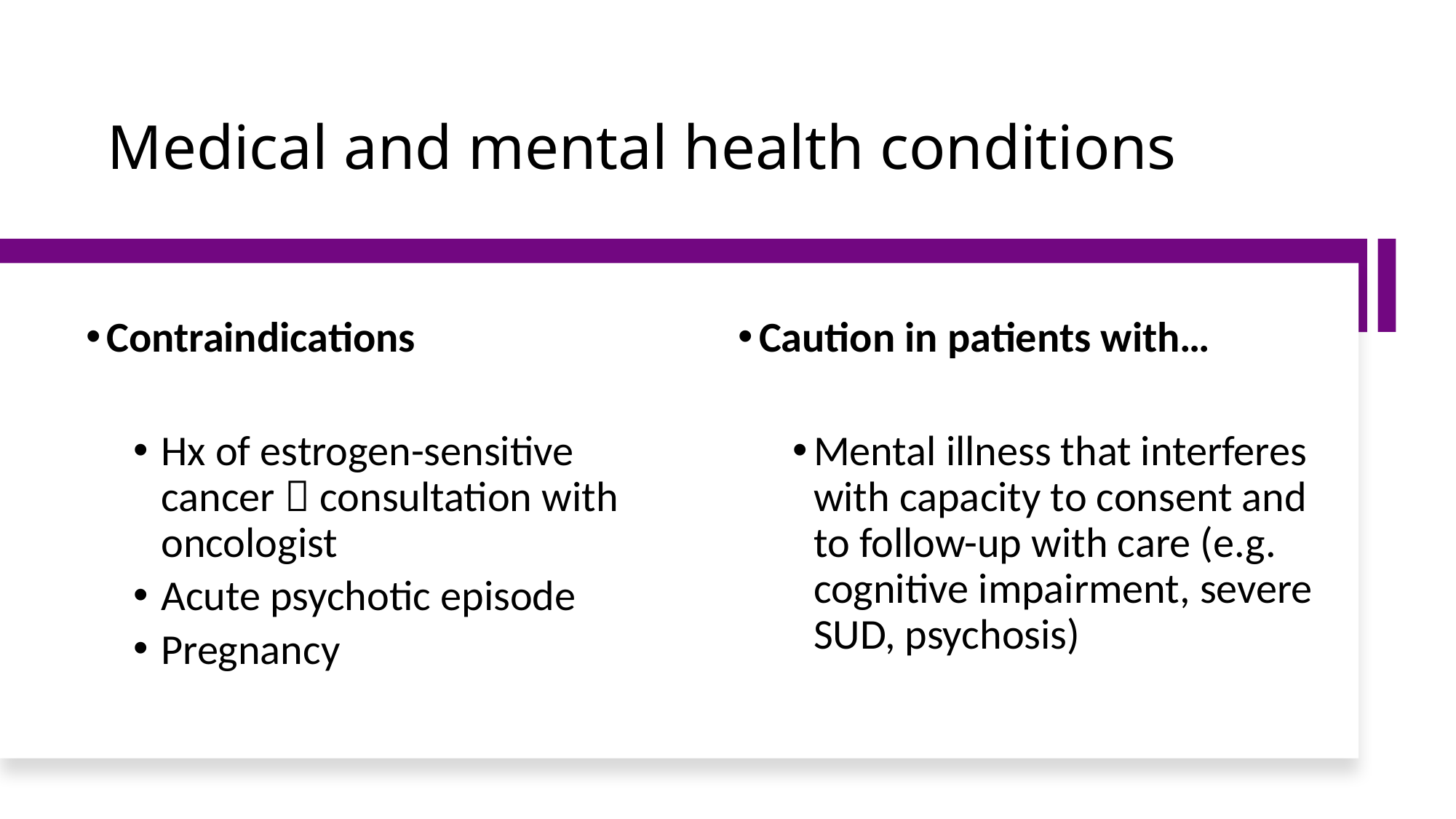

# Medical and mental health conditions
Contraindications
Hx of estrogen-sensitive cancer  consultation with oncologist
Acute psychotic episode
Pregnancy
Caution in patients with…
Mental illness that interferes with capacity to consent and to follow-up with care (e.g. cognitive impairment, severe SUD, psychosis)

## Slide 33
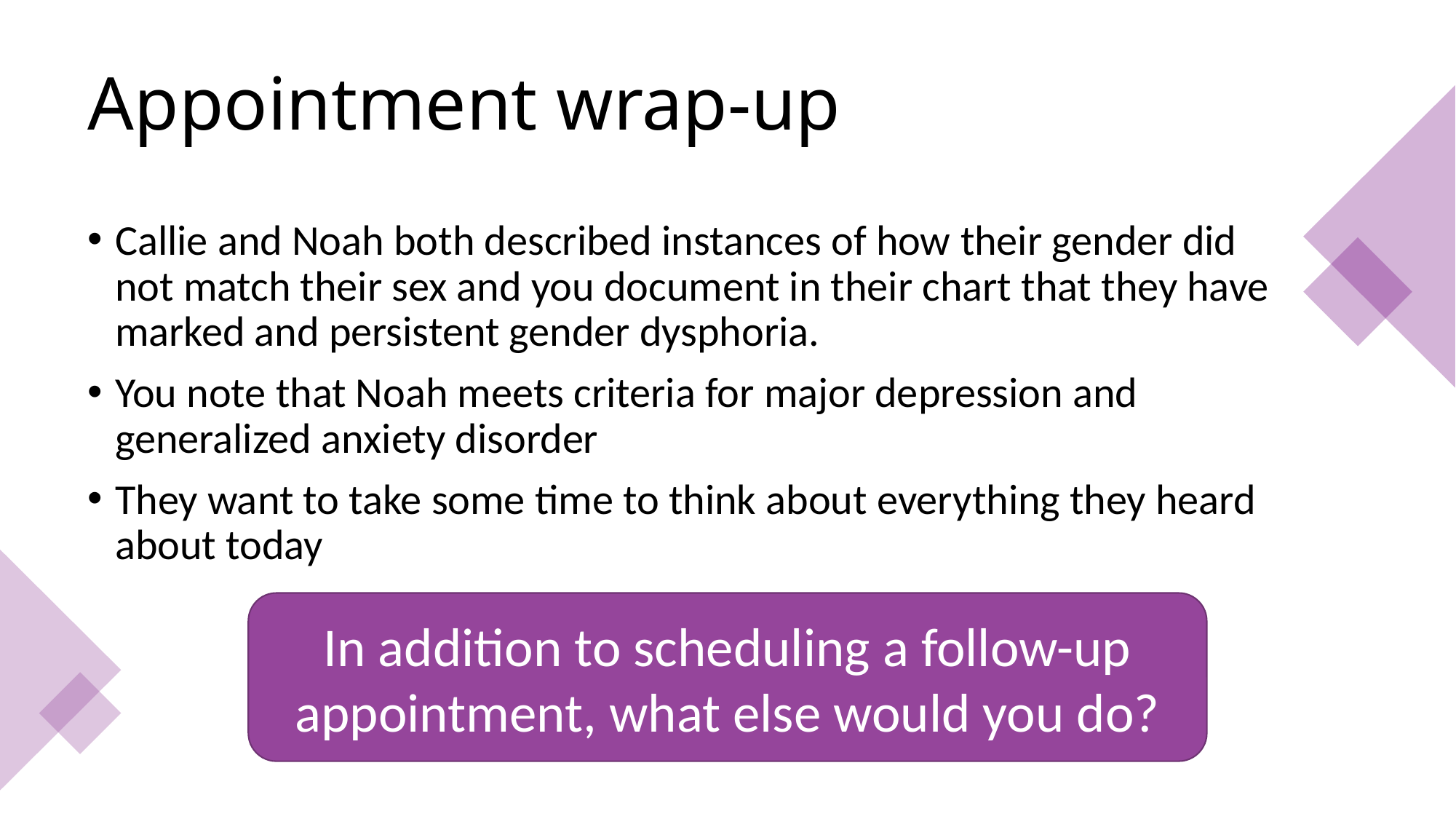

# Appointment wrap-up
Callie and Noah both described instances of how their gender did not match their sex and you document in their chart that they have marked and persistent gender dysphoria.
You note that Noah meets criteria for major depression and generalized anxiety disorder
They want to take some time to think about everything they heard about today
In addition to scheduling a follow-up appointment, what else would you do?

## Slide 34
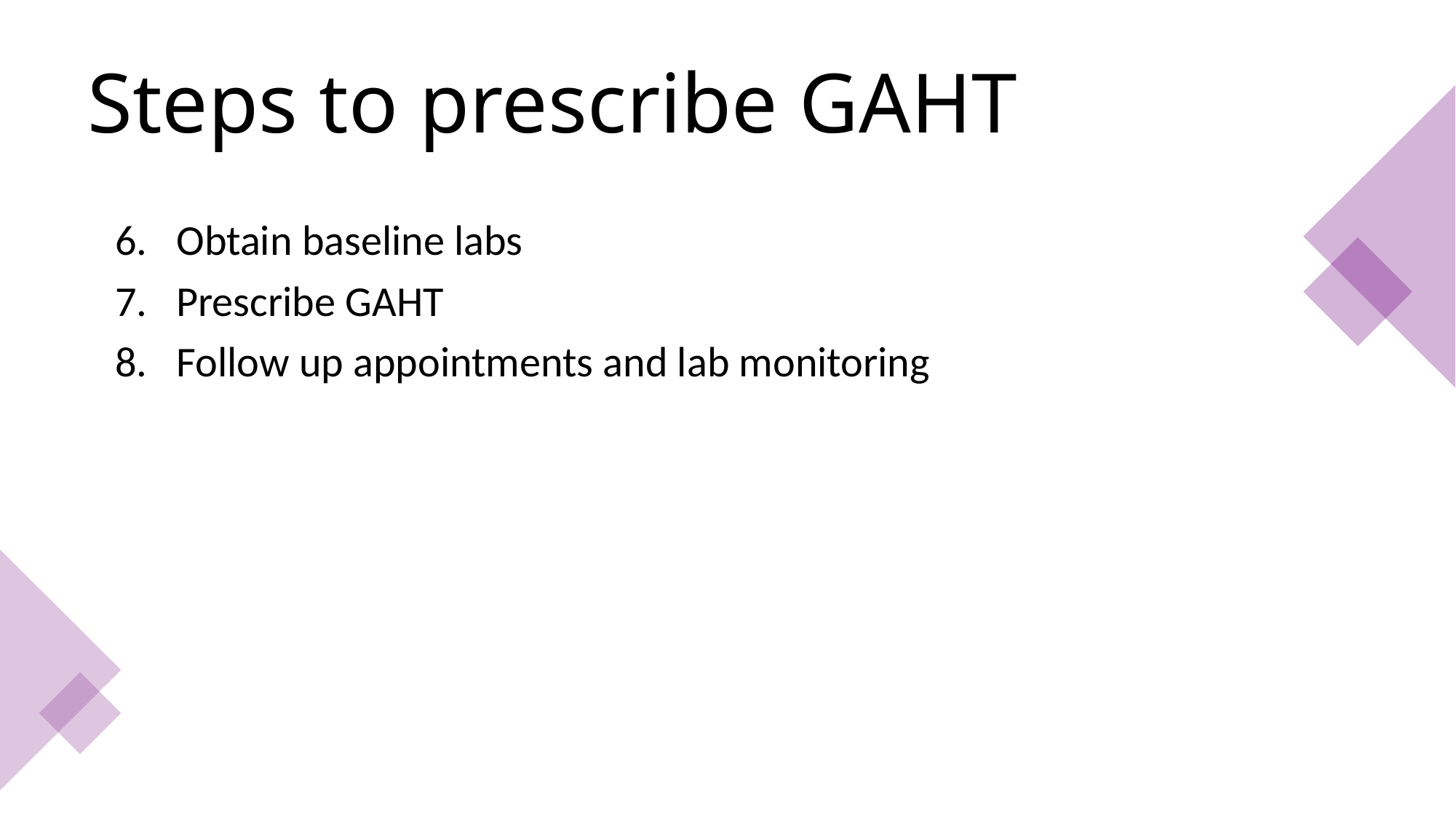

# Steps to prescribe GAHT
Obtain baseline labs
Prescribe GAHT
Follow up appointments and lab monitoring

## Slide 35
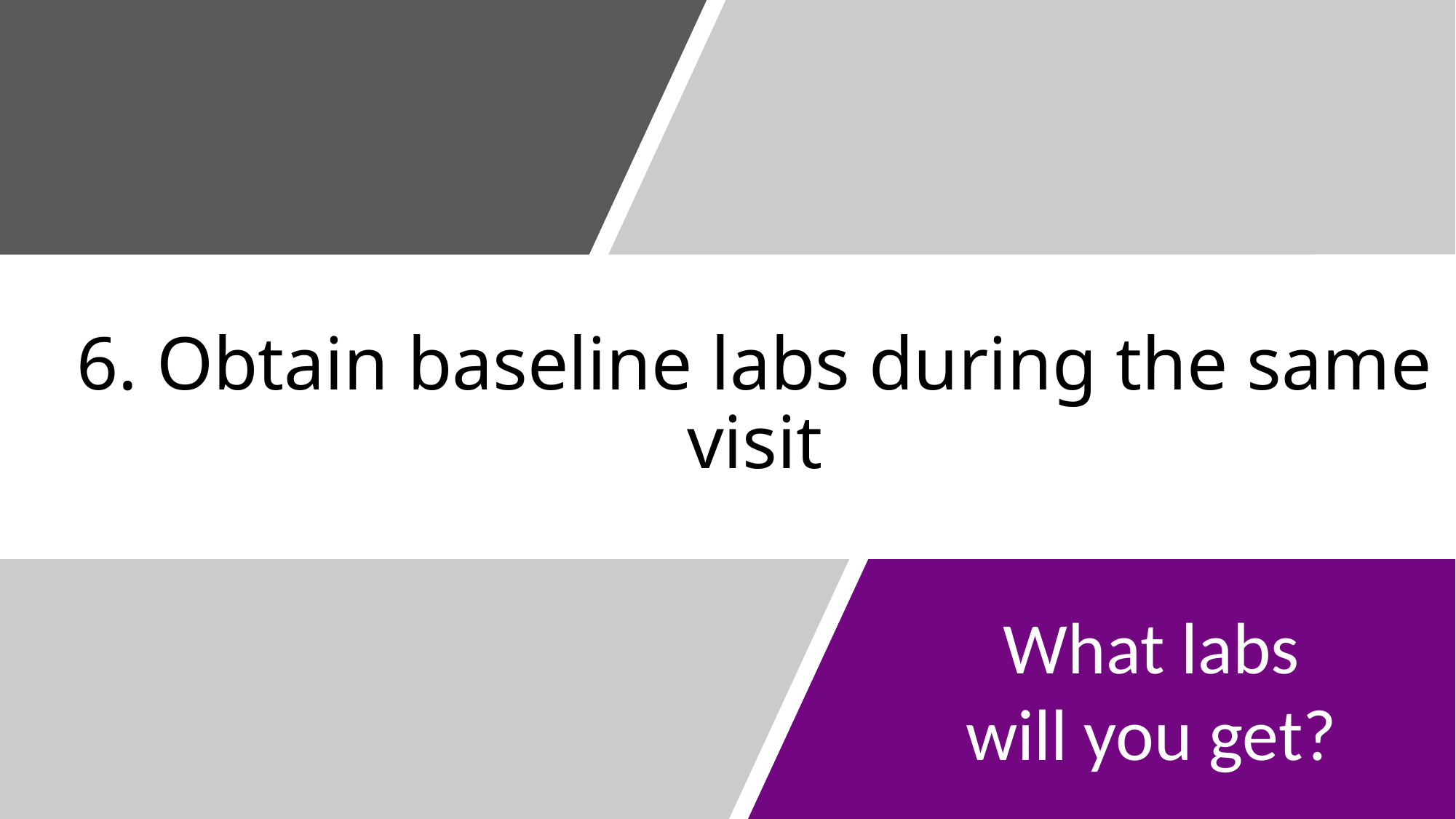

# 6. Obtain baseline labs during the same visit
What labs will you get?

## Slide 36
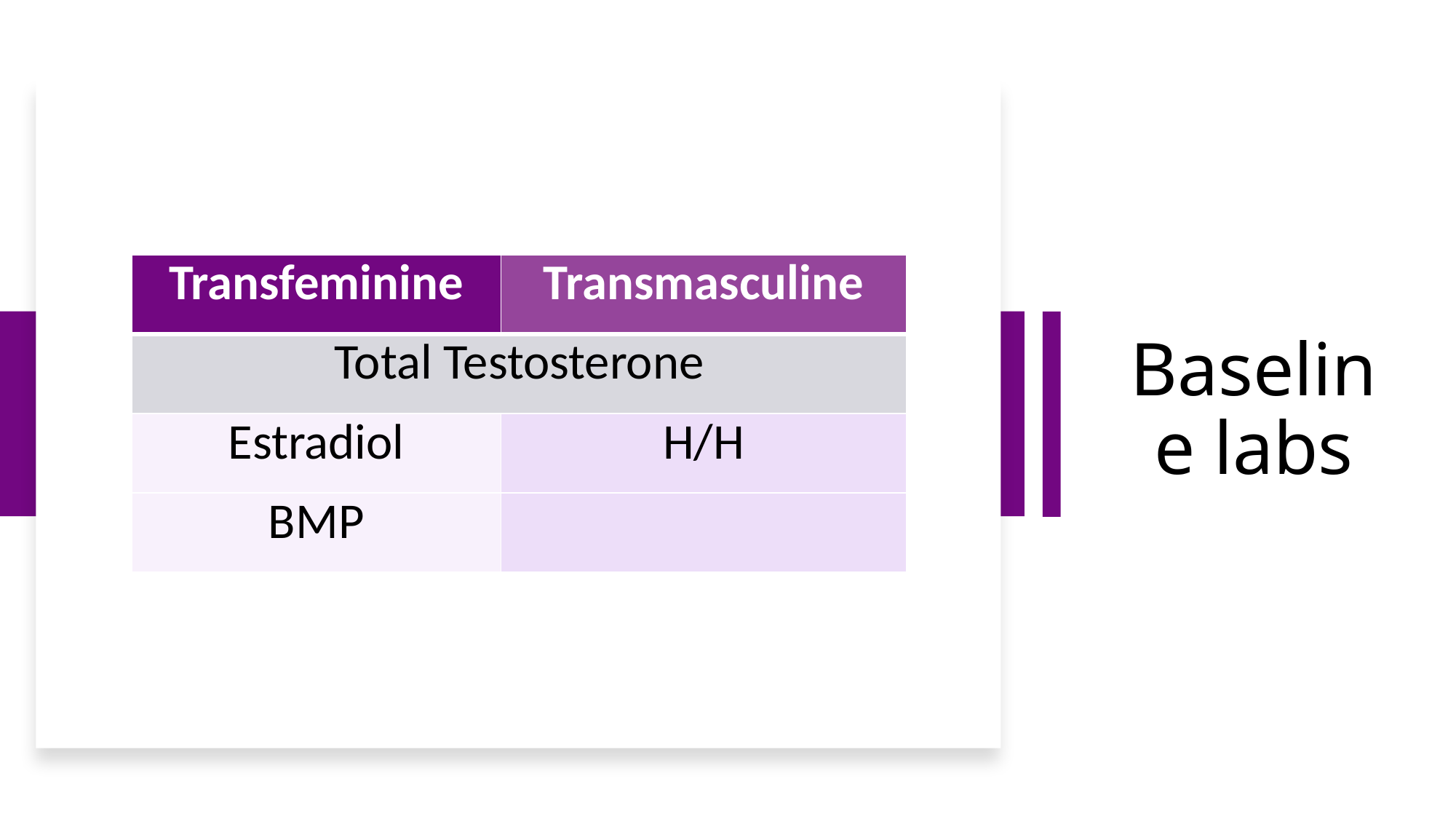

# Baseline labs
| Transfeminine | Transmasculine |
| --- | --- |
| Total Testosterone | |
| Estradiol | H/H |
| BMP | |

## Slide 37
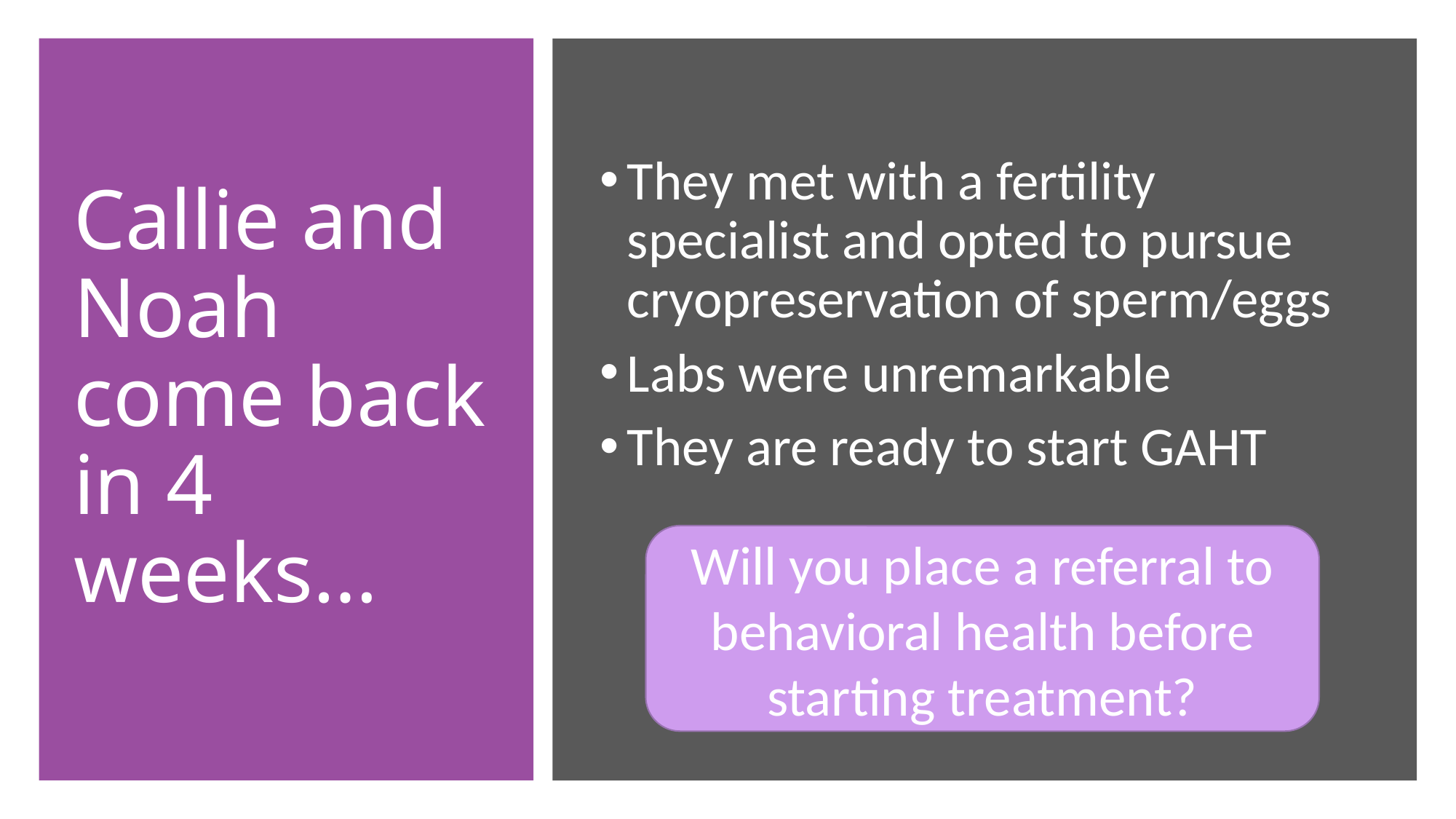

# Callie and Noah come back in 4 weeks…
They met with a fertility specialist and opted to pursue cryopreservation of sperm/eggs
Labs were unremarkable
They are ready to start GAHT
Will you place a referral to behavioral health before starting treatment?

## Slide 38
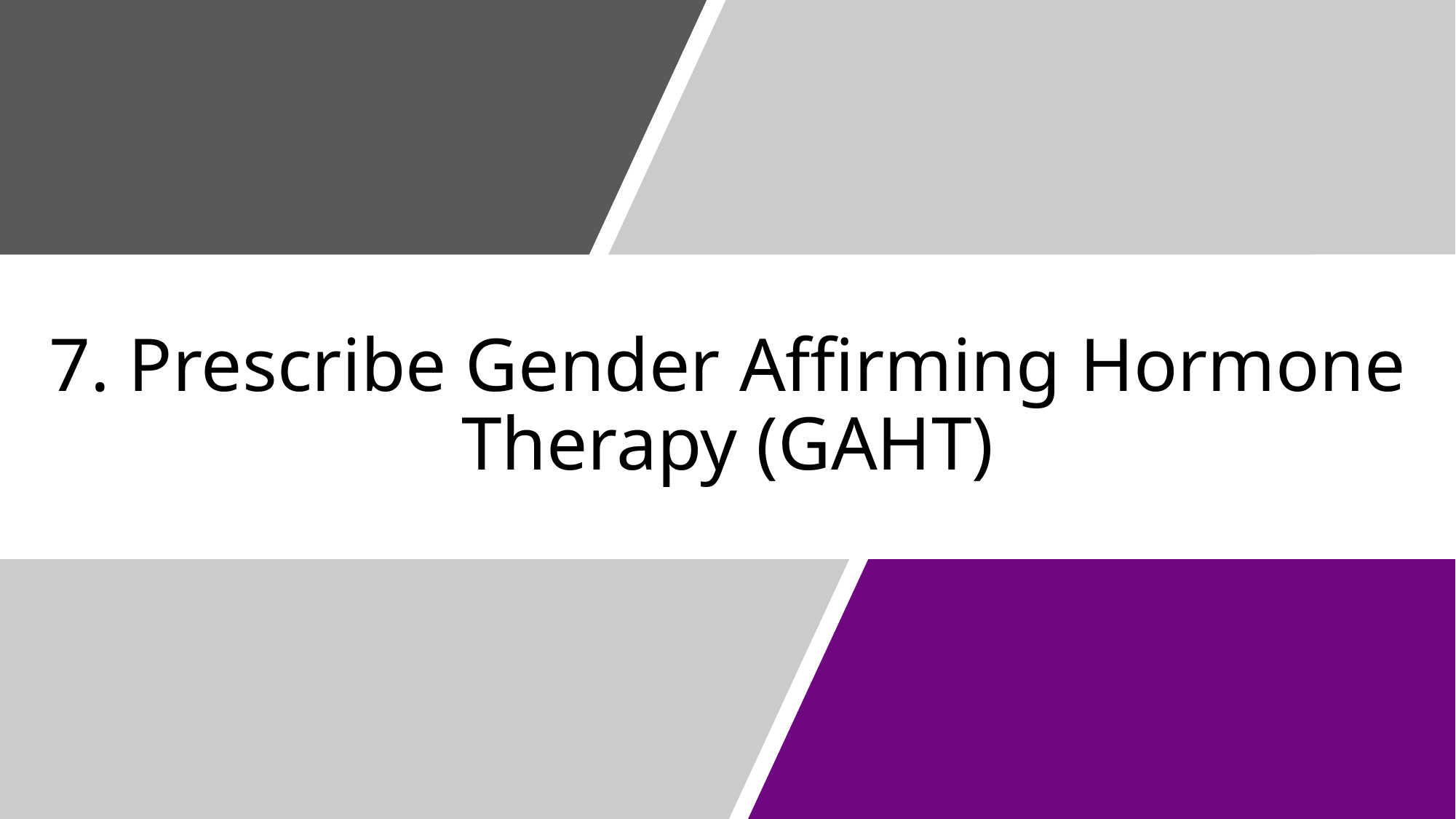

# 7. Prescribe Gender Affirming Hormone Therapy (GAHT)

## Slide 39
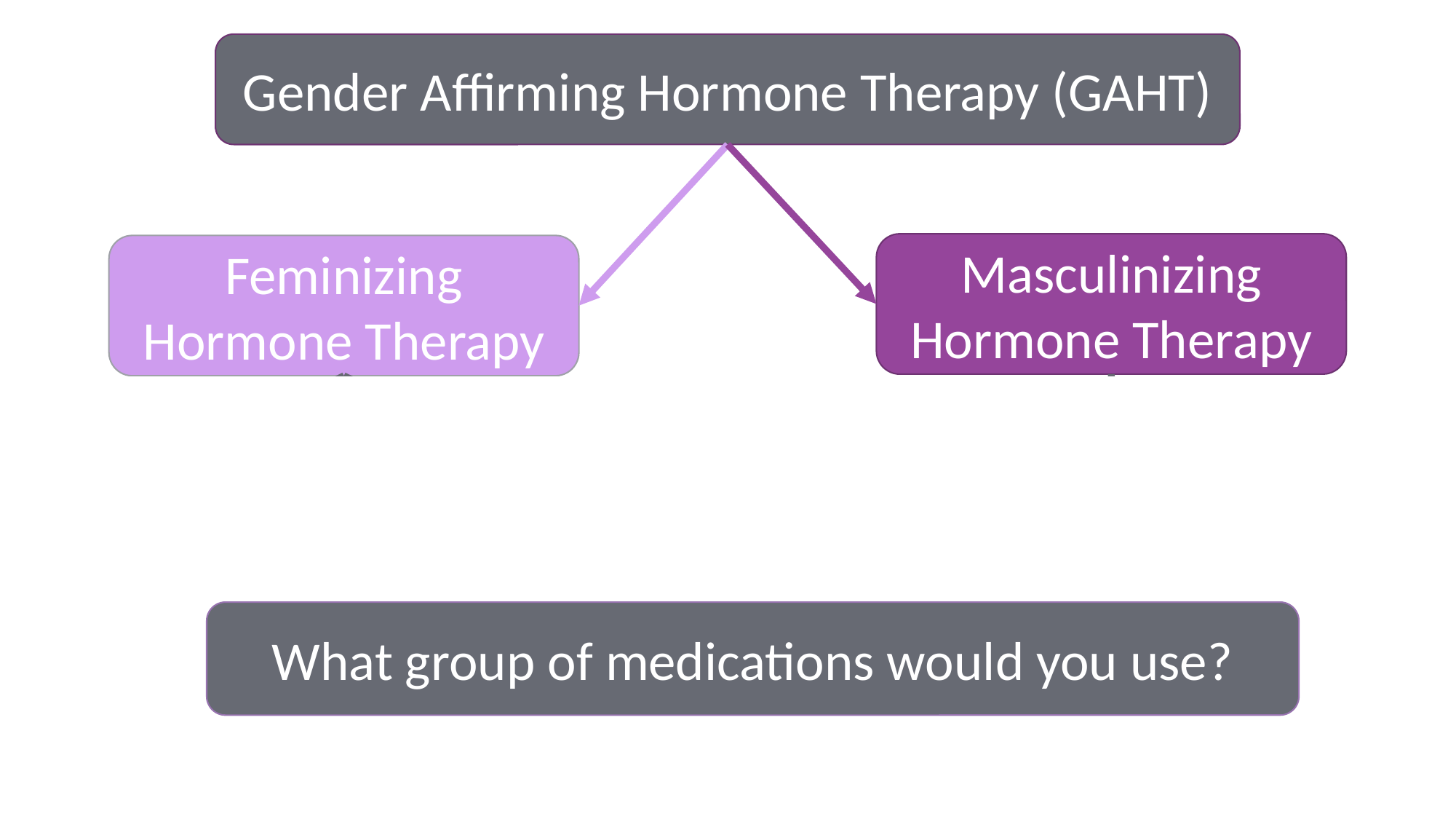

Gender Affirming Hormone Therapy (GAHT)
Masculinizing
Hormone Therapy
Feminizing
Hormone Therapy
Estrogen
Androgen Blocker
Testosterone
What group of medications would you use?
Estradiol
Spironolactone

## Slide 40
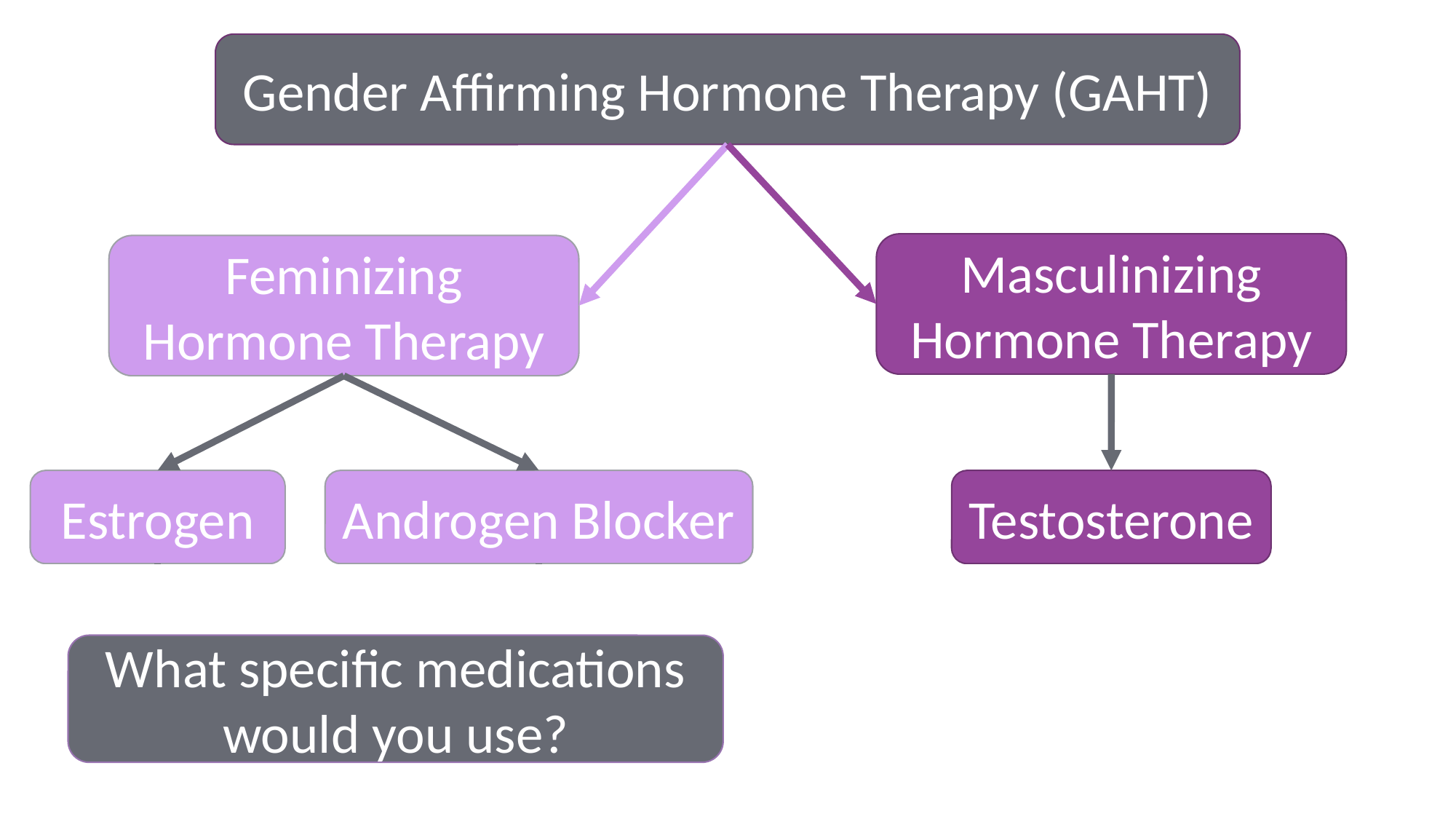

Gender Affirming Hormone Therapy (GAHT)
Masculinizing
Hormone Therapy
Feminizing
Hormone Therapy
Estrogen
Androgen Blocker
Testosterone
What specific medications would you use?
Estradiol
Spironolactone

## Slide 41
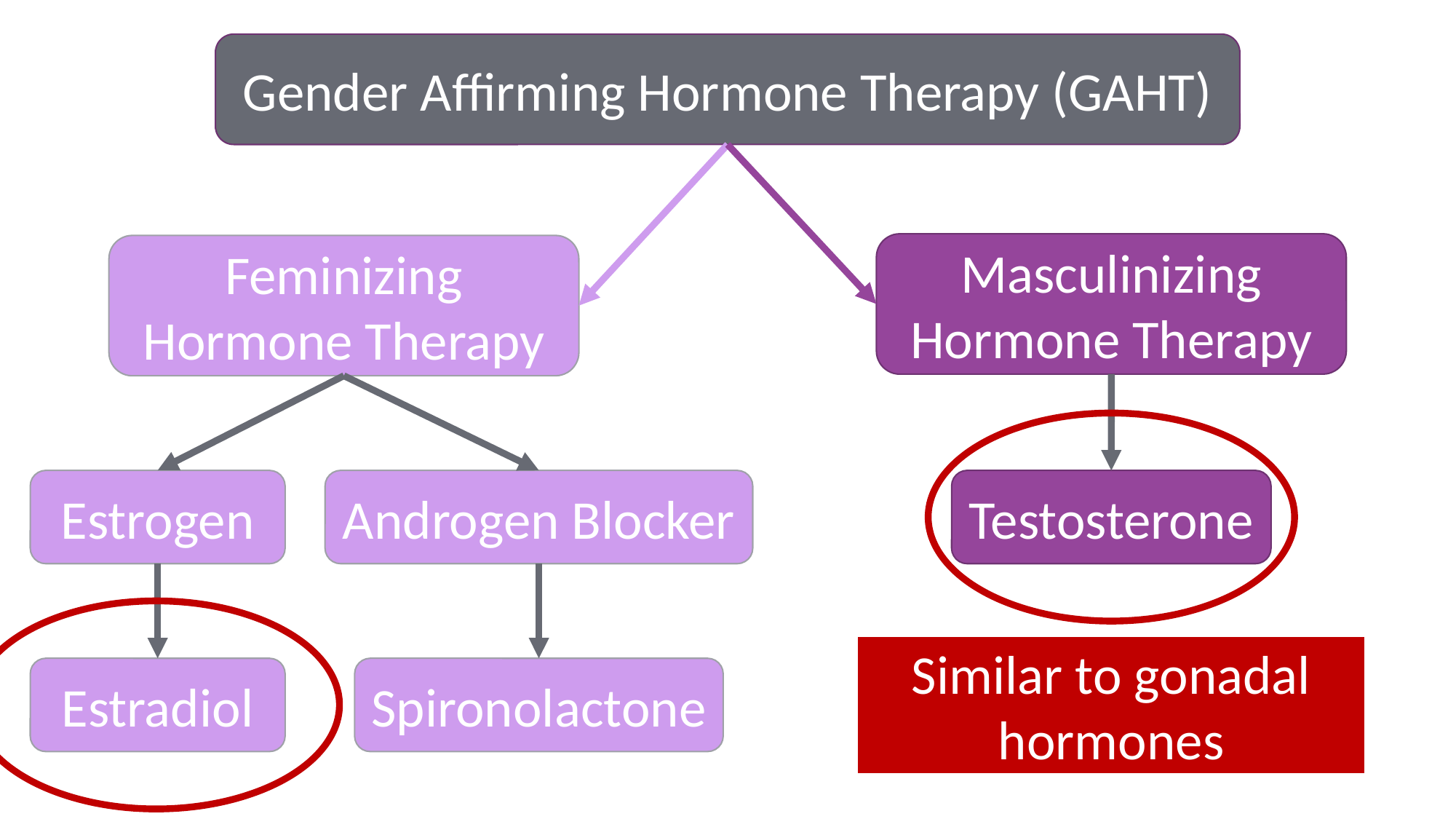

Gender Affirming Hormone Therapy (GAHT)
Masculinizing
Hormone Therapy
Feminizing
Hormone Therapy
Estrogen
Androgen Blocker
Testosterone
Similar to gonadal hormones
Estradiol
Spironolactone

## Slide 42
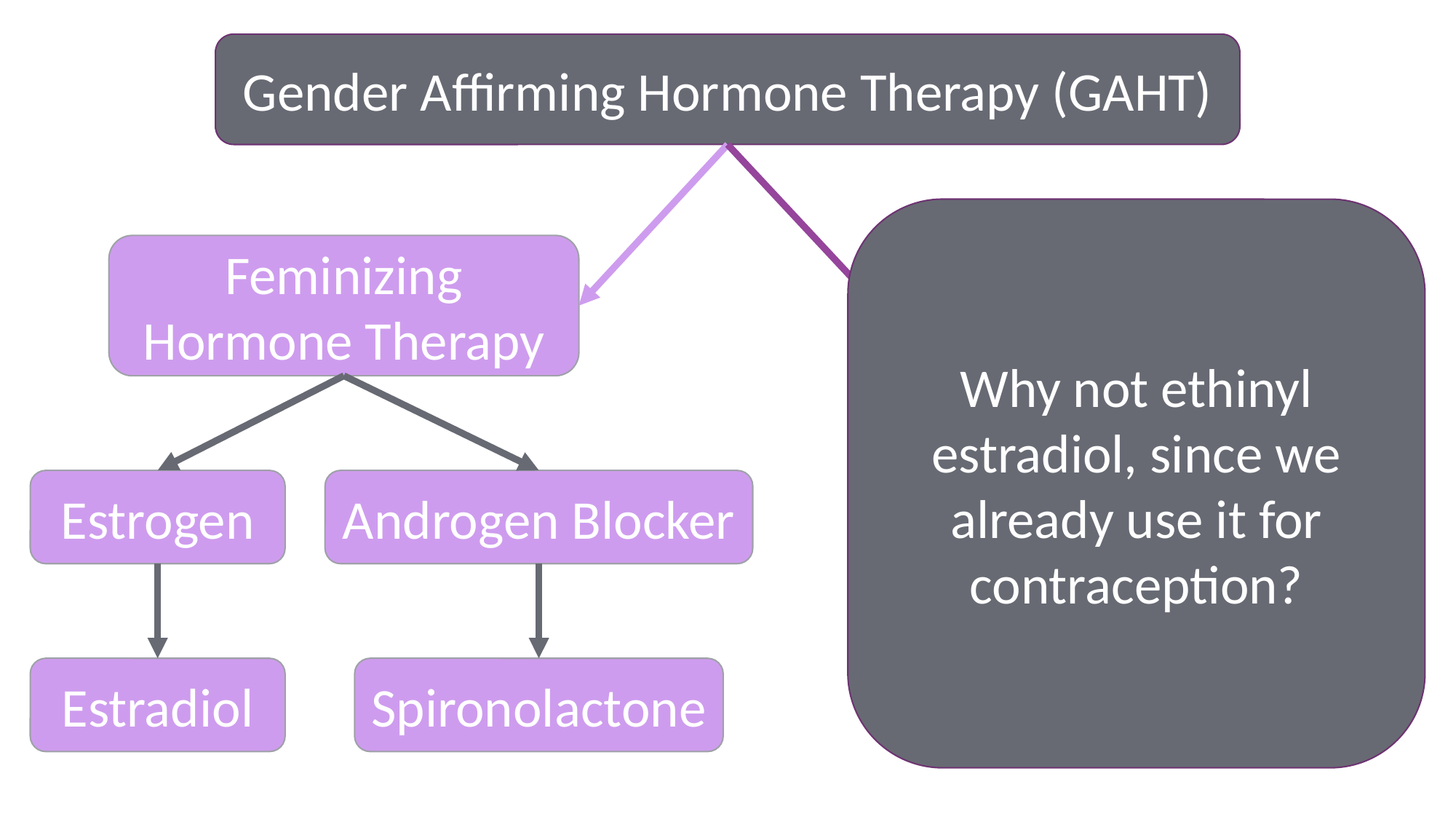

Gender Affirming Hormone Therapy (GAHT)
Why not ethinyl estradiol, since we already use it for contraception?
Masculinizing
Hormone Therapy
Feminizing
Hormone Therapy
Estrogen
Androgen Blocker
Testosterone
Estradiol
Spironolactone

## Slide 43
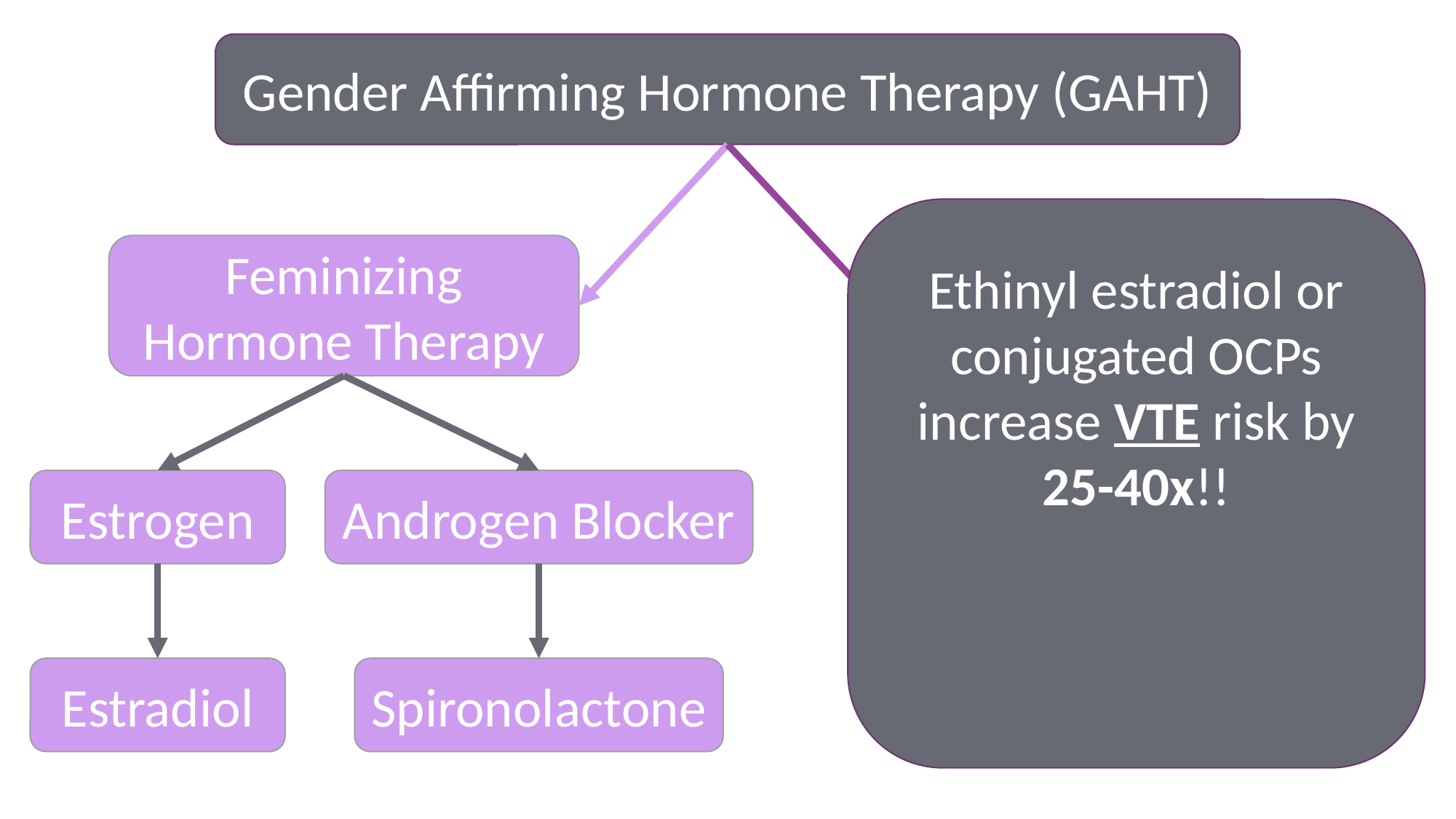

Gender Affirming Hormone Therapy (GAHT)
Ethinyl estradiol or conjugated OCPs increase VTE risk by 25-40x!!
Masculinizing
Hormone Therapy
Feminizing
Hormone Therapy
Estrogen
Androgen Blocker
Testosterone
Estradiol
Spironolactone

## Slide 44
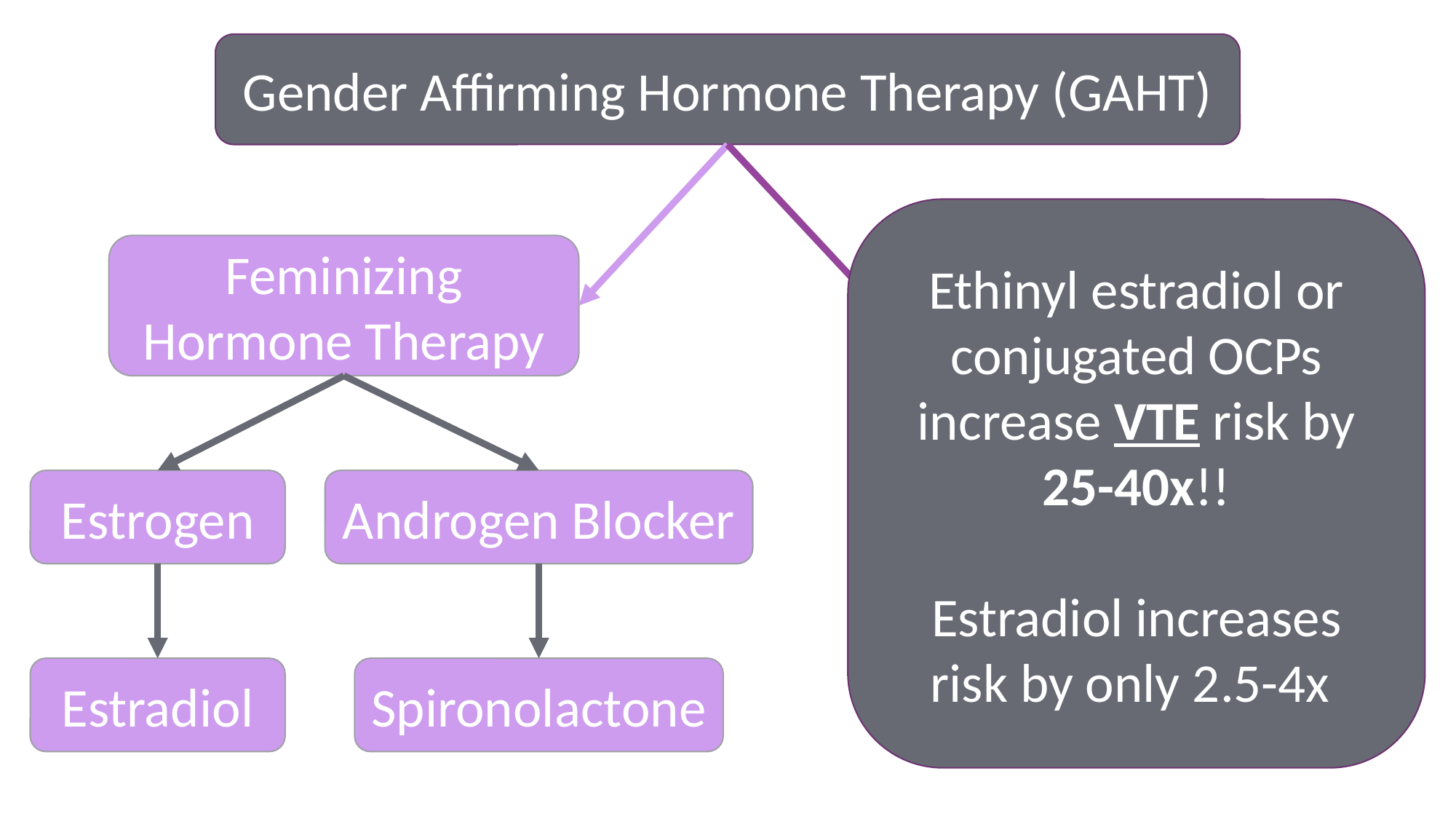

Gender Affirming Hormone Therapy (GAHT)
Ethinyl estradiol or conjugated OCPs increase VTE risk by 25-40x!!
Estradiol increases risk by only 2.5-4x
Masculinizing
Hormone Therapy
Feminizing
Hormone Therapy
Estrogen
Androgen Blocker
Testosterone
Estradiol
Spironolactone

## Slide 45
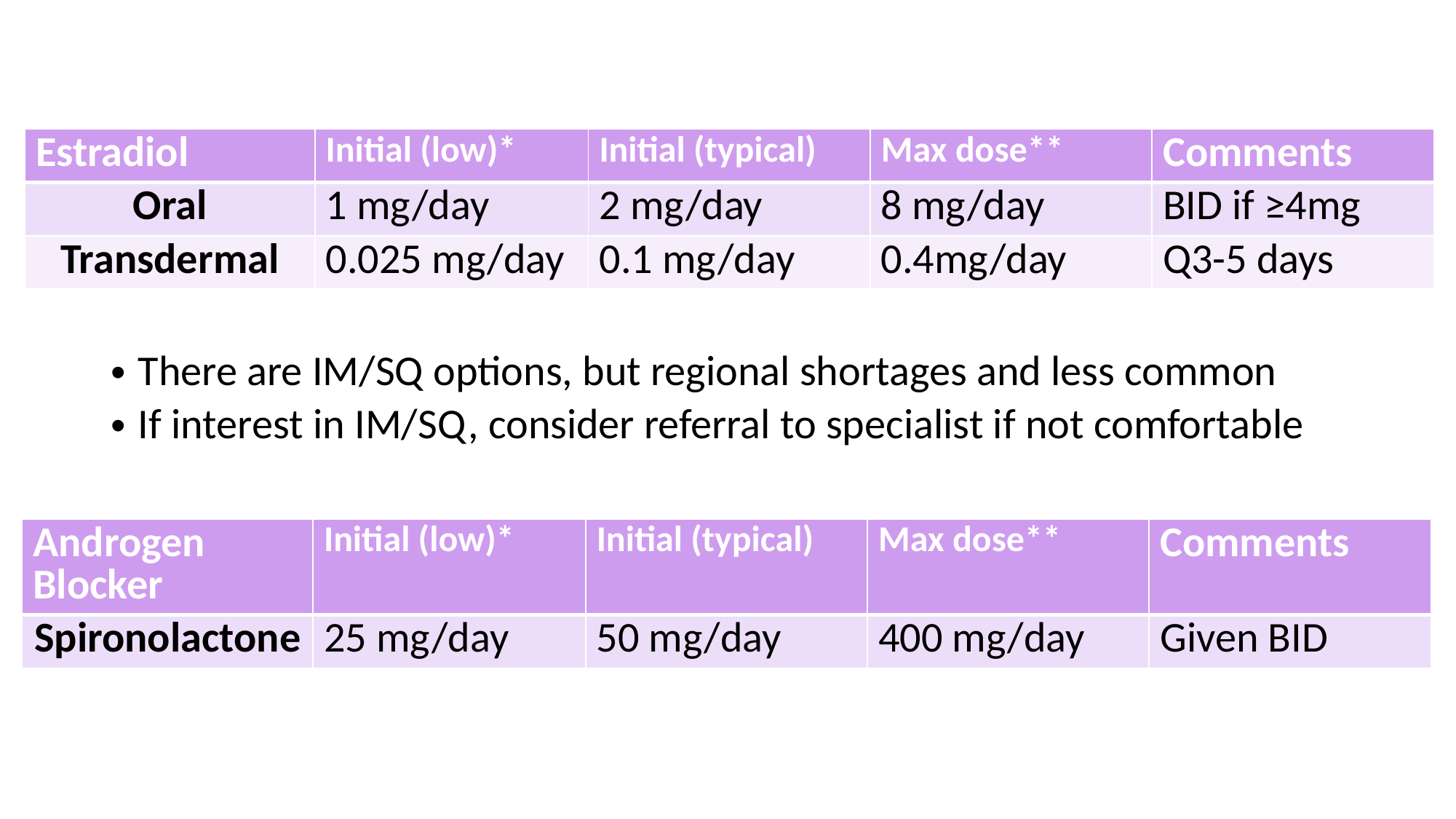

| Estradiol | Initial (low)\* | Initial (typical) | Max dose\*\* | Comments |
| --- | --- | --- | --- | --- |
| Oral | 1 mg/day | 2 mg/day | 8 mg/day | BID if ≥4mg |
| Transdermal | 0.025 mg/day | 0.1 mg/day | 0.4mg/day | Q3-5 days |
There are IM/SQ options, but regional shortages and less common
If interest in IM/SQ, consider referral to specialist if not comfortable
| Androgen Blocker | Initial (low)\* | Initial (typical) | Max dose\*\* | Comments |
| --- | --- | --- | --- | --- |
| Spironolactone | 25 mg/day | 50 mg/day | 400 mg/day | Given BID |

## Slide 46
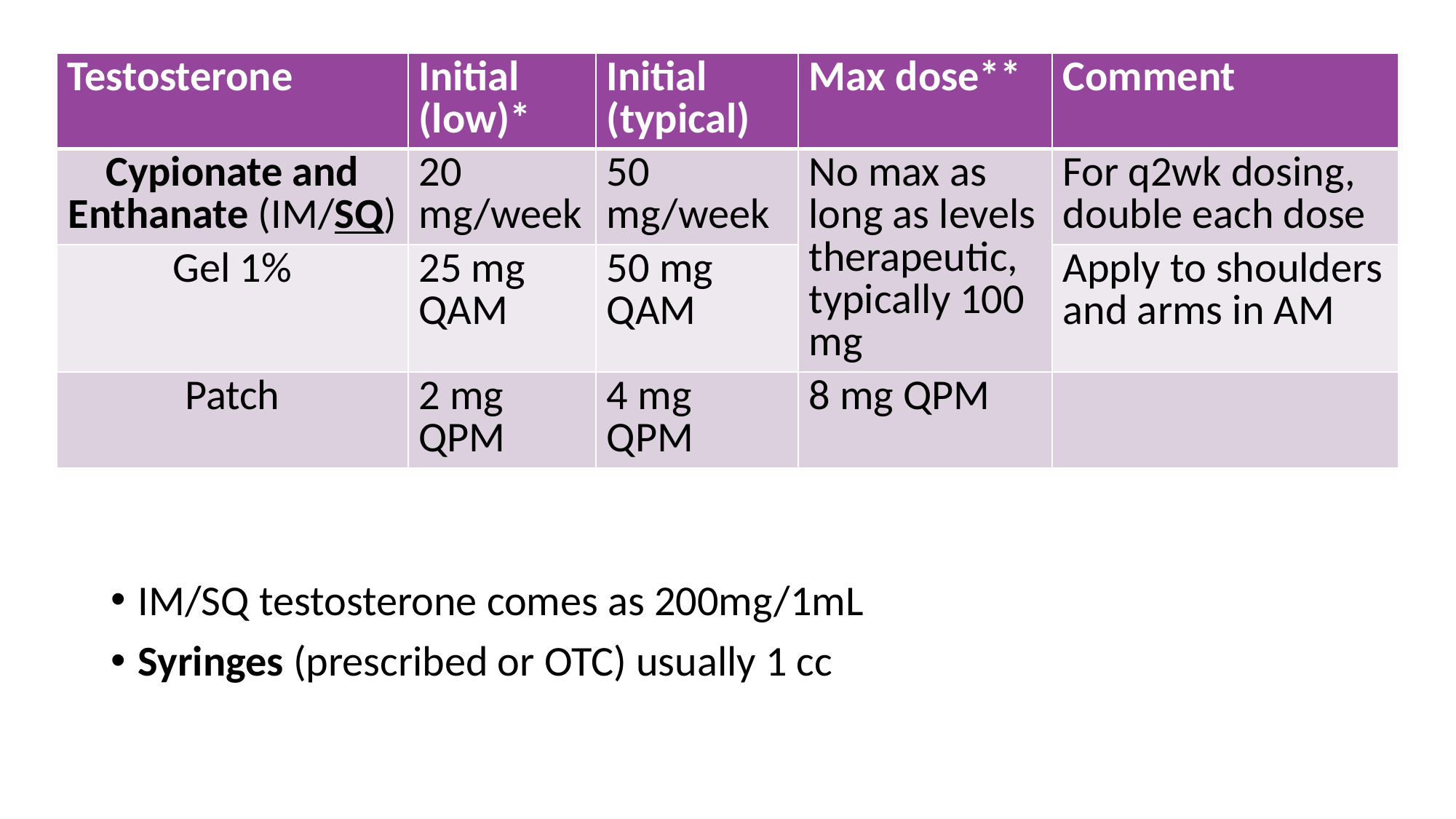

| Testosterone | Initial (low)\* | Initial (typical) | Max dose\*\* | Comment |
| --- | --- | --- | --- | --- |
| Cypionate and Enthanate (IM/SQ) | 20 mg/week | 50 mg/week | No max as long as levels therapeutic, typically 100 mg | For q2wk dosing, double each dose |
| Gel 1% | 25 mg QAM | 50 mg QAM | | Apply to shoulders and arms in AM |
| Patch | 2 mg QPM | 4 mg QPM | 8 mg QPM | |
IM/SQ testosterone comes as 200mg/1mL
Syringes (prescribed or OTC) usually 1 cc

## Slide 47
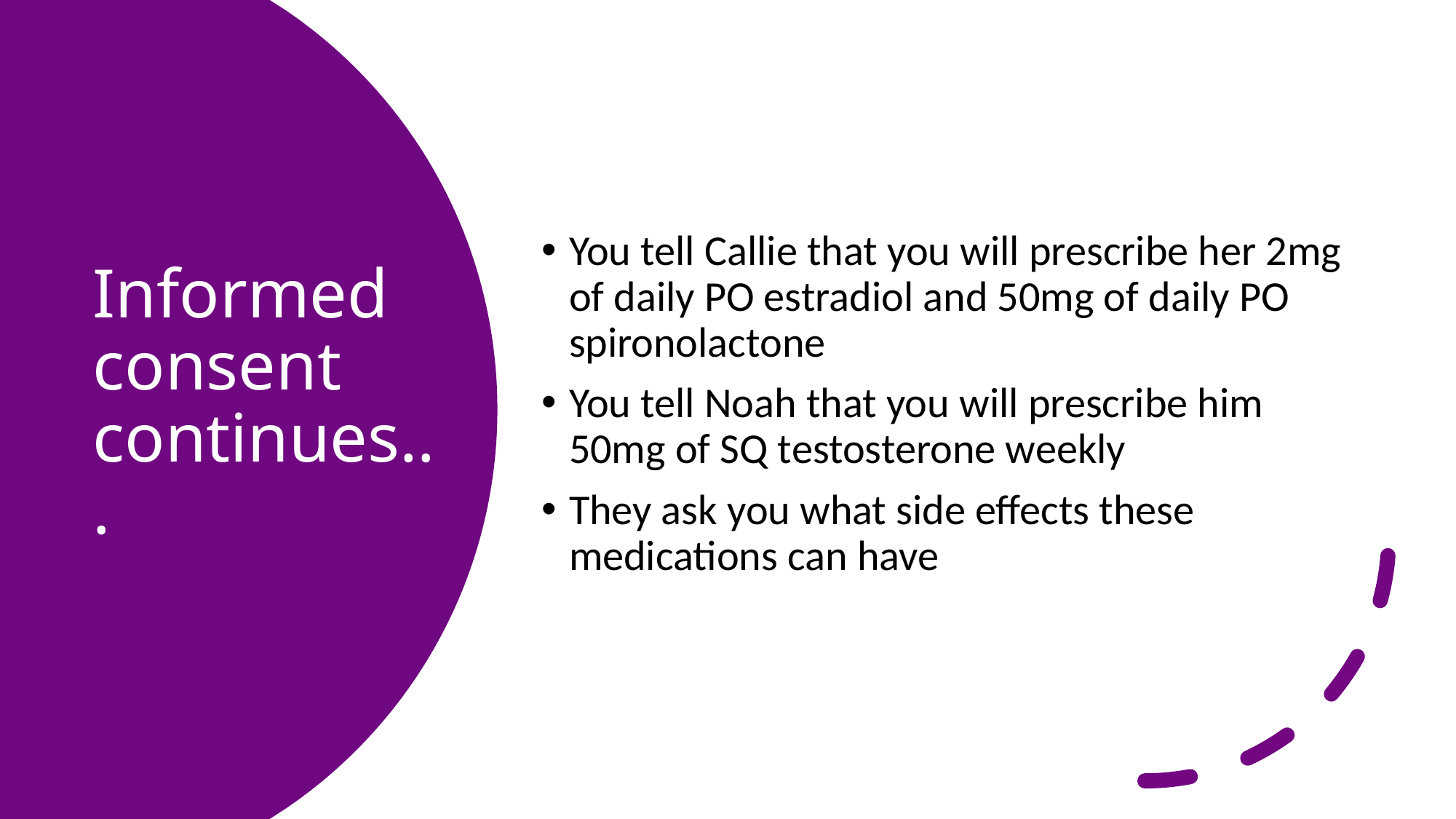

You tell Callie that you will prescribe her 2mg of daily PO estradiol and 50mg of daily PO spironolactone
You tell Noah that you will prescribe him 50mg of SQ testosterone weekly
They ask you what side effects these medications can have
# Informed consent continues...

## Slide 48
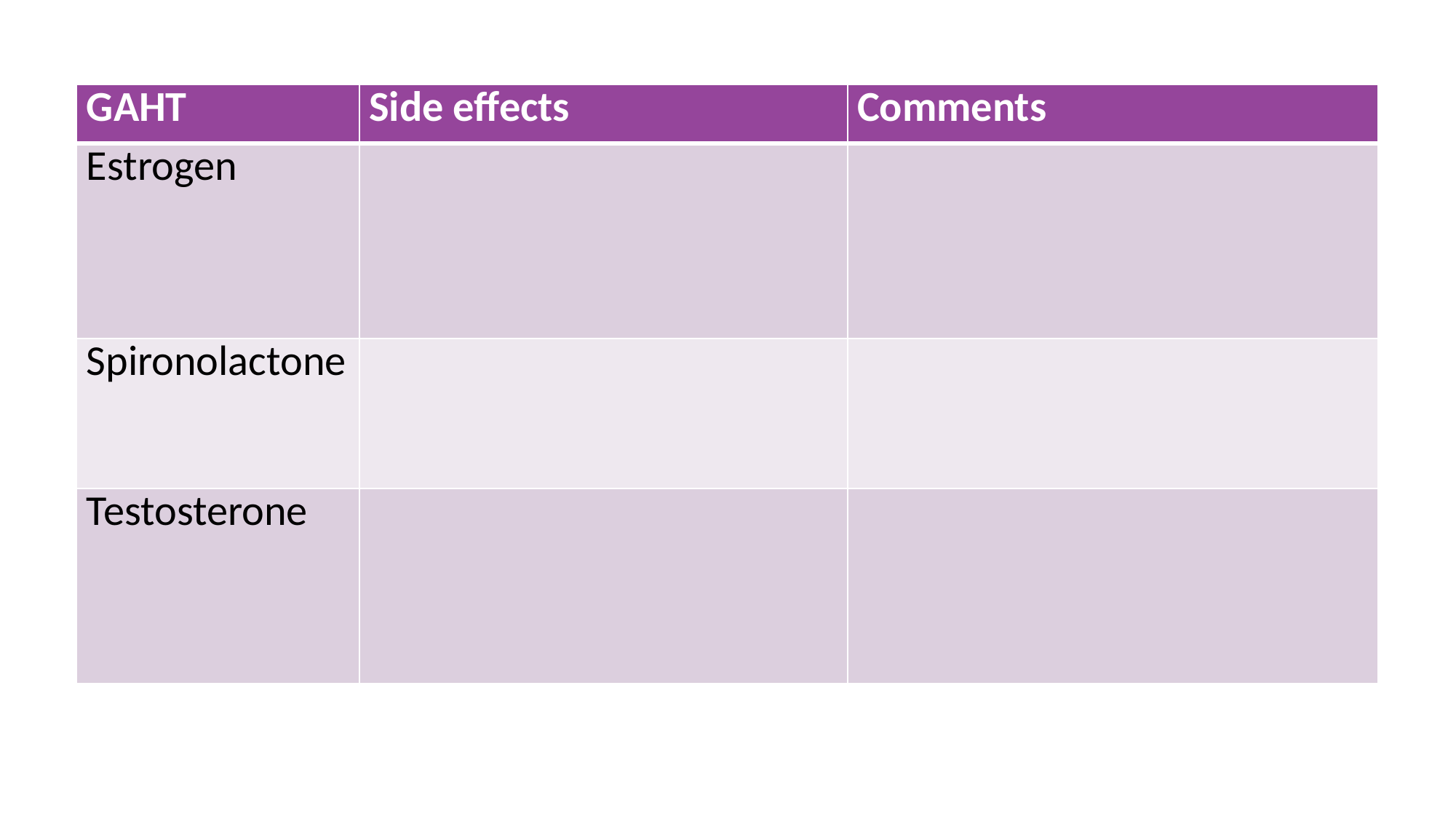

| GAHT | Side effects | Comments |
| --- | --- | --- |
| Estrogen | | |
| Spironolactone | | |
| Testosterone | | |

## Slide 49
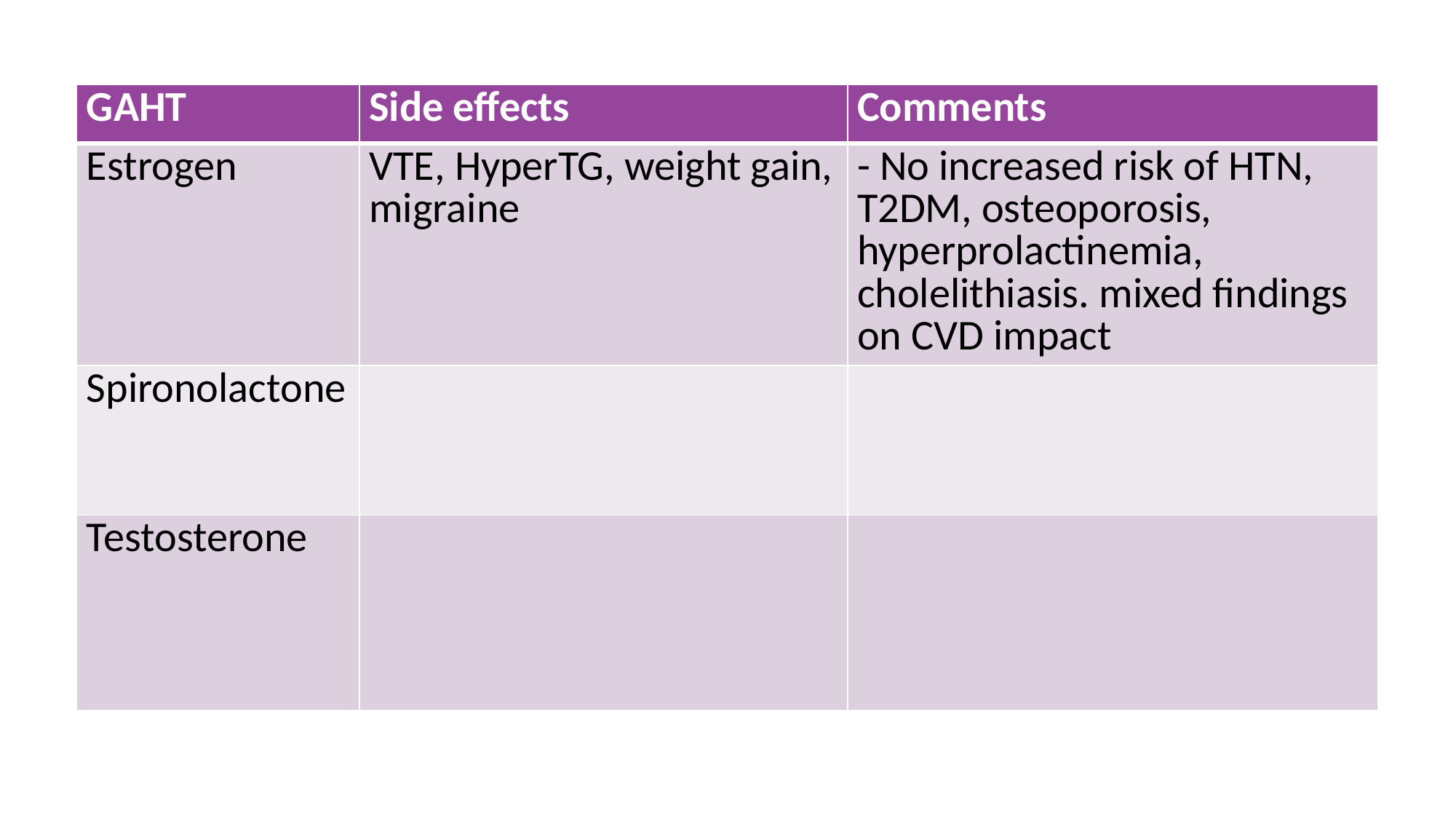

| GAHT | Side effects | Comments |
| --- | --- | --- |
| Estrogen | VTE, HyperTG, weight gain, migraine | - No increased risk of HTN, T2DM, osteoporosis, hyperprolactinemia, cholelithiasis. mixed findings on CVD impact |
| Spironolactone | | |
| Testosterone | | |

## Slide 50
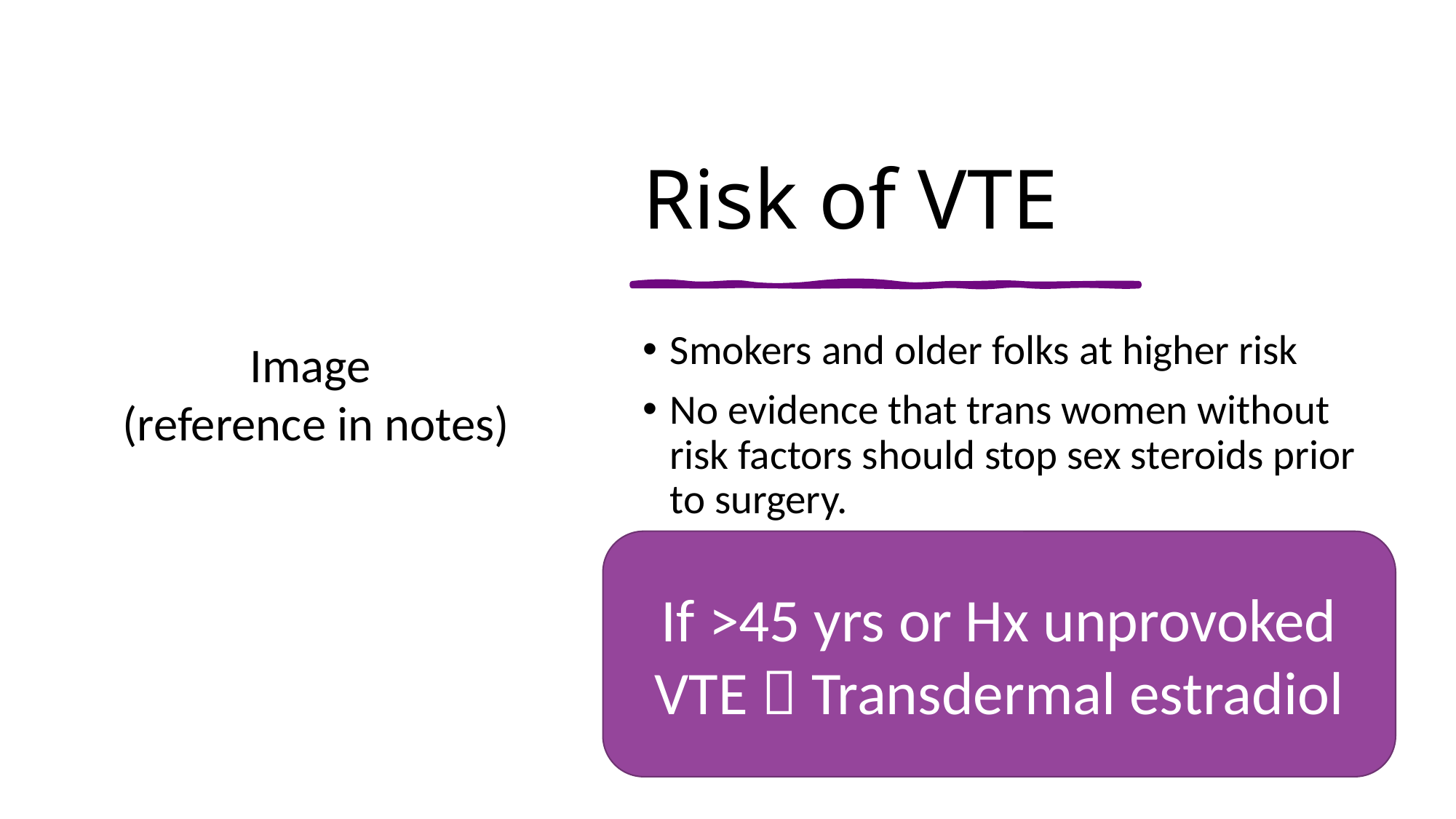

# Risk of VTE
Smokers and older folks at higher risk
No evidence that trans women without risk factors should stop sex steroids prior to surgery.
If high risk: Transdermal estradiol (no increased risk) and change modifiable risk factors (e.g. smoking)
Image
(reference in notes)
If >45 yrs or Hx unprovoked VTE  Transdermal estradiol

## Slide 51
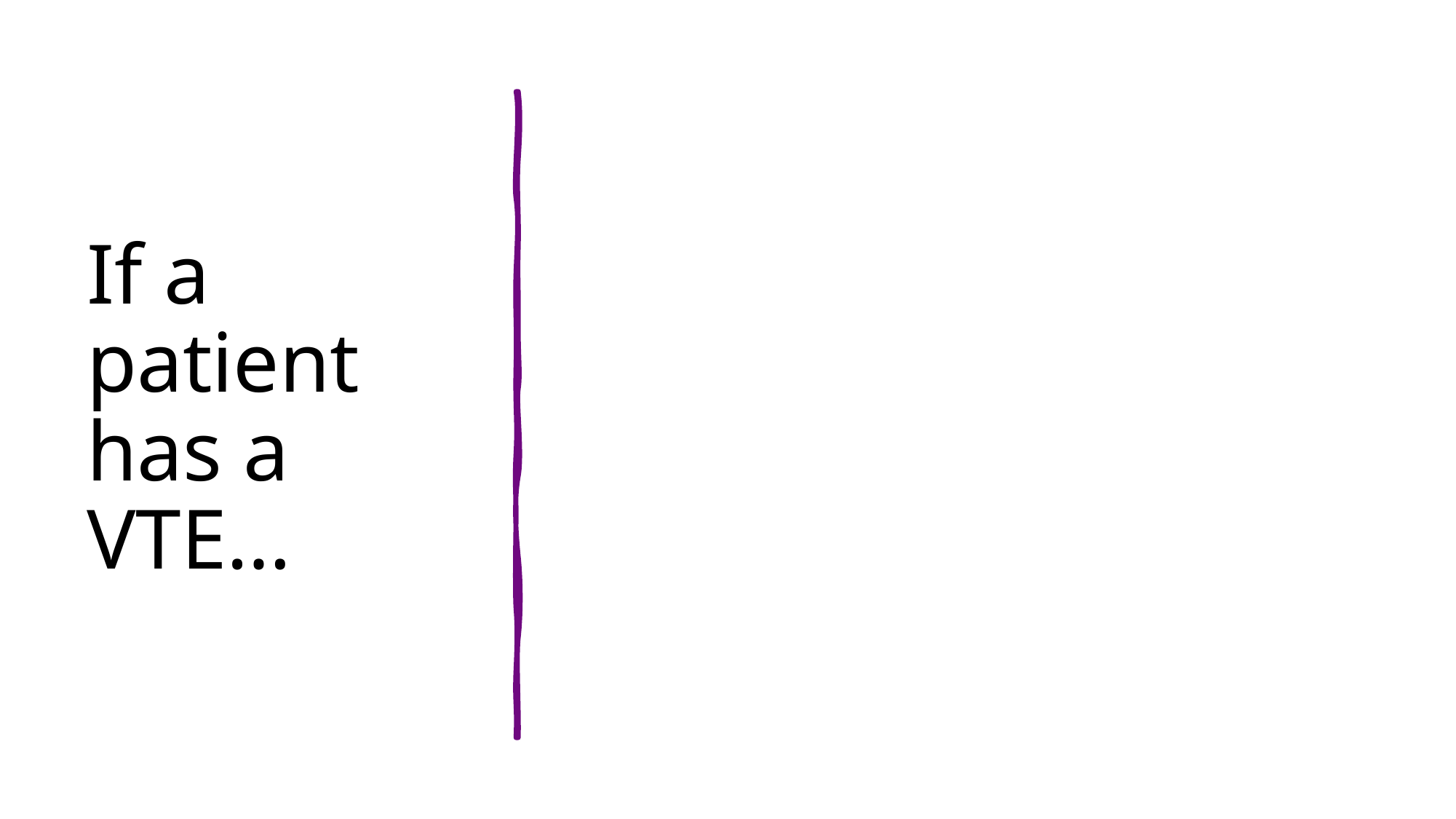

# If a patient has a VTE…

## Slide 52
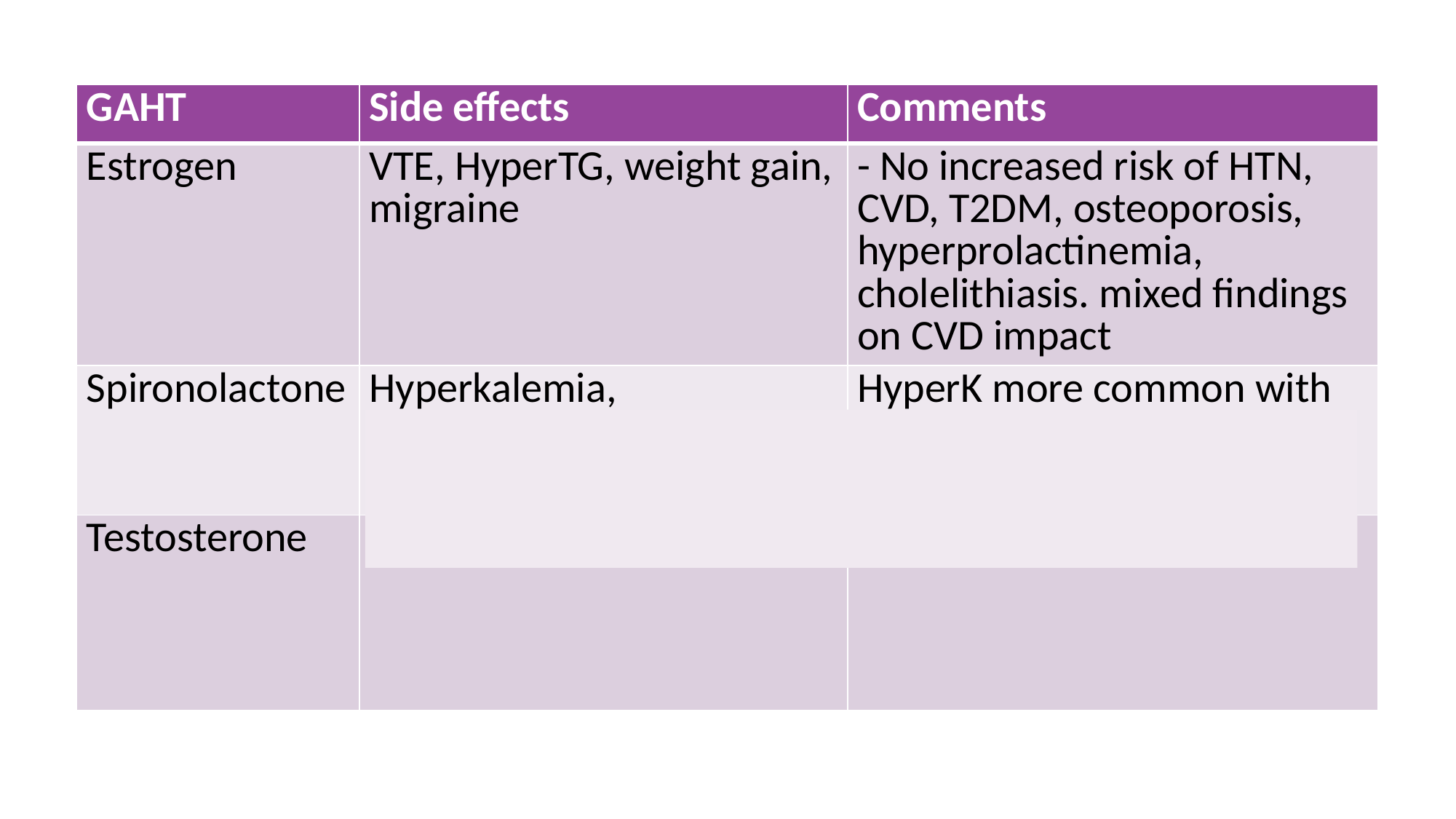

| GAHT | Side effects | Comments |
| --- | --- | --- |
| Estrogen | VTE, HyperTG, weight gain, migraine | - No increased risk of HTN, CVD, T2DM, osteoporosis, hyperprolactinemia, cholelithiasis. mixed findings on CVD impact |
| Spironolactone | Hyperkalemia, hypotension, frequency, polyuria, polydipsia | HyperK more common with other meds. It is uncommon and transient |
| Testosterone | | |

## Slide 53
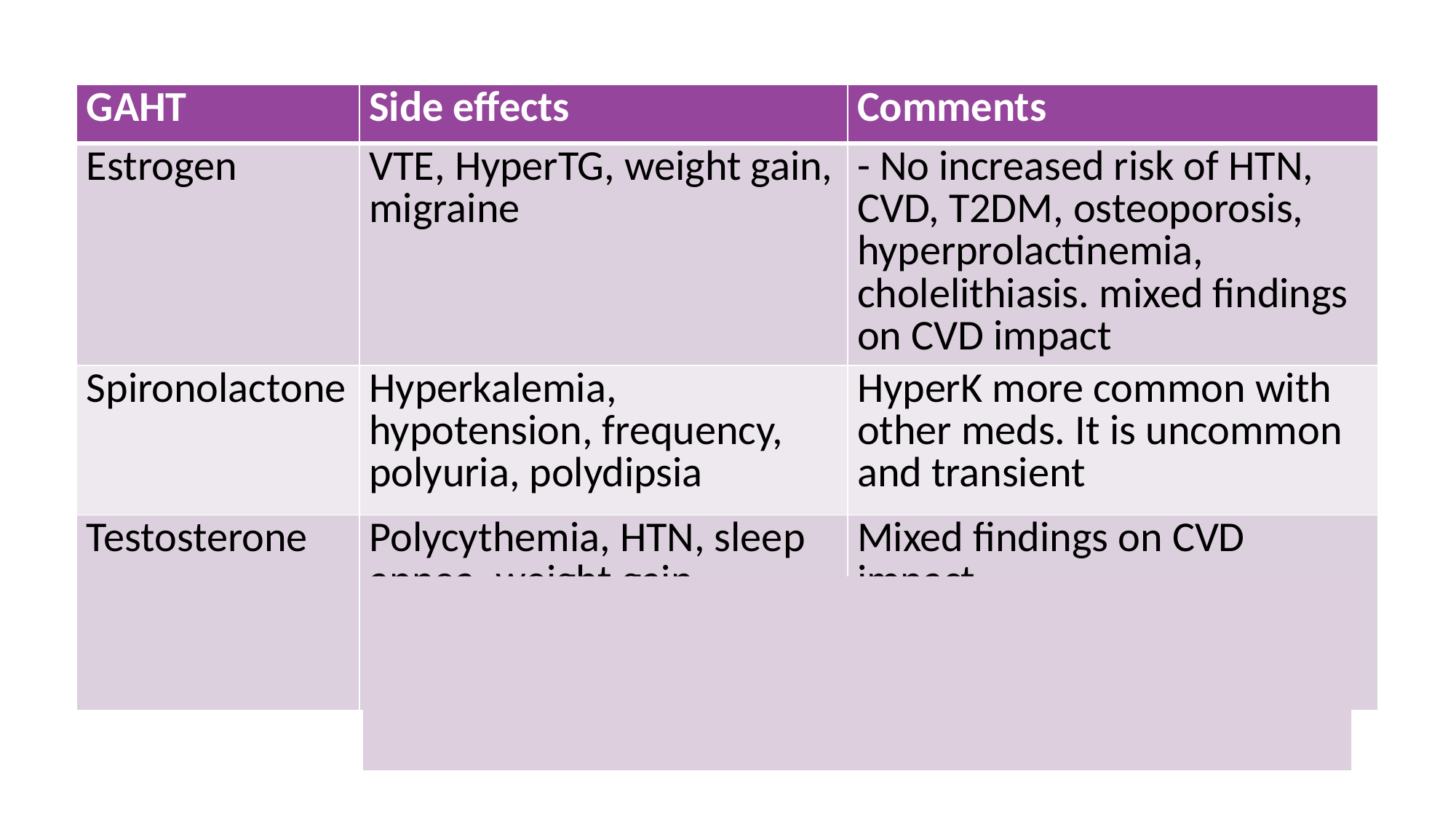

| GAHT | Side effects | Comments |
| --- | --- | --- |
| Estrogen | VTE, HyperTG, weight gain, migraine | - No increased risk of HTN, CVD, T2DM, osteoporosis, hyperprolactinemia, cholelithiasis. mixed findings on CVD impact |
| Spironolactone | Hyperkalemia, hypotension, frequency, polyuria, polydipsia | HyperK more common with other meds. It is uncommon and transient |
| Testosterone | Polycythemia, HTN, sleep apnea, weight gain, increased LDL, decreased HDL | Mixed findings on CVD impact |

## Slide 54
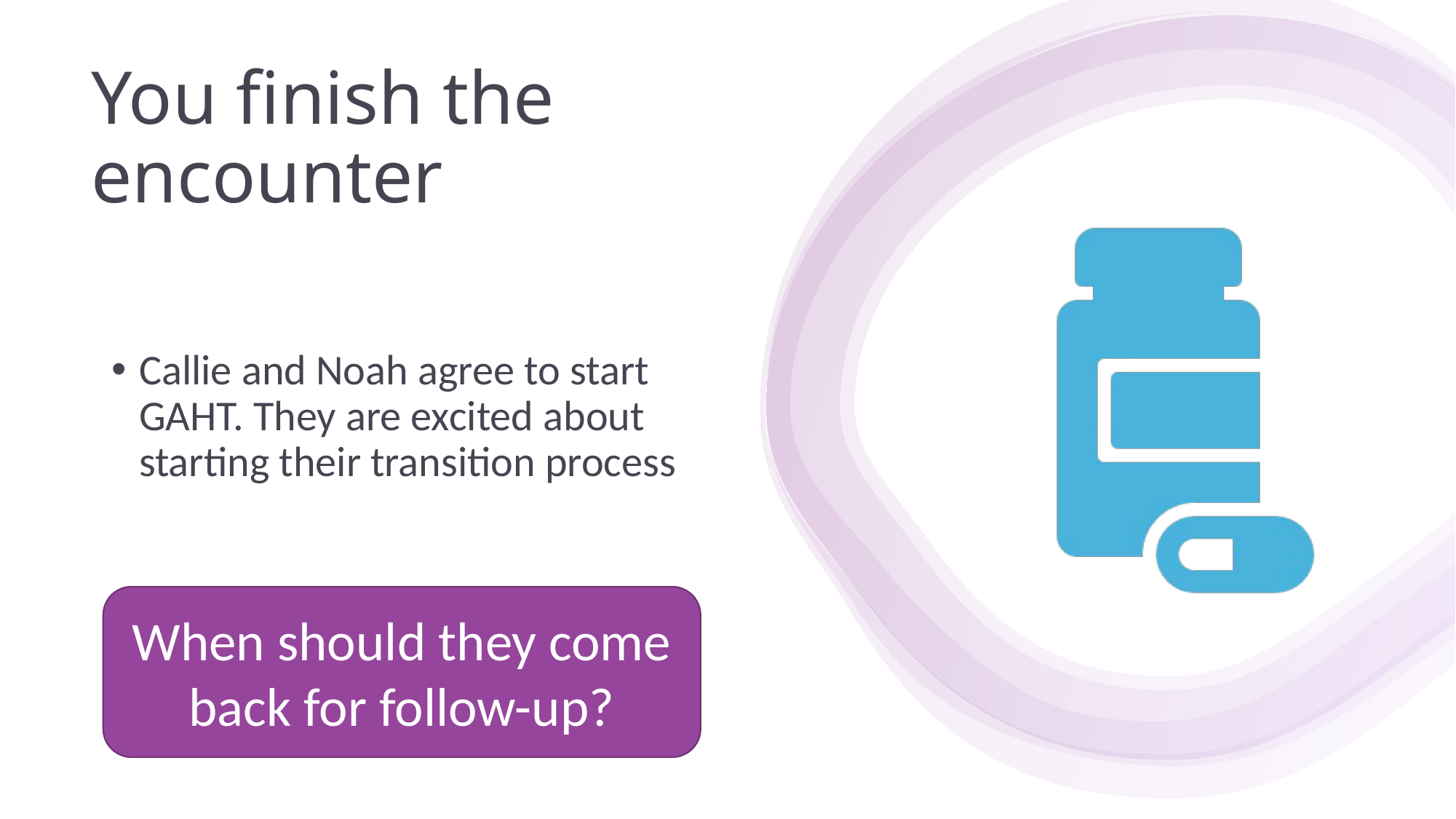

# You finish the encounter
Callie and Noah agree to start GAHT. They are excited about starting their transition process
When should they come back for follow-up?

## Slide 55
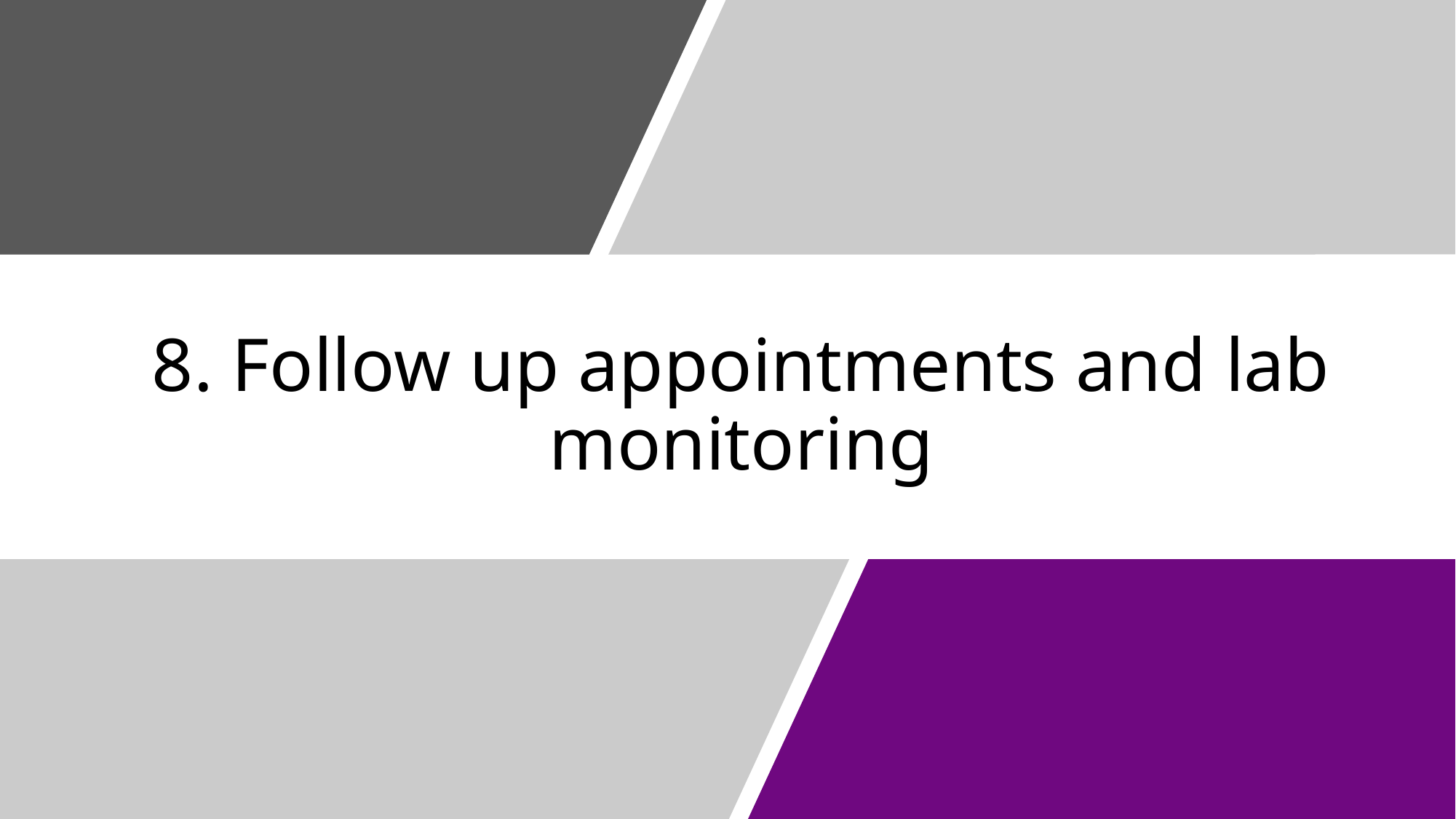

# 8. Follow up appointments and lab monitoring

## Slide 56
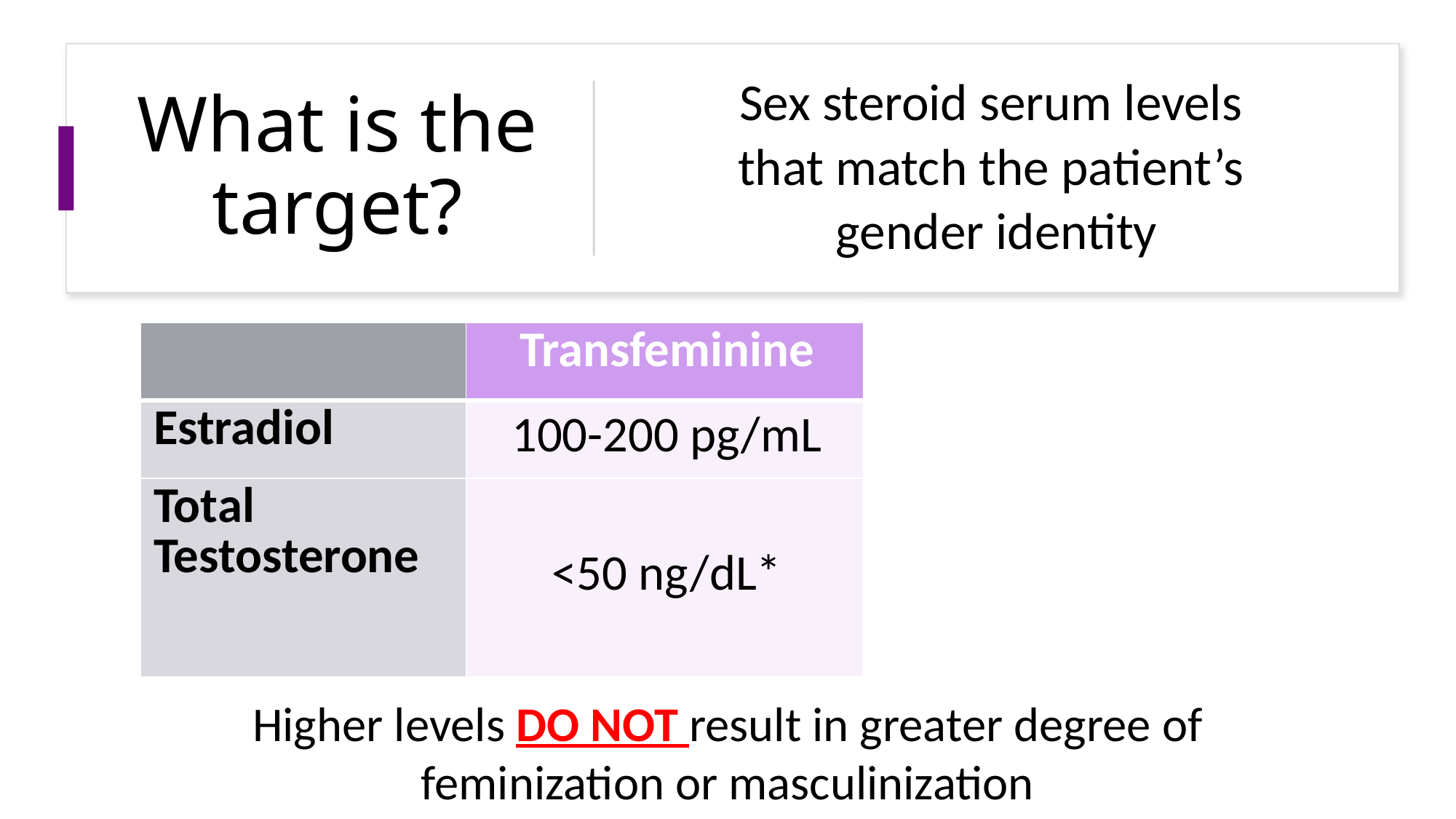

# What is the target?
Sex steroid serum levels
that match the patient’s
gender identity
| | Transfeminine | Transmasculine |
| --- | --- | --- |
| Estradiol | 100-200 pg/mL | Cessation of menses |
| Total Testosterone | <50 ng/dL\* | 400-700 ng/dL (midway between injections) |
Higher levels DO NOT result in greater degree of
feminization or masculinization

## Slide 57
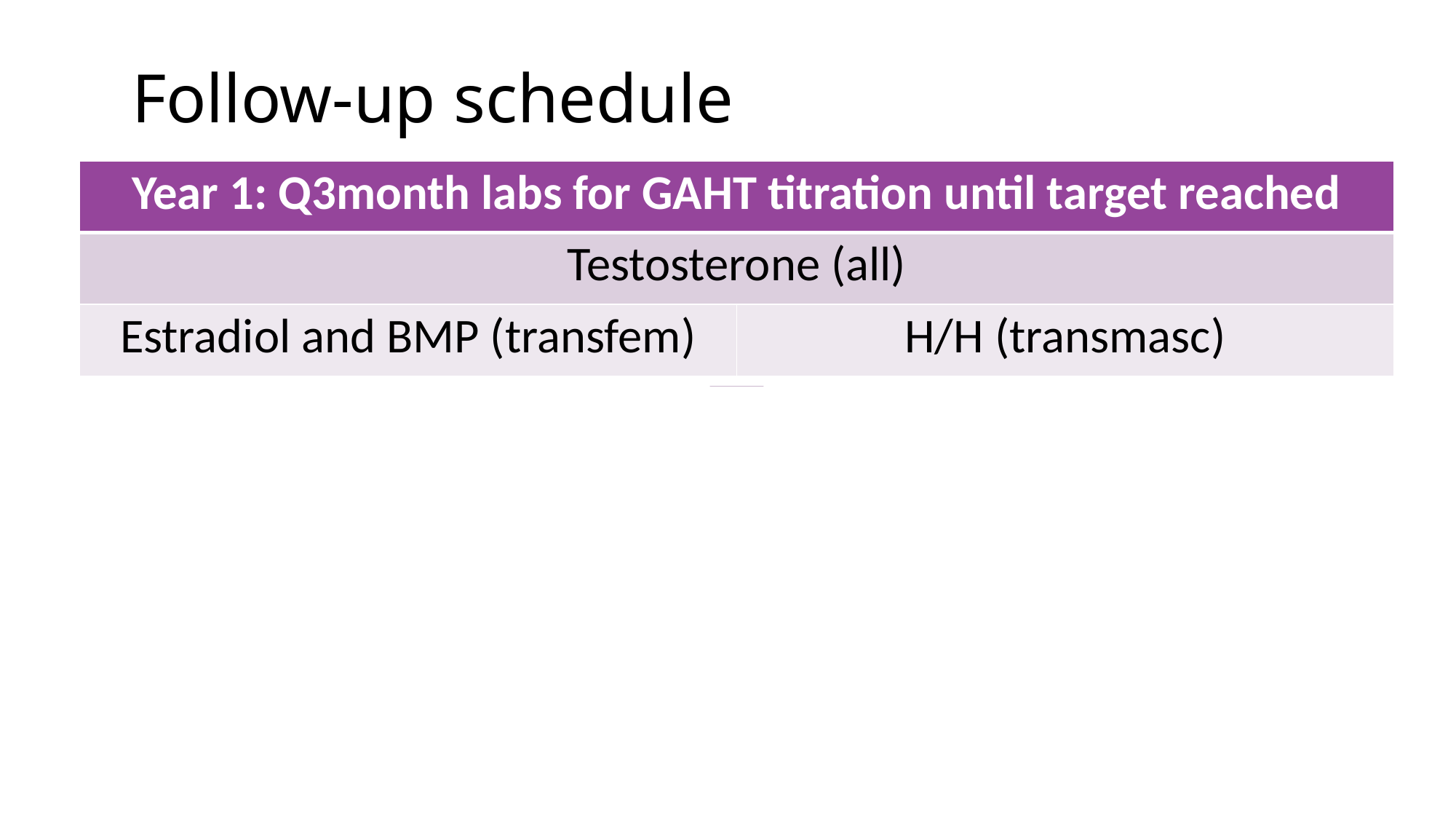

Follow-up schedule
| Year 1: Q3month labs for GAHT titration until target reached | |
| --- | --- |
| Testosterone (all) | |
| Estradiol and BMP (transfem) | H/H (transmasc) |
| Once target levels reached  yearly visits | | |
| --- | --- | --- |
| Testosterone (all) | | |
| Estradiol and BMP (transfem) | H/H (transmasc) | Other labs PRN |

## Slide 58
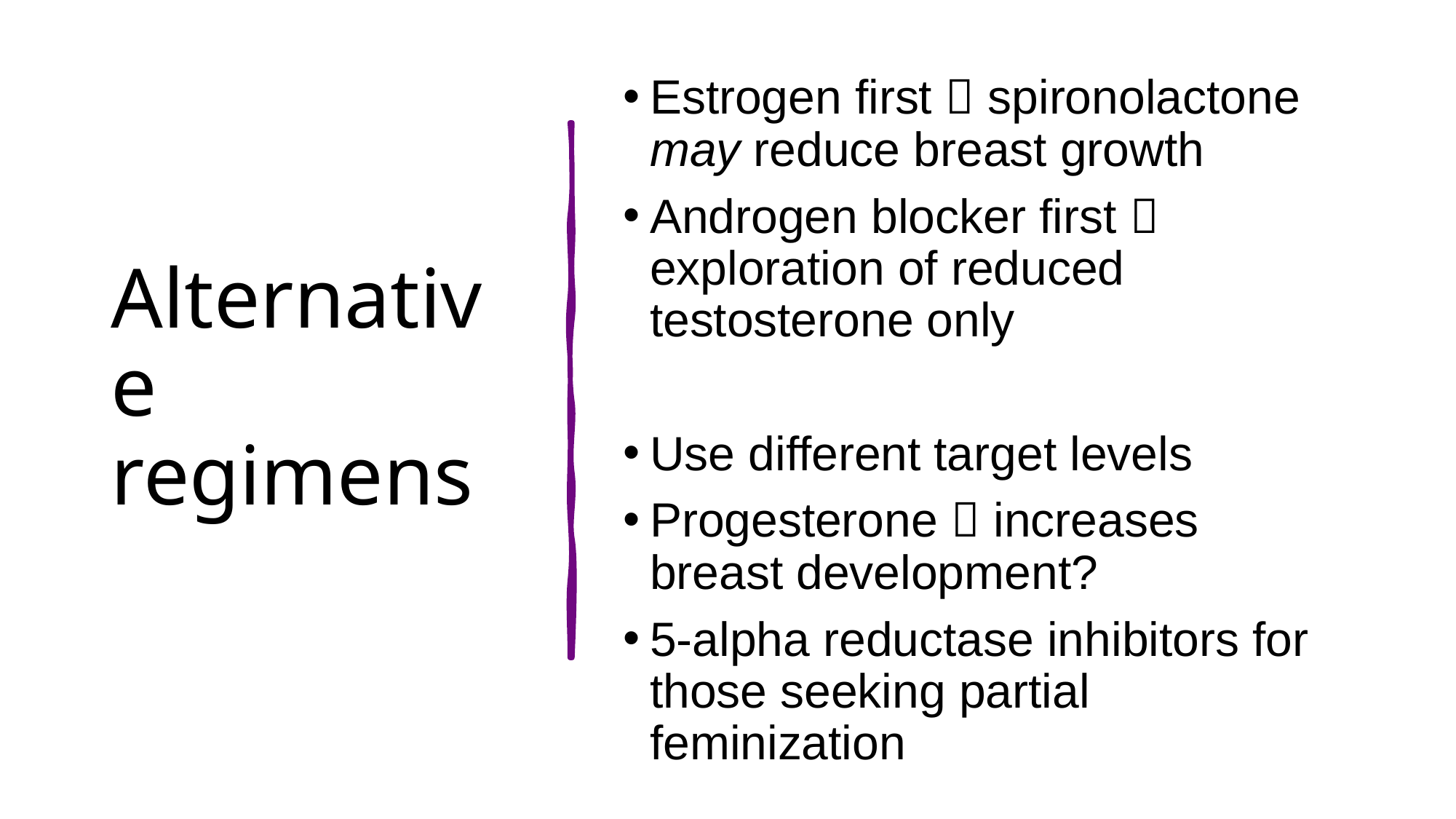

# Alternative regimens
Estrogen first  spironolactone may reduce breast growth
Androgen blocker first  exploration of reduced testosterone only
Use different target levels
Progesterone  increases breast development?
5-alpha reductase inhibitors for those seeking partial feminization

## Slide 59
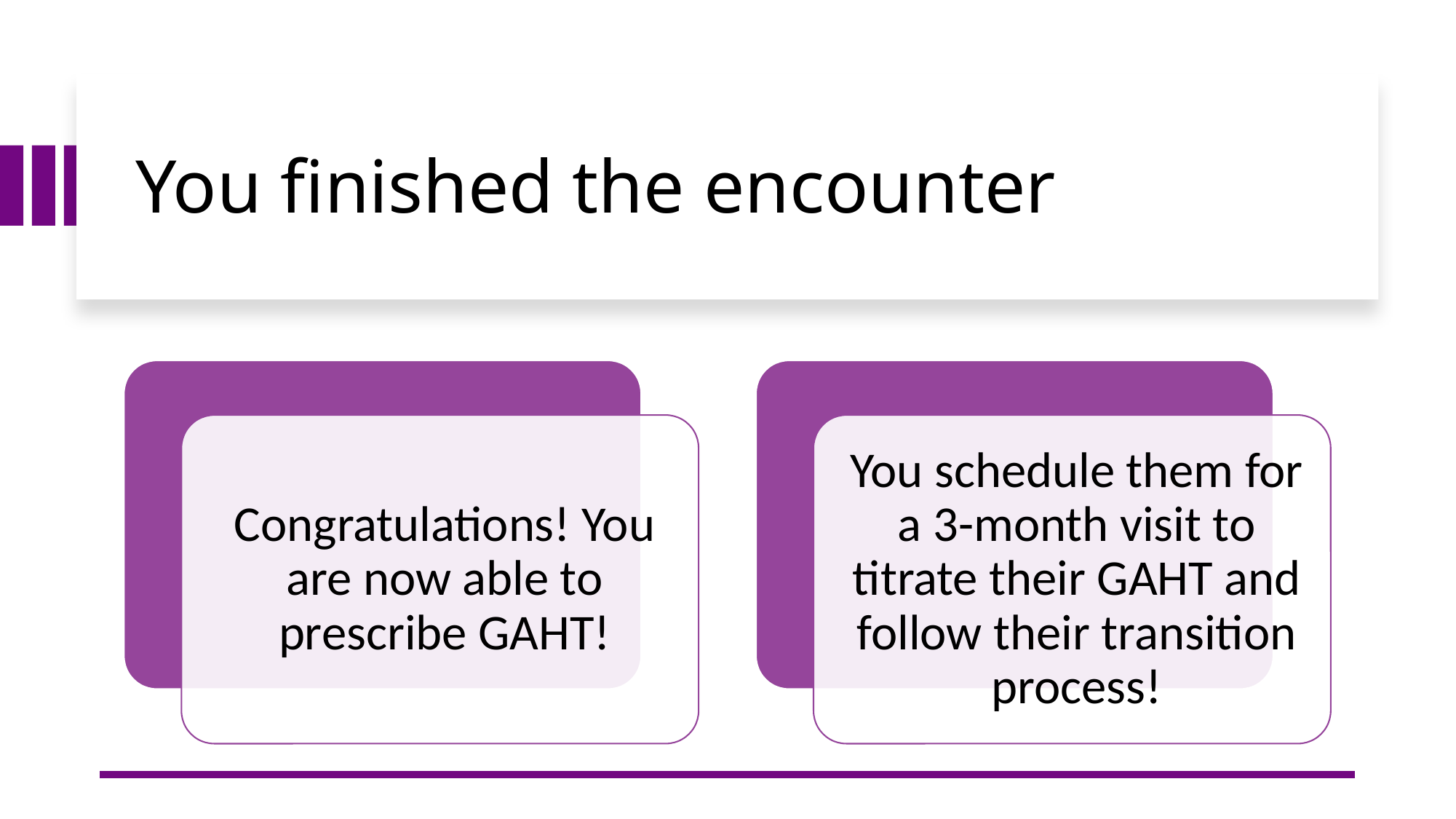

# You finished the encounter

## Slide 60
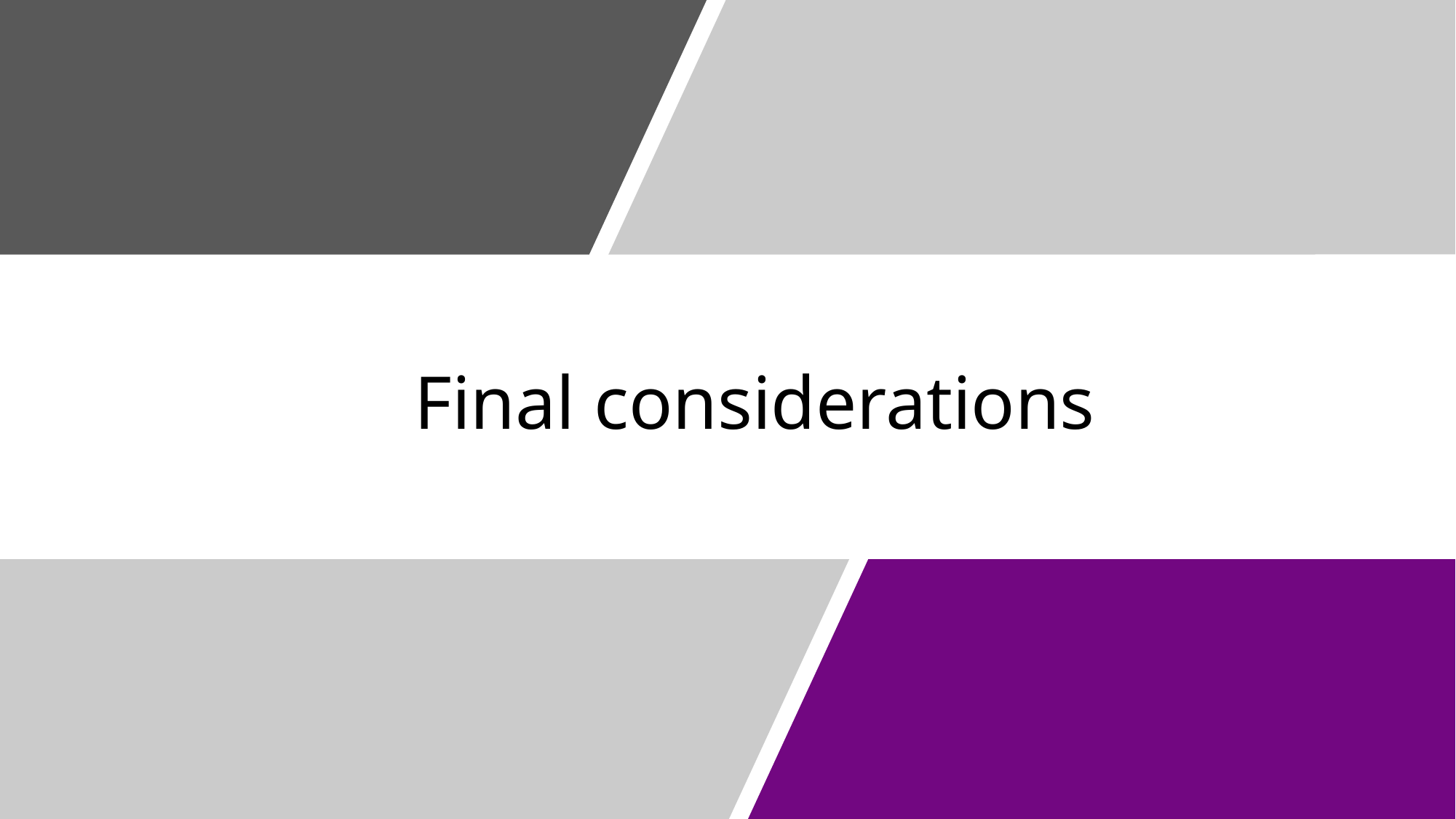

# Final considerations

## Slide 61
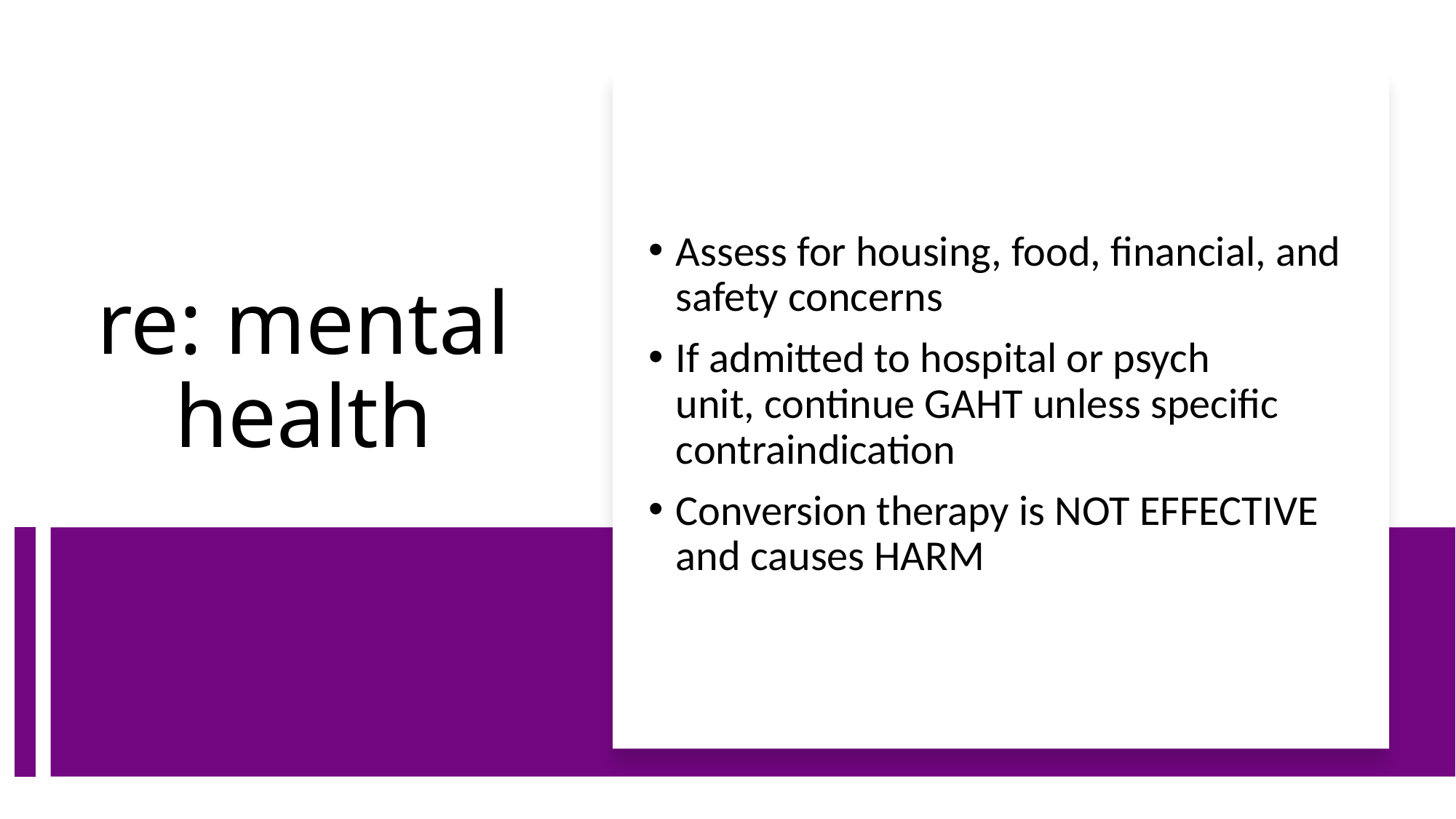

Assess for housing, food, financial, and safety concerns
If admitted to hospital or psych unit, continue GAHT unless specific contraindication
Conversion therapy is NOT EFFECTIVE and causes HARM
# re: mental health

## Slide 62
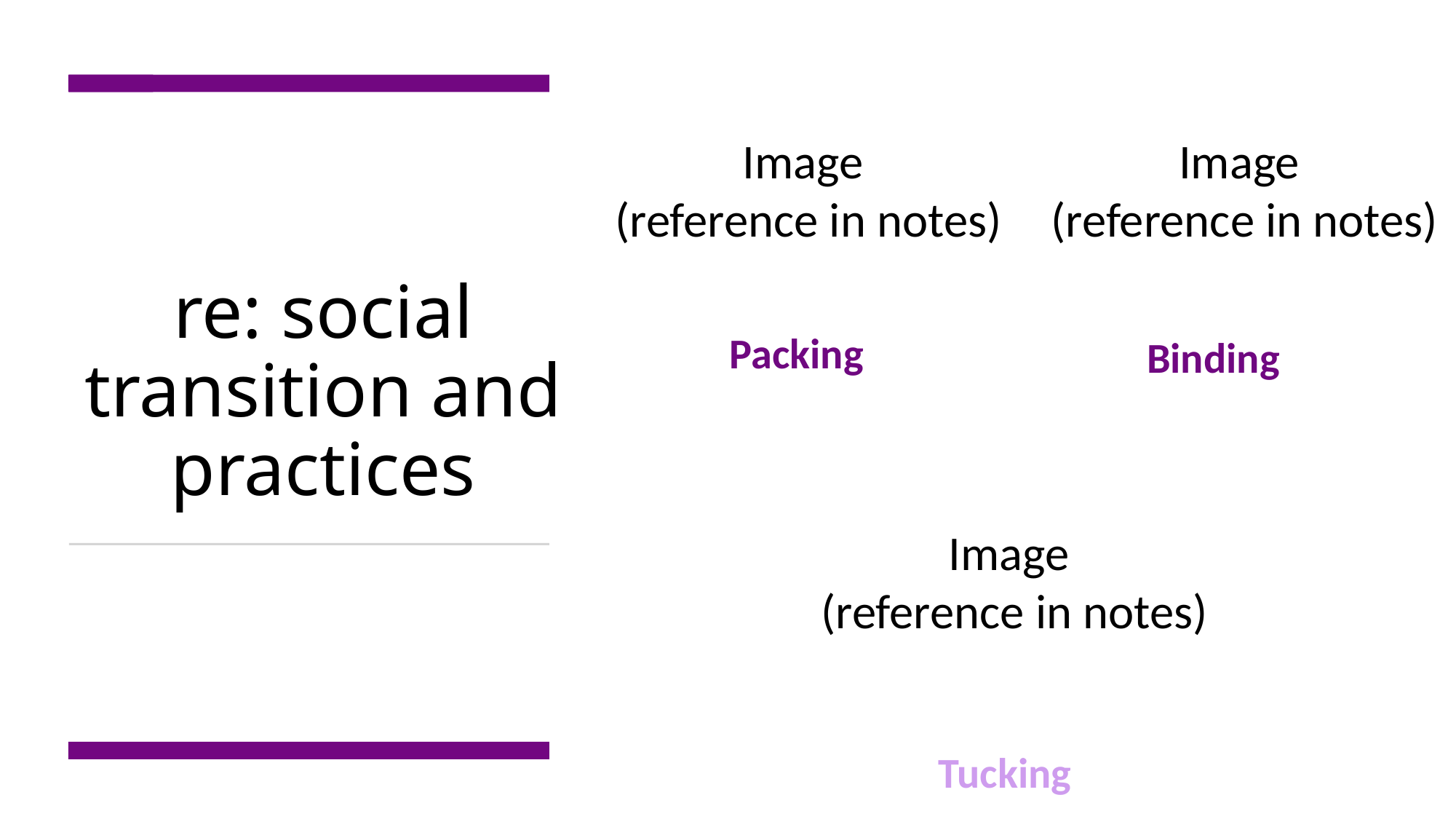

Image
(reference in notes)
Image
(reference in notes)
# re: social transition and practices
Packing
Binding
Image
(reference in notes)
Tucking

## Slide 63
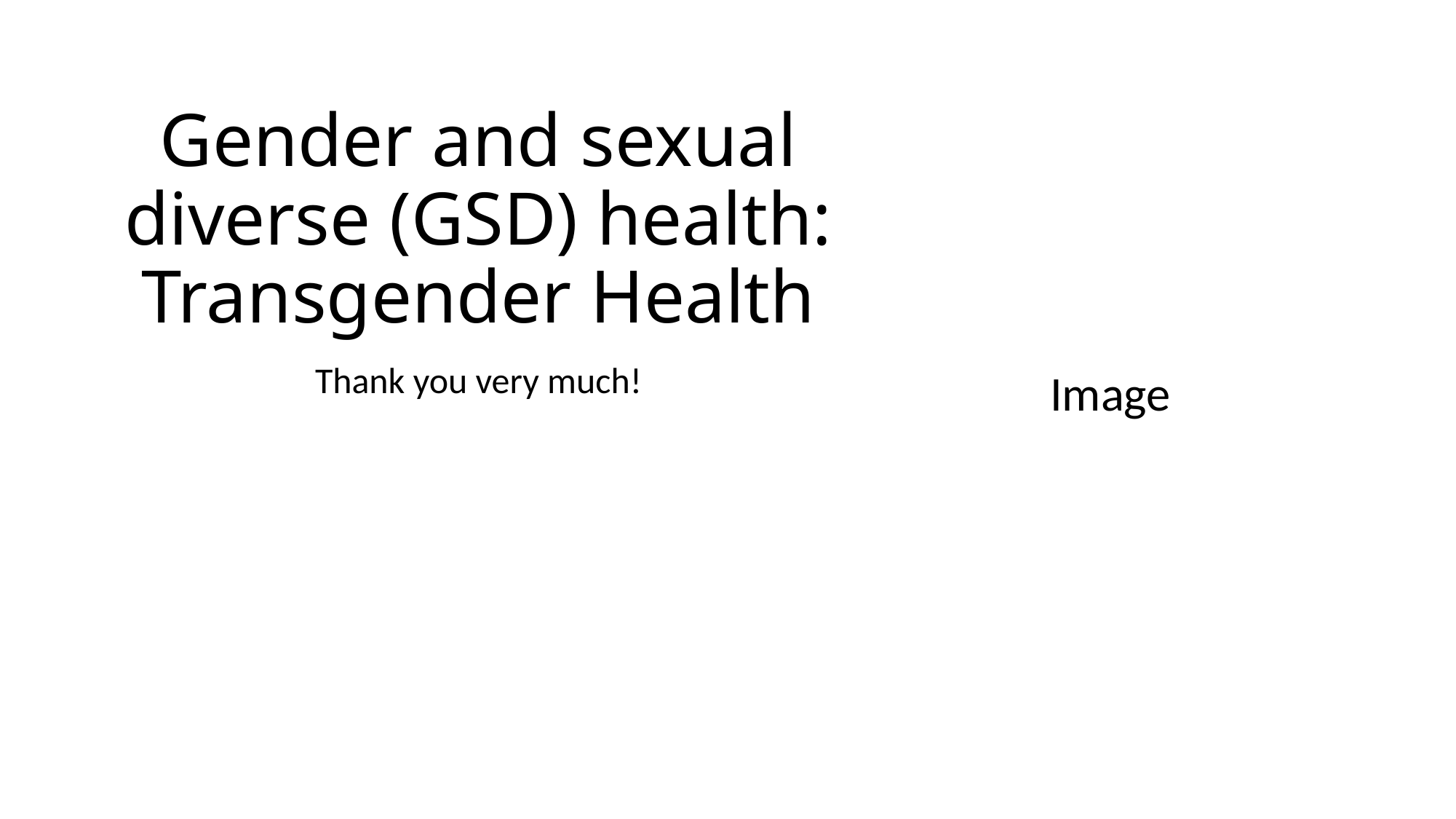

# Gender and sexual diverse (GSD) health: Transgender Health
Thank you very much!
Image

## Slide 64
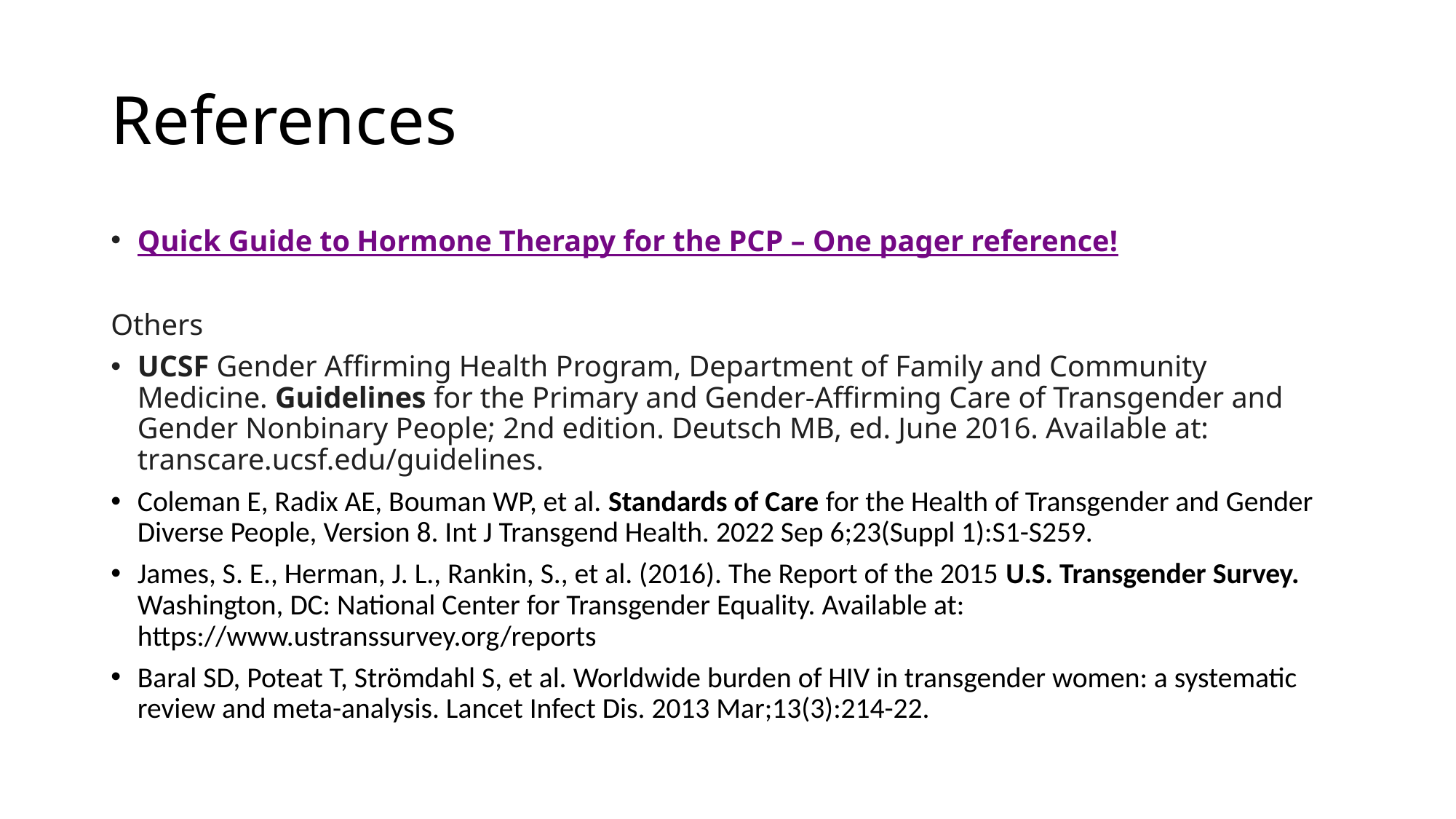

# References
Quick Guide to Hormone Therapy for the PCP – One pager reference!
Others
UCSF Gender Affirming Health Program, Department of Family and Community Medicine. Guidelines for the Primary and Gender-Affirming Care of Transgender and Gender Nonbinary People; 2nd edition. Deutsch MB, ed. June 2016. Available at: transcare.ucsf.edu/guidelines.
Coleman E, Radix AE, Bouman WP, et al. Standards of Care for the Health of Transgender and Gender Diverse People, Version 8. Int J Transgend Health. 2022 Sep 6;23(Suppl 1):S1-S259.
James, S. E., Herman, J. L., Rankin, S., et al. (2016). The Report of the 2015 U.S. Transgender Survey. Washington, DC: National Center for Transgender Equality. Available at: https://www.ustranssurvey.org/reports
Baral SD, Poteat T, Strömdahl S, et al. Worldwide burden of HIV in transgender women: a systematic review and meta-analysis. Lancet Infect Dis. 2013 Mar;13(3):214-22.
