# Supplementary material for: A Systematic and Practical Framework on Gender and Sexual Diverse (GSD) Health for Internal Medicine Residents
Source: MedEdPORTAL. 2025 Jun 17;21:11535. doi: 10.15766/mep_2374-8265.11535 (PMC12170925; doi:10.15766/mep_2374-8265.11535)
Supplement: Supplementary file 1 — GSD Health Handout.pptxGAHT Handout.pptxFacilitator Guide.docxGSD Health - Part 1.pptxGSD Health - Transgender Health.pptxGSD Health Survey.docxTGD Health Survey.docx [file mep_2374-8265.11535-s001.zip › F. GSD Health Survey.docx]

You are invited to participate in a survey about gender and sexual diverse (GSD) health. GSD is an umbrella term that encompasses people who identify as lesbian, gay, bisexual, transgender, queer (LGBTQ), or as any other gender or sexual minorities. The purpose of this study is to explore knowledge, attitudes, and perceptions of GSD health among IM residents. Your participation will require approximately 7 minutes.

You will receive no direct benefits from participating in this research study. There are no foreseeable risks associated with this questionnaire. It is completely voluntary. You may refuse to complete the questionnaire or exit at any time without penalty. We will not collect identifying information and all your responses will be stored initially with Qualtrics and kept strictly confidential.

Due to the absence of validated questionnaires in the literature, we adapted questions from previous studies^1,2,3,4^ and modified to align with the curriculum’s learning objectives:

1. Perucho J, Alzate-Duque L, Bhuiyan A, Sánchez JP, Sánchez NF. PrEP (Pre-Exposure Prophylaxis) Education for Clinicians: Caring for an MSM Patient. MedEdPORTAL. 2020;16:10908
2. Ufomata E, Eckstrand KL, Spagnoletti C, et al. Comprehensive Curriculum for Internal Medicine Residents on Primary Care of Patients Identifying as Lesbian, Gay, Bisexual, or Transgender. MedEdPORTAL. 2020;16:10875
3. Gallego J, Knudsen J. LGBTQI' Defined: An Introduction to Understanding and Caring for the Queer Community. MedEdPORTAL. 2015;11:10189
4. Sawning S, Steinbock S, Croley R, Combs R, Shaw A, Ganzel T. A first step in addressing medical education Curriculum gaps in lesbian-, gay-, bisexual-, and transgender-related content: The University of Louisville Lesbian, Gay, Bisexual, and Transgender Health Certificate Program. Educ Health (Abingdon). 2017;30(2):108-114

Clicking the “Next” button below indicates your consent to participate.

**Questionnaire: Sexual and Gender Diverse Health**

*How important do you feel it is…*

|  | Not important | Minimally important | Somewhat important | Very Important | Extremely important |
| --- | --- | --- | --- | --- | --- |
| … for you to discuss pronouns and gender identity with your primary care patients? | 1 | 2 | 3 | 4 | 5 |
| … to take a comprehensive sexual history with your primary care patients? | 1 | 2 | 3 | 4 | 5 |
| … to routinely discuss pre-exposure prophylaxis (PrEP) for HIV with high sexual risk patients? | 1 | 2 | 3 | 4 | 5 |

1. When screening for STIs in a cisgender man who had insertive oral sex with another man, you should routinely:
   1. Swab the pharynx for gonorrhea and chlamydia
   2. Swab the pharynx for gonorrhea only
   3. Swab the urethra for gonorrhea and chlamydia
   4. Swab the pharynx and the urethra for gonorrhea and chlamydia
   5. **Get urine sample for gonorrhea and chlamydia**
2. Which of the following is least likely to be transmitted between women who have sex with women (WSW)?
   1. Bacterial vaginosis
   2. Herpes simplex virus
   3. Trichomonas
   4. **Chlamydia**
3. Who is NOT eligible for PrEP?
   1. A patient with creatinine clearance of 67 ml/min
   2. **Patient with active hepatitis B infection**
   3. Patient with active hepatitis C infection
   4. Patient with diagnosis of syphilis within the last month
   5. Patient with history of pathologic fractures
   6. All of the above are eligible for PrEP
4. You are seeing a 42 y/o M with PMH of CKD 2/2 T1DM (eGFR 54 ml/min) and obesity in your clinic. Your patient identifies as gay. After your assessment you determine this patient meets criteria to start PrEP. What regimen could you use in this patient?
   1. Truvada once daily
   2. Truvada every other day to account for kidney function
   3. Descovy once daily
   4. Descovy every other day to account for kidney function
   5. Apretude once monthly for 2 months, then every 2 months
   6. B and D
   7. B and C
   8. **C and E**
5. After starting PrEP, when should patients come back for repeat bloodwork?
   1. 1 month
   2. 2 months
   3. **3 months**
   4. 6 months
   5. 12 months

*Select your level of agreement with the following statements.*

**“I feel confident in my abilities to…**

|  | Strongly Disagree | Disagree | Neutral | Agree | Strongly AgreeA |
| --- | --- | --- | --- | --- | --- |
| …discuss gender identity and sexual orientation with my patients | 1 | 2 | 3 | 4 | 5 |
| …use inclusive language during patient encounters | 1 | 2 | 3 | 4 | 5 |
| …take a comprehensive sexual history | 1 | 2 | 3 | 4 | 5 |
| …perform comprehensive STI screening (including appropriate swabs for different sites) | 1 | 2 | 3 | 4 | 5 |
| …prescribe HIV pre-exposure prophylaxis (PrEP) when indicated and monitor for side effects | 1 | 2 | 3 | 4 | 5 |

1. I feel more comfortable discussing sexual history with a heterosexual cisgender patient than a GSD patient
   1. Disagree, neutral, agree
2. I routinely take a comprehensive sexual history
   1. Disagree, neutral, agree
3. Do you identify as lesbian, gay, bisexual, transgender, queer or as a gender and sexual diverse person?
   1. Yes
   2. No
   3. Questioning
   4. Prefer not to answer

17. Are you in the Categorical or Primary Care Track?

1. Categorical
2. Primary Care Track

**Post-training questions**

*How important do you feel it is…*

|  | Not important | Minimally important | Somewhat important | Very Important | Extremely important |
| --- | --- | --- | --- | --- | --- |
| … for you to discuss pronouns and gender identity with your primary care patients? | 1 | 2 | 3 | 4 | 5 |
| … to take a comprehensive sexual history with your primary care patients? | 1 | 2 | 3 | 4 | 5 |
| … to routinely discuss pre-exposure prophylaxis (PrEP) for HIV with high sexual risk patients? | 1 | 2 | 3 | 4 | 5 |

1. When screening for STIs in a cisgender man who had insertive oral sex with another man, you should routinely:
2. Swab the pharynx for gonorrhea and chlamydia
3. Swab the pharynx for gonorrhea only
4. Swab the urethra for gonorrhea and chlamydia
5. Swab the pharynx and the urethra for gonorrhea and chlamydia
6. **Get urine sample for gonorrhea and chlamydia**
7. Which of the following is least likely to be transmitted between women who have sex with women (WSW)?
   1. Bacterial vaginosis
   2. Herpes simplex virus
   3. Trichomonas
   4. **Chlamydia**
8. Who is NOT eligible for PrEP?
   1. A patient with creatinine clearance of 67 ml/min
   2. **Patient with active hepatitis B infection**
   3. Patient with active hepatitis C infection
   4. Patient with diagnosis of syphilis within the last month
   5. Patient with history of pathologic fractures
   6. All of the above are eligible for PrEP
9. You are seeing a 42 y/o M with PMH of CKD 2/2 T1DM (eGFR 54 ml/min) and obesity in your clinic. Your patient identifies as gay. After your assessment you determine this patient meets criteria to start PrEP. What regimen could you use in this patient?
   1. Truvada once daily
   2. Truvada every other day to account for kidney function
   3. Descovy once daily
   4. Descovy every other day to account for kidney function
   5. Apretude once monthly for 2 months, then every 2 months
   6. B and D
   7. B and C
   8. **C and E**
10. After starting PrEP, when should patients come back for repeat bloodwork?
    1. 1 month
    2. 2 months
    3. **3 months**
    4. 6 months
    5. 12 months

*Select your level of agreement with the following statements.*

**“I feel confident in my abilities to…**

|  | Strongly Disagree | Disagree | Neutral | Agree | Strongly Agree |
| --- | --- | --- | --- | --- | --- |
| …discuss gender identity and sexual orientation with my patients | 1 | 2 | 3 | 4 | 5 |
| …use inclusive language during patient encounters | 1 | 2 | 3 | 4 | 5 |
| …take a comprehensive sexual history | 1 | 2 | 3 | 4 | 5 |
| …perform comprehensive STI screening (including appropriate swabs for different sites) | 1 | 2 | 3 | 4 | 5 |
| …prescribe HIV pre-exposure prophylaxis (PrEP) when indicated and monitor for side effects | 1 | 2 | 3 | 4 | 5 |

*Select your level of agreement with the following statements:*

|  | Strongly Disagree | Disagree | Neutral | Agree | Strongly Agree |
| --- | --- | --- | --- | --- | --- |
| The session was presented clearly (e.g. definitions, clarity of concepts, slides) | 1 | 2 | 3 | 4 | 5 |
| The session was well organized (flow and balance between didactic and interactive activities) | 1 | 2 | 3 | 4 | 5 |
| This session will improve my communication with GSD patients | 1 | 2 | 3 | 4 | 5 |
| The instructor(s) was/were prepared and knowledgeable | 1 | 2 | 3 | 4 | 5 |

**Overall Evaluation:**

|  | Poor | Fair | Average | Very Good | Excellent |
| --- | --- | --- | --- | --- | --- |
| Please rate the utility of this session to you as an internal medicine resident | 1 | 2 | 3 | 4 | 5 |

1. Describe TWO things that you have learned from this session that you plan to apply in your practice
2. Describe one way in which this session could be improved
