# Supplementary material for: A Systematic and Practical Framework on Gender and Sexual Diverse (GSD) Health for Internal Medicine Residents
Source: MedEdPORTAL. 2025 Jun 17;21:11535. doi: 10.15766/mep_2374-8265.11535 (PMC12170925; doi:10.15766/mep_2374-8265.11535)
Supplement: Supplementary file 1 — GSD Health Handout.pptxGAHT Handout.pptxFacilitator Guide.docxGSD Health - Part 1.pptxGSD Health - Transgender Health.pptxGSD Health Survey.docxTGD Health Survey.docx [file mep_2374-8265.11535-s001.zip › G. TGD Health Survey.docx]

You are invited to participate in a survey about transgender and gender diverse (TGD) health. The purpose of this study is to explore knowledge, attitudes, and perceptions of TGD health among IM residents. Your participation will require approximately 7 minutes.

You will receive no direct benefits from participating in this research study. There are no foreseeable risks associated with this questionnaire. It is completely voluntary. You may refuse to complete the questionnaire or exit at any time without penalty. We will not collect identifying information and all your responses will be stored initially with Qualtrics and kept strictly confidential.

Due to the absence of validated questionnaires in the literature, we adapted questions from previous studies^1,2,3,4^ and modified to align with the curriculum’s learning objectives:

1. Perucho J, Alzate-Duque L, Bhuiyan A, Sánchez JP, Sánchez NF. PrEP (Pre-Exposure Prophylaxis) Education for Clinicians: Caring for an MSM Patient. MedEdPORTAL. 2020;16:10908
2. Ufomata E, Eckstrand KL, Spagnoletti C, et al. Comprehensive Curriculum for Internal Medicine Residents on Primary Care of Patients Identifying as Lesbian, Gay, Bisexual, or Transgender. MedEdPORTAL. 2020;16:10875
3. Gallego J, Knudsen J. LGBTQI' Defined: An Introduction to Understanding and Caring for the Queer Community. MedEdPORTAL. 2015;11:10189
4. Sawning S, Steinbock S, Croley R, Combs R, Shaw A, Ganzel T. A first step in addressing medical education Curriculum gaps in lesbian-, gay-, bisexual-, and transgender-related content: The University of Louisville Lesbian, Gay, Bisexual, and Transgender Health Certificate Program. Educ Health (Abingdon). 2017;30(2):108-114

Clicking the “Next” button below indicates your consent to participate.

**Questionnaire: Transgender and Gender Diverse (TGD) Health**

TGD: Transgender and sexual diverse
GAHT: Gender affirming hormone therapy

*How important do you feel it is…*

|  | Not important | Minimally important | Somewhat important | Very Important | Extremely important |
| --- | --- | --- | --- | --- | --- |
| … for you to discuss gender identity with your primary care patients? | 1 | 2 | 3 | 4 | 5 |
| … to assess for gender dysphoria/incongruence in TGD patients? | 1 | 2 | 3 | 4 | 5 |
| ... to offer transition-related care for TGD patients in your primary care clinic vs. referring to a specialized clinic? | 1 | 2 | 3 | 4 | 5 |
| … to prescribe GAHT for TGD patients as a PCP? | 1 | 2 | 3 | 4 | 5 |

1. Which of the following is true about the initial evaluation of TGD patients prior to starting GAHT?
   1. All patients need a letter of support from a mental health provider
   2. Explain that specific sex characteristics will vary according to the hormone regimen
   3. **Offer fertility specialist referral to all patients**
   4. GAHT will affect sperm/oocyte function after 3 months, so no contraception is required thereafter
2. Which of the following is a likely contraindication for GAHT?
   1. Coronary artery disease with a history of CABG and stent placement
   2. Venous thromboembolism after midline placement
   3. **History of breast cancer**
   4. Opioid use disorder on suboxone
   5. Major Depression on SSRIs
3. Which lab is not required as part of the initial assessment to prescribe GAHT?
   1. Total testosterone
   2. Estradiol
   3. BMP
   4. **LFT**
   5. CBC
4. What is true about feminization hormone therapy?
   1. Oral estradiol increases risk of venous thromboembolism by a factor of 45
   2. Ethinyl estradiol is first line therapy
   3. **Transdermal estradiol should be considered in patients with a history of venous thromboembolism**
   4. There is increased risk of hyperprolactinemia and cardiovascular disease
5. What is true about masculinization hormone therapy?
   1. Oral testosterone is first line therapy
   2. Typical frequency of IM or SQ testosterone is every 4 weeks
   3. Testosterone increases the risk of cardiovascular disease in transgender men
   4. **Fertility is mostly reversible**
   5. Testosterone can be continued throughout pregnancy

*Select your level of agreement with the following statements.*

**“I feel confident in my abilities to…**

|  | Strongly Disagree | Disagree | Neutral | Agree | Strongly Agree |
| --- | --- | --- | --- | --- | --- |
| …discuss gender identity with my patients | 1 | 2 | 3 | 4 | 5 |
| …assess for gender dysphoria/incongruence | 1 | 2 | 3 | 4 | 5 |
| …explain the physical changes and the impact on fertility when prescribing GAHT | 1 | 2 | 3 | 4 | 5 |
| …prescribe (GAHT) | 1 | 2 | 3 | 4 | 5 |
| …monitor the side effects and hormone target levels after initiation of GAHT | 1 | 2 | 3 | 4 | 5 |

1. Do you identify as lesbian, gay, bisexual, transgender, queer or as a gender and sexual diverse person?
   1. Yes
   2. No
   3. Questioning
   4. Prefer not to answer
2. Are you in the Categorical or Primary Care Track?
3. Categorical
4. Primary Care Track

**Post-training questions**

*How important do you feel it is…*

|  | Not important | Minimally important | Somewhat important | Very Important | Extremely important |
| --- | --- | --- | --- | --- | --- |
| … for you to discuss gender identity with your primary care patients? | 1 | 2 | 3 | 4 | 5 |
| … to assess for gender dysphoria/incongruence in TGD patients? | 1 | 2 | 3 | 4 | 5 |
| ... to offer transition-related care for TGD patients in your primary care clinic vs. referring to a specialized clinic? | 1 | 2 | 3 | 4 | 5 |
| … to prescribe GAHT for TGD patients as a PCP? | 1 | 2 | 3 | 4 | 5 |

1. Which of the following is true about the initial evaluation of TGD patients prior to starting GAHT?
   1. All patients need a letter of support from a mental health provider
   2. Explain that specific sex characteristics will vary according to the hormone regimen
   3. **Offer fertility specialist referral to all patients**
   4. GAHT will affect sperm/oocyte function after 3 months, so no contraception is required thereafter
2. Which of the following is a likely contraindication for GAHT?
   1. Coronary artery disease with a history of CABG and stent placement
   2. Venous thromboembolism after midline placement
   3. **History of breast cancer**
   4. Opioid use disorder on suboxone
   5. Major Depression on SSRIs
3. Which lab is not required as part of the initial assessment to prescribe GAHT?
   1. Total testosterone
   2. Estradiol
   3. BMP
   4. **LFT**
   5. CBC
4. What is true about feminization hormone therapy?
   1. Oral estradiol increases risk of venous thromboembolism by a factor of 45
   2. Ethinyl estradiol is first line therapy
   3. **Transdermal estradiol should be considered in patients with a history of venous thromboembolism**
   4. There is increased risk of hyperprolactinemia and cardiovascular disease
5. What is true about masculinization hormone therapy?
   1. Oral testosterone is first line therapy
   2. Typical frequency of IM or SQ testosterone is every 4 weeks
   3. Testosterone increases the risk of cardiovascular disease in transgender men
   4. **Fertility is mostly reversible**
   5. Testosterone can be continued throughout pregnancy

*Select your level of agreement with the following statements.*

**“I feel confident in my abilities to…**

|  | Strongly Disagree | Disagree | Neutral | Agree | Strongly Agree |
| --- | --- | --- | --- | --- | --- |
| …discuss gender identity with my patients | 1 | 2 | 3 | 4 | 5 |
| …assess for gender dysphoria/incongruence | 1 | 2 | 3 | 4 | 5 |
| …explain the physical changes and the impact on fertility when prescribing GAHT | 1 | 2 | 3 | 4 | 5 |
| …prescribe (GAHT) | 1 | 2 | 3 | 4 | 5 |
| …monitor the side effects and hormone target levels after initiation of GAHT | 1 | 2 | 3 | 4 | 5 |

*Select your level of agreement with the following statements:*

|  | Strongly Disagree | Disagree | Neutral | Agree | Strongly Agree |
| --- | --- | --- | --- | --- | --- |
| The session was presented clearly (e.g. definitions, clarity of concepts, slides) | 1 | 2 | 3 | 4 | 5 |
| The session was well organized (flow and balance between didactic and interactive activities) | 1 | 2 | 3 | 4 | 5 |
| This session will improve my ability to prescribe GAHT for TGD patients | 1 | 2 | 3 | 4 | 5 |
| The instructor(s) was/were prepared and knowledgeable | 1 | 2 | 3 | 4 | 5 |

**Overall Evaluation:**

|  | Poor | Fair | Average | Very Good | Excellent |
| --- | --- | --- | --- | --- | --- |
| Please rate the utility of this session to you as an internal medicine resident | 1 | 2 | 3 | 4 | 5 |

1. Describe TWO things that you have learned from this session that you plan to apply in your practice
2. Describe one way in which this session could be improved
