# Supplementary material for: A Systematic and Practical Framework on Gender and Sexual Diverse (GSD) Health for Internal Medicine Residents
Source: MedEdPORTAL. 2025 Jun 17;21:11535. doi: 10.15766/mep_2374-8265.11535 (PMC12170925; doi:10.15766/mep_2374-8265.11535)
Supplement: Supplementary file 1 — GSD Health Handout.pptxGAHT Handout.pptxFacilitator Guide.docxGSD Health - Part 1.pptxGSD Health - Transgender Health.pptxGSD Health Survey.docxTGD Health Survey.docx [file mep_2374-8265.11535-s001.zip › A. GSD Health Handout.pptx]

## Slide 1
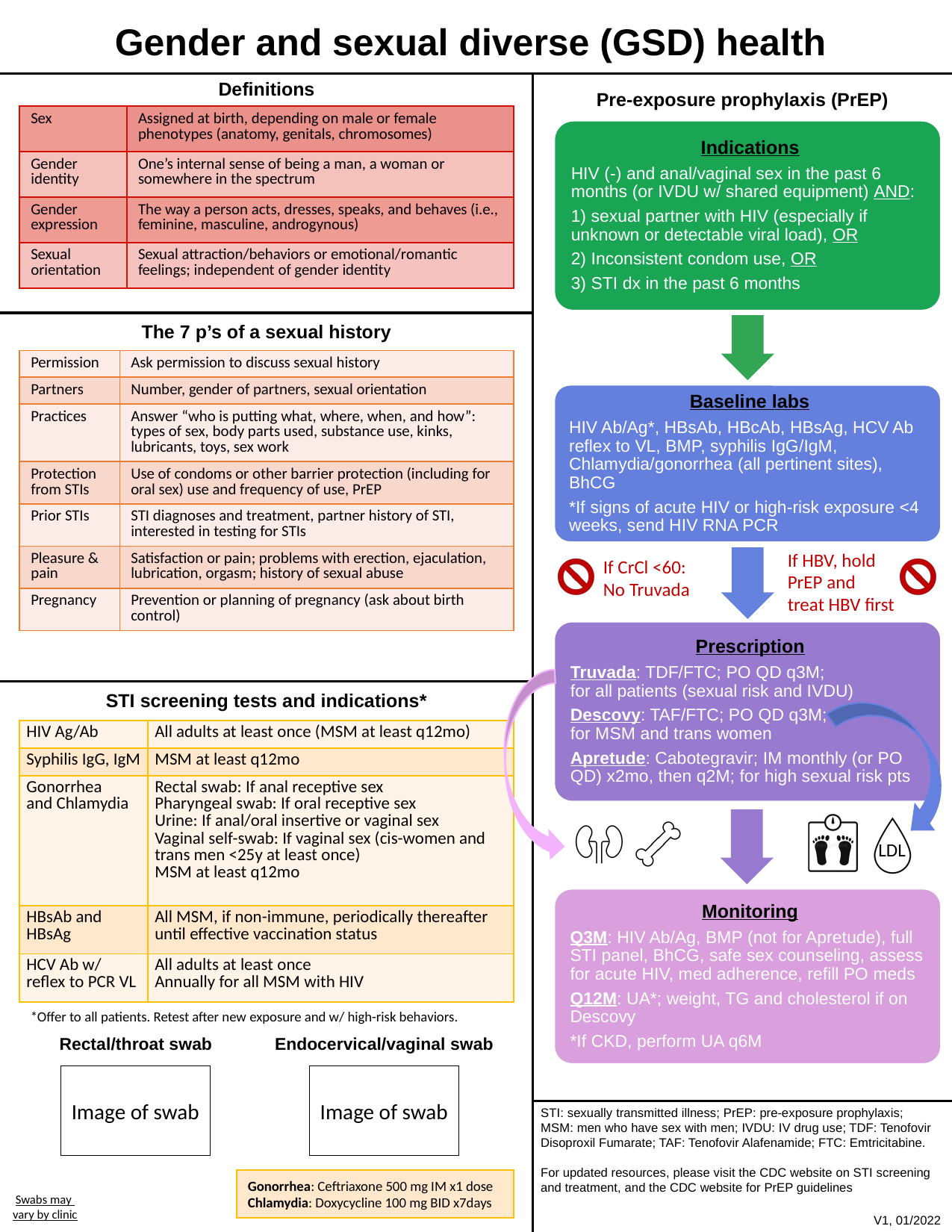

Gender and sexual diverse (GSD) health
Definitions
Pre-exposure prophylaxis (PrEP)
| Sex | Assigned at birth, depending on male or female phenotypes (anatomy, genitals, chromosomes) |
| --- | --- |
| Gender identity | One’s internal sense of being a man, a woman or somewhere in the spectrum |
| Gender expression | The way a person acts, dresses, speaks, and behaves (i.e., feminine, masculine, androgynous) |
| Sexual orientation | Sexual attraction/behaviors or emotional/romantic feelings; independent of gender identity |
The 7 p’s of a sexual history
| Permission | Ask permission to discuss sexual history |
| --- | --- |
| Partners | Number, gender of partners, sexual orientation |
| Practices | Answer “who is putting what, where, when, and how”: types of sex, body parts used, substance use, kinks, lubricants, toys, sex work |
| Protection from STIs | Use of condoms or other barrier protection (including for oral sex) use and frequency of use, PrEP |
| Prior STIs | STI diagnoses and treatment, partner history of STI, interested in testing for STIs |
| Pleasure & pain | Satisfaction or pain; problems with erection, ejaculation, lubrication, orgasm; history of sexual abuse |
| Pregnancy | Prevention or planning of pregnancy (ask about birth control) |
If HBV, hold
PrEP and
treat HBV first
If CrCl <60:
No Truvada
STI screening tests and indications*
| HIV Ag/Ab | All adults at least once (MSM at least q12mo) |
| --- | --- |
| Syphilis IgG, IgM | MSM at least q12mo |
| Gonorrhea and Chlamydia | Rectal swab: If anal receptive sex Pharyngeal swab: If oral receptive sex Urine: If anal/oral insertive or vaginal sex Vaginal self-swab: If vaginal sex (cis-women and trans men <25y at least once) MSM at least q12mo |
| HBsAb and HBsAg | All MSM, if non-immune, periodically thereafter until effective vaccination status |
| HCV Ab w/ reflex to PCR VL | All adults at least once Annually for all MSM with HIV |
LDL
*Offer to all patients. Retest after new exposure and w/ high-risk behaviors.
Rectal/throat swab
Endocervical/vaginal swab
Image of swab
Image of swab
STI: sexually transmitted illness; PrEP: pre-exposure prophylaxis; MSM: men who have sex with men; IVDU: IV drug use; TDF: Tenofovir Disoproxil Fumarate; TAF: Tenofovir Alafenamide; FTC: Emtricitabine.
For updated resources, please visit the CDC website on STI screening and treatment, and the CDC website for PrEP guidelines
Gonorrhea: Ceftriaxone 500 mg IM x1 dose
Chlamydia: Doxycycline 100 mg BID x7days
Swabs may
vary by clinic
V1, 01/2022
