# Supplementary material for: A Systematic and Practical Framework on Gender and Sexual Diverse (GSD) Health for Internal Medicine Residents
Source: MedEdPORTAL. 2025 Jun 17;21:11535. doi: 10.15766/mep_2374-8265.11535 (PMC12170925; doi:10.15766/mep_2374-8265.11535)
Supplement: Supplementary file 1 — GSD Health Handout.pptxGAHT Handout.pptxFacilitator Guide.docxGSD Health - Part 1.pptxGSD Health - Transgender Health.pptxGSD Health Survey.docxTGD Health Survey.docx [file mep_2374-8265.11535-s001.zip › B. GAHT Handout.pptx]

## Slide 1
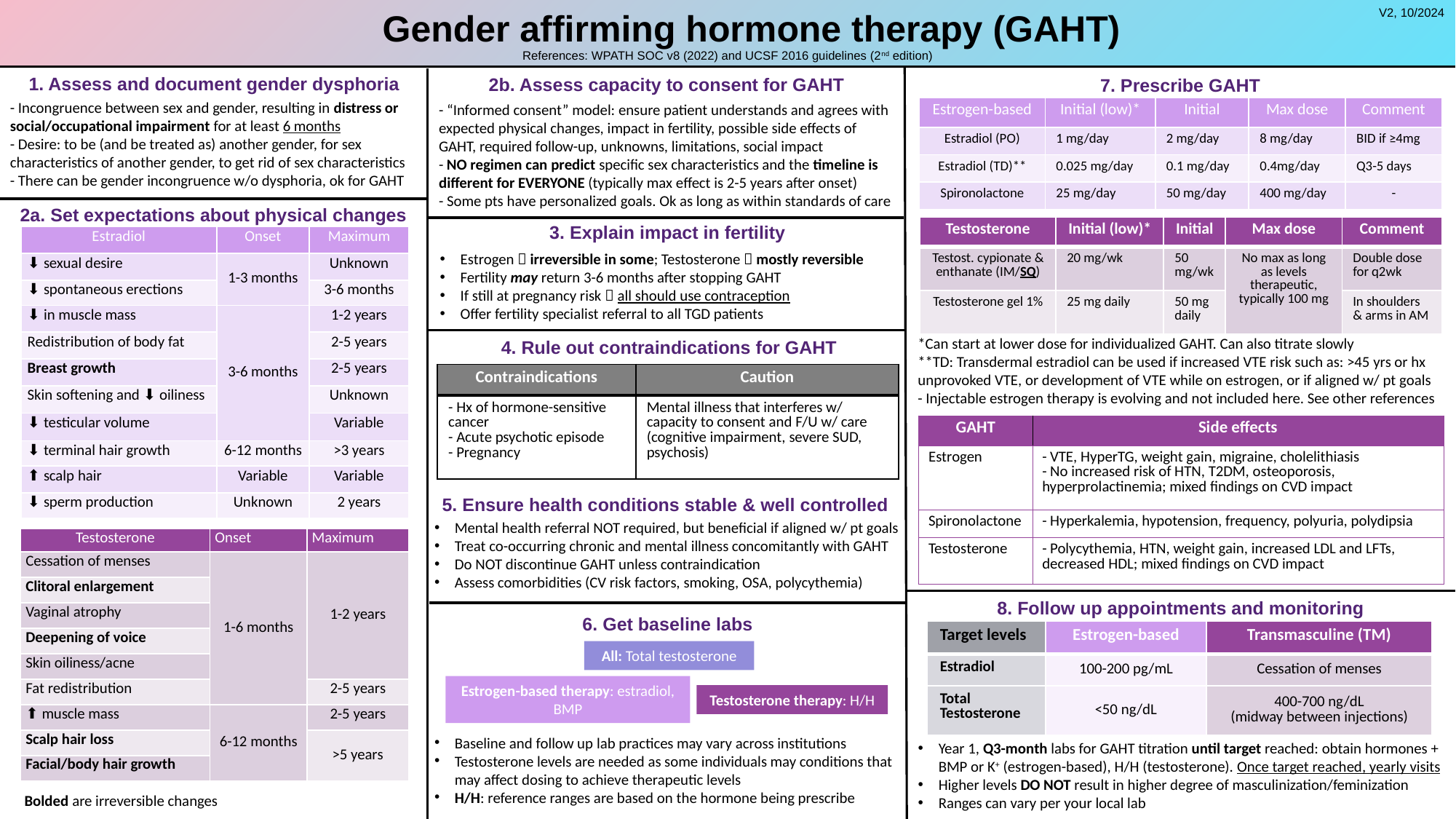

Gender affirming hormone therapy (GAHT)
V2, 10/2024
References: WPATH SOC v8 (2022) and UCSF 2016 guidelines (2nd edition)
1. Assess and document gender dysphoria
2b. Assess capacity to consent for GAHT
7. Prescribe GAHT
- Incongruence between sex and gender, resulting in distress or social/occupational impairment for at least 6 months
- Desire: to be (and be treated as) another gender, for sex characteristics of another gender, to get rid of sex characteristics
- There can be gender incongruence w/o dysphoria, ok for GAHT
- “Informed consent” model: ensure patient understands and agrees with expected physical changes, impact in fertility, possible side effects of GAHT, required follow-up, unknowns, limitations, social impact
- NO regimen can predict specific sex characteristics and the timeline is different for EVERYONE (typically max effect is 2-5 years after onset)
- Some pts have personalized goals. Ok as long as within standards of care
| Estrogen-based | Initial (low)\* | Initial | Max dose | Comment |
| --- | --- | --- | --- | --- |
| Estradiol (PO) | 1 mg/day | 2 mg/day | 8 mg/day | BID if ≥4mg |
| Estradiol (TD)\*\* | 0.025 mg/day | 0.1 mg/day | 0.4mg/day | Q3-5 days |
| Spironolactone | 25 mg/day | 50 mg/day | 400 mg/day | - |
2a. Set expectations about physical changes
3. Explain impact in fertility
| Testosterone | Initial (low)\* | Initial | Max dose | Comment |
| --- | --- | --- | --- | --- |
| Testost. cypionate & enthanate (IM/SQ) | 20 mg/wk | 50 mg/wk | No max as long as levels therapeutic, typically 100 mg | Double dose for q2wk |
| Testosterone gel 1% | 25 mg daily | 50 mg daily | | In shoulders & arms in AM |
| Estradiol | Onset | Maximum |
| --- | --- | --- |
| ⬇ sexual desire | 1-3 months | Unknown |
| ⬇ spontaneous erections | | 3-6 months |
| ⬇ in muscle mass | 3-6 months | 1-2 years |
| Redistribution of body fat | | 2-5 years |
| Breast growth | | 2-5 years |
| Skin softening and ⬇ oiliness | | Unknown |
| ⬇ testicular volume | | Variable |
| ⬇ terminal hair growth | 6-12 months | >3 years |
| ⬆️ scalp hair | Variable | Variable |
| ⬇ sperm production | Unknown | 2 years |
Estrogen  irreversible in some; Testosterone  mostly reversible
Fertility may return 3-6 months after stopping GAHT
If still at pregnancy risk  all should use contraception
Offer fertility specialist referral to all TGD patients
*Can start at lower dose for individualized GAHT. Can also titrate slowly
**TD: Transdermal estradiol can be used if increased VTE risk such as: >45 yrs or hx unprovoked VTE, or development of VTE while on estrogen, or if aligned w/ pt goals
- Injectable estrogen therapy is evolving and not included here. See other references
4. Rule out contraindications for GAHT
| Contraindications | Caution |
| --- | --- |
| - Hx of hormone-sensitive cancer - Acute psychotic episode - Pregnancy | Mental illness that interferes w/ capacity to consent and F/U w/ care (cognitive impairment, severe SUD, psychosis) |
| GAHT | Side effects |
| --- | --- |
| Estrogen | - VTE, HyperTG, weight gain, migraine, cholelithiasis - No increased risk of HTN, T2DM, osteoporosis, hyperprolactinemia; mixed findings on CVD impact |
| Spironolactone | - Hyperkalemia, hypotension, frequency, polyuria, polydipsia |
| Testosterone | - Polycythemia, HTN, weight gain, increased LDL and LFTs, decreased HDL; mixed findings on CVD impact |
5. Ensure health conditions stable & well controlled
Mental health referral NOT required, but beneficial if aligned w/ pt goals
Treat co-occurring chronic and mental illness concomitantly with GAHT
Do NOT discontinue GAHT unless contraindication
Assess comorbidities (CV risk factors, smoking, OSA, polycythemia)
| Testosterone | Onset | Maximum |
| --- | --- | --- |
| Cessation of menses | 1-6 months | 1-2 years |
| Clitoral enlargement | | |
| Vaginal atrophy | | |
| Deepening of voice | | |
| Skin oiliness/acne | | |
| Fat redistribution | | 2-5 years |
| ⬆️ muscle mass | 6-12 months | 2-5 years |
| Scalp hair loss | | >5 years |
| Facial/body hair growth | | |
8. Follow up appointments and monitoring
6. Get baseline labs
| Target levels | Estrogen-based | Transmasculine (TM) |
| --- | --- | --- |
| Estradiol | 100-200 pg/mL | Cessation of menses |
| Total Testosterone | <50 ng/dL | 400-700 ng/dL (midway between injections) |
All: Total testosterone
Estrogen-based therapy: estradiol, BMP
Testosterone therapy: H/H
Baseline and follow up lab practices may vary across institutions
Testosterone levels are needed as some individuals may conditions that may affect dosing to achieve therapeutic levels
H/H: reference ranges are based on the hormone being prescribe
Year 1, Q3-month labs for GAHT titration until target reached: obtain hormones + BMP or K+ (estrogen-based), H/H (testosterone). Once target reached, yearly visits
Higher levels DO NOT result in higher degree of masculinization/feminization
Ranges can vary per your local lab
Bolded are irreversible changes
