# Supplementary material for: A Systematic and Practical Framework on Gender and Sexual Diverse (GSD) Health for Internal Medicine Residents
Source: MedEdPORTAL. 2025 Jun 17;21:11535. doi: 10.15766/mep_2374-8265.11535 (PMC12170925; doi:10.15766/mep_2374-8265.11535)
Supplement: Supplementary file 1 — GSD Health Handout.pptxGAHT Handout.pptxFacilitator Guide.docxGSD Health - Part 1.pptxGSD Health - Transgender Health.pptxGSD Health Survey.docxTGD Health Survey.docx [file mep_2374-8265.11535-s001.zip › _Educational Summary Report_11535.pdf]

MedEdPORTAL® | The AAMC Journal of Teaching and Learning Resources

Original Publication

Open Access

A Systematic and Practical Framework on Gender and Sexual Diverse (GSD) Health for Internal Medicine Residents

Sebastian Suarez, MD, MPH*, Emily Lupton Lupez, MD, MS, MPH, Katherine L. Modzelewski, MD, Chad Hinkle, MD, Carl G. Streed Jr., MD, MPH, Jennifer Siegel, MD

*Corresponding author: sxs4395@med.miami.edu

Abstract

Introduction: Internal medicine (IM) residents often lack the knowledge, comfort, and competency to care for gender and sexual diverse (GSD) patients, contributing to health disparities. We developed a practical, evidence-based framework providing residents with a systematic, stepwise approach to addressing GSD health needs. **Methods:** The curriculum was implemented at an urban safety-net hospital for 46 IM residents. Two 2-hour sessions utilized a case-based, small-group, interactive scenario of a transgender woman requiring screening and treatment of sexually transmitted illnesses (STIs), pre-exposure prophylaxis (PrEP) prescription, and gender-affirming hormone therapy (GAHT) initiation. One-page handouts with step-by-step instructions were provided. Surveys evaluated knowledge, attitudes toward the importance of GSD care, and perceptions of confidence in performing specific skills related to GSD care. Wilcoxon signed-rank tests were used to compare pre- and postsession answers. **Results:** Residents' perceived confidence in providing GSD sexual health care and prescribing PrEP and GAHT increased after the sessions ($p < .001$). Attitudes regarding the importance of obtaining comprehensive sexual histories, discussing gender identity, assessing gender dysphoria, and offering GAHT for GSD patients also increased ($p < .001$). The median number of correct responses to five knowledge-based questions increased from 1 to 4 ($p < .001$). **Discussion:** This curriculum improved residents' perceptions of the importance of providing GSD care, as well as their knowledge and confidence in clinical skills related to STI screening and treatment, PrEP prescription, and GAHT initiation. This curriculum offers IM residents one of the first systematic frameworks, with immediately useable materials, to address GSD health.

Keywords
Gender and Sexual Diverse, Pre-Exposure Prophylaxis, PrEP, Gender Equity, Health Equity, Human Sexuality, LGBTQ+ Health, Internal Medicine, Case-Based Learning

Educational Objectives

By the end of this activity, learners will be able to:

1. Identify the differences between sex, gender identity, gender expression, and sexual orientation.
2. Obtain an inclusive medical history using language to avoid assumptions about gender and sexual diverse (GSD) patients.
3. Obtain a sexual history using the 7 P's: permission, partners, practices, protection from sexually transmitted illnesses (STIs), prior STIs, pleasure/pain, and pregnancy.
4. Describe the screening strategies for STIs using an inclusive approach.
5. Recognize the most up-to-date therapeutic regimens for gonorrhea and chlamydia.
6. Describe the indications, prescription regimens, and monitoring parameters for pre-exposure prophylaxis (PrEP) to prevent infection with human immunodeficiency virus (HIV).
7. Adapt an evidence-based eight-step approach on how to prescribe gender-affirming hormone therapy (GAHT) for patients with gender dysphoria.

Citation:
Suarez S, Lupton Lupez E, Modzelewski KL, Hinkle C, Streed CG Jr, Siegel J. A systematic and practical framework on gender and sexual diverse (GSD) health for internal medicine residents. *MedEdPORTAL*. 2025;21:11535. https://doi.org/10.15766/mep_2374-8265.11535

Introduction

Gender and sexual diverse (GSD) patients, including those who identify as lesbian, gay, bisexual, transgender, queer, and intersex (LGBTQI+), face significant health inequities.[1] These disparities are exacerbated by clinicians' insufficient knowledge,

Copyright © 2025 Suarez et al. This is an open-access publication distributed under the terms of the Creative Commons Attribution license.

comfort, and competency in providing care to GSD patients. GSD patients often have to educate health care providers about their own needs,[2,3] which deters them from engaging with the health care system.[4] In a 2022 US transgender survey, nearly half of transgender individuals reported having negative experiences with health care providers, including refusal of care, misgendering, verbal abuse, or physical mistreatment.[5]

Availability and content of medical school curricula on GSD health remain variable,[6,7] leading to differences in knowledge and perceived competency in caring for GSD patients.[8-11] A dearth of curricula in residency programs is also evident.[12-14] The educational gap is especially pronounced in transgender health and gender-affirming care,[15] with almost no published curricula specifically targeting internal medicine (IM) residents.[16,17] Since many IM residents will serve as primary care providers (PCPs) for GSD patients, addressing these gaps is crucial. The American College of Physicians recommends that IM residency programs "incorporate LGBT health issues into their curricula,"[18] but the ACGME has yet to provide specific guidelines, stating only that residents should meet the competency of "respect and responsiveness to diverse patient populations."[19] Given the lack of curricula in residency programs and limited faculty with expertise in GSD health, there has been a "call to action" for graduate medical education to enhance LGBTQI+ health education.[13]

Current published curricula tend to be siloed into individual topics, such as pre-exposure prophylaxis (PrEP) for men who have sex with men.[20] In *MedEdPORTAL*, most of the published curricula focused on GSD health are targeted to medical students, and are primarily introductory and didactic in format.[9,20-22] There are some curricula targeting IM residents, with one program focused on teaching sexual history taking and screening for sexually transmitted infections (STIs),[23] and another on health promotion and disease prevention for GSD patients.[24] These educational interventions involved mostly passive learning, such as lectures, and did not develop standardized clinical approaches.[9,12,21,22,25] Curricula using standardized patients (SPs) have focused on teaching sexual history taking[26] or assisting family medicine residents in formulating hormone-prescribing plans.[27] The only curriculum involving real patients had residents take sexual histories, provide counseling, and prescribe PrEP,[28] which increased the residents' self-reported confidence and comfort.

We previously conducted a needs assessment among IM residents at our institution that revealed varying levels of knowledge, comfort, and perceived competency in caring for GSD patients, while also identifying implementation facilitators, barriers, and preferred educational methods.[29] We designed the instructional approach of our curriculum based on the preferences reported by residents in our needs assessment. Residents specifically preferred small-group discussions and case-based learning, the former promoting critical thinking and participation,[30] and the latter encouraging self-directed learning and integration into practice.[31] Based on these survey results and considering the developmental stage of IM residents, we developed a novel, systematic, stepwise curriculum grounded in the World Professional Association for Transgender Health (WPATH) Standards of Care for Transgender and Gender Diverse People, version 8 (SOC-8)[32] guidelines and the AAMC competencies to address GSD health needs comprehensively. This curriculum emphasizes concrete, high-value skills tailored for busy residents, supported by a user-friendly handout as a practical reference for infrequent GSD patient encounters.

Methods

Setting

We developed a curriculum at Boston Medical Center (BMC), an urban safety-net hospital in Boston, Massachusetts. BMC is committed to delivering exceptional care, particularly to vulnerable populations, as evidenced by its GenderCare Center, which provides care to transgender and gender-diverse patients. Similarly, the IM residency program at BMC recruits residents with a strong social mission and a history of community engagement. Target learners consisted of residents in the IM residency program, with 46 residents in each postgraduate year over a span of 3 years.

Curriculum Design

The curriculum was collaboratively designed by a team of internists with diverse clinical and academic expertise in GSD health, primary care, medical education, infectious diseases, and endocrinology. We designed this curriculum using Kern's six-step approach.[33] First, we identified a lack of GSD health education in the residency program. An interest group was created among residents and faculty to discuss gaps and priorities. Then, we completed a targeted needs assessment,[29] which provided unique insights to implement a learner-centered and developmentally appropriate curriculum. We developed objectives using the AAMC competencies to create a systematic framework on practical skills to care for GSD patients. With our survey results, the educational strategy was selected based on the residents' preferences for small-group, case-based discussions during protected time. The 2-hour case-based, interactive sessions were implemented for an entire cohort of

Copyright © 2025 Suarez et al. This is an open-access publication distributed under the terms of the Creative Commons Attribution license.

IM residents. All participants completed pre- and postsession surveys that evaluated changes in knowledge and perceptions regarding GSD health care. Participants were asked for feedback on the sessions.

Through an iterative process, we selected learning objectives (Figure) based on our needs assessment results,[29] the AAMC Professional Competencies to Improve Health Care for People Who Are or May Be LGBT, Gender Nonconforming, and/or Born With DSD,[34] and prior work previously published in *MedEdPORTAL*.[24] The curricular content was specifically tailored for general internists and PCPs, who commonly serve as primary providers for GSD patients. The content to address the learning objectives was primarily derived and adapted from the Fenway Guide to Lesbian, Gay, Bisexual, and Transgender Health,[35] the CDC guidelines on taking a sexual history,[36,37] STIs,[38] and PrEP,[39] the WPATH SOC-8 (new compared to most curricula),[32] the University of California, San Francisco (UCSF) Guidelines for the Primary and Gender-Affirming Care of Transgender and Gender Nonbinary People,[40] and the Johns Hopkins Quick Guide to Hormone Therapy for the PCP.[41]

The first author (Sebastian Suarez) performed an extensive review of the literature and developed the framework used in this curriculum. Senior authors (Jennifer Siegel and Katherine L. Modzelewski) with expertise in GSD health and endocrinology further reviewed the curriculum for accuracy and clinical relevance. The curriculum consisted of two 2-hour sessions centered on a clinical scenario involving a transgender woman with diverse health care needs, including STI screening and treatment, PrEP prescription, and gender-affirming hormone therapy (GAHT) initiation. To foster an environment conducive to active learning and engagement, this partly learner-led curriculum was designed so that more than 50% of the time was allocated to activities such as small-group discussions, think-pair-share exercises, problem-based learning–style interactions, multiple-choice questions, and interactive activities. To aid learners and facilitate spaced learning and repetition, two concise one-page documents were created to include the framework (Appendix A and Appendix B), offering practical step-by-step instructions on how to address GSD sexual health and GAHT needs.

Curriculum Implementation

A step-by-step implementation strategy for this curriculum is included in the Facilitator Guide (Appendix C). We used PowerPoint (Microsoft Corporation, Redmond, Washington) to portray the curricular content and activity prompts using basic audiovisual equipment. The first 2-hour session (Appendix D) was conducted in February 2022 for all IM interns during academic half-day, a dedicated and mandatory educational block for all residents during their ambulatory weeks (BMC follows an X+Y scheduling model). We chose interns because the initial session covered topics essential to sexual health, which are universally applicable to all patients during routine annual examinations. The second 2-hour session (Appendix E), conducted in February and March 2023, targeted the same cohort of residents during their PGY-2, when they possessed foundational knowledge allowing discussions on gender dysphoria and GAHT. The Institutional Review Board at Boston University Medical Campus and BMC considered the study exempt since it constituted a routine educational activity within an established setting.

Facilitators were senior residents (Emily Lupton Lupez and Chad Hinkle) and a chief medical resident (Sebastian Suarez) at BMC, all with special interest in GSD health and experience in medical education. Sessions were attended by preceptors with expertise in GSD health and medical education (Jennifer Siegel and Katherine L. Modzelewski).

Evaluation

We evaluated each session with anonymous and voluntary 7-minute–long surveys (Appendix F and Appendix G) that explored knowledge, attitudes toward the importance of providing GSD care in primary care, and perceptions of confidence in performing specific skills related to GSD care. These surveys were collaboratively created by the lead author (Sebastian Suarez) and reviewed by faculty members specializing in GSD health (Jennifer Siegel) and endocrinology (Katherine L. Modzelewski) to ensure content validity. Due to the absence of validated questionnaires in the literature, we adapted questions from previous studies and modified to align with the curriculum's learning objectives.[20,24,42,43] Attitudes toward the importance of providing GSD care in the clinic and perceptions of confidence in performing specific skills related to GSD care were evaluated using 5-point Likert scales (ratings based on degree of importance [1 = *not important*, 5 = *extremely important*] and level of agreement [1 = *strongly disagree*, 5 = *strongly agree*]), with questions related to gender and sexuality, sexual health, PrEP prescription, and initiation of GAHT. Knowledge of STI screening, PrEP, and GAHT was assessed through multiple-choice questions. Feedback was requested with open-ended questions. Presession surveys were emailed to residents 1 week before each session, with reminders sent 2 days prior to each session. To increase response rates, a QR code was displayed on screen before the session began. At the end of the session, another QR code was shown with identical postsession surveys.

Copyright © 2025 Suarez et al. This is an open-access publication distributed under the terms of the Creative Commons Attribution license.

AAMC Domain	AAMC Competency	Learning Objectives and/or Teaching Points Across Our Curriculum
Patient Care	1. Sensitively and effectively eliciting relevant information about sex anatomy, sex development, sexual behavior, sexual history, sexual orientation, sexual identity, and gender identity from all patients in a developmentally appropriate manner.	- Identify the differences between sex, gender identity, gender expression, and sexual orientation
	3. Describing the special health care needs and available options for quality care for transgender patients and for patients born with DSD (e.g., specialist counseling, pubertal suppression, elective and nonelective hormone therapies, elective and nonelective surgeries, etc.).	- Describe the screening strategies for STIs using an inclusive approach and recognize the most up-to-date therapeutic regimens for gonorrhea and chlamydia<br>- Describe the indications, prescription regimens, and monitoring parameters for PrEP to prevent infection with HIV<br>- Adapt an evidence-based eight-step approach on how to prescribe GAHT for patients with gender dysphoria
	4. Assessing unique needs and tailoring the physical exam and counseling and treatment recommendations to any of the individuals described above, taking into account any special needs, impairments, or disabilities.	
	5. Recognizing the unique health risks and challenges often encountered by the individuals described above, as well as their resources, and tailoring health messages and counseling efforts to boost resilience and reduce high-risk behaviors.	
	6. Providing effective primary care and anticipatory guidance by utilizing screening tests, preventive interventions, and health care maintenance for the populations described above.	
Knowledge for Practice	1. Defining and describing the differences among: sex and gender; gender expression and gender identity; gender discordance, gender nonconformity, and gender dysphoria; and sexual orientation, sexual identity, and sexual behavior.	- Identify the differences between sex, gender identity, gender expression, and sexual orientation<br>- Identify how to assess and document gender dysphoria (or incongruence)
Interpersonal and Communication Skills	1. Developing rapport with all individuals (patient, families, and/or members of the health care team) regardless of others' gender identities, gender expressions, body types, sexual identities, or sexual orientations, to promote respectful and affirming interpersonal exchanges, including by staying current with evolving terminology.	- Discuss how to take an inclusive medical history and use language to avoid assumptions about GSD patients<br>- Identify the differences between sex, gender identity, gender expression, and sexual orientation
	3. Understanding that implicit (i.e., automatic or unconscious) bias and assumptions about sexuality, gender, and sex anatomy may adversely affect verbal, nonverbal, and/or written communication strategies involved in patient care, and engaging in effective corrective self-reflection processes to mitigate those effects.	- Discuss how to take an inclusive medical history and use language to avoid assumptions about GSD patients
	4. Identifying communication patterns in the health care setting that may adversely affect care of the described populations, and learning to effectively address those situations in order to protect patients from the harmful effects of implicit bias or acts of discrimination.	- Discuss how to take an inclusive medical history and use language to avoid assumptions about GSD patients
Professionalism	2. Recognizing the unique aspects of confidentiality regarding gender, sex, and sexuality issues, especially for the patients described above, across the developmental spectrum, and by employing appropriate consent and assent practices.	- Identify and interpret the seven Ps framework to obtain a sexual history: permission, partners, practices, protection from STIs, prior STIs, pleasure/pain, and pregnancy
Interprofessional Collaboration	Valuing the importance of interprofessional communication and collaboration in providing culturally competent, patient-centered care to the individuals described above and participating effectively as a member of an interdisciplinary health care team.	- Discuss when to refer patients to specialists, including psychiatry and endocrinology, whenever there are questions regarding gender-affirming hormone therapy

Figure. Learning objectives linked to the AAMC Competencies to Improve Health Care for People Who Are or May Be LGBT, Gender Nonconforming, and/or Born With DSD. Abbreviations: DSD, differences of sexual development; STI, sexually transmitted infection; PrEP, pre-exposure prophylaxis; GAHT, gender-affirming hormone therapy; GSD, gender and sexual diverse.

Copyright © 2025 Suarez et al. This is an open-access publication distributed under the terms of the Creative Commons Attribution license.

Statistical Analysis
Descriptive statistics were analyzed using the RStudio program. As data were non-normally distributed, a Wilcoxon signed-rank test was employed to compare pre- and postsession answers. Reliability was not evaluated given the small number of respondents per item. Responses to open-ended questions were analyzed using content and theme analysis, determining the frequency and percentage of responses in each theme.

Results

Of the 46 eligible IM residents, 42 (91%) participated in the first session, of whom 41 (98%) completed the presession survey and 34 (81%) completed the postsession survey. Additionally, 41 residents (89%) participated in the second session, of whom 39 (95%) completed the presession survey and 40 (98%) completed the postsession survey. Four residents (9%) were in the primary care track, while three residents (6%) did not disclose their track, and the rest were in the categorical track. Nine residents (22%) identified as lesbian, gay, bisexual, transgender, or queer or as a GSD individual.

On the presession survey from the first session, when participants were asked if they routinely take a comprehensive sexual history, 11 respondents (27%) disagreed, 19 (46%) were neutral, 11 (27%) agreed, and 1 (2.4%) did not respond. When asked if they feel more comfortable discussing sexual history with a cisgender, heterosexual patient than with a GSD patient, 8 respondents (20%) disagreed, 19 (46%) were neutral, 14 (34%) agreed, and 1 (2.4%) did not respond.

Outcome data represented Kirkpatrick level 2 evaluation of learning. From pre- to postsession, there was a significant increase in Likert-scale scores regarding the residents' perception, as a PCP, of the importance of taking a GSD patient's comprehensive sexual history and discussing gender identity, assessing gender dysphoria, and offering GAHT to GSD individuals (Table 1). Similarly, after the sessions, there was a significant increase in residents' confidence in their ability to adequately care for GSD patients.

When the IM residents completed the knowledge-based, multiple-choice portion of the surveys, the pre- and postsession median scores remained unchanged across the five questions. However, the Wilcoxon signed-rank test showed statistically significant changes in median scores after the sessions (Table 2). The median number of correct responses to the five knowledge-based questions increased from 1 to 4 ($p < .001$; Table 2). This suggests that while the central tendency did not change,

Table 1. Participant Likert-Scale Scores on Attitudes Regarding the Importance of Providing GSD Care in Primary Care and Perceptions of Confidence in Performing Specific Skills Related to GSD Care

	Mdn (IQR)		
**Question/Statement**	**Pre**	**Post**	***p***
How important do you feel it is[a]:			
For you to discuss pronouns and gender identity with your primary care patients?	4 (3-4)	4 (4-5)	<.001
To take a comprehensive sexual history with your primary care patients?	4 (4-4)	4 (4-5)	.001
To routinely discuss PrEP for HIV with high sexual risk patients?	4 (4-5)	5 (4-5)	.16
To assess for gender dysphoria/ incongruence in TGD patients?	4 (4-5)	4 (4-5)	.003
To offer transition-related care for TGD patients in your primary care clinic vs. referring to a specialized clinic?	4 (3-5)	4.5 (4-5)	<.001
To prescribe GAHT for TGD patients as a PCP?	4 (3-4)	4 (4-5)	<.001
I feel confident in my abilities to[b]:			
Discuss gender identity and sexual orientation with my patients.	3 (3-4)	4 (4-4)	<.001
Use inclusive language during patient encounters.	4 (3-4)	4 (4-5)	.001
Take a comprehensive sexual history.	4 (3-4)	4 (4-5)	.003
Perform comprehensive STI screening (including appropriate swabs for different sites).	3 (2-4)	4 (4-5)	<.001
Prescribe HIV PrEP when indicated and monitor for side effects	2 (2-3)	4 (3.75-4)	<.001
Assess for gender dysphoria/incongruence	3 (2-3)	4 (4-4)	<.001
Explain the physical changes and the impact on fertility when prescribing GAHT	2 (2-3)	4 (4-4)	<.001
Prescribe GAHT	2 (1-2)	4 (3-4)	<.001
Monitor the side effects and hormone target levels after initiation of GAHT	2 (1-2)	4 (4-4)	<.001

Abbreviations: GSD, gender and sexual diverse; PrEP, pre-exposure prophylaxis; HIV, human immunodeficiency virus; TGD, transgender and gender diverse; GAHT, gender affirming hormone therapy; PCP, primary care provider; STI, sexually transmitted infection.
[a]Rated based on a 5-point Likert scale (1 = *not important*, 5 = *extremely important*).
[b]Rated based on a 5-point Likert scale (1 = *strongly disagree*, 5 = *strongly agree*).

Table 2. Median Number of Participant Correct Answers to Knowledge Questions on GSD Health

	Mdn		
**Content of Knowledge Questions**	**Pre**	**Post**	***p***
Session 1			
STI screening in MSM	0	1	.01
STI risk in WSW	0	1	.06
PrEP eligibility	0	1	<.001
PrEP prescription	0	1	<.001
PrEP monitoring	0	1	<.001
Total score (IQR)[a]	1 (1-2)	4 (3-5)	<.001
Session 2			
Initial evaluation of TGD patients to start GAHT	0	1	<.001
Contraindication for GHAT	0	1	<.001
Baseline labs for initial assessment	0	1	<.001
Estrogen-centered hormone therapy	0	1	<.001
Testosterone hormone therapy	0	0	<.001
Total score (IQR)[a]	1 (0-2)	4 (4-5)	<.001

Abbreviations: GSD, gender and sexual diverse; STI, sexually transmitted infection; MSM, men who have sex with men; WSW, women who have sex with women; PrEP, pre-exposure prophylaxis; TGD, transgender and gender-diverse; GAHT, gender-affirming hormone therapy.
[a]Total median number of correct answers out of five questions.

Copyright © 2025 Suarez et al. This is an open-access publication distributed under the terms of the Creative Commons Attribution license.

individual experiences increased toward the median, and the variance decreased.

For the first and second sessions, 97% and 100% of residents, respectively, either agreed or strongly agreed that the sessions were clearly presented and well organized, and that the instructors were knowledgeable and prepared.

When asked to rate the utility of the session as IM residents, 24 residents (71%) rated the first session as *excellent* and 8 (24%) as *very good*. All residents either strongly agreed or agreed that the content of the first session would improve their communication with GSD patients. For the second session, 19 (48%) rated it as *excellent* and 19 (48%) as *very good*, while 1 (3%) rated the session as *average* and 1 (3%) as *poor*. Among the survey respondents, 26 (65%) strongly agreed, 13 (33%) agreed, and 1 (3%) strongly disagreed that the second session would improve their ability to prescribe GAHT for transgender and gender-diverse patients.

Through open-ended responses, residents suggested improvements, including adding more interactive components, cases, and questions; receiving the handout prior to the session; involving faculty with more patient care experience; and incorporating real patients to apply what was learned.

Discussion

After implementing this evidence-based, practical step-by-step approach on GSD health for IM residents, there was an immediate increase in residents' perception of the importance of providing GSD care, as well as in their knowledge and confidence in clinical skills related to STI screening and treatment, PrEP prescription, and GAHT initiation. Before the curriculum, most IM residents did not feel comfortable discussing GSD health issues with their patients, and many felt either neutral or more comfortable discussing these topics with cisgender, heterosexual patients than with GSD patients. Even in our program with faculty expertise and high rates of residents self-identifying as GSD, residents had poor knowledge of and discomfort with GSD care, suggesting a similar baseline state as that of residents elsewhere. One advantage of implementation was the lack of resistance from leadership, along with residents' high awareness, which helped minimize biases during the session.

One of our key successes was the improvement in residents' confidence in both their communication skills and their ability to prescribe PrEP and GAHT for GSD patients. We believe this could be partly attributed to the instructional approach that aligned with the needs assessment and focused on practical application of knowledge. By focusing on both communication skills and medical management of GSD health, we equipped residents with essential skills to provide holistic and compassionate care to GSD patients. Similarly, we provided residents with a user-friendly handout that served as a consolidated resource and framework for patient encounters, enabling them to focus on understanding the framework rather than memorizing it. Anecdotally, residents reached out to the instructors to share that they were using the framework and handout to prescribe PrEP and monitor GAHT in their clinic, and that the sessions were pivotal in empowering them to provide GSD care without referring to specialty clinics. Although anecdotal, it suggests some durability to the curriculum and reflects the benefit of providing materials that residents could put to immediate use in clinic. Some residents even asked if they could share the handout with people outside the institution.

This curriculum was included in mandatory educational blocks for all residents, which likely contributed to the high response rates, enhancing the internal validity of our study. As the curriculum was implemented by a chief medical resident and senior residents with interest in the topic, it potentially allows for other training programs to adapt the implementation. Similarly, our curriculum serves as a practical resource to support situations where cost or feasibility (e.g., absence of a GSD health–centered clinic or infrequent patient encounters related to GSD health) prevent the implementation of other educational strategies. If adapted at institutions with less diversity of expertise, we suggest inviting a patient who identifies as GSD to share their story about discrimination in the health care system as well as in other aspects of their lives, which could foster empathy and enhance learning. Residents in settings without GSD experts can leverage online resources like the WPATH or UCSF guidelines, collaborate with interdisciplinary teams including social workers and psychologists who may have additional resources, and refer to endocrinologists who are more likely to have the necessary training to support GSD patients.

This curriculum has limitations. Since there were no validated questionnaires available when the curriculum was created, we used prior study surveys as references.[24] Future iterations should refine the questionnaire to enhance question quality and align with best practices, such as avoiding negatives and ensuring choices are mutually exclusive. Additionally, we intentionally limited survey length to encourage participation and completion rates. We did not include doxycycline postexposure prophylaxis (doxy PEP) since the CDC guidelines were published after our

Copyright © 2025 Suarez et al. This is an open-access publication distributed under the terms of the Creative Commons Attribution license.

curriculum was developed. However, the current curriculum is adaptable to updates in best practices, such as doxy PEP. Future iterations should incorporate more multiple-choice questions and interactive components throughout the sessions. Similar to the experience of others working in GSD health issues,[27] we now recognize that using terms like "masculinizing" and "feminizing" are not specific and may perpetuate gender stereotypes. We recommend using gender-neutral language (e.g., testosterone therapy and estrogen-centered therapy) instead of language focused on the effect that hormones may have on a patient's body.

As a single-site study with a gender care center, the findings should be generalized with caution and adapted to local contexts that have different expertise in this subject. Delivering this curriculum without prior GSD health experience may be challenging. To support implementation, we included a detailed facilitator guide. Future efforts could include the development of facilitator trainings or a CME module. The short-term nature of our assessment made it difficult to evaluate whether the changes in our outcomes were sustained with time. Furthermore, we did not measure whether the curriculum impacted residents' skills, practices, or GAHT prescription behaviors, although we did hear anecdotal reports from residents that were encouraging. The impact could be further studied with qualitative research that evaluates the residents' experiences of using the framework and handout in their clinical encounters.

In the future, this curriculum could be expanded to include additional modules focused on surgical options and preventive health of GSD patients. It could also include SPs or direct observation in a clinic dedicated to GSD patients, as has been done in other studies.[26-28,44,45] Given the robust evidence supporting the use of SPs in other areas of medical education, their inclusion in future GSD health curricula should be a priority. Curriculum designers have an opportunity to actively engage GSD SPs and ensure appropriate compensation when doing so. By distributing this curriculum across the US, we can provide targeted and practical GSD health education to a diversity of IM and family medicine residency programs. Lastly, this curriculum can be used to train faculty who may currently lack knowledge in this area, so that they can better support learners and care for GSD patients. A "train the trainer" approach, as employed previously,[24] could be particularly effective in achieving this goal.

In conclusion, this evidence-based, practical framework increased IM residents' knowledge, confidence in GSD health clinical skills, and perception of the importance of GSD care. The curriculum, grounded in the AAMC competencies, along with a user-friendly handout, could provide a quick and practical resource for real-life patient encounters.

Appendices

A. GSD Health Handout.pptx
B. GAHT Handout.pptx
C. Facilitator Guide.docx
D. GSD Health - Part 1.pptx
E. GSD Health - Transgender Health.pptx
F. GSD Health Survey.docx
G. TGD Health Survey.docx

All appendices are peer reviewed as integral parts of the Original Publication.

Sebastian Suarez, MD, MPH: Associate Program Director, UM/JMH Internal Medicine Residency Program, University of Miami Miller School of Medicine; ORCID: https://orcid.org/0000-0001-6339-8675

Emily Lupton Lupez, MD, MS, MPH: Assistant Professor of Medicine, Section of General Internal Medicine, Department of Medicine, Boston University Chobanian and Avedisian School of Medicine

Katherine L. Modzelewski, MD: Associate Program Director, Internal Medicine Residency Program, Department of Medicine, Boston University Medical Center

Chad Hinkle, MD: First-Year Fellow, Section of Infectious Diseases and Global Health, Department of Medicine, The University of Chicago

Carl G. Streed Jr., MD, MPH: Associate Professor of Medicine, Section of General Internal Medicine, Department of Medicine, Boston University Chobanian and Avedisian School of Medicine; Research Lead, Gender Care Center, Boston Medical Center

Jennifer Siegel, MD: Medical Director, Transgender Health Program; Primary Care Program Director, Internal Medicine Residency Program, Massachusetts General Hospital

Acknowledgments
We would like to acknowledge and thank Dr. Alan Chan and Dr. Jorge Nicolas Ruiz-Lopez for their contributions during the initial discussions of this project. We would also like to thank Dr. Alexandra Bachorik for precepting the sessions and contributing to the nuances of sexually transmitted infection treatment in pregnant patients.

Disclosures
None to report.

Funding/Support
None to report.

Copyright © 2025 Suarez et al. This is an open-access publication distributed under the terms of the Creative Commons Attribution license.

Prior Presentations

Suarez S, Lupton Lupez E, Hinkle C, Modzelewski K, Streed C, Siegel J. A systematic and practical framework on gender and sexual diverse (GSD) health for internal medicine residents. Poster presented at: The Academic Alliance for Internal Medicine Academic Internal Medicine Week 2025 (AIMW25); April 2025; New Orleans, LA.

Suarez S, Lupez EL, Modzelewski KL, Hinkle C, Streed C, Siegel J. A systematic and practical framework on gender and sexual diverse (GSD) health for internal medicine residents. Poster presented at: The Society of General Internal Medicine (SGIM) 2025 Annual Meeting; May 2025; Hollywood, FL.

Ethical Approval

The Boston University Medical Campus and Boston Medical Center Institutional Review Board reviewed this project.

References

1. *Understanding the Well-Being of LGBTQI+ Populations*. National Academies of Sciences, Engineering, and Medicine; 2020.
2. *When Health Care Isn't Caring: Lambda Legal's Survey of Discrimination Against LGBT People and People with HIV*. Lambda Legal; 2010. https://www.lambdalegal.org/sites/default/files/publications/downloads/whcic-report_when-health-care-isnt-caring.pdf
3. James SE, Herman JL, Rankin S, Keisling M, Mottet L, Anafi M. *Executive Summary: The Report of the 2015 U.S. Transgender Survey*. National Center for Transgender Equality; 2016.
4. *Discrimination in America: Experiences and Views of LGBTQ Americans*. National Public Radio, Robert Wood Johnson Foundation, Harvard T.H. Chan School of Public Health; 2017.
5. James SE, Herman JL, Durso LE, Heng-Lehtinen R. *Early Insights: A Report of the 2022 U.S. Transgender Survey*. National Center for Transgender Equality; 2024.
6. Obedin-Maliver J, Goldsmith ES, Stewart L, et al. Lesbian, gay, bisexual, and transgender-related content in undergraduate medical education. *JAMA*. 2011;306(9):971-977. https://doi.org/10.1001/jama.2011.1255
7. Streed CG Jr, Michals A, Quinn E, et al. Sexual and gender minority content in undergraduate medical education in the United States and Canada: current state and changes since 2011. *BMC Med Educ*. 2024;24(1):482. https://doi.org/10.1186/s12909-024-05469-0
8. Streed CG Jr., Lunn MR, Siegel J, Obedin-Maliver J. Meeting the patient care, education, and research missions: academic medical centers must comprehensively address sexual and gender minority health. *Acad Med*. 2021;96(6):822-827. https://doi.org/10.1097/ACM.0000000000003703
9. Weingartner L, Noonan EJ, Bohnert C, Potter J, Shaw MA, Holthouser A. Gender-affirming care with transgender and genderqueer patients: a standardized patient case. *MedEdPORTAL*. 2022;18:11249. https://doi.org/10.15766/mep_2374-8265.11249
10. Beltran CP, Wilhite JA, Hayes RW, et al. Practice makes perfect: objective structured clinical examinations across the UME-to-GME continuum improve care of transgender simulated patients. *J Grad Med Educ*. 2024;16(2):182-194.
11. Jewell TI, Petty EM. LGBTQ+ health education for medical students in the United States: a narrative literature review. *Med Educ Online*. 2024;29(1):2312716. https://doi.org/10.1080/10872981.2024.2312716
12. Streed CG Jr, Hedian HF, Bertram A, Sisson SD. Assessment of internal medicine resident preparedness to care for lesbian, gay, bisexual, transgender, and queer/questioning patients. *J Gen Intern Med*. 2019;34(6):893-898. https://doi.org/10.1007/s11606-019-04855-5
13. Pregnall AM, Churchwell AL, Ehrenfeld JM. A call for LGBTQ content in graduate medical education program requirements. *Acad Med*. 2021;96(6):828-835. https://doi.org/10.1097/ACM.0000000000003581
14. Mains-Mason JB, Ufomata E, Peebles JK, et al. Knowledge retention and clinical skills acquisition in sexual and gender minority health curricula: a systematic review. *Acad Med*. 2022;97(12):1847-1853. https://doi.org/10.1097/ACM.0000000000004768
15. Dubin SN, Nolan IT, Streed CG Jr, Greene RE, Radix AE, Morrison SD. Transgender health care: improving medical students' and residents' training and awareness. *Adv Med Educ Pract*. 2018;9:377-391. https://doi.org/10.2147/AMEP.S147183
16. Cooper RL, Ramesh A, Radix AE, et al. Affirming and inclusive care training for medical students and residents to reduce health disparities experienced by sexual and gender minorities: a systematic review. *Transgend Health*. 2023;8(4):307-327. https://doi.org/10.1089/trgh.2021.0148
17. Borowicz C, Daniel L, Futcher RD, Wilfong DN. Transgender and non-binary patient simulations can foster cultural sensitivity and knowledge among internal medicine residents: a pilot study. *Adv Simul (Lond)*. 2024;9(1):12. https://doi.org/10.1186/s41077-024-00284-5
18. Daniel H, Butkus R; Health and Public Policy Committee of American College of Physicians. Lesbian, gay, bisexual, and transgender health disparities: executive summary of a policy position paper from the American College of Physicians. *Ann Intern Med*. 2015;163(2):135-137. https://doi.org/10.7326/M14-2482
19. *ACGME Program Requirements for Graduate Medical Education in Internal Medicine*. Accreditation Council for Graduate Medical Education. Updated July 1, 2023. Accessed May 2, 2025. https://www.acgme.org/globalassets/pfassets/programrequirements/140_internalmedicine_2023.pdf
20. Perucho J, Alzate-Duque L, Bhuiyan A, Sánchez JP, Sánchez NF. PrEP (Pre-Exposure Prophylaxis) education for clinicians: caring

Copyright © 2025 Suarez et al. This is an open-access publication distributed under the terms of the Creative Commons Attribution license.

for an MSM patient. *MedEdPORTAL*. 2020;16:10908. https://doi.org/10.15766/mep_2374-8265.10908

21. Roth LT, Friedman S, Gordon R, Catallozzi M. Rainbows and "ready for residency": integrating LGBTQ health into medical education. *MedEdPORTAL*. 2020;16:11013. https://doi.org/10.15766/mep_2374-8265.11013
22. Cooper MB, Chacko M, Christner J. Incorporating LGBT health in an undergraduate medical education curriculum through the construct of social determinants of health. *MedEdPORTAL*. 2018;14:10781. https://doi.org/10.15766/mep_2374-8265.10781
23. Maldonado ME, Rusiecki J. An advanced, risk-driven sexual health curriculum for first-year internal medicine residents. *MedEdPORTAL*. 2022;18:11287. https://doi.org/10.15766/mep_2374-8265.11287
24. Ufomata E, Eckstrand KL, Spagnoletti C, et al. Comprehensive curriculum for internal medicine residents on primary care of patients identifying as lesbian, gay, bisexual, or transgender. *MedEdPORTAL*. 2020;16:10875. https://doi.org/10.15766/mep_2374-8265.10875
25. Roth LT, Catallozzi M, Soren K, Lane M, Friedman S. Bridging the gap in graduate medical education: a longitudinal pediatric lesbian, gay, bisexual, transgender, queer/questioning health curriculum. *Acad Pediatr*. 2021;21(8):1449-1457. https://doi.org/10.1016/j.acap.2021.05.027
26. Mayfield JJ, Ball EM, Tillery KA, et al. Beyond men, women, or both: a comprehensive, LGBTQ-inclusive, implicit-bias-aware, standardized-patient-based sexual history taking curriculum. *MedEdPORTAL*. 2017;13:10634. https://doi.org/10.15766/mep_2374-8265.10634
27. Hersh BJ, Rdesinski RE, Milano C, Cantone RE. An effective gender-affirming care and hormone prescribing standardized patient case for residents. *MedEdPORTAL*. 2022;18:11258. https://doi.org/10.15766/mep_2374-8265.11258
28. Frasca K, Castillo-Mancilla J, McNulty MC, et al. A mixed methods evaluation of an inclusive sexual history taking and HIV prevention curriculum for trainees. *J Gen Intern Med*. 2019;34(7):1279-1288. https://doi.org/10.1007/s11606-019-04958-z
29. Suarez S, Lupez E, Demers L, Streed CG Jr, Siegel J. Gender and sexual diverse health education: a needs assessment among internal medicine residents. *LGBT Health*. 2022;9(8):589-594. https://doi.org/10.1089/lgbt.2022.0058
30. Steinert Y. Student perceptions of effective small group teaching. *Med Educ*. 2004;38(3):286-293. https://doi.org/10.1046/j.1365-2923.2004.01772.x
31. Thistlethwaite JE, Davies D, Ekeocha S, et al. The effectiveness of case-based learning in health professional education. A BEME systematic review: BEME guide no. 23. *Med Teach*. 2012;34(6):e421-444. https://doi.org/10.3109/0142159X.2012.680939
32. Coleman E, Radix AE, Bouman WP, et al. Standards of care for the health of transgender and gender diverse people, version 8. *Int J Transgend Health*. 2022;23(suppl 1):S1-S259. https://doi.org/10.1080/26895269.2022.2100644
33. Thomas P, Kern D, Hughes M, Chen B. *Curriculum Development for Medical Education: A Six-Step Approach*. 3rd ed. Johns Hopkins University Press; 2016.
34. Hollenbach AD, Eckstrand KL, Dreger A, eds; AAMC Advisory Committee on Sexual Orientation GI, and Sex Development. *Implementing Curricular and Institutional Climate Changes to Improve Health Care for Individuals Who are LGBT, Gender Nonconforming, or Born with DSD*. Association of American Medical Colleges; 2014. Accessed May 2, 2025. https://store.aamc.org/downloadable/download/sample/sample_id/129/
35. Makadon HJ, Mayer KH, Potter J, Goldhammer H. *Fenway Guide to Lesbian, Gay, Bisexual, and Transgender Health*. 2nd ed. American College of Physicians; 2015.
36. *A Guide to Taking a Sexual History*. Centers for Disease Control and Prevention; 2005. https://stacks.cdc.gov/view/cdc/12303
37. Suarez S, Lupez E, Siegel J, Streed C Jr. The annual examination for lesbian, gay, and bisexual patients. primary care: clinics in office practice. 2021;48(2):191-212. https://doi.org/10.1016/j.pop.2021.02.001
38. *Sexually Transmitted Diseases Treatment Guidelines, 2021*. Centers for Disease Control and Prevention; 2021. Accessed May 2, 2025. https://www.cdc.gov/std/treatment-guidelines/STI-Guidelines-2021.pdf
39. *Preexposure Prophylaxis for the Prevention of HIV Infection in the United States—2021 Update: A Clinical Practical Guideline*. Centers for Disease Control and Prevention; 2021. Accessed May 2, 2025. https://stacks.cdc.gov/view/cdc/112360
40. Duetsch MB, ed. *Guidelines for the Primary and Gender-Affirming Care of Transgender and Gender Nonbinary People*. 2nd ed. UCSF Center of Excellence for Transgender Health; 2016. Accessed May 2, 2025. https://www.google.com/url?sa=t&source=web&rct=j&opi=89978449&url=https://transcare.ucsf.edu/sites/transcare.ucsf.edu/files/Transgender-PGACG-6-17-16.pdf&ved=2ahUKEwjMq7uQ0YuNAxXkGlkFHRLKB38QFnoECCAQAQ&usg=AOvVaw3yJnmDNIoM7o7fnJ1TJkaH
41. Hedian HF, Norwood A, Siegel J, Loeb D. *Transgender and Gender Diverse Hormone Therapy*. Updated January 2023. Accessed May 2, 2025. https://www.hopkinsmedicine.org/-/media/center-for-transgender-health/documents/tgd-gaht-quick-guide.ashx
42. Gallego J, Knudsen J. LGBTQI' defined: an introduction to understanding and caring for the queer community. *MedEdPORTAL*. 2015;11:10189. https://doi.org/10.15766/mep_2374-8265.10189
43. Sawning S, Steinbock S, Croley R, Combs R, Shaw A, Ganzel T. A first step in addressing medical education curriculum gaps in lesbian-, gay-, bisexual-, and transgender-related content: The

Copyright © 2025 Suarez et al. This is an open-access publication distributed under the terms of the Creative Commons Attribution license.

University of Louisville Lesbian, Gay, Bisexual, and Transgender Health Certificate Program. *Educ Health (Abingdon)*. 2017;30(2):108-114. https://doi.org/10.4103/efh.EfH_78_16

44. Kreines FM, Quinn GP, Cardamone S, et al. Training clinicians in culturally relevant care: a curriculum to improve knowledge and comfort with the transgender and gender diverse population. *J Assist Reprod Genet*. 2022;39(12):2755-2766. https://doi.org/10.1007/s10815-022-02655-1

45. Stevenson MO, Sineath RC, Haw JS, Tangpricha V. Use of standardized patients in endocrinology fellowship programs to teach competent transgender care. *J Endocr Soc*. 2020;4(1):bvz007. https://doi.org/10.1210/jendso/bvz007

Received: January 7, 2025
Accepted: April 21, 2025
Published: June 17, 2025

Copyright © 2025 Suarez et al. This is an open-access publication distributed under the terms of the Creative Commons Attribution license.
